# Development and piloting of a survey to estimate the frequency and nature of potentially harmful preventable problems in primary care from a UK patient’s perspective

**DOI:** 10.1136/bmjopen-2017-017786

**Published:** 2018-02-03

**Authors:** Susan J Stocks, Ailsa Donnelly, Aneez Esmail, Joanne Beresford, Carolyn Gamble, Sarah Luty, Richard Deacon, Avril Danczak, Nicola Mann, David Townsend, James Ashley, Paul Bowie, Stephen M Campbell

**Affiliations:** 1 NIHR Greater Manchester Primary Care Patient Safety Translational Research Centre, Centre for Primary Care, Division of Population Health, Health Services Research and Primary Care, University of Manchester, Manchester, UK; 2 Research User Group (RUG) of the NIHR Greater Manchester Primary Care Patient Safety Translational Research Centre, Centre for Primary Care,Division of Population Health, Health Services Research and Primary Care, University of Manchester, Manchester, UK; 3 General Practitioner NHS Greater Glasgow and Clyde, Medical Directorate, NHS Education for Scotland, Glasgow, Scotland; 4 St Gabriels Medical Centre, Manchester, UK; 5 Central and South Manchester Specialty Training Programme for General Practice, Health Education England North West (HEENWE) Education and Research Centre, Wythenshawe Hospital, Manchester, UK; 6 Woodlands Dental Practice, Wirral, UK; 7 Medical Directorate, NHS Education for Scotland, Glasgow, UK; 8 Institute of Health and Wellbeing, University of Glasgow, Glasgow, UK

**Keywords:** primary Care, quality in health care

## Abstract

**Objectives:**

To design and pilot a survey to be used at the population level to estimate the frequency of patient-perceived potentially harmful preventable problems occurring in UK primary care. To explore the nature of the problems, patient-suggested strategies for prevention and opinions of clinicians and the public regarding the potential for harm.

**Design:**

A survey was codesigned by three members of the public and one researcher and piloted through public and patient involvement and engagement networks.

**Setting:**

Self-selected sample of the UK population.

**Participants:**

977 members of the public accessed the online survey during October and November 2015.

**Primary outcome measures:**

Respondent feedback about the ease of completion of the survey, quality of responses in terms of review by clinicians and members of the public, preliminary estimates of the frequency and nature of patient-perceived potentially harmful problems occurring in the last 12 months.

**Results:**

638 (65%) members of the public completed the survey and few respondents reported any difficulty in understanding or completing the survey. 132 (21%) respondents reported experiencing a potentially harmful preventable problem during the past 12 months and 108 (82%) of these respondents provided a description that was adequate for at least one clinician to form an opinion about the potentially harmful problem. Respondents were older than the UK generally, more likely to work or volunteer in the healthcare sector and tended to use primary care more frequently but their confidence and trust in their own general practitioner (GP) was similar to that of the UK population as measured by the annual English GP patient survey.

**Conclusions:**

The survey was acceptable to patients and mostly provided data of sufficient quality for review by clinicians and members of the public. It is now ready to use at a population level to estimate the frequency and nature of potentially harmful preventable problems in primary care from a patient’s perspective.

**Strengths and limitations of this study**

- We have designed and tested a survey to measure the frequency and nature of potentially harmful preventable problems in primary care from the patient’s perspective.
- The survey was codesigned by three members of the public and piloted through extensive public and patient involvement (PPI).
- The patient-described scenarios were reviewed by primary care clinicians.
- The study respondents were self-selected through PPI and engagement groups.
- The survey is ready to be administered to a representative sample of the general population.

## Background

Patients are thought to take a different view of patient safety to healthcare professionals.^1^ They tend to view safety in terms of the overall balance of benefit and harm over time whereas healthcare professionals often see high-quality healthcare occasionally punctuated by safety incidents and adverse events.^2^ Furthermore, patients may hold different opinions about how to improve patient safety^3 4^ or different priorities to clinicians, for example, identifying psychological and emotional harm rather than technical errors.^5^ Involving patients in identifying errors and reducing harm occurs in secondary care,^6^ but patient-reported outcomes can show poor concordance between patients and clinicians, for example, in reporting adverse symptom events in the context of drug safety.^7^ Nonetheless, patients are thought to be capable of reporting medical errors accurately.^6 8^ Involving patients is advocated as a way to improve safety,^9^ and this approach would be facilitated through patients and professionals having an understanding of each other’s expectations and priorities.

Studies that quantify patient safety problems in primary care are uncommon and incidence estimates from record review or incident reporting by clinicians range from less than 1 to 24 per 100 consultations or record review.^10–12^ The National Reporting and Learning System in England and Wales records patient safety incidents reported by healthcare professionals; only 1% of these reports originate from primary care^13^ which likely reflects under-reporting.^14 15^ Still fewer studies have quantified patient safety problems in primary care from the patient’s perspective.^16^ A 2013 European survey of the UK public reported that 43% of respondents felt that it was ‘likely’ that patients could be harmed by non-hospital healthcare, an increase from 37% in 2009.^17^ In Norway, a population-level survey found that the patient-reported lifetime probability of ever experiencing an adverse event was 10%, of which around two-thirds of respondents attributed the cause of their event as their general practitioner (GP).^4^ In Spain, a telephone survey of patients estimated that around 7% of patients experienced a self-reported adverse event during a 1-year period.^18^ A USA practice-based website observed an incidence rate of patient-reported adverse events of 1.4% over 2 years.^19^ Data from the UK are sparse; this may be partly due to the lack of a valid and reliable instrument to make a comprehensive measurement of safety in primary care.^20^ The Patient Reported Experiences and Outcomes of Safety in Primary Care questionnaire should help to address this knowledge gap.^21 22^

Although it is acknowledged that patients tend to take a different view to professionals,^1 2^ most research into patient safety is initiated by clinicians with patients invited to contribute. We choose to take an alternative approach whereby the study design was conceived, designed and implemented by a team of three members of the public and one researcher with primary care professionals being invited to contribute later. Previous work has shown that patient-initiated surveys can provide meaningful feedback and guide improvements.^23^ Our aim was to design a survey asking about potentially harmful preventable problems occurring in UK primary care in partnership with the Greater Manchester Primary Care Patient Safety Translational Research Centre Research User Group, (GMPSTRC RUG), a public and patient involvement (PPI) group.^24^ Specifically, we aimed to:

1. codesign (with PPI partners) and test a survey asking about problems occurring in primary care that caused, or had the potential to cause, preventable harm as perceived by patients;
2. pilot the survey to examine the usefulness and overall quality of the information collected with respect to describing the patient-perceived problems, the primary care service involved, how the problem was discussed (if it was) and how it might have been prevented;
3. compare the opinions of the survey respondents, members of the public and primary care clinicians as to the likelihood the patient-reported scenario describes a potentially harmful preventable problem.

## Methods

### Designing and piloting of the survey (aim 1)

Our main aim was to design a survey asking about problems occurring in primary care that caused, or had the potential to cause, preventable harm as perceived by patients that was easily understood and free from jargon. Currently, there is no well-established terminology for asking such a question.^8^ The process began with a discussion between three members of the GMPSTRC RUG (AD, JB, CG) and one academic researcher (SJS). Questions used in previous surveys addressing a similar question^4 17–19^ were shared among the project team and used to generate several candidate questions. These questions were then discussed privately among the project team’s friends and family and within the project team (SJS, AD, JB, CG). The discussion was facilitated by making the candidate questions available online. After two iterations of this process. the survey (see box and online supplementary appendix 1 box A) was piloted online through newsletters or group mailings of several PPI and public engagement networks during November and December 2015. These networks were the associate GMPSTRC RUG, the Public Programmes team at Central Manchester Foundation Trust, the Citizen Scientist project, the Primary Care Research in Manchester Engagement Resource, North West People in Research Forum and Help Beat Diabetes volunteers (details of these groups and networks are provided in online supplementary appendix 1 box B).

10.1136/bmjopen-2017-017786.supp1 Supplementary file 1

The first question (Q1, box) was taken from the English GP patient survey in order to compare the overall level of confidence and trust in their GP among the survey respondents with that across England.^25^ The second question (Q2 in box) is the main screening question; those responding negatively to Q2 (ie, not experienced a preventable-problem) were directed to a more specific question with a list of commonly understood patient safety events (Q10, online supplementary appendix 1 box A). If this prompted recognition of experiencing a potentially harmful preventable problem, they were returned to Q4 (box). The rationale behind this approach was that the screening question (Q2, box) should be non-leading and encourage the respondents to describe their preventable problems through the subsequent questions without the suggestion that inevitably occurs following a list of possible potentially harmful preventable problems. However, if the respondent did not believe that they had experienced a potentially harmful preventable problem, then the prompt question (Q10, box) would ensure that this was the case and also test the sensitivity of Q2 (box). The option to answer on behalf of a friend or relative was offered to those who have not had a personal experience to report. This was to ensure sufficient responses to adequately test the questionnaire and also to discourage respondents from answering with another person’s experience as their own. Respondents were also asked whether they worked or volunteered in the healthcare profession and to comment on the ease of completion of the questionnaire.

### Coding of reported events (aims 2 and 3)

#### Type of problem (aim 2)

The nature of the problem in each described scenario was coded at face value, that is, as the patient described without further interpretation, by one author (SJS) and checked by a second author (JA for dental scenarios, PB for all other scenarios). A bottom-up (inductive) approach was used to identify similar topics which were coded then cross-matched to an existing taxonomy for errors in general practice^26 27^ (online supplementary appendix 1 table A). All the new codes matched the existing taxonomy within the higher two levels and the medication-related scenarios were coded to a finer level (online supplementary appendix 1 table B).

#### Likelihood the scenario described a potentially harmful preventable problem (aim 3)

Five GPs, one general dental practitioner and seven members of the public estimated the likelihood that, in their opinion, each patient-described scenario was a potentially harmful preventable problem. Brief biographies of the coders are provided in online supplementary appendix 1 table C. Some examples of the information provided to the coders are shown in boxes 1–23 in online supplementary appendix file 2 and consisted of the responses to Q5 to Q9 (box). They were not given any demographic information or the patient’s estimate of the impact on their health (Q4, box). Coders were asked to score each scenario from very likely (5) to definitely not (1) in response to the question ‘How likely do you think it is the patient was correct in thinking that their health might be worsened, or actually was made worse, because of a mistake or a problem in primary care that could have been prevented?’ Coders could also respond ‘insufficient information’, ‘Don’t know’ and give free text feedback (online supplementary appendix 1 table D). The clinician scores were used to categorise the scenarios into groups with higher or lower estimated likelihoods that they were a potentially harmful preventable problem as below.

- Higher threshold: Median score of 5 (‘very likely or certain’) or 4 (‘probably’) or at least one score of 5 (‘very likely or certain’).
- Lower threshold: Median score of 3 (‘possibly’) or at least one score of 4 (‘probably’ or higher).
- All other scenarios: Median score below 3 (‘possibly’) and zero scores above 3 (‘possibly’).

10.1136/bmjopen-2017-017786.supp2 Supplementary file 2

### Statistical analysis

Simple cross-tabulations were used to describe the data and a binary logistic regression model was used to explore whether particular types of patient were more likely to perceive potentially harmful preventable problems, for example, by demographics or their opinions. Comparisons between demographics and outcomes for the respondents and the UK (or England) population were made using a χ^2^ test. All analyses were done using Stata V.14.

### Public and patient involvement

PPI was central to this codesign study and was provided through the GMPSTRC RUG^24^ and other PPI networks (online supplementary appendix 1 box B). The study was conceived, designed, implemented and analysed by a team of three members of the public (AD, CG, JB) and one researcher (SJS). At the outset, the researcher presented the existing literature on this topic to the PPI members of the research team who then codesigned the first draft of the survey which was tested through the PPI members’ personal contacts. The piloting of the survey was through existing PPI networks as listed in online supplementary appendix 1 box B. The scoring of the questions as to the likelihood they described a potentially harmful preventable problem was undertaken by seven members of the public, two of whom had no previous experience in PPI (as well as five GPs and one general dental practitioner as described in online supplementary appendix 1 table C). These findings will be disseminated to all the PPI groups that contributed to the pilot study and the authors will forward these results to their personal contacts who contributed to the questionnaire design.

## Results

### The survey design (aim 1)

The involvement of the PPI partners in the survey design had a profound impact on the piloted version of the survey. Professional researchers may have focused more on asking questions in a way that forces the responses into categories but the PPI partners were more concerned that respondents should have the freedom to express themselves and the categorisation should occur during the analysis. They themselves had often completed surveys where there was no appropriate option in the categorical responses. We did not find any of the previous approaches^4 17–19^ suitable for this survey and chose to design a new question. The best option was felt to be an open question with a prompt question for individuals who did not recognise the concept of a preventable potentially harmful event. Another point of debate was whether we should ask initially about a ‘problem’ then ask if it was ‘preventable’ in a second question. The difficulty with simply asking about a ‘problem’ is that most patients visit their GP because they have a health problem; therefore, we thought it was more practical to focus immediately on the concept of a preventable problem encapsulated in a single phrase with a back-up question to ensure it was indeed preventable.

### Ease of use of the survey (aim 1)

Over 250 respondents provided free text feedback on the survey, 200 comments reported that the questionnaire was easy to complete and understand and just one comment described the survey as complex. Most of the remaining comments expressed the desire to be able to provide more information, for example, more than one event or report for a relative or as a carer (reporting on behalf of another person was excluded for events occurring more than 12 months ago) and 13 comments actually provided this unrequested information. Nobody used the ‘Do not understand the question’ option as their response to Q2 of box. A few respondents found it difficult to find a suitable option to describe their pattern of use of primary care or their role as a worker or volunteer in healthcare. Demographic information was not provided by 83 (13%) respondents, possibly due to lack of clarity about the end of the survey since they completed all other questions.

### Summary statistics (aim 2)

In total, 977 members of the public accessed the online pilot survey and 638 (65%) completed the survey during October and November 2015. The majority of respondents were recruited through the Help Beat Diabetes group (533, 84%, online supplementary appendix 1 box B). A flow chart of respondents through the survey is shown in online supplementary appendix 1 figure A; 223/638 (35%) of respondents reported ever experiencing a potentially harmful preventable problem in primary care of which 132 occurred within the past 12 months (21%). Sixty-two (10%) of these problems were not identified through the initial screening question (Q2) but required prompting through Q10 (box). A further 18 potentially harmful preventable problems involving friends or relatives where the respondent was present and occurred in the last 12 months were reported 13/418 (3%, online supplementary appendix 1 figure B).

### Characteristics of the respondents (aim 2)

The majority of respondents (592, 93%) had confidence and trust in the GP seen at their last appointment similar to the 2016 England proportion of 92% (Q1, box and table 1). Respondents were older than the UK generally, more likely to work or volunteer in the healthcare sector and tended to use primary care more frequently (table 1). Older respondents and those working or volunteering in the healthcare sector were no more likely to report a potentially harmful preventable problem occurring within the last 12 months but those using primary care more frequently were more likely to report a problem (table 2). There was a high response from healthcare professionals or volunteers (30% of respondents compared with approximately 3% of the UK adult population, table 1), but they were not more likely to report a preventable problem than non-healthcare workers/volunteers (35%, Pχ^2^=0.28).

Box **Brief summary of questionnaire (see online supplementary appendix 1 box A for full version of survey)**

1. Did you have confidence and trust in the GP you saw or spoke to at your last appointment? (benchmarking question)
2. When using primary care, have you ever felt concerned that your health might be worsened, or actually was made worse, because of a mistake or a problem that could have been prevented?If no to Q10, if yes to Q3
3. How long ago did the mistake or preventable problem happen?
4. How did this affect your health?
5. Which primary care service were you using when the mistake or preventable problem occurred?
6. Briefly describe the mistake or problem and how it happened.
7. Could the mistake or problem have been avoided? If so, how?
8. Were you able to talk about the mistake or problem with anybody working in the primary care service? If not, why not?
9. If you discussed the mistake or problem with somebody working in primary care, please describe their job or role.
10. In the list below are some examples of preventable problems* that might happen when using primary care. Has anything similar happened to you in the last 12 months? If yes, go to Q4.

*See Q10 in online supplementary appendix 1 box A for the list of preventable problems.

**Table 1:** Characteristics of survey respondents

Variable	All respondents n=638	Ever had problem n=223	Had problem in last 12 months n=132	UK population comparator
GP satisfaction	Missing=0	Missing=0	Missing=0	English GP patient survey^25^
Yes, definitely	384 (60%)	81 (36%)	55 (42%)	64%
Yes, to some extent	208 (33%)	110 (49%)	52 (39%)	28%
No, not at all	39 (6%)	27 (12%)	21 (16%)	4%
Do not know/cannot say	7 (1%)	5 (2%)	4 (3%)	3%
Worked or volunteered in healthcare	Missing=92	Missing=40	Missing=19	NHS workforce*
Yes	166 (30%)	64 (35%)	41 (36%)	3%
Gender	Missing=87	Missing=38	Missing=16	ONS mid-2015 estimates†
Female	268 (49%)	106 (57%)	63 (54%)	51%
Age	Missing=85	Missing=37	Missing=15	ONS mid-2015 estimates†
16–34 years	42 (8%)	22 (12%)	11 (9%)	31%
35–54 years	143 (26%)	54 (29%)	34 (29%)	34%
55–64 years	162 (29%)	59 (32%)	31 (27%)	14%
65–74 years	170 (31%)	44 (24%)	32 (27%)	12%
Over 75 years	36 (7%)	7 (4%)	9 (8%)	9%
Last primary care contact	Missing=88	Missing=39	Missing=14	English GP patient survey^25^
Within last week	169 (31%)	65 (35%)	48 (41%)	84% within last 12 months
Within last month	248 (45%)	79 (43%)	47 (40%)	
Within the last 12 months	121 (22%)	34 (18%)	20 (17%)	
Over 12 months ago	12 (2%)	6 (3%)	3 (3%)	15%
Usual primary care usage	Missing=88	Missing=40	Missing=17	
At least once a month	181 (33%)	73 (40%)	52 (45%)	-
At least once per 6 months	285 (52%)	79 (43%)	45 (39%)	-
Once per 12 months or less	84 (15%)	31 (17%)	18 (16%)	-

*http://content.digital.nhs.uk/searchcatalogue?productid=24139&topics=1_2fWorkforce_2fSt aff+numbers&sort=Relevance&size=10&page=1#top

†https://www.ons.gov.uk/peoplepopulationandcommunity/populationandmigration/populationestimates/bulletins/annualmidyearpopulationestimates/latest

GP, general practitioner; NHS, National Health Service; ONS, Office for National Statistics.

### The nature of the potentially harmful preventable problems (aim 2)

**Figure 1:**
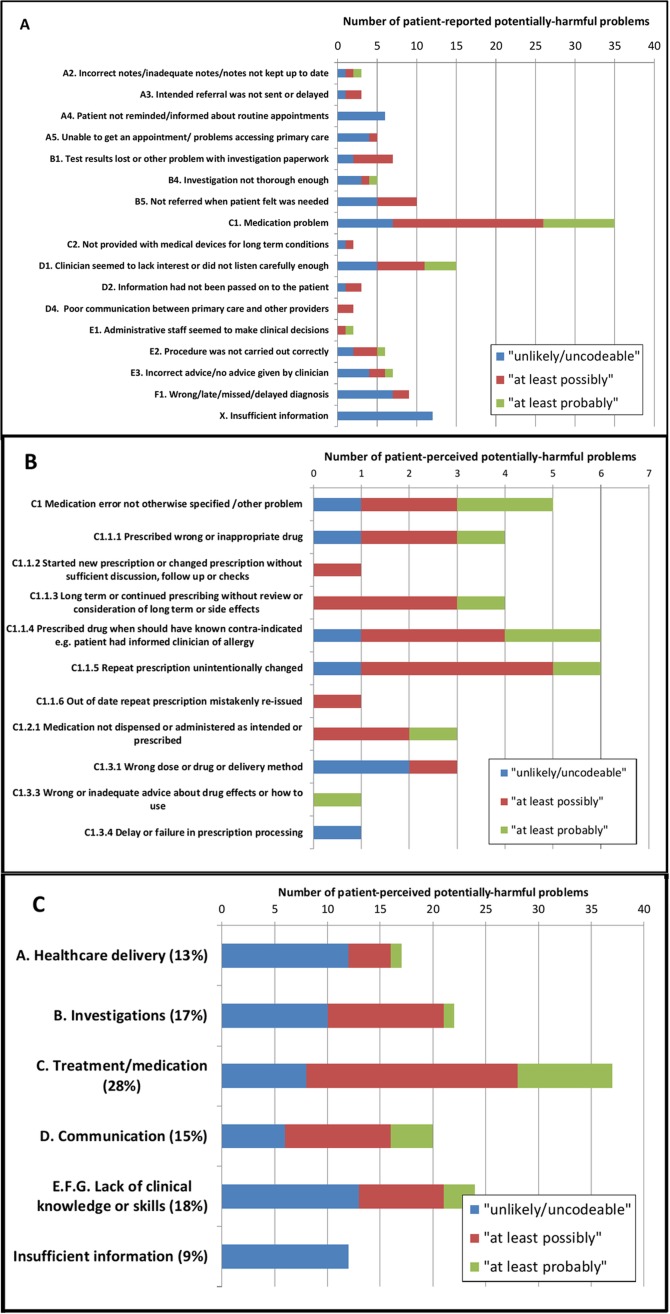
Numbers of patient-perceived problems occurring in the last 12 months categorised by type of problem as described in Tables A & B, online Appendix 1 (A coded to 2 levels, B medication problems coded to 3 levels, C coded to 1 level). Colour coding describes clinican ranking as to the likelihood it is "probably" or "possibly" a potentially-harmful preventable problem as defined in Table 5.

The types of patient-reported scenarios and their categorisation following clinician review are shown in figure 1. Medication-related problems were most frequently reported type of problem and also more likely to be ranked as a potentially harmful problem by clinicians, as were communication problems. The type of scenario categorised according to whether it arose from the open-ended screening question (Q2) or prompted through the list of potential problems (Q10) is shown in online supplementary appendix 1 figures C,D. Scenarios describing problems with appointments, accessing healthcare or loss of test results were more likely to arrive via the prompt question suggesting that patients did not see these as a potentially harmful problem in the first instance. The majority of potentially harmful preventable problems in the past 12 months occurred in general practice (73%, table 3) and pharmacy (5%, table 3).

**Table 2:** Prevalence of respondents reporting a potentially harmful preventable problem within the last 12 months and unadjusted and adjusted ORs estimated by logistic regression

Respondent characteristics n=638	Frequency— all reported n=132	Unadjusted OR— all reports	Adjusted* OR— all reports	Adjusted* OR— after GP review (lower threshold, table 5)
Gender (87 missing)				
Male	53/283 (19%)	1 (ref)	1 (ref)	1 (ref)
Female	63/268 (24%)	1.3 (0.9 to 2.0)	1.4 (0.9 to 2.2)	1.3 (0.7 to 2.3)
Age (85 missing)				
16–34 years	11/42 (26%)	1 (ref)	1 (ref)	1 (ref)
35–54 years	34/143 (24%)	0.9 (0.4 to 1.9)	0.8 (0.3 to 1.8)	0.8 (0.3 to 2.1)
55–64 years	31/162 (19%)	0.7 (0.3 to 1.5)	0.7 (0.3 to 1.5)	0.6 (0.2 to 1.7)
65–74 years	32/170 (19%)	0.7 (0.3 to 1.4)	0.6 (0.3 to 1.4)	0.4 (0.2 to 1.2)
Over 75 years	9/36 (25%)	0.9 (0.3 to 2.6)	1.1 (0.4 to 3.2)	0.9 (0.2 to 3.2)
Last primary care contact (88 missing)				
Within last week	48/169 (28%)	1 (ref)	1 (ref)	1 (ref)
Within last month	47/248 (19%)	0.6 (0.4 to 0.9)	0.7 (0.4 to 1.1)	0.6 (0.3 to 1.0)
Within the last 12 months	20/121 (17%)	0.5 (0.3 to 0.9)	0.6 (0.3 to 1.2)	0.5 (0.2 to 1.3)
Over 12 months ago	3/12 (25%)	0.8 (0.2 to 4.0)	0.9 (0.2 to 4.2)	0.4 (0.0 to 3.9)
Usual primary care usage (88 missing)				
At least once a month	52/181 (29%)	1 (ref)	1 (ref)	1 (ref)
At least once per 6 months	45/285 (16%)	0.5 (0.3 to 0.7)	0.6 (0.3 to 0.9)	0.5 (0.3 to 0.9)
Once per 12 months or less	18/84 (21%)	0.7 (0.4 to 1.2)	0.8 (0.4 to 1.6)	0.7 (0.3 to 1.8)
Works or volunteers in healthcare (92 missing)				
No	72/380 (19%)	1 (ref)	1 (ref)	1 (ref)
Yes	41/166 (25%)	1.4 (0.9 to 2.2)	1.3 (0.8 to 2.1)	1.5 (0.9 to 2.7)

*Adjusted for gender, age, last primary care contact, usual primary care usage, works or volunteers in healthcare.

GP, general practitioner.

### The patient’s response to the potentially harmful preventable problem (aim 2)

Around half the respondents had not discussed their problem with anybody working in primary care (51%, table 3). The most common reasons for not discussing the problem were being unable to find a primary care professional with whom to discuss the problem (31%, table 3) or they did not feel comfortable discussing their concerns (24%, table 3). The respondent’s suggestions for ways to prevent the problem from happening are summarised in table 4. The most frequent suggestions were that clinicians should involve the patient more fully in the healthcare process (ie, listen to the patient and trust their judgement more) and be up to date with, and apply, the most recent information about the patient’s condition (ie, take into account all of the patient’s information—their medical history and results and letters).

**Table 3:** The patient’s response to their perceived potentially harmful preventable problem and the primary care service involved for problems occurring in the last 12 months

Primary care service	All reported problems	Clinician ranked ‘possibly or higher’ (lower threshold)
All services	132	71
GP surgery	97 (73%)	61 (86%)
Out of hours care/A&E/ambulance	4 (3%)	1 (1%)
Walk-in clinic	2 (2%)	0
Dental surgery	4 (3%)	1 (1%)
Pharmacy	7 (5%)	6 (8%)
Community or district nursing	4 (3%)	0
Opticians	2 (2%)	1 (1%)
Mental health services	1 (1%)	0
Missing	11 (8%)	1 (1%)
Did you discuss the problem with primary care staff?		
All respondents	132	71
Yes—discussed with primary care staff	56 (42%)	42 (59%)
No—did not discuss with primary care staff	67 (51%)	29 (41%)
Missing	9 (7%)	0
Reason not discussed with primary care staff		
All not discussing problem	67	29
Did not feel comfortable to discuss the problem	16 (24%)	8 (28%)
Could not find anybody with whom to discuss the problem	21 (31%)	10 (34%)
Unconcerned about the problem	7 (10%)	5 (17%)
Did not notice the problem at the time (or too ill)	11 (16%)	4 (14%)
Other	5 (7%)	2 (7%)
Missing	7 (10%)	0
Profession of discussant		
All discussing problem	56	42
GP	28 (50%)	19 (45%)
Practice manager	5 (9%)	5 (21%)
Receptionist	2 (4%)	1 (2%)
Practice nurse	6 (11%)	5 (12%)
Pharmacist or dispenser	7 (13%)	7 (17%)
General dental practitioner	2 (4%)	1 (2%)
Dietician	1 (2%)	1 (2%)
Missing	5 (9%)	3 (7%)
Role of discussant in patient’s care		
Member of staff directly involved	23 (41%)	16 (38%)
Another member of staff at same institution	25 (45%)	20 (48%)
Above unclear	8 (14%)	6 (14%)

A&E, accident and emergency; GP, general practitioner.

### Likelihood the patient-reported scenario described a potentially harmful preventable problem (aim 3)

**Figure 2:**
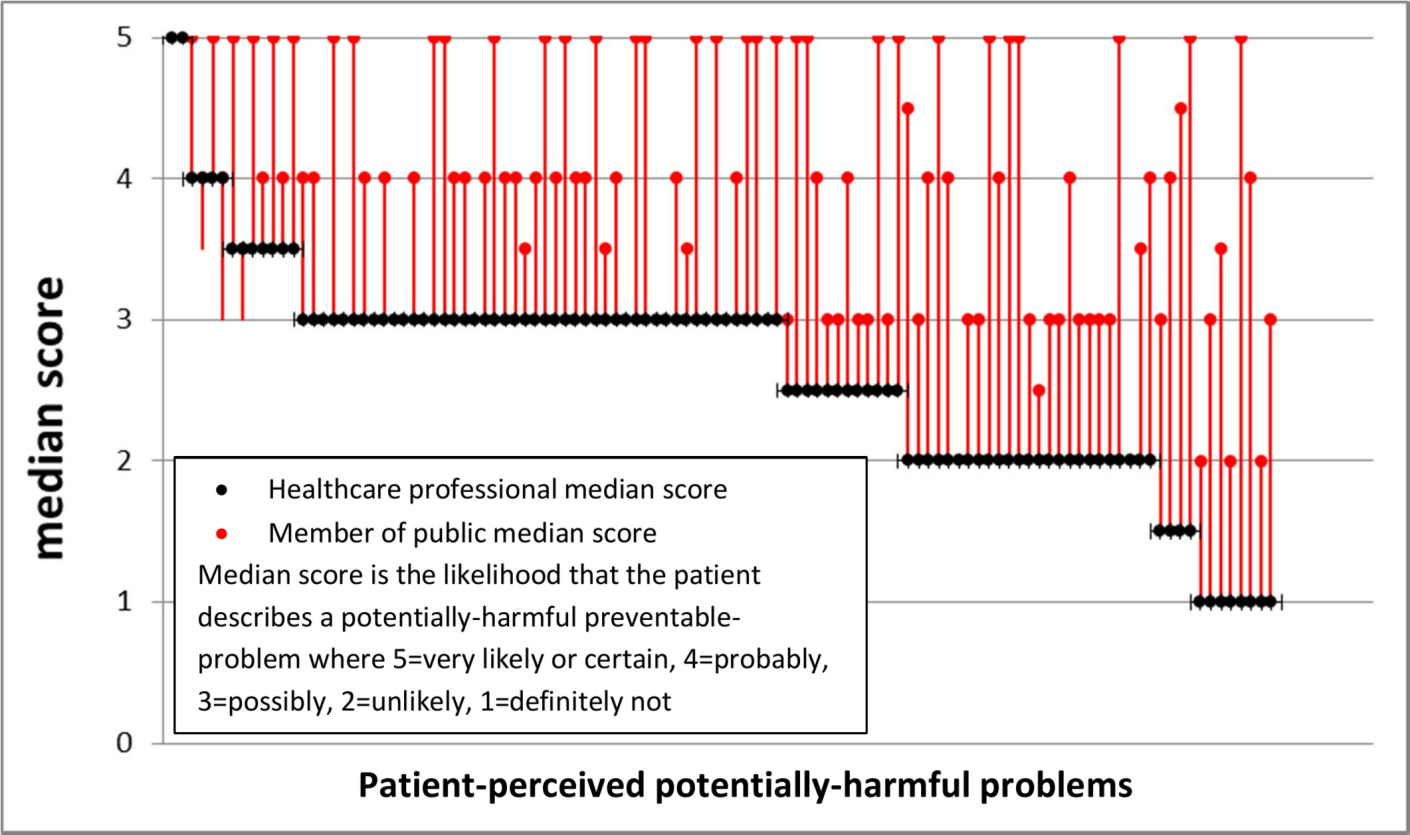
Median estimates as to the likelihood that the patient describes a potentially harmful preventable problem occurring in the last 12 months by six clinicians and seven members of the public.

Generally, the members of the public assigned a higher probability to the likelihood that the patient-described scenario was a potentially harmful preventable problem compared with clinicians (figure 2, table 5). In 89/108 (82%) scenarios, the median score for the PPI researchers was higher than for the clinicians, and for 38 (35%) scenarios, the PPI median score was two or more points higher in a five-point scale. Following clinician review, 3% of the respondents were judged to have ‘probably’ experienced a potentially unsafe preventable problem during the past 12 months and 11% as ‘possibly’ (using higher and lower thresholds described in table 5). Scenarios described by healthcare professionals or volunteers were significantly more likely to be categorised as a potentially harmful preventable problem following clinician review using both the lower (9% vs 16%, Pχ^2^=0.01) and higher threshold (2% vs 6%, Pχ^2^=0.004). Examples of the patient-reported scenarios with higher clinician rankings are shown in boxes 1–15, online supplementary appendix 2 and those with greatest disagreement between members of the public and clinicians in boxes 16–23, online supplementary appendix 2.

**Table 4:** Patient suggestions as to how the potentially harmful preventable problem might have been prevented

How could it be prevented?	All reported problems n=132	Clinician ranked ‘possibly or higher’ (lower threshold) n=71
1. More resources—all	14 (11%)	3 (4%)
1.1 Quicker access to primary care	7 (5%)	2 (3%)
1.2 More thorough and quicker investigations	2 (2%)	1 (1%)
1.3 Fewer demands on primary care—more staff or fewer patients	1 (1%)	0
1.4 More time with clinicians for treatment and diagnosis	2 (2%)	0
1.9 Provision of resources to manage long-term conditions	1 (1%)	0
1.10 Provision of patient travel service for routine appointments	1 (1%)	0
2. Improved communication and involvement of patients	26 (20%)	18 (25%)
2.1 Listen to the patient and trust their judgement more	21 (16%)	15 (21%)
2.2 Tell patients about their diagnosis, test results, changes in medication or loss of results	3 (2%)	1 (1%)
2.3 Improve communication between staff (within or outside primary care)	2 (2%)	2 (3%)
3. Better organisation and administration	17 (13%)	10 (14%)
3.1 Follow-up referrals and appointments to ensure they happen, be consistent in sending routine reminders	10 (8%)	3 (4%)
3.2 Log in or process results as soon as received to avoid loss	1 (1%)	1 (1%)
3.3 Keep the notes up to date, well-organised, safe and ensure information is transcribed accurately	5 (4%)	5 (7%)
3.4 Keep a record of the location of equipment	1 (1%)	1 (1%)
4. Improved prescribing systems	18 (14%)	17 (24%)
4.1 More checks on prescribing and dispensing	8 (6%)	8 (11%)
4.2 Check repeat prescriptions carefully, especially for transcribing errors	8 (6%)	7 (10%)
4.3 Use medication reviews and computerised clinical decision support systems	2 (2%)	2 (3%)
5. Better clinical practice	19 (14%)	10 (14%)
5.1 Take in to account all the patient’s information—their medical history and results and letters	13 (10%)	7 (10%)
5.2 Address the patient’s problem in some way—patients can feel their problem is being ignored	5 (4%)	2 (3%)
5.3 Act on advice from other clinicians and test results	1 (1%)	1 (1%)
6. Staff training	11 (8%)	7 (10%)
6.1 More informed and better trained staff	11 (8%)	7 (10%)
Other responses	27 (20%)	6 (8%)
Do not know/missing	21 (16%)	3 (4%)
Problem was due to an individual member of staff	2 (2%)	1 (1%)
Prescribe right, better, different, more, less medicine	1 (1%)	0
Better organisation	1 (1%)	0
Laboratory procedures were the problem	2 (2%)	2 (3%)

**Table 5:** Categorisation of patient perceived potentially harmful preventable problems occurring in the last 12 months following review by clinicians and members of the public

Group label	Threshold criteria	Clinician scores n=132	Members of the public scores n=132
1. Higher threshold	Median score of ‘very likely or certain’ or ‘probably’ or at least one score of ‘very likely or certain’	18 (14%)	87 (66%)
2. Lower threshold	Median score of ‘possibly’ or at least one score of ‘probably’ or higher	71 (54%)	104 (79%)
3. Any possibility	At least one score of ‘unlikely’ or higher	106 (80%)	109 (83%)
4. No problem	All scores ‘definitely not’ or not coded	1 (1%)	0
5. Not coded	Insufficient information for coding by all coders	25 (19%)	23 (17%)

## Discussion

We have designed and tested a survey to measure the frequency of occurrence of potentially harmful preventable problems in primary care and found it to be well understood and acceptable to patients. The open-ended questions (Q6–Q9, box) led to patient-described scenarios that mapped well to an existing taxonomy designed and used by clinicians and researchers (see online supplementary appendix 1 26 27). This implies agreement between clinicians, researchers and patients in identifying the characteristics of a potentially harmful problem. Furthermore, the use of an open-ended screening question (Q2, box) to ensure that any problems unique to the patient perspective were identified did not find additional new types of problem. However, the open-ended question elicited more problems related to communication and medication suggesting that the public are more likely to view these as safety problems than problems related to appointments and referrals or investigations (see online supplementary appendix 1 figure C,D) in agreement with clinicians who were more likely to rank these types of scenarios as potentially harmful. The observation that members of the public were generally more likely to rank the scenarios as a potentially harmful preventable problem than clinicians (figure 2) is important;primary care should not only be safe but also be perceived as safe by patients.

### Strengths and weaknesses of the study

We believe that our survey captures the true patient perspective due to the involvement of members of the public as research partners through data acquisition to analysis and reporting in a codesigned study. By the use of a simple non-leading screening question, we encouraged respondents to express their own perspective on what constituted a potentially harmful preventable problem rather than directing them towards existing definitions. To ensure that we did not miss any problems, we followed up with a prompt that encouraged respondents to think in terms of the traditional view of patient safety problems. Furthermore, our survey goes further than describing and counting the frequency of occurrence of potentially harmful preventable problems and provides information about how patients dealt with the problem and how it could have been prevented that offers insight into ways to reduce the frequency of their occurrence. The absence of a link between practices and the patients allows for responses that might not occur if this survey were administered through the individual’s practice. The main weakness of the study is the self-selection of the respondents who were older and tended to use primary care more frequently. More frequent users of primary care were more likely to report a problem, but age was not associated with the likelihood of reporting a problem. Our benchmarking question (Q1, box) showed that the respondents were similar to the English GP patient survey^25^ in terms of their level of confidence and trust in their GP and not a group with a more negative attitude towards primary care as might have happened given the nature of the survey. We also acknowledge that, by design, this study is totally from the patient perspective. We aim to provide insight into the patient’s perspective and not to imply that one or the other point of view is more important but rather there are differences in perceptions that need to be understood and reconciled.

### Strengths and weaknesses in relation to other studies

Our finding that 35% of respondents perceived that they had experienced a potentially harmful problem in their lifetime is consistent with a European survey (43% of UK respondents felt that it was ‘likely’ that patients could be harmed by non-hospital healthcare).^17^ This study offers some insight into the type of concerns that might underlie this apparent lack of confidence in primary care. A face-to-face interview in family practice waiting rooms in the USA reported that 16% of respondents believed a physician had made a mistake in their care.^28^ The types of problem and patient responses to the problem are similar to those that have been described qualitatively,^1 22^ but we have taken this a step further by quantifying their frequency of occurrence and other descriptors of the problem from the patient’s perspective. In this small study, we did not find that patients were particularly likely to attribute blame to individual members of staff as has been observed previously,^3 4^ perhaps partly due to the high proportion of respondents working or volunteering in healthcare.

### Unanswered questions and future research

Our finding that 21% of respondents perceived that they had experienced a potentially harmful problem in the last 12 months, and the corresponding proportion following clinician review of 3% (higher threshold) to 11% (lower threshold) may well reflect the self-selected nature of the study population and needs to be validated in a large population level survey. We anticipate that a population-level survey would be fruitful since this approach yielded a number of patient-described scenarios that were amenable to further analysis including coding by clinicians. The high response to this pilot survey by healthcare professionals and volunteers probably reflects the population invited to complete the survey as well as an interest in this topic. It is likely that these respondents are better at articulating their potentially harmful problem given the higher ranking given by clinicians to scenarios originating from healthcare professionals. Healthcare professionals are an educated and accessible group with the expectations of a patient and with an understanding of the healthcare system who could provide a valuable resource for learning about preventable problems in primary care. Further work is also needed to understand and reconcile the differences between members of the public and clinicians’ perceptions of a potentially harmful problem. In 1997, Professor Berwick stated ‘The ultimate measure by which to judge the quality of a medical effort is whether it helps patients (and their families) as they see it. Anything done in healthcare that does not help a patient or family is, by definition, waste, whether or not the professions and their associations traditionally hallow it.’ If this tenet still holds, then we suggest there is a real need to influence patient’s expectations and beliefs about primary care.

## Supplementary Material

Reviewer comments

Author's manuscript

## References

[1] Rhodes P, Campbell S, Sanders C Trust, temporality and systems: how do patients understand patient safety in primary care? A qualitative study. Health Expect 2016;19:253–63. 10.1111/hex.12342 25644998 PMC5024004

[2] Vincent C, Amalberti R Safer Healthcare: Strategies for the Real World. Springer, 2016. 29465922

[3] Blendon RJ, DesRoches CM, Brodie M, et al Views of practicing physicians and the public on medical errors. N Engl J Med 2002;347:1933–40. 10.1056/NEJMsa022151 12477944

[4] Hotvedt R, Førde OH Doctors are to blame for perceived medical adverse events. a cross sectional population study. The Tromsø Study. BMC Health Serv Res 2013;13:46 10.1186/1472-6963-13-46 23384420 PMC3623705

[5] Kuzel AJ, Woolf SH, Gilchrist VJ, et al Patient reports of preventable problems and harms in primary health care. Ann Fam Med 2004;2:333–40. 10.1370/afm.220 15335132 PMC1466690

[6] The Health Foundation. Evidence scan: Involving patients in improving safety, 2013 http://www.health.org.uk/sites/health/files/InvolvingPatientsInImprovingSafety.pdf (accessed 04 Apr 2017).

[7] Basch E The missing voice of patients in drug-safety reporting. N Engl J Med 2010;362:865–9. 10.1056/NEJMp0911494 20220181 PMC3031980

[8] King A, Daniels J, Lim J, et al Time to listen: a review of methods to solicit patient reports of adverse events. Qual Saf Health Care 2010;19:148–57. 10.1136/qshc.2008.030114 20351164

[9] Longtin Y, Sax H, Leape LL, et al Patient participation: current knowledge and applicability to patient safety. Mayo Clin Proc 2010;85:53–62. 10.4065/mcp.2009.0248 20042562 PMC2800278

[10] Sandars J, Esmail A The frequency and nature of medical error in primary care: understanding the diversity across studies. Fam Pract 2003;20:231–6. 10.1093/fampra/cmg301 12738689

[11] Panesar SS, deSilva D, Carson-Stevens A, et al How safe is primary care? A systematic review. BMJ Qual Saf 2016;25:544–53. 10.1136/bmjqs-2015-004178 26715764

[12] Michel P, Brami J, Chanelière M, et al Patient safety incidents are common in primary care: A national prospective active incident reporting survey. PLoS One 2017;12:e0165455 10.1371/journal.pone.0165455 28196076 PMC5308773

[13] NHS Improvement. National quarterly data on patient safety incident reports. 2016 https://improvement.nhs.uk/resources/national-quarterly-data-patient-safety-incident-reports-september-2016/ (accessed 04 Apr 2017).

[14] Hutchinson A, Young TA, Cooper KL, et al Trends in healthcare incident reporting and relationship to safety and quality data in acute hospitals: results from the National Reporting and Learning System. Qual Saf Health Care 2009;18:5–10. 10.1136/qshc.2007.022400 19204125

[15] NHS England. General practice patient safety reporting form launched. 26th. 2015 https://www.england.nhs.uk/2015/02/gp-patient-safety-reporting/ (accessed 04 Apr 2017).

[16] The Health Foundation. Evidence Scan: Improving safety in primary care. 2011 http://www.health.org.uk/sites/health/files/ImprovingSafetyInPrimaryCare.pdf (accessed 04 Apr 2017).

[17] European Commission. Special Eurobarometer 411 Patient Safety and Quality of Care. 2014 https://ec.europa.eu/health/sites/health/files/patient_safety/docs/ebs_411_sum_en.pdf (accessed 04 Apr 2017).

[18] Mira JJ, Nebot C, Lorenzo S, et al Patient report on information given, consultation time and safety in primary care. Qual Saf Health Care 2010;19:e33 10.1136/qshc.2009.037978 20511244

[19] Wasson JH, MacKenzie TA, Hall M Patients use an internet technology to report when things go wrong. Qual Saf Health Care 2007;16:213–5. 10.1136/qshc.2006.019810 17545349 PMC2465004

[20] Ricci-Cabello I, Gonçalves DC, Rojas-García A, et al Measuring experiences and outcomes of patient safety in primary care: a systematic review of available instruments. Fam Pract 2015;32:106–19. 10.1093/fampra/cmu052 25192905

[21] Ricci-Cabello I, Avery AJ, Reeves D, et al Measuring Patient Safety in Primary Care: the Development and Validation of the "patient reported experiences and outcomes of safety in primary care" (PREOS-PC). Ann Fam Med 2016;14:253–61. 10.1370/afm.1935 27184996 PMC4868564

[22] Ricci-Cabello I, Saletti-Cuesta L, Slight SP, et al Identifying patient-centred recommendations for improving patient safety in general poractices in England: a qualitative content analysis of free-text responses using the patient reported experiences and outcomes of safety in primary care (PREOS-PC) questionnaire. Health Expect 2017;20:961–72. 10.1111/hex.12537 28244631 PMC5600214

[23] Southwick FS, Cranley NM, Hallisy JA A patient-initiated voluntary online survey of adverse medical events: the perspective of 696 injured patients and families. BMJ Qual Saf 2015;24:620–9. 10.1136/bmjqs-2015-003980 PMC4602250 26092166

[24] Stocks SJ, Giles SJ, Cheraghi-Sohi S, et al Application of a tool for the evaluation of public and patient involvement in research. BMJ Open 2015;5:e006390 10.1136/bmjopen-2014-006390 PMC4360721 25770228

[25] NHS England. GP Patient Survey – National summary report. 2016 http://gp-survey-production.s3.amazonaws.com/archive/2016/January/January+2016+National+Summary+Report.pdf (accessed 04 Apr 2017).

[26] Dovey SM, Meyers DS, Phillips RL, et al A preliminary taxonomy of medical errors in family practice. Qual Saf Health Care 2002;11:233–8. 10.1136/qhc.11.3.233 12486987 PMC1743626

[27] Makeham MA, Dovey SM, County M, et al An international taxonomy for errors in general practice: a pilot study. Med J Aust 2002;177:68–72. 12098341 10.5694/j.1326-5377.2002.tb04668.x

[28] Kistler CE, Walter LC, Mitchell CM, et al Patient perceptions of mistakes in ambulatory care. Arch Intern Med 2010;170:1480–7. 10.1001/archinternmed.2010.288 20837835 PMC3070906

