# Supplementary material for: Development and piloting of a survey to estimate the frequency and nature of potentially harmful preventable problems in primary care from a UK patient’s perspective
Source: BMJ Open. 2018 Feb 3;8(2):e017786. doi: 10.1136/bmjopen-2017-017786 (PMC5829776; doi:10.1136/bmjopen-2017-017786)
Supplement: Supplementary file 1 [file bmjopen-2017-017786supp001.pdf]

Appendix 1. Details of survey, coding systems, public and clinician contributors and supplementary results

Box A. Pilot survey administered online November and December 2015

Q1. Did you have confidence and trust in the GP you saw or spoke to at your last appointment?

Response options: Yes, definitely, Yes, to some extent, No, not at all, Don't know / can't say

Q2. When using primary care have you ever felt concerned that your health might be worsened, or actually was made worse, because of a mistake or a problem that could have been prevented?

Response options: Yes, No- *go to Q10,* Do not understand the question- *go to Q10*, Don't know / can't remember- *go to Q10*

Q3. How long ago did the mistake or preventable problem happen?

Response options: Within the last 12 months, More than 12 months ago- *go to Q10,* Can't remember- *go to Q10*

Q4. In your opinion did this experience

Response options: Make your health worse, Not certain but it might have made your health worse, Could have made your health worse if you had not noticed the problem, Delayed your treatment but had no effect on your health, Not affect you, or your health, Other, please explain

Q5. Which primary care service were you using when the mistake or preventable problem occurred?

Response options: GP surgery, Out of hours care, Walk in clinic, Dental, Pharmacy, Community or district nursing, Ambulance, Opticians, Other- please specify

Q6. Briefly describe the mistake or problem and how it happened

Response options: free text

Q7. Could the mistake or problem have been avoided? If so how?

Response options: free text

Q8. Were you able to talk about the mistake or problem with anybody working in the primary care service?

Response options: Yes, Yes had the opportunity but did not feel comfortable to discuss the mistake or problem, No I could not find anybody with whom I could discuss the mistake or problem, No I was not concerned about the problem, No I did not notice the mistake or problem at the time, I was too distressed to discuss the mistake or problem, Other or don't know - please describe

Q9. If you discussed the mistake or problem with somebody working in primary care please describe their job or role

Response options: free text

Q10. In the list below are some examples of preventable problems that might happen when using primary care. Has anything similar happened to you in the last 12 months? Please check as many as applicable or "NONE OF BELOW"

NONE OF BELOW
Wrong or late diagnosis
Not referred for further investigation when needed
Test results being lost or mixed up
Receiving the wrong medicine or wrong dose
Should not be prescribed the medicine because of another health problem
Should not be prescribed the medicine because of another medication already taking
Poor communication leading to misunderstanding of diagnosis or treatment
Not referred to a specialist when needed

Unclear instructions about treatment<br>Not offering of prevention or screening programmes eg CVD/stroke prevention clinics<br>Failure to recognise or act on vulnerable people's needs eg child abuse, suicide risk or mental health problems<br>Mistake with a procedure eg dental treatment, injection, ear syringing, physiotherapy<br>Failure to notify about recommended vaccinations eg flu, HPV<br>Poor hygiene<br>Unsafe building or premises<br>Any other preventable problem in the last 12 months (in your opinion)<br>Other, please explain below
Q11. Are you male or female? Response options: Male, Female, prefer not to say
Q12. How old are you?<br>Response options: under 16, 16 to 24, 25 to 34, 45 to 54, 55 to 64, 65 to 74, 75 to 84, 85 or older
Q13. When was your last contact with primary care?<br>Response options: Last week, Last month, Last 12 months, Over 12 months ago
Q14. What best describes your usual pattern of use of primary care? Response options: Once per week, Once per 2 weeks, Once per month, Once per 6 months, Once per 12 months or less often
Q15. Are you registered with a GP practice?<br>Response options: Yes, No, I only use walk in centres, Don't know
Q16. Do you work or volunteer in healthcare or healthcare research as a professional, patient, carer or member of the public? (if you are retired answer for your occupation before retirement)<br>Response options: Yes, No
Q17. We are still trying to improve this questionnaire so would be grateful for any feedback about how easy you found the questionnaire to complete? How can it be improved?<br>Response options: free text

Box B. List of public and patient involvement groups used to distribute the pilot survey
Associate Research User Group of the Greater Manchester Primary Care Patient Safety Translational Research Centre http://research.bmh.manchester.ac.uk/primary-care-patient-safety/GetInvolved/
The Primary Care Research in Manchester Engagement Resource http://research.bmh.manchester.ac.uk/PRIMER/about/
HelpBeatDiabetes https://www.researchforthefuture.org/diabetes/
The Nowgen Centre https://research.cmft.nhs.uk/getting-involved/involvement
The Citizen Scientist project http://www.citizenscientist.org.uk/
North West People in Research Forum https://www.northwestpeopleinresearchforum.org/

Table A. Coding of patient-reported potentially-unsafe scenarios in primary care

**1. Errors in the process of the healthcare delivery system**	
Makeham 2002, Dovey 2002	Common threads reported in this study
1.1. Errors in the process of conducting an administrative task	A1. Administrative problem not otherwise specified
1.1.1. Information filed in wrong place or wrong time	
1.1.2. Unavailability of information that should have been in patients charts<br>1.1.2.1. Entire chart or part of chart could not be accessed when needed<br>1.1.2.2. Care provided was not documented<br>1.1.2.3. Item(s) of information missing from chart	A2. Incorrect notes/inadequate notes/notes not kept up to date
1.1.3. Errors in patient's movement through the healthcare delivery system	A3. Intended referral was not sent or delayed<br>A4. Patient not reminded, informed or assisted to attend regular check-ups or other necessary routine treatments
1.1.4. Errors in the taking and distributing of messages	
1.1.5. Errors in managing appointments for healthcare	A5. Unable to get an appointment/other problems with making appointment<br>A6. Ambulance delayed or did not arrive
**1.2. Errors in the process of investigating a patient's condition**	
1.2.1. Laboratory errors<br>1.2.1.1. Wrong test ordered or test not ordered when appropriate<br>1.2.1.2. Errors in the process of obtaining or processing a laboratory specimen<br>1.2.1.3. Error in the process of physician receiving accurate laboratory results in a timely fashion<br>1.2.1.4. Inappropriate response to an abnormal laboratory result	B1. Test results lost or other problem with investigation paperwork<br>B2. Incorrect interpretation of tests or other investigation results<br>B3. Clinician did not consider patient history sufficiently/did not use patient's notes adequately<br>B4. Investigation not thorough enough<br>B5. Not referred when patient felt was needed
1.2.3. Errors in the processes of other investigations<br>1.2.3.1. Wrong test ordered or test not ordered when appropriate<br>1.2.3.2. Errors in the process of obtaining or processing of other diagnostic investigation<br>1.2.3.3. Error in the process of physician receiving accurate test results of other investigation in a timely fashion<br>1.2.3.4. Inappropriate response to an abnormal result of other investigation	
**1.3. Errors in the process of treating a patient's condition**	
1.3.1. Errors in the process of treating with medications<br>1.3.1.1. Wrong medication or wrong dose of medication ordered or medication not ordered by physician when appropriate<br>1.3.1.2. Error in the process of delivering a medication order or inappropriate medication order by a provider working under physician supervision<br>1.3.1.3. Error in the process of dispensing medication as ordered	C1. Medication problem<br><br>C2. Not provided with medical devices needed to manage long term conditions

1.3.2. Errors in other treatments	C3. Problem with dental treatment or diagnosis
**1.4. Errors in the process of communication**	
1.4.1. Errors in communication between primary healthcare provider and patients	D1. Clinician seemed to lack interest in the patient's health problem or did not listen carefully enough<br>D2. Information about the patient's health had not been passed on to the patient who felt it should have been<br>D3. Communication problem between patient and primary care staff
1.4.2. Errors in communication between healthcare providers	D4. Problem with communication between primary care and other types of care including secondary care<br>D5. Disagreement between 2 clinicians
**2. Errors arising from lack of clinical knowledge or skills**	
2.1. Errors in the execution of a clinical task<br>2.1.1. Non-clinical staff made the wrong clinical decision<br>2.1.2. Failed to follow standard practice<br>2.1.3. Lacked needed experience or expertise in a clinical task	E1. Administrative staff seemed to make clinical decisions<br>E2. Procedure was not carried out correctly<br>E3. Incorrect advice/no advice given by clinician
**2.2. Errors in diagnosis**<br>2.2.1. Wrong or delayed diagnosis	F1. Wrong/late/missed/delayed diagnosis
**2.3. Wrong treatment decision**	G1. Wrong treatment decision
	H. Other
	X. Not a problem/ insufficient information/refused/don't know

Table B. Level 4 coding of patient-reported potentially-unsafe medication scenarios

Common threads reported in this study grouped as described by Makeham 2002, Dovey 2002
C1 Medication error not otherwise specified /other problem
• **1.3.1.1. Ordering medications (prescribing)**
C1.1.1 Prescribed wrong or inappropriate drug
C1.1.2 Started new prescription or changed prescription without sufficient discussion, follow up or checks
C1.1.3 Long term or continued prescribing without review or consideration of long term or side effects
C1.1.4 Prescribed drug when should have known contra-indicated *e.g.* patient had informed clinician of allergy, adverse reaction or it was in the records
C1.1.5 Repeat prescription unintentionally changed
C1.1.6 Out of date repeat prescription mistakenly re-issued
• **1.3.1.2./1.3.1.3. Implementing or receiving medications (dispensing or issuing)**
C1.2.1 Medication not dispensed or administered as intended or prescribed
• **1.3.1.1/1.3.1.2./1.3.1.3. Ordering, implementing or receiving medications**
C1.3.1 Wrong dose or drug or delivery method
C1.3.2 Being given another patient's drugs or prescription
C1.3.3 Wrong or inadequate advice about drug effects or how to use
C1.3.4 Delay or failure in prescription processing

Table C. Demographics of clinicians and members of the public reviewing the patient-reported problems and estimating the likelihood the scenarios describes a potentially-harmful preventable-problem occurring in primary care

**Demographics of GP and dentist coders**	**frequency n=6**
**Gender**	
Female	3
Male	3
**Years working as a GP or dentist**	
Less than 15 years	1
15 to 25 years	2
Over 25 years	3
**Current position**	
Partner	4
Retired within last 12 months	2
**Demographics of the members of the public**	**frequency n=7**
**Gender**	
Female	6
Male	1
**Age**	
30 to 39 years	2
40 to 49 years	1
50 to 59 years	2
60 to 69 years	2
**Ethnicity**	
White British	5
British Indian	2
**Years of PPI experience**	
None	2
Less than 1 year	1
1 to 5 years	2
Over 5 years	2
**Further background information**	
PPI reviewer 1. Currently working freelance on education and PPI projects; previously worked in a pastoral role at a college; a lay representative for courses training healthcare scientists.	
PPI reviewer 2. Retired primary school teacher with several long term health conditions; single parent; was a young carer for a parent with a long term condition.	
PPI reviewer 3. Former higher education administrator; current university tutor; patient partner on varied research projects; carer for family members aged 0-100 with physical and/or mental health long term conditions.	
PPI reviewer 4. Currently working as a civil servant and has several long term health conditions.	
PPI reviewer 5. Full-time parent of school age children; previously ten years working in a medical school in an administrative role and 5 years working in the drug and alcohol sector	
PPI reviewer 6. Lay representative for several healthcare-related professional bodies and involved in health research at several universities; family-carer for over 35 years; has had over 6 years of involvement with a mental and community health as a carer	
PPI reviewer 7. Retired university administrator; a parent and carer for elderly parents.	

Table D. Scoring for likelihood that the patient-reported scenario is potentially-harmful preventable-problem

Score	How likely do you think it is the patient was correct in thinking that their health might be worsened, or actually was made worse, because of a mistake or a problem in primary care that could have been prevented? Choose from the options below.
5	Very likely or certain (75-100% confident is a potentially-harmful preventable-problem)
4	Probably (50-74% confident is a potentially-harmful preventable-problem)
3	Possibly (25-49% confident is a potentially-harmful preventable-problem)
2	Unlikely (bottom 25% confident is a potentially-harmful preventable-problem)
1	Definitely not a potentially unsafe event (0% chance is a potentially-harmful preventable-problem)
-	Insufficient information
-	Don’t know
-	Other - add text at end of row

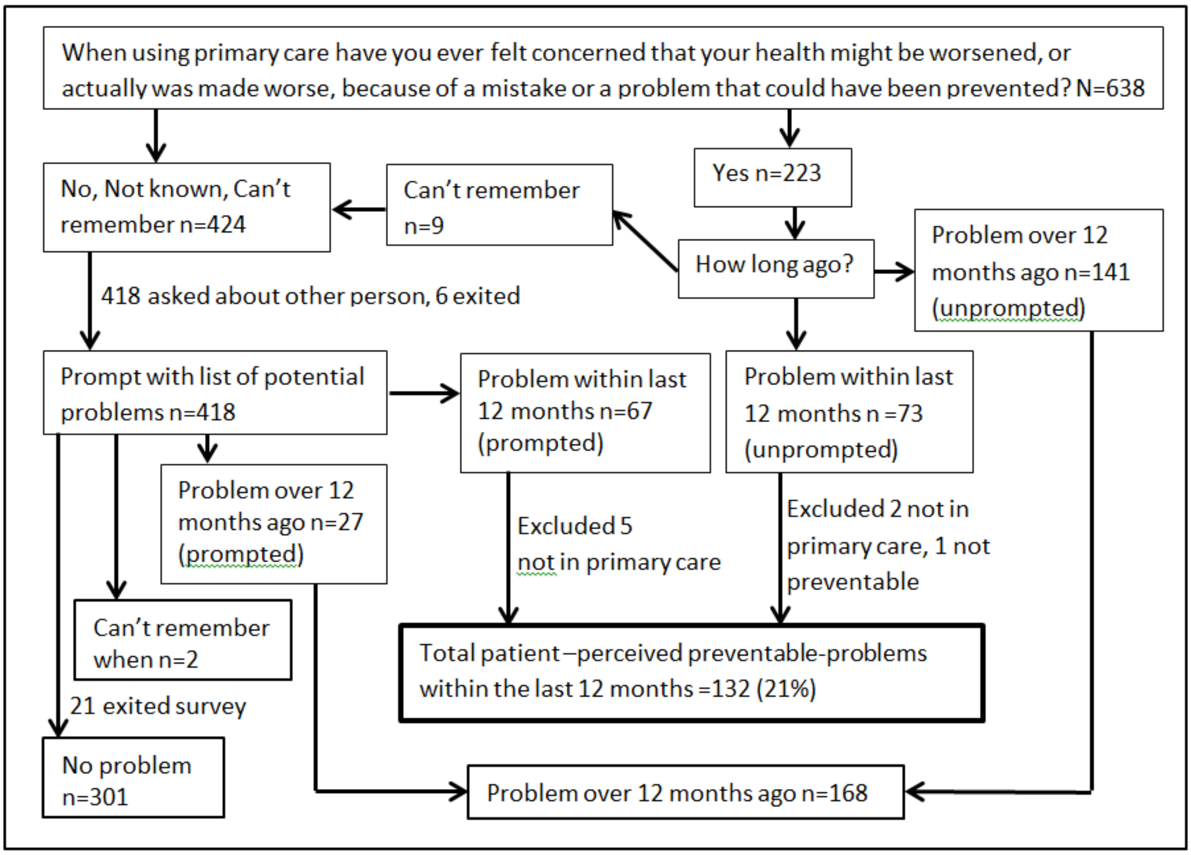

Figure A. Flow chart of participants who reported a potentially-unsafe preventable-problem in primary care through the online pilot survey

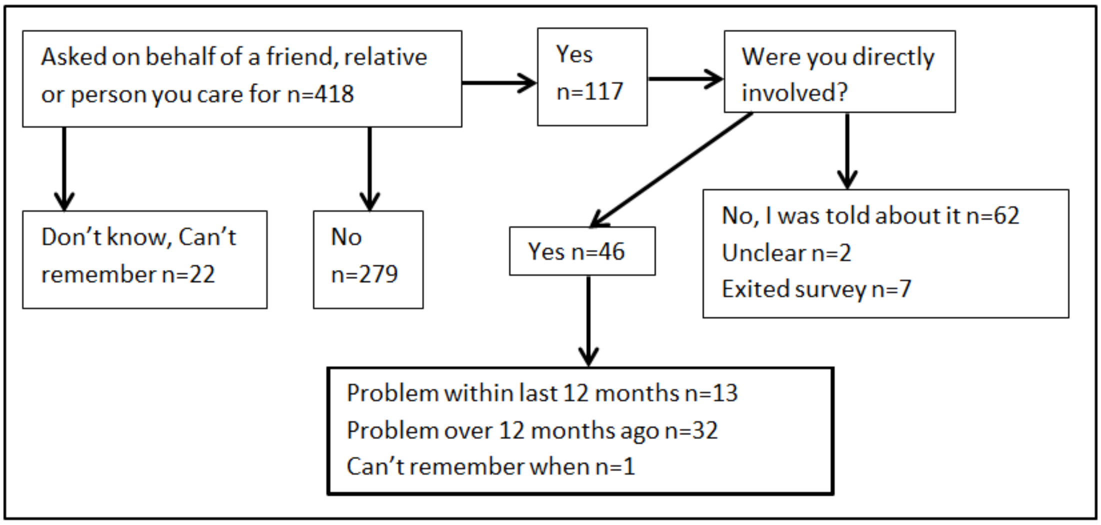

Figure B. Flow chart of participants who reported a potentially-unsafe preventable-problem in primary care on behalf of another person through the online pilot survey

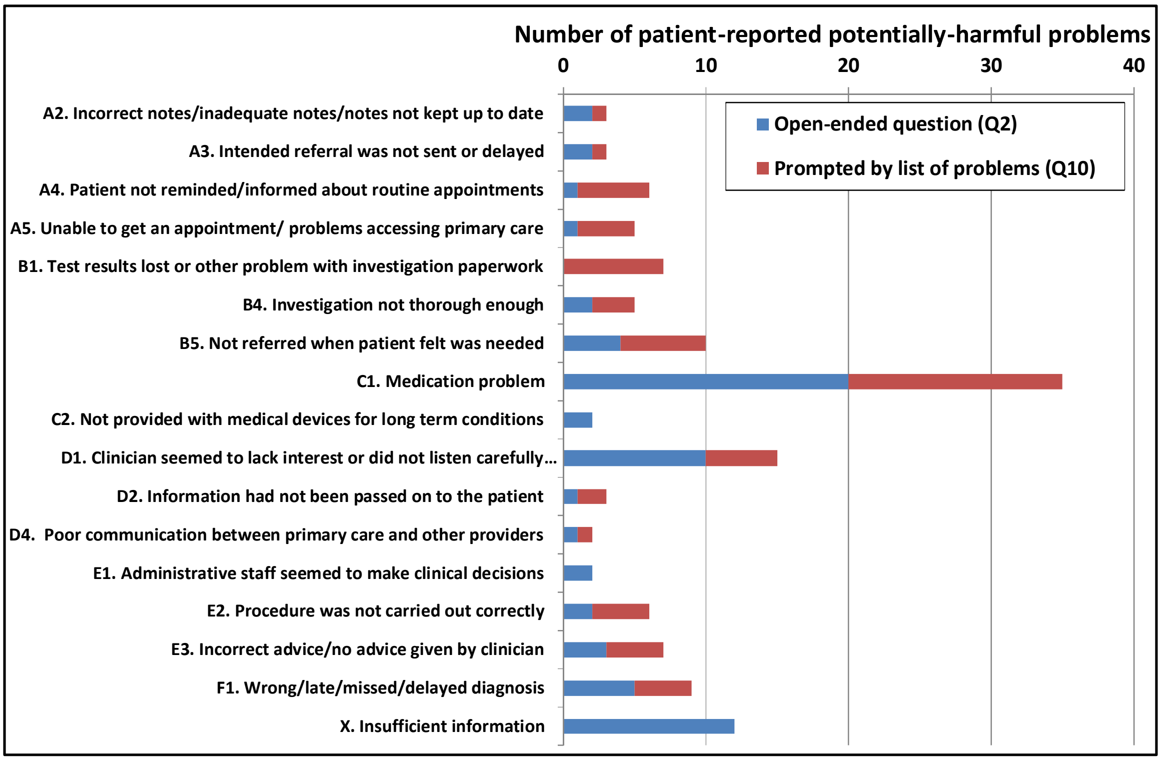

Fig C. Numbers of patient-perceived problems occurring in the last 12 months categorised according to the patient's description (see Table 2) and route through survey *i.e.* originated from open-ended question (Q2) or prompted by list of potential safety problems (Q10)

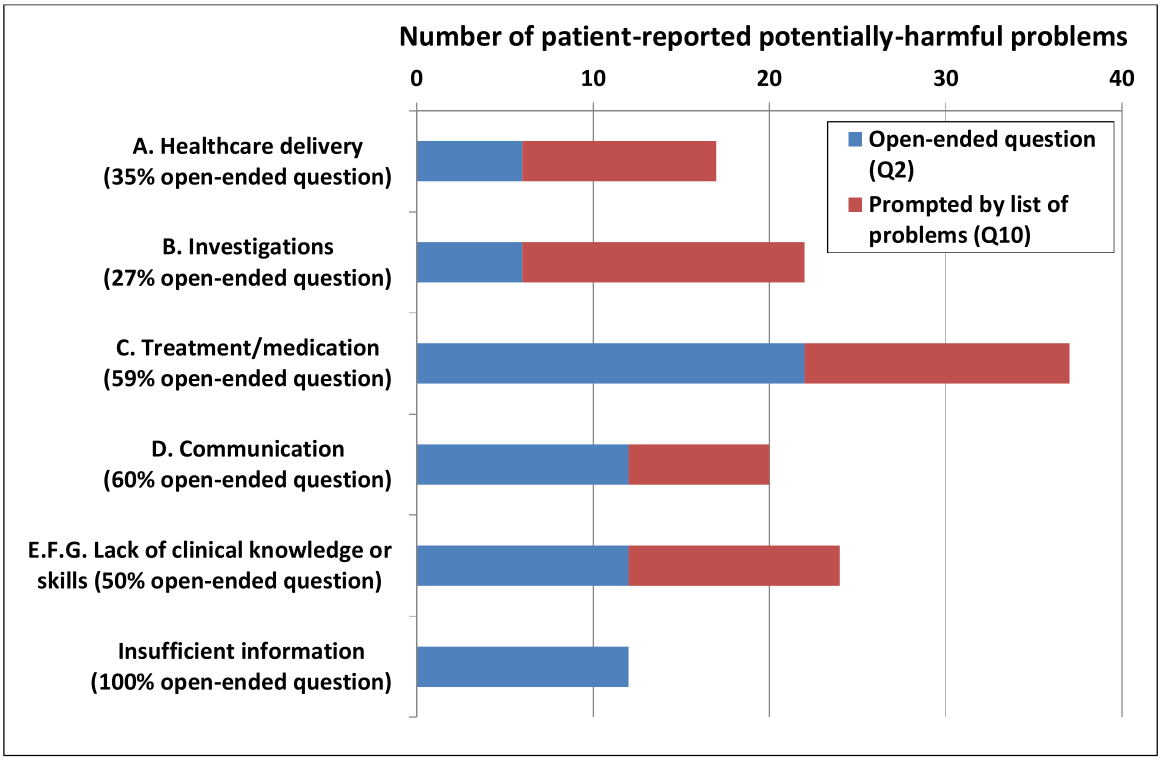

Fig D. Numbers of patient-perceived problems occurring in the last 12 months categorised according to the patient's description coded at a higher level (see Table 2) and route through survey *i.e.* originated from open-ended question (Q2) or prompted by list of potential safety problems (Q10)
