# Supplementary material for: Development and piloting of a survey to estimate the frequency and nature of potentially harmful preventable problems in primary care from a UK patient’s perspective
Source: BMJ Open. 2018 Feb 3;8(2):e017786. doi: 10.1136/bmjopen-2017-017786 (PMC5829776; doi:10.1136/bmjopen-2017-017786)
Supplement: Supplementary file 2 [file bmjopen-2017-017786supp002.pdf]

Appendix 2. Boxes 1 to 15

Patient reported scenarios occurring during the past 12 months that GPs scored as higher likelihood to be a potentially-unsafe preventable-problem in primary care (median score is higher than "possibly" and at least 2 GPs gave a score or one GP scored "very likely or certain")

Scenario1. GP surgery

Briefly describe the mistake or problem and how it happened. *"Prescription drug, anti-inflammatory for arthritis, caused acute stomach pains & violent vomiting. Repeat prescription for twelve years without any discussion."*

Could the mistake or problem have been avoided? If so how? "*Possible discussion about dangers of continuous taking of prescription drugs, which in the event were stopped after the incident."*

Were you able to talk about the mistake or problem with anybody working in the primary care service? *"No I did not notice the mistake or problem at the time"*

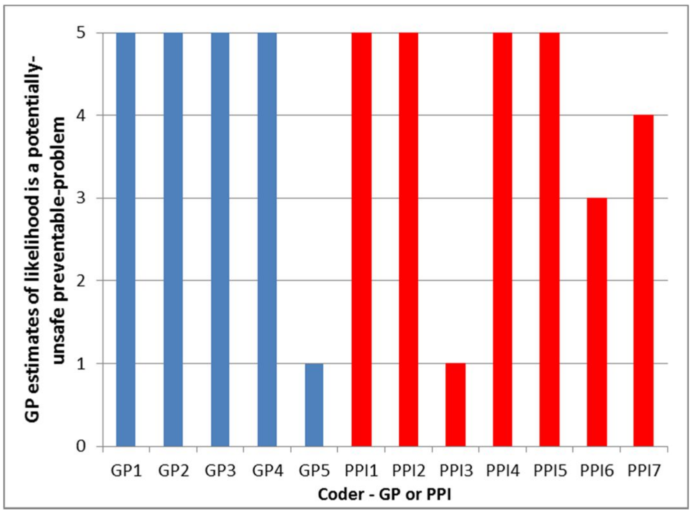

5=very likely or certain, 4=probably, 3=possibly, 2=unlikely, 1=definitely not, 0 = insufficient information or don't know

Patient-reported prospect of harm: suspected your health has been made worse by a problem or error that could have been prevented

Patient-perspective problem-type code: C1.1.3 Long term or continued prescribing without review or consideration of long term or side effects

Scenario2. GP surgery

Briefly describe the mistake or problem and how it happened. *"Insulin type was changed by specialist but previous insulin prescribed by GP as notes had not been updated"*

Could the mistake or problem have been avoided? If so how? "*Yes GP notes should have been updated with new medication"*

Were you able to talk about the mistake or problem with anybody working in the primary care service? *"Practice manager resolved the problem and apologised"*

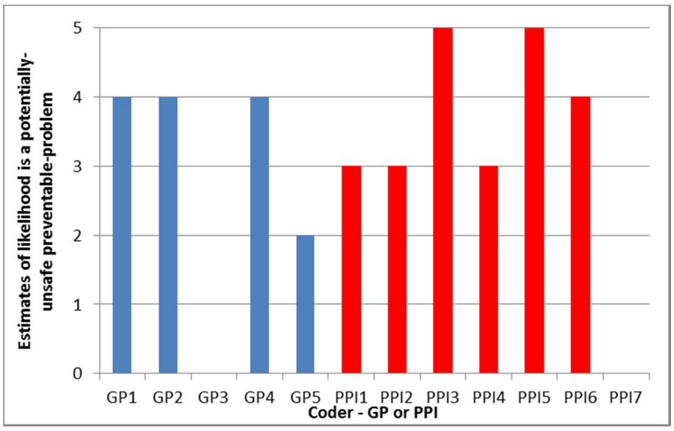

5=very likely or certain, 4=probably, 3=possibly, 2=unlikely, 1=definitely not, 0 = insufficient information or don't know

Patient-reported prospect of harm: prompted via Q10 (Box 1 main paper)

Patient-perspective problem-type code: A2. Incorrect notes/inadequate notes/notes not kept up to date; C1.1.6 Out of date repeat prescription mistakenly re-issued

Scenario3. GP surgery

Briefly describe the mistake or problem and how it happened. *"Two out of three Doctors not listening to what I was asking; April I had two big bleeds from my Penis, Doctor 1 did a test and gave antibiotics. Went to 2nd Doctor for Diabetic check and told him of problem - nothing except another test come back in ten days. Went to the third doctor who said the test didn't show anything but when I mentioned my feelings about a problem, he look and said yes you do have a problem. In 2 weeks I was in having tests and 3 operations for cancer."*

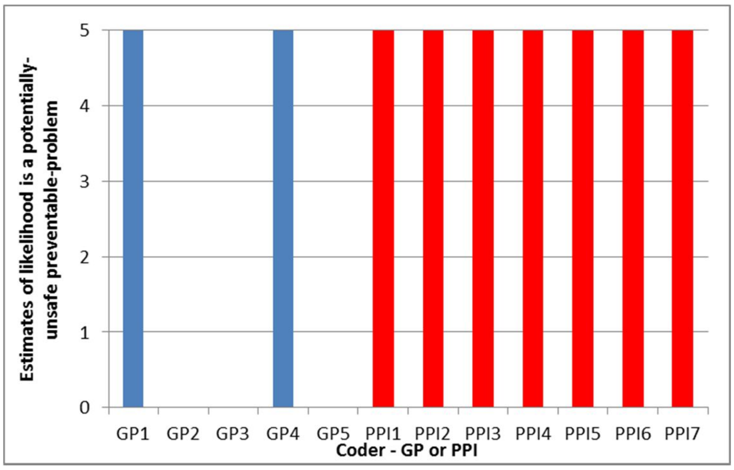

5=very likely or certain, 4=probably, 3=possibly, 2=unlikely, 1=definitely not, 0 = insufficient information or don't know

Could the mistake or problem have been avoided? If so how? "*Listen to me"*

Were you able to talk about the mistake or problem with anybody working in the primary care service? *"No, I could not find anybody with whom I could discuss the mistake or problem (The third doctor was amazing with me. He said to keep in touch and if I had any problems to ring him and he still wants me to ring him after my three operations.)"*

Patient-reported prospect of harm: suspected your health has been made worse by a problem or error that could have been prevented

Patient-perspective problem-type code: D1. Clinician seemed to lack interest in the patient's health problem or did not listen carefully enough; F1. Wrong/late/missed/delayed diagnosis

Scenario4. GP surgery

Briefly describe the mistake or problem and how it happened. *"Changed diabetes medication to an alternative which my notes from 1980's should show I respond badly to"*

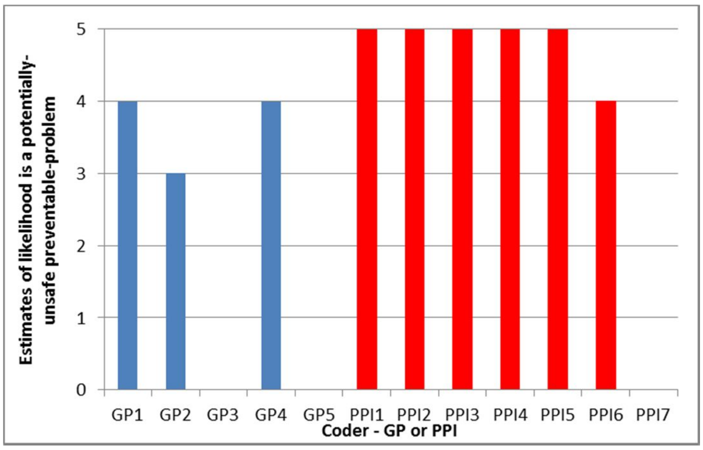

5=very likely or certain, 4=probably, 3=possibly, 2=unlikely, 1=definitely not, 0 = insufficient information or don't know

Could the mistake or problem have been avoided? If so how? "*Read the notes on every medication change but unfortunately that is unrealistic under the time restrictions on GP's. Put early notes on-line and flag medication allergies/problems."*

Were you able to talk about the mistake or problem with anybody working in the primary care service? *"Yes, my own GP who had returned from holiday"*

Patient-reported prospect of harm: suspected your health has been made worse by a problem or error that could have been prevented

Patient-perspective problem-type code: C1.1.4 Prescribed drug when should have known contra-indicated *e.g.* patient had informed clinician of allergy, adverse reaction or it was in the records

Scenario5. GP surgery

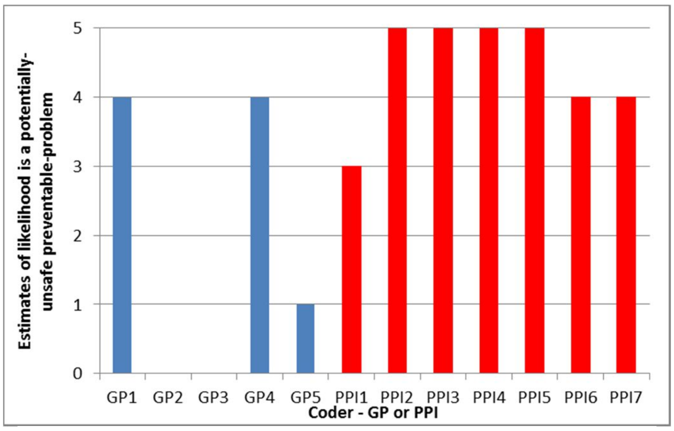

5=very likely or certain, 4=probably, 3=possibly, 2=unlikely, 1=definitely not, 0 = insufficient information or don't know

Briefly describe the mistake or problem and how it happened. *"Told the GP the medication was making my hair fall out & he kept me on it for another 3 months. I had to see another GP to get him to change my medication. In the meantime I have lost 3/4 of my hair. Not sure if it will ever grow back."*

Could the mistake or problem have been avoided? If so how? "*yes, by the GP listening to what I was saying."*

Were you able to talk about the mistake or problem with anybody working in the primary care service? *"Yes, GP"*

Patient-reported prospect of harm: suspected your health has been made worse by a problem or error that could have been prevented

Patient-perspective problem-type code: D1. Clinician seemed to lack interest in the patient's health problem or did not listen carefully enough; C1.1.3 Long term or continued prescribing without review or consideration of long term or side effects

Scenario6. GP surgery

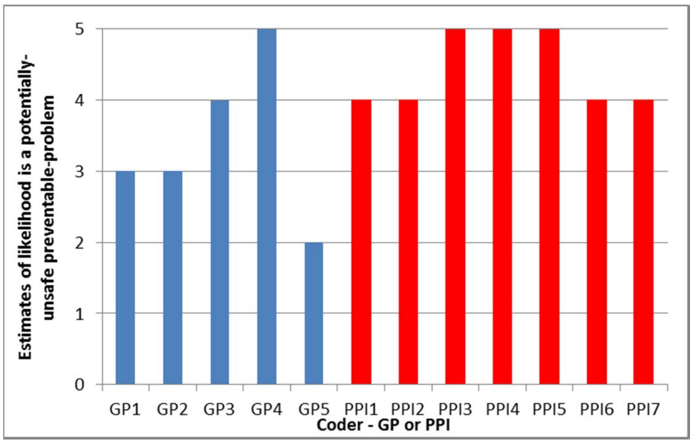

5=very likely or certain, 4=probably, 3=possibly, 2=unlikely, 1=definitely not, 0 = insufficient information or don't know

Briefly describe the mistake or problem and how it happened. *"Successfully treated for prostate cancer 2006 but suffered some loss of sexual performance; Viagra recommended BUT I take isosorbide nitrate for a following heart attack; the two are contradictory and could produce further heart problems. A routine diabetes check-up at which the sexual problem was discussed saw an automatic prescribing of Viagra; obviously without reference to my medical records."*

Could the mistake or problem have been avoided? If so how? "*Read the medical notes."*

Were you able to talk about the mistake or problem with anybody working in the primary care service? *"No; I felt I was going to cause trouble"*

Patient-reported prospect of harm: prompted via Q10 (Box 1 main paper)

Patient-perspective problem-type code: C1.1.1 Prescribed wrong or inappropriate drug

Scenario7. GP surgery

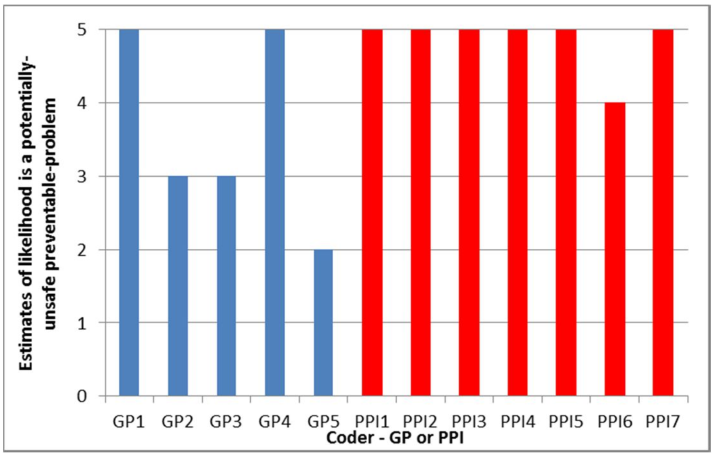

5=very likely or certain, 4=probably, 3=possibly, 2=unlikely, 1=definitely not, 0 = insufficient information or don't know

Briefly describe the mistake or problem and how it happened. *"I was given steroids for a chest infection but not alerted to the fact they make your sugars go massively high! Within a few hours I was high and not able to bring them down, fearing a DKA I headed for the hospital to correct a very easily avoidable issue. I also attended my GP 6 years ago to be given strong antacids for pain in my stomach that was actually a DKA I was admitted to hospital a few hours later! The GP never even suggested it could be linked to my diabetes and as it was my first DKA I had no idea that's how they can feel"*

Could the mistake or problem have been avoided? If so how? "*Both could have been avoided The steroids - if the prescribing nurse had considered my diabetes I'd have been given proper advice as to how to deal with them as a diabetic or given different meds. The DKA simple questions or explanation as to how DKAs can present would have made me family and the doctor realise I was in trouble."*

Were you able to talk about the mistake or problem with anybody working in the primary care service? *"I wrote a letter to the surgery concerning the steroids anonymously to alert them of my concern and the DKA. I was too poorly to even consider seeking correction or explanation"*

Patient-reported prospect of harm: health was actually made worse by a problem or error that could have been prevented

Patient-perspective problem-type code: C1.1.4 Prescribed drug when should have known contra-indicated *e.g.* patient had informed clinician of allergy, adverse reaction or it was in the records; E3. Incorrect advice/no advice given by clinician

Scenario8. GP surgery

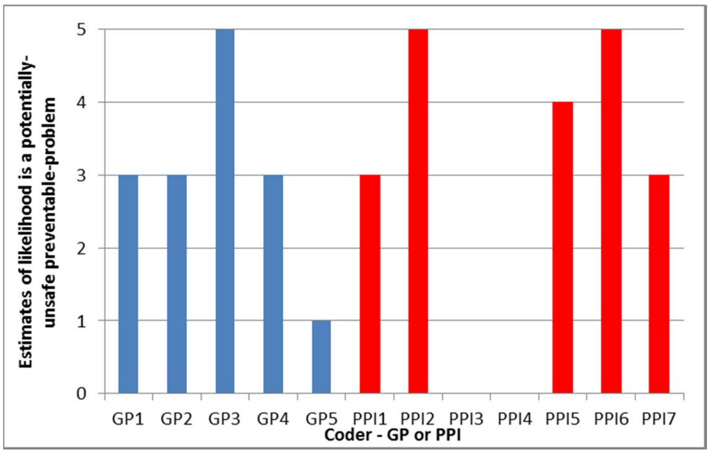

5=very likely or certain, 4=probably, 3=possibly, 2=unlikely, 1=definitely not, 0 = insufficient information or don't know

Briefly describe the mistake or problem and how it happened. *"reception staff making clinical decisions which were at odds with what had been discussed with my GP"*

Could the mistake or problem have been avoided? If so how? "*Yes, reception staff shouldn't be making clinical decisions"*

Were you able to talk about the mistake or problem with anybody working in the primary care service? *"No, had the opportunity but did not feel comfortable to discuss the mistake or problem"*

Patient-reported prospect of harm: suspected your health has been made worse by a problem or error that could have been prevented

Patient-perspective problem-type code: E1. Administrative staff seemed to make clinical decisions

Scenario9. Pharmacist

Briefly describe the mistake or problem and how it happened. *"I was given a medicine belonging to somebody else as part of my monthly repeat prescription"*

Could the mistake or problem have been avoided? If so how? "*More care and attention when checking"*

Were you able to talk about the mistake or problem with anybody working in the primary care service? *"Yes, pharmacist"*

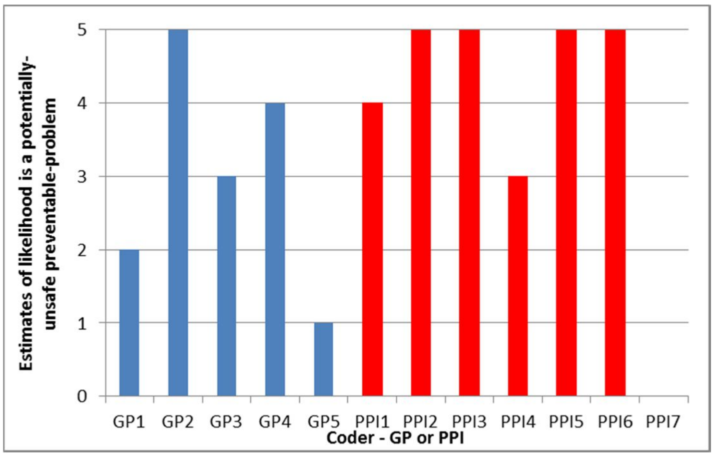

5=very likely or certain, 4=probably, 3=possibly, 2=unlikely, 1=definitely not, 0 = insufficient information or don't know

Patient-reported prospect of harm: prompted via Q10 (Box 1 main paper)

Patient-perspective problem-type code: C1.3.3
Wrong or inadequate advice about drug effects or how to use

Scenario10. GP surgery

Briefly describe the mistake or problem and how it happened. *"Poor diabetic annual review, foot check not correctly done just tested my foot pulses and nothing else"*

Could the mistake or problem have been avoided? If so how? "*Better training of staff"*

Were you able to talk about the mistake or problem with anybody working in the primary care service? *"No, had the opportunity but did not feel comfortable to discuss the mistake or problem"*

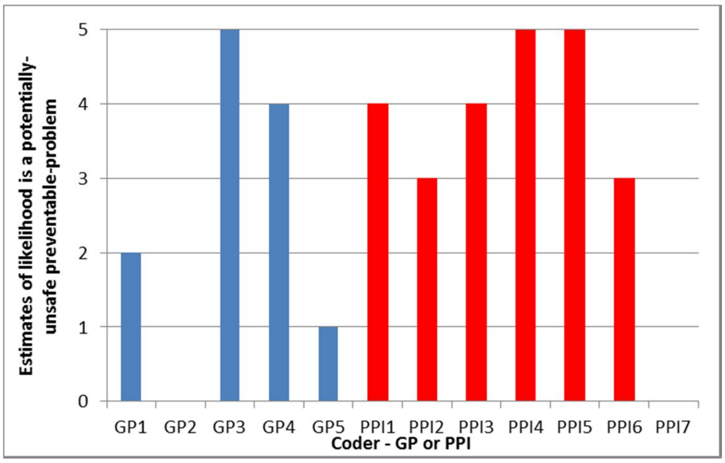

5=very likely or certain, 4=probably, 3=possibly, 2=unlikely, 1=definitely not, 0 = insufficient information or don't know

Patient-reported prospect of harm: suspected your health has been made worse by a problem or error that could have been prevented

Patient-perspective problem-type code: E2. Procedure was not carried out correctly

Scenario11. GP surgery

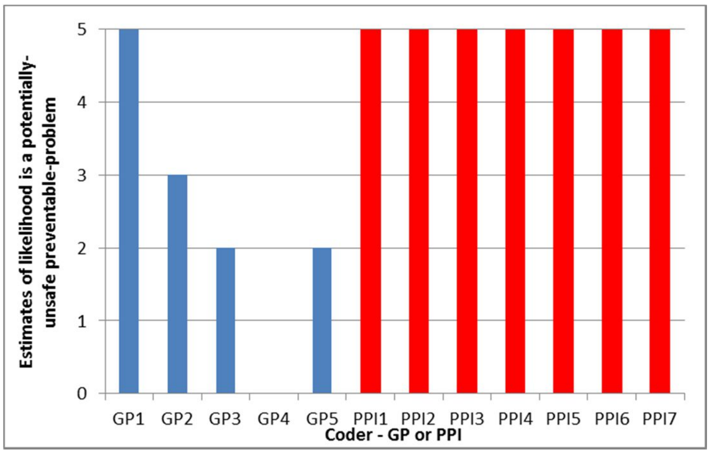

5=very likely or certain, 4=probably, 3=possibly, 2=unlikely, 1=definitely not, 0 = insufficient information or don't know

Briefly describe the mistake or problem and how it happened. *"Prior to a pain killing injection into my knee, I asked the GP who suggested the injection AND the GP who carried out the injection whether, as someone living with Type 1 diabetes, it would have any effect on my blood glucose levels. On both occasions, I was given an unequivocal No . In the event, within a few hours of the injection, my blood glucose rose significantly and remained high for several days. I felt unable to eat anything for 24 hours while I took on more and more insulin in order to bring my glucose levels down - I did not want to go to sleep that night simply because of the massive amount of insulin in my system."*

Could the mistake or problem have been avoided? If so how? "*Yes. I feel that both GPs should have a knowledge about the side effects of drugs they prescribe, administer and recommend."*

Were you able to talk about the mistake or problem with anybody working in the primary care service? *"No I could not find anybody with whom I could discuss the mistake or problem"*

Patient-reported prospect of harm: your health has been made worse by a problem or error that could have been prevented

Patient-perspective problem-type code: E3. Incorrect advice/no advice given by clinician

Scenario12. GP surgery

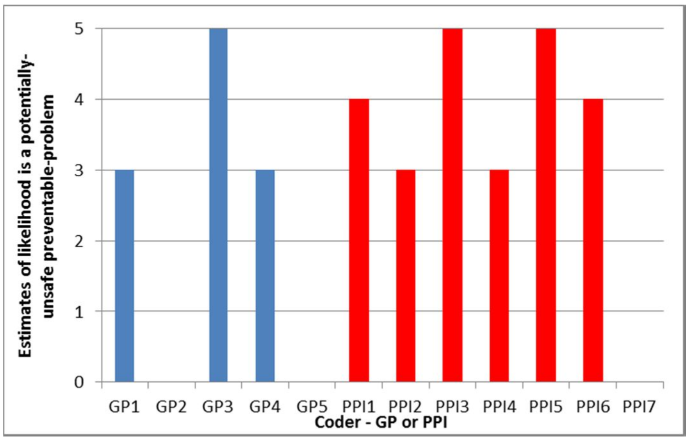

5=very likely or certain, 4=probably, 3=possibly, 2=unlikely, 1=definitely not, 0 = insufficient information or don't know

Briefly describe the mistake or problem and how it happened. *"GP completely overlooked symptoms and prescribed antibiotic after antibiotic without investigation or referral"*

Could the mistake or problem have been avoided? If so how? "*Yes by listening to history of complaints, carrying out appropriate tests instead of just giving antibiotics"*

Were you able to talk about the mistake or problem with anybody working in the primary care service? *"No I did not notice the mistake or problem at the time"*

Patient-reported prospect of harm: prompted via Q10 (Box 1 main paper)

Patient-perspective problem-type code: D1. Clinician seemed to lack interest in the patient's health problem or did not listen carefully enough; F1. Wrong/late/missed/delayed diagnosis

Scenario13. GP surgery

Briefly describe the mistake or problem and how it happened. *"Several times prescriptions have been incorrectly issued due to similar names for drugs or the same name with different strengths"*

Could the mistake or problem have been avoided? If so how? "*Yes, by more accurate or double data entry. Now solved by self-request using web systems."*

Were you able to talk about the mistake or problem with anybody working in the primary care service? "Yes, *they did not want to know or seem to care unless a formal complaint was made"*

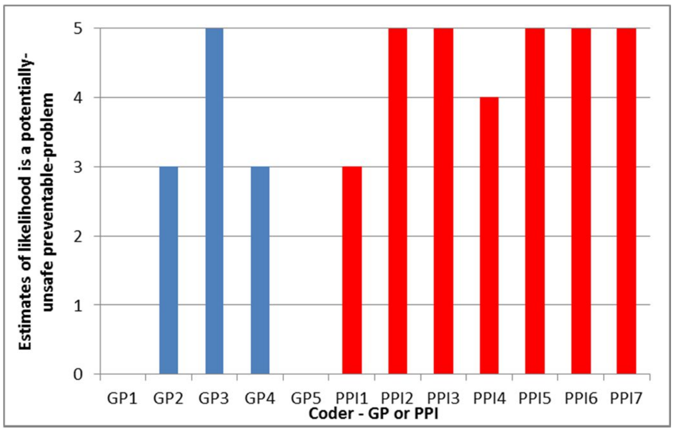

5=very likely or certain, 4=probably, 3=possibly, 2=unlikely, 1=definitely not, 0 = insufficient information or don't know

Patient-reported prospect of harm: your health has been made worse by a problem or error that could have been prevented

Patient-perspective problem-type code: C1.1.5 Repeat prescription unintentionally changed

Scenario14. GP surgery

Briefly describe the mistake or problem and how it happened. *"A simple error occurred with an incorrect prescription. When I tried to bring this to the attention of the receptionist she treated me with disdain and in a challenging manner. She then proceeded to start to read my notes aloud in the public reception area. I felt that this was unacceptable behaviour. When I tried to tackle the receptionist about her behaviour I felt as if I was under threat. It caused me to feel very stressed, frustrated and ill tempered."*

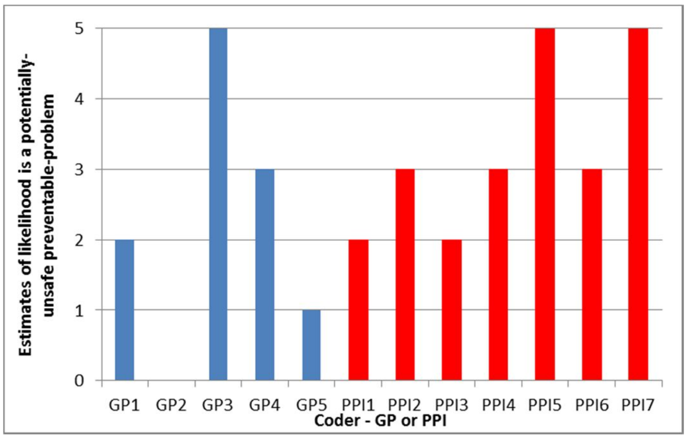

5=very likely or certain, 4=probably, 3=possibly, 2=unlikely, 1=definitely not, 0 = insufficient information or don't know

Could the mistake or problem have been avoided? If so how? "*If the receptionist had been willing to listen to what I was saying."*

Were you able to talk about the mistake or problem with anybody working in the primary care service? *"I did speak to a lady who said she was the practice manager but I felt that they were not interested in resolving the problem"*

Patient-reported prospect of harm: suspected your health has been made worse by a problem or error that could have been prevented

Patient-perspective problem-type code: D3. Communication problem between patient and primary care staff; C1 Medication error not otherwise specified /other problem

Scenario15. GP Surgery

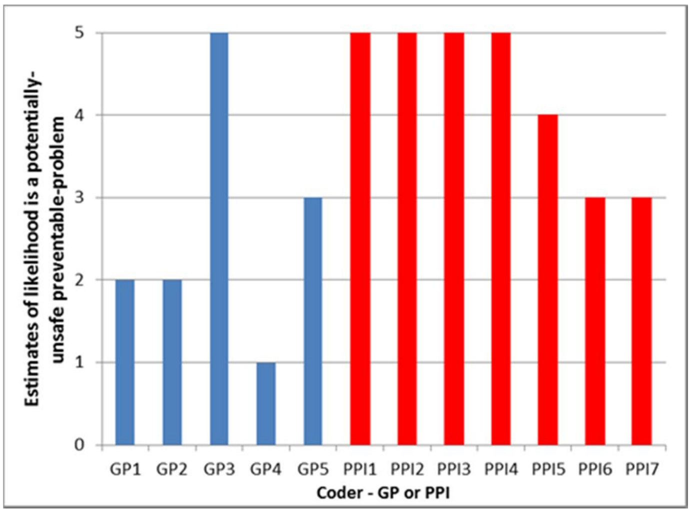

5=very likely or certain, 4=probably, 3=possibly, 2=unlikely, 1=definitely not, 0 = insufficient information or don't know

Briefly describe the mistake or problem and how it happened. "*Went to see GP because I feared the pain in one of my legs may have been Peripheral Artery Disease - hardening of the arteries, having had a (non-blood) relative who suffered from this and subsequently died - of a heart attack. Oh yes, said the GP, well, you will have it won't you? Why? I asked expecting her to say eg because you are a smoker, or maybe my age (65) or something else I wasn't aware of. But what she actually told me was 'Because you are a diabetic!' Whaaat? I exclaimed - you mean ALL diabetics will inevitably get this, and there's no way to prevent it? Yes she said and shrugged. I said 'Thanks for nothing then' and left. Instead I left, came home and went straight on-line to make an appointment with someone more sensible, which I did and after taking my leg/ankle pulses and BPs etc - he chatted to me and said he would refer me for a cardiology consultation at the hospital. This IS what I expected in the first place and now it IS being taken care of***.**"

Could the mistake or problem have been avoided? If so how? "*By training the GP properly in the first place*"

Were you able to talk about the mistake or problem with anybody working in the primary care service? "? *"I explained to GP 2 But I don't know what if anything was done about it, or how I could find that out."*

Patient-reported prospect of harm: your health has been made worse by a problem or error that could have been prevented

Patient-perspective problem-type code: D1. Clinician seemed to lack interest in the patient's health problem or did not listen carefully enough

Appendix 2. Boxes 16 to 23

Patient reported scenarios occurring during the past 12 months that PPIs scored as higher likelihood to be a potentially-unsafe preventable-problem in primary care compared with GPs

Scenario16. GP Surgery

Briefly describe the mistake or problem and how it happened. *"I had a severe reaction to Atorvastatin after a dose increase so much so that I was almost immobile and took 4 months to recover"*

Could the mistake or problem have been avoided? If so how? *"According to guidelines I should have been on the increased dose - it took a long time to convince the GP that I needed blood tests to find out why I couldn't walk. My GP was very hesitant to admit that I did have a reaction to statins."*

Were you able to talk about the mistake or problem with anybody working in the primary care service? **"***No I could not find anybody with whom I could discuss the mistake or problem. It was not really the GPs fault per se, just took a lot of convincing that there was a problem"*

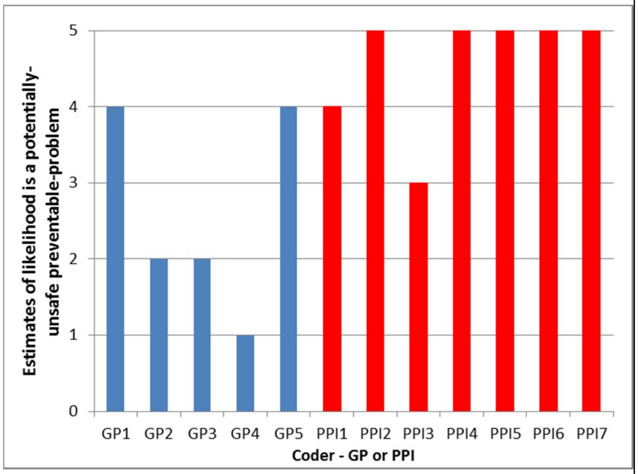

5=very likely or certain, 4=probably, 3=possibly, 2=unlikely, 1=definitely not, 0 = insufficient information or don't know

Patient-reported prospect of harm: health could have been made worse had someone not noticed a problem or error

Patient-perspective problem-type code: C1.1.3 Long term or continued prescribing without review or consideration of long term or side effects

Scenario17. GP Surgery

Briefly describe the mistake or problem and how it happened. *"Doctor kept saying I had vitamin deficiency B1, it turned out I had peripheral neuropathy which is very painful"*

Could the mistake or problem have been avoided? If so how? *"I just needed the proper medication to help"*

Were you able to talk about the mistake or problem with anybody working in the primary care service? *"Just saw another Doctor and she knew straight away what the problem was - she was experienced with Diabetic problems. Yes had the opportunity but did not feel comfortable to discuss the mistake or problem"*

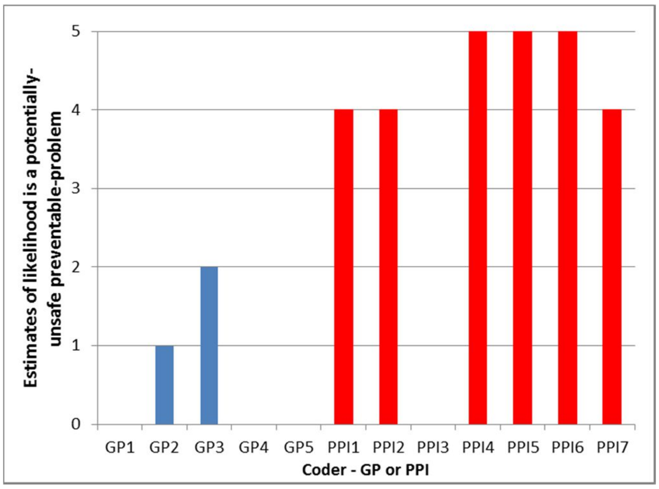

5=very likely or certain, 4=probably, 3=possibly, 2=unlikely, 1=definitely not, 0 = insufficient information or don't know

Patient-reported prospect of harm: prompted via Q10 (Box 1 main paper)

Patient-perspective problem-type code: F1. Wrong/late/missed/delayed diagnosis

Scenario18. GP Surgery

Briefly describe the mistake or problem and how it happened. *"Incapable diabetic doctor trying to take blood out the back of my hand haphazardly, not listening and resulting in me fitting and the student watching having to get help."*

Could the mistake or problem have been avoided? If so how? *"Yes. By listening to me"*

Were you able to talk about the mistake or problem with anybody working in the primary care service? *"No I could not find anybody with whom I could discuss the mistake or problem"*

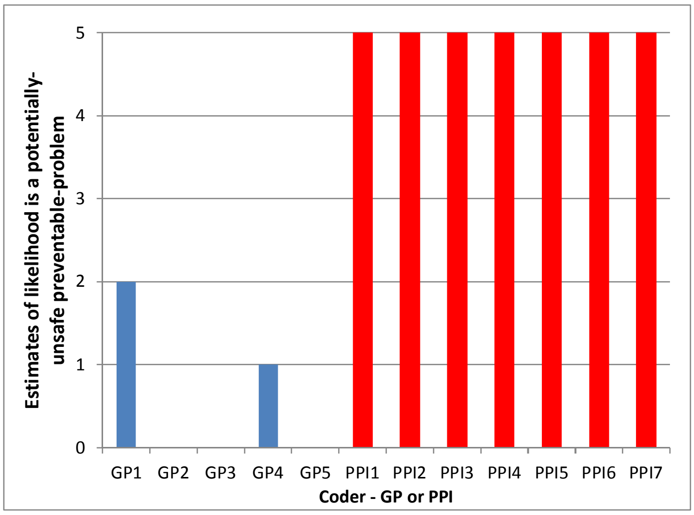

5=very likely or certain, 4=probably, 3=possibly, 2=unlikely, 1=definitely not, 0 = insufficient information or don't know

Patient-reported prospect of harm: prompted via Q10 (Box 1 main paper)

Patient-perspective problem-type code: E2. Procedure was not carried out correctly; D1. Clinician seemed to lack interest in the patient's health problem or did not listen carefully enough

Scenario19. Dental Surgery

Briefly describe the mistake or problem and how it happened. *"I had an infection under my wisdom tooth. They agreed that the only way to solve the problem was to take the tooth out. They gave me an appointment to do this in 6 weeks. I am a type 1 diabetic and the infection was affecting my blood sugars and I was concerned that I would have to go to A&E if my blood sugars continued to rise due to the infection. It would have affected my health if I had not paid to go to a private dentist."*

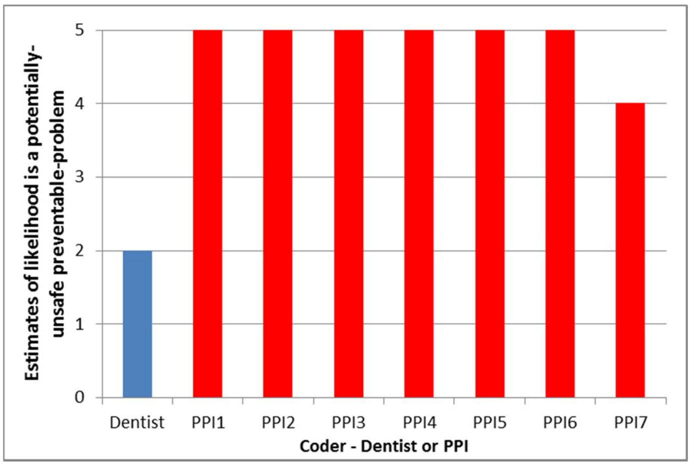

5=very likely or certain, 4=probably, 3=possibly, 2=unlikely, 1=definitely not, 0 = insufficient information or don't know

Could the mistake or problem have been avoided? If so how? *"They could have taken out the tooth straight away. I was happy to wait at the emergency dentist for them to do this."*

Were you able to talk about the mistake or problem with anybody working in the primary care service? *"I explained but they said I would have to wait. They also asked if I needed a sugary drink when I said that my sugars were high so I was too scared to eat and had not eaten in 12hrs. It was clear they didn't understand diabetes."*

Patient-reported prospect of harm: health could have been made worse had someone not noticed a problem or error

Patient-perspective problem-type code: A5. Unable to get an appointment/other problems with making appointment

Scenario20. Dental Surgery

Briefly describe the mistake or problem and how it happened. *"Caries, cavities and problem with crown not diagnosed or treated"*

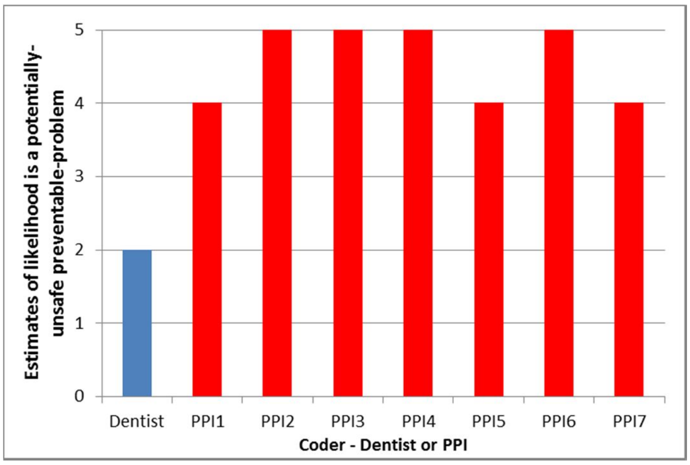

5=very likely or certain, 4=probably, 3=possibly, 2=unlikely, 1=definitely not, 0 = insufficient information or don't know

Could the mistake or problem have been avoided? If so how? *"Better dentist & not working to tight time-scale imposed by company owning dental surgery"*

Were you able to talk about the mistake or problem with anybody working in the primary care service? *"No I could not find anybody with whom I could discuss the mistake or problem"*

Patient-reported prospect of harm: prompted via Q10 (Box 1 main paper)

Patient-perspective problem-type code: C3. Problem with dental treatment or diagnosis

Scenario21. GP Surgery

Briefly describe the mistake or problem and how it happened. *"Using the summary on discharge from hospital, one GP transcribed incorrectly on to my electronic notes ie size of ovarian cyst was 7.5cms and he put 7.5 mms. Another GP requested diagnostic bone density scan but either forgot or did not record it and she ended up questioning why I had it and who requested it. She also referred me for an orthopedic consultation then said I was not funded for the steroid injection put into my swollen elbows."*

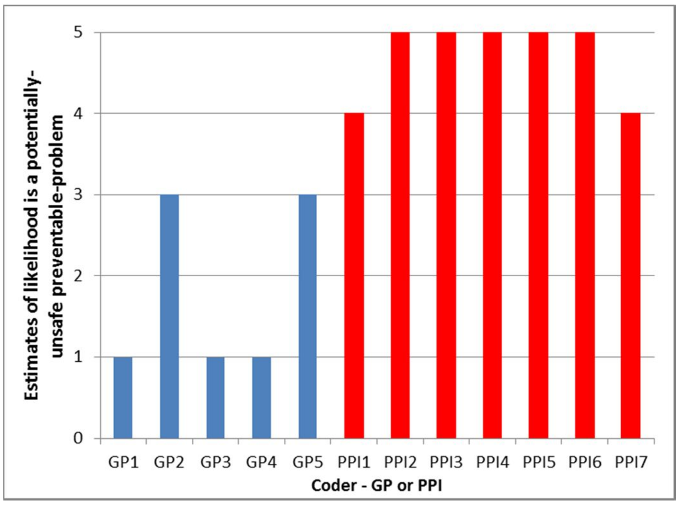

Could the mistake or problem have been avoided? If so how? *"Yes"*

5=very likely or certain, 4=probably, 3=possibly, 2=unlikely, 1=definitely not, 0 = insufficient information or don't know

Were you able to talk about the mistake or problem with anybody working in the primary care service? *"I was too scared to discuss my concerns for fear of being labelled a trouble maker"*

Patient-reported prospect of harm: health could have been made worse had someone not noticed a problem or error

Patient-perspective problem-type code: A2. Incorrect notes/inadequate notes/notes not kept up to date

Scenario22. GP Surgery

Briefly describe the mistake or problem and how it happened. *"GP prescribed pills, but then got phone call saying not to take them"*

Could the mistake or problem have been avoided? If so how? *"Not sure"*

Were you able to talk about the mistake or problem with anybody working in the primary care service? *"No I was not concerned about the problem"*

Patient-reported prospect of harm: prompted via Q10 (Box 1 main paper)

Patient-perspective problem-type code: C1. Medication problem

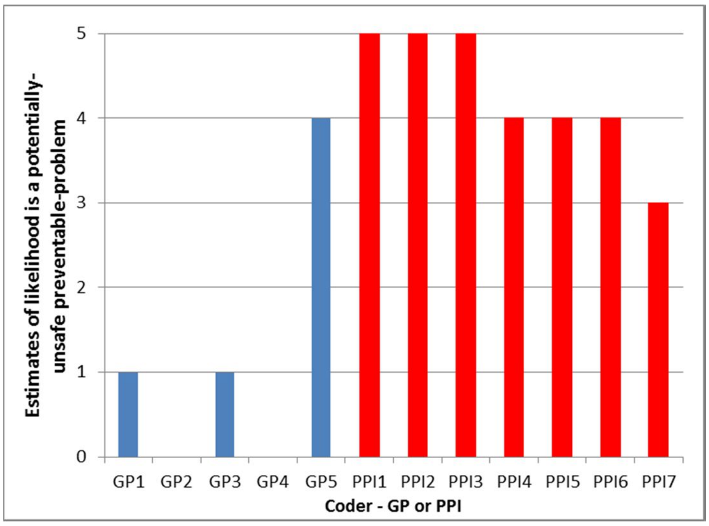

5=very likely or certain, 4=probably, 3=possibly, 2=unlikely, 1=definitely not, 0 = insufficient information or don't know

Scenario23. GP Surgery

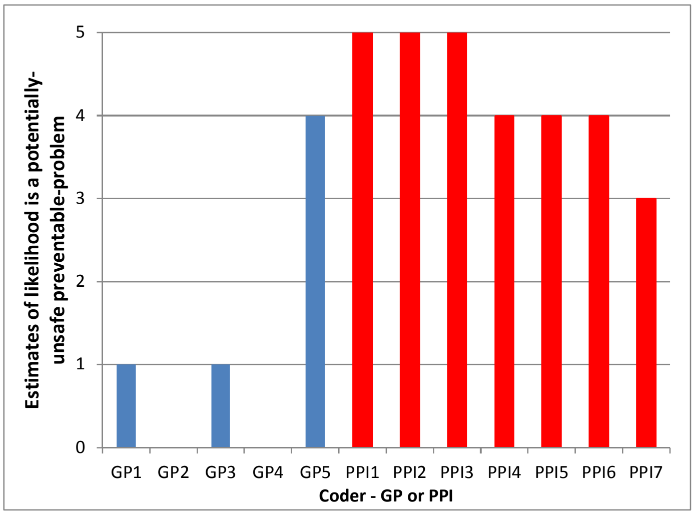

5=very likely or certain, 4=probably, 3=possibly, 2=unlikely, 1=definitely not, 0 = insufficient information or don't know

Briefly describe the mistake or problem and how it happened. *"I had a burst appendix and peritonitis, something that even a scan couldn't detect adequately. My first visit to GP was when I said I think I have appendicitis, no other symptoms only the pain. It was ten days before seeing a consultant, a further 10 days to have a scan, then 2 weeks to be told that I had a lump on my colon which is what my GP had said 5 weeks previously. It was a further 2 weeks before I had surgery."*

Could the mistake or problem have been avoided? If so how? *"If my GP had referred me for a scan immediately it would have saved 3 weeks out of the seven. It was two weeks from scan to results and I hear that is usual, but they're not looking at them for 2 weeks"*

Were you able to talk about the mistake or problem with anybody working in the primary care service? *"Had the outcome been different my widow might have pursued the matter further. The system is at fault rather than any individual."*

Patient-reported prospect of harm: your health has been made worse by a problem or error that could have been prevented

Patient-perspective problem-type code: B5. Not referred when patient felt was needed
