# Supplementary material for: Development and piloting of a survey to estimate the frequency and nature of potentially harmful preventable problems in primary care from a UK patient’s perspective
Source: BMJ Open. 2018 Feb 3;8(2):e017786. doi: 10.1136/bmjopen-2017-017786 (PMC5829776; doi:10.1136/bmjopen-2017-017786)
Supplement: Author's manuscript [file bmjopen-2017-017786.draft_revisions.pdf]

BMJ Open

BMJ Open is committed to open peer review. As part of this commitment we make the peer review history of every article we publish publicly available.

When an article is published we post the peer reviewers' comments and the authors' responses online. We also post the versions of the paper that were used during peer review. These are the versions that the peer review comments apply to.

The versions of the paper that follow are the versions that were submitted during the peer review process. They are not the versions of record or the final published versions. They should not be cited or distributed as the published version of this manuscript.

BMJ Open is an open access journal and the full, final, typeset and author-corrected version of record of the manuscript is available on our site with no access controls, subscription charges or pay-per-view fees (http://bmjopen.bmj.com).

If you have any questions on BMJ Open's open peer review process please email editorial.bmjopen@bmj.com

BMJ Open

Development and piloting of a survey to estimate the frequency and nature of potentially-harmful preventable-problems in primary care from a UK patient's perspective

Journal:	*BMJ Open*
Manuscript ID	bmjopen-2017-017786
Article Type:	Research
Date Submitted by the Author:	16-May-2017
Complete List of Authors:	Stocks, Susan; University of Manchester, GM Patient Safety Translational Research Centre<br>Donnelly, Ailsa; University of Manchester, PRIMER<br>Esmail, Aneez; University of Manchester, School of Primary Care<br>Beresford, Joanne; Research User Group (RUG) of the NIHR Greater Manchester Primary Care Patient Safety Translational Research Centre<br>Gamble, Carolyn; University of Manchester, GM Patient Safety Translational Research Centre<br>Luty, Sarah; NHS Scotland<br>Deacon, Richard; St Gabriels Medical Centre<br>Danczak, Avril; Central and South Manchester Specialty Training Programme for General Practice<br>Mann, Nicola; University of Manchester, GMPSTRC<br>Townsend, David; University of Manchester, GM Patient Safety Translational Research Centre<br>Ashley, James; Division of Dentistry, School of Medical Sciences, University of Manchester<br>Bowie, Paul; NHS Scotland<br>Campbell, Stephen; University of Manchester, Centre for Primary Care
**Primary Subject Heading**:	Patient-centred medicine
Secondary Subject Heading:	Research methods, Patient-centred medicine, General practice / Family practice, Health services research
Keywords:	STATISTICS & RESEARCH METHODS, PRIMARY CARE, Quality in health care < HEALTH SERVICES ADMINISTRATION & MANAGEMENT

SCHOLARONE™
Manuscripts

Development and piloting of a survey to estimate the frequency and nature of potentially-harmful preventable-problems in primary care from a UK patient's perspective

S Jill Stocks *research fellow*[1], Ailsa Donnelly *public and patient involvement volunteer*[2], Aneez Esmail *professor*[1], Joanne Beresford *public and patient involvement volunteer*[2] Carolyn Gamble *public and patient involvement volunteer*[2], Sarah Luty *general practitioner*[3], Richard Deacon *general practitioner*[4], Avril Danczak *general practitioner*[5], Nicola Mann *general practitioner*[2], David Townsend *general practitioner*[2], James Ashley *dentist*[6], Paul Bowie *programme director*[7,8], Stephen M Campbell *professor*[1]

1. NIHR Greater Manchester Primary Care Patient Safety Translational Research Centre, Centre for Primary Care, Division of Population Health, Health Services Research and Primary Care, University of Manchester, Manchester M13 9PL, UK

2. Research User Group (RUG) of the NIHR Greater Manchester Primary Care Patient Safety Translational Research Centre, Centre for Primary Care, Division of Population Health, Health Services Research and Primary Care, University of Manchester, Manchester M13 9PL, UK

3. General Practitioner NHS Greater Glasgow and Clyde, Medical Directorate, NHS Education for Scotland, Glasgow G3 8BW

4. St Gabriels Medical Centre, 4, Bishops Road, Prestwich, Manchester M25 0HT, UK

5. Central and South Manchester Specialty Training Programme for General Practice, Health Education England North West (HEENWE) Education and Research Centre, Wythenshawe Hospital, Manchester M23 9LT, UK

6. Division of Dentistry, School of Medical Sciences, University of Manchester, Manchester M13 9PL, UK

7. Medical Directorate, NHS Education for Scotland, 2 Central Quay, Glasgow G3 8BW, UK.

8. Institute of Health and Wellbeing, University of Glasgow, Glasgow G12 0XH, UK

Correspondence to jill.stocks@manchester.ac.uk

Word count: 3333

For peer review only - http://bmjopen.bmj.com/site/about/guidelines.xhtml

Abstract

Objectives: To design and pilot a survey to be used at the population level to estimate the frequency of patient-perceived potentially-harmful preventable-problems occurring in UK primary care. To explore the nature of the problems, patient-suggested strategies for prevention and opinions of clinicians and the public regarding the potential for harm

Design: a survey was co-designed by three members of the public and one researcher and piloted through public and patient involvement and engagement networks

Setting: self-selected sample of the UK population

Participants: 977 members of the public accessed the online survey during October and November 2015

Primary outcome measures: respondent feedback about the ease of completion of the survey, quality of responses in terms of review by clinicians and members of the public, preliminary estimates of the frequency and nature of patient-perceived potentially-harmful problems occurring in the last 12 months

Results: 638 members of the public completed the survey (65% response rate) and few respondents reported any difficulty in understanding or completing the survey. 132 (21%) respondents reported experiencing a potentially-harmful preventable-problem during the past 12 months and 108 (82%) of these provided adequate information for at least one clinician to estimate the likelihood the respondent described a potentially-harmful problem. Respondents were older than the UK generally, more likely to work or volunteer in the healthcare sector and tended to use primary care more frequently but their trust and confidence in their GP was similar to that of the UK population as measured by the annual population level GP patient survey.

Conclusions: the survey was acceptable to patients and mostly provided data of sufficient quality for review by clinicians and members of the public. It is now ready to use at a population level to estimate the frequency and nature of potentially-harmful preventable-problems in primary care from a patient's perspective.

Strengths and limitations of this study

- We have designed and tested a survey to measure the frequency and nature of potentially-harmful preventable-problems in primary care from the patient's perspective
- The survey was co-designed by three members of the public and piloted through extensive public and patient involvement
- The patient-described scenarios were reviewed by primary care clinicians
- The study respondents were self-selected through public and patient involvement and engagement groups
- The survey is ready to be administered to a representative sample of the general population

For peer review only - http://bmjopen.bmj.com/site/about/guidelines.xhtml

Background

Patients are thought to take a different view of patient safety to healthcare professionals. (1) They tend to view safety in terms of the overall balance of benefit and harm over time whereas healthcare professionals often see high quality healthcare occasionally punctuated by safety incidents and adverse events.(2) Furthermore patients may different opinions about how to improve patient safety.(3, 4) or different priorities to clinicians, for example identifying psychological and emotional harm rather than technical errors.(5) Involving patients in identifying errors and reducing harm occurs in secondary care (6) but patient reported outcomes can show poor concordance between patients and clinicians, for example, in reporting adverse symptom events in the context of drug safety.(7) Nonetheless patients are thought to be capable of reporting medical errors accurately. (6, 8) Involving patients is advocated as a way to improve safety (9) and this approach would be facilitated through patients and professionals understanding each other's expectations and priorities.

Studies that quantify patient safety problems in primary care are uncommon and incidence estimates range from less than 1 to 24 per 100 consultations or record review.(10-12) The National Reporting and Learning System (NRLS) in England and Wales records just 1% of reports as originating in primary care (13) that likely reflects under-reporting.(14, 15) Still fewer studies have quantified patient safety problems in primary care from the patient's perspective. (16) A 2013 European survey reported that 43% of UK respondents felt that it was "likely" that patients could be harmed by non-hospital healthcare, an increase from 37% in 2009.(17) In Norway the patient-reported lifetime probability of ever experiencing an adverse event was 10%, of which around two thirds of respondents attributed the cause of their event as their general practitioner (GP).(4) In Spain it was estimated that around 7% of patients experienced an adverse event during a 1 year period. (18) A USA practice-based website observed an incidence rate of safety events of 1.4% over 2 years.(19) Data from the UK is sparse; this may be partly due to the lack of a valid and reliable instrument to make a comprehensive measurement of safety in primary care.(20) The PREOS-PC should help to address this knowledge gap.(21, 22)

We aimed to design and pilot a survey to be used at the population level to estimate the frequency of patient-perceived potentially-harmful preventable-problems occurring in UK primary care. We relied on public and patient involvement (PPI) from the outset in order to ensure that our survey was easily understood by the public. We also aimed to explore the nature of the problems, patient-suggested strategies for prevention and differences in opinion between primary care clinicians and the public regarding the potential for harm in the patient-described scenarios. The study was conceived, designed and implemented by a team of three members of the public and one researcher. Primary care professionals provided their opinions after collection of the data. The specific aims of the study were to:

1. co-design and pilot a survey asking about problems occurring in primary care that caused, or had the potential to cause, preventable harm as perceived by patients using PPI.
2. describe the type of patient-perceived problems reported, describe the demographics of the patients reporting a problem, the primary care service involved, with whom in the primary care

For peer review only - http://bmjopen.bmj.com/site/about/guidelines.xhtml

service the problem was discussed (if it was) and patient suggestions as to how it might have been prevented.

3. compare the opinions of the reporting patient, members of the public and clinicians as to the likelihood the scenario described was a potentially-harmful preventable-problem.

Methods

Designing and piloting of the survey

Our aim was to design a survey asking about problems occurring in primary care that caused, or had the potential to cause, preventable harm as perceived by patients that was easily understood and free from jargon. Currently there is no well-established terminology for asking such a question.(8) The process began with a discussion between three members of the public (AD, JB, CG) who were members of the public and patient involvement (PPI) group of the Greater Manchester Primary Care Patient Safety Translational Research Centre Research User Group (23)(GMPSTRC RUG, see Appendix 1 for more details) and one academic researcher (SJS). Questions used in previous surveys addressing a similar question (4, 17-19) were shared among the project team and used to generate several candidate questions. These questions were then discussed privately among the project team's friends and family and within the project team (SJS, AD, JB, CG). The discussion was facilitated by making the candidate questions available online. After two iterations of this process the survey (online Appendix 1, Box 1) was piloted online through newsletters or group mailings of several PPI and public engagement networks during November and December 2015. These networks were the associate GMPSTRC RUG, the Public Programmes team at Central Manchester Foundation Trust, the Citizen Scientist project, the Primary Care Research in Manchester Engagement Resource, North West People in Research Forum and HelpBeatDiabetes volunteers. (Details of these groups and networks is in online Appendix 1.)

The first question (Q1 Box 1) was taken from the English GP patient survey in order to compare the overall level of confidence and trust in their GP among the survey respondents with that across England.(24) The second question (Q2 Box 1) is the main screening question, those responding negatively to Q2 (*i.e.* not experienced a preventable-problem) were directed to a more specific question with a list of commonly understood patient safety events (online Appendix 1). If this prompted recognition of experiencing a potentially-harmful preventable-problem they were returned to Q4 (Box1). The rationale behind this approach was that the screening question (Q2 Box 1) should be non-leading and encourage the respondents to describe their preventable-problems through the subsequent questions without the suggestion that inevitably occurs following a list of possible potentially-harmful preventable-problems. However if the respondent did not believe that they had experienced a potentially-harmful preventable-problem then the prompt question (Q10, Box 1) would ensure that this was the case and also test the sensitivity of Q2 (Box 1). The option to answer on behalf of a friend or relative was offered to those who had not a personal experience to report. This was to ensure sufficient responses to adequately test the questionnaire but also to discourage respondents from answering with another person's experience as their own. Respondents were also asked whether they worked or volunteered in the healthcare profession and to comment on the ease of completion of the questionnaire.

Coding of reported events

For peer review only - http://bmjopen.bmj.com/site/about/guidelines.xhtml

Type of problem

The nature of the problem in each described scenario was coded at face value, *i.e.* as the patient described without further interpretation, by one author (SJS) and checked by a second author (JA for dental scenarios, PB for all other scenarios). A bottom-up (inductive) approach was used to identify similar topics which were coded then cross-matched to an existing taxonomy for errors in general practice (25, 26) (Table A, online Appendix 2). All the new codes matched the existing taxonomy within the higher two levels and the medication-related scenarios were coded to a finer level (Table B, online Appendix 2).

Likelihood the scenario described a potentially-harmful preventable-problem

Five GPs, one dentist and seven members of the public estimated the likelihood that, in their opinion, each patient-described scenario was a potentially-harmful preventable-problem. Brief biographies of the coders are provided in online Appendix 1. Some examples of the information provided to the coders are shown in online Appendix 2 and consisted of the responses to Q5 to Q9 (Box 1). They were not given any demographic information or the patient's estimate of the impact on their health (Q4, Box 1). Coders were asked to score each scenario from very likely (5) to definitely not (1) in response to the question *"How likely is it that the patient was correct in thinking that their health might be worsened, or actually was made worse, because of the preventable problem described below?"* Coders could also respond "insufficient information", "Don't know" and give free text feedback (Table C, Appendix 2). The clinician scores were used to categorise the scenarios in to groups with higher or lower estimated likelihoods that they were a potentially-harmful preventable-problem as below.

- Higher threshold - Median score of 5 ("very likely or certain") or 4 ("probably") or at least one score of 5 ("very likely or certain")
- Lower threshold - Median score of 3 ("possibly") or at least one score of 4 ("probably" or higher)
- All other scenarios – Median score below 3 ("possibly") and zero scores above 3 ("possibly")

Statistical analysis

Simple cross tabulations were used to describe the data and a binary logistic regression model was used to explore whether particular types of patient were more likely to perceive a potentially-harmful preventable-problems *e.g.* by demographics or their opinions. Comparisons between demographics and outcomes for the respondents and the UK (or England) population were made using a χ^2 test. All analyses were done using Stata 14.

Public and Patient Involvement (PPI)

PPI was central to this co-design study and was provided through the GMPSTRC RUG (23) and other PPI networks (online Appendix 1). The study was conceived, designed, implemented and analysed by a team of three members of the public (AD, CG, JB) and one researcher (SJS). At the outset the researcher presented the existing literature on this topic to the PPI members of the research team who then co-designed the first draft of the survey which was tested through the PPI members' personal contacts. The piloting of the survey was through existing PPI networks as listed in online Appendix 1. The scoring of the questions as to the likelihood they described a potentially-harmful preventable-problem was undertaken by 7 members of the public, 2 of whom had no previous

For peer review only - http://bmjopen.bmj.com/site/about/guidelines.xhtml

experience in PPI (as well as 5 GPs and 1 dentist as described in online Appendix 1). These findings will be disseminated to all the PPI groups that contributed to the pilot study and the authors will forward these results to their personal contacts who contributed to the questionnaire design.

Ethical approval was granted by the University of Manchester Ethics Committee 2 (Approval 15372).

Results

In total 977 members of the public accessed the online pilot survey and 638 (65%) completed the survey during October and November 2015. Flow charts of the participants through the survey are shown in Figures A&B, online Appendix 1. In total 223/638 (35%) of respondents reported ever experiencing a potentially-harmful preventable-problem in primary care of which 132 occurred within the past 12 months (21%, Fig A, online Appendix 1) and 62 (10%) of these came required prompting through question 10 (Box 1 and Fig A, online Appendix 1). A further 18 potentially-harmful preventable-problems involving friends or relatives where the respondent was present and occurred in the last 12 months were reported 13/418 (3%, Fig B, online Appendix 1). The majority of respondents (592, 93%) had confidence and trust in the GP seen at their last appointment similar to the 2016 England proportion of 92% (Q1, Box1 &Table 1). Demographic information was not provided by 83 (13%) respondents, possibly due to lack of clarity about the end of the survey since they completed all other questions. Respondents were older than the UK generally, more likely to work or volunteer in the healthcare sector and tended to use primary care more frequently (Table 1).

The majority of respondents were recruited through the HelpBeatDiabetes group (533, 84%, online Appendix 1). Over 250 respondents provided free text feedback on the survey, 200 comments reported that the questionnaire was easy to complete and understand and just one comment described the survey as complex. Most of the remaining comments expressed the desire to be able to provide more information, *e.g.* more than one event or report for a relative or as a carer (reporting on behalf of another person was excluded for events occurring more than 12 months ago) and 13 comments actually provided this unrequested information. A few respondents found it difficult to find a suitable option to describe their pattern of use of primary care or their role as a worker or volunteer in healthcare.

The high completion rate and positive free text feedback suggested that respondents found the questionnaire easy to complete. Furthermore nobody used the “Do not understand the question” option as their response to Q2 Box1. There was a high response from healthcare professionals or volunteers (30% of respondents compared to approximately 3% of the UK adult population, Table 1) but they were no more likely to report a preventable problem than non-healthcare workers/volunteers (35%, $P\chi^2$=0.28). However the scenarios described by healthcare professionals or volunteers were significantly more likely to be categorised as a potentially-harmful preventable-problem following to clinician review using both the higher (9% vs 16%, $P\chi^2$=0.01) and lower threshold (2% vs 6%, $P\chi^2$=0.004).

Likelihood the scenario described a potentially-harmful preventable-problem

Generally the members of the public assigned a higher probability to the likelihood that the patient-described scenario was a potentially-harmful preventable-problem compared with GPs (Fig 1, Table

For peer review only - http://bmjopen.bmj.com/site/about/guidelines.xhtml

2). In 89/108 (82%) scenarios the median score for the PPI researchers was higher than for the clinicians and for 38 (35%) scenarios the PPI median score was 2 or more points higher in a 5 point scale. Following clinician review 3% of the patient-reported scenarios occurring in the last 12 months were categorised as "probably" a potentially-unsafe preventable-problem and 11% as "possibly" (Table 2). Examples of the patient-reported scenarios with higher clinician rankings and those with greatest disagreement between members of the public and clinicians are shown in online Appendix 2.

The nature of the potentially-harmful preventable-problems

The types of patient-reported scenarios and their categorisation following clinician review are shown in Figure 2. Medication-related problems were most frequently reported type of problem and also were ranked as more likely to be a potentially-harmful problem by clinicians. Information about the patient's response to the potentially-harmful preventable-problem and the primary care service involved is provided in Table 4. The majority of potentially-harmful preventable-problems in the past 12 months occurred in general practice (73%, Table 4) and pharmacy (5%, Table 4). Around half the respondents had not discussed their problem with anybody working in primary care (51%, Table 4). The most common reasons for not discussing the problem were being unable to find a primary care professional with whom to discuss the problem (31%, Table 4) or they did not feel comfortable with discussing their concerns (24%, Table 4) The patient suggestions for ways to prevent the problem from happening are summarised in Table 5. The most frequently occurring suggestions were that clinicians should involve the patient more fully in the healthcare process (*i.e.* listen to the patient and trust their judgement more) and be up to date with, and apply, the most recent information about the patient's condition (*i.e.* take in to account all of the patient's information - their medical history and results and letters).

Discussion

We have designed and tested a survey to measure the frequency of occurrence of potentially-harmful preventable-problems in primary care and found it to be well understood by patients. The survey is acceptable to patients based on the high completion rate and positive feedback. Furthermore none of the respondents indicated that they did not understand the screening question (Q2, Box1). The open-ended questions (Q6 to Q9, Box 1) led to patient-described scenarios that mapped well to an existing taxonomy designed and used by clinicians and researchers.(25, 26) This implies agreement between clinicians, researchers and patients in identifying the characteristics of a potentially-harmful problem. Furthermore, using a non-leading screening question (Q2, Box 1) to ensure that any problems unique to the patient perspective were identified did not find additional types of problem. Members of the public, however, ranked the scenarios as being more likely to describe a potentially-harmful preventable-problem compared with clinicians (Fig 1).

Strengths and weaknesses of the study

We believe that our survey captures the true patient perspective due to the involvement of members of the public as research partners through data acquisition to analysis and reporting in a co-designed study. By the use of a simple non-leading screening question we encouraged respondents to express their own perspective on what constituted a potentially-harmful

For peer review only - http://bmjopen.bmj.com/site/about/guidelines.xhtml

preventable-problem rather than directing them towards existing definitions. To ensure that we did not miss any problems we followed up with a prompt that encouraged respondents to think in terms of the traditional view of patient safety problems. Furthermore our survey goes further than describing and counting the frequency of occurrence of potentially-harmful preventable-problems and provides information about how patients dealt with the problem and how it could have been prevented that offers insight in to ways to reduce the frequency of their occurrence. The absence of a link between practices and the patients allows for responses that might not occur if this survey were administered through the individual's practice. The main weakness of the study is the self-selection of the respondents who were older and tended to use primary care more frequently. More frequent users of primary care were more likely to report a problem but age was not associated with the likelihood of reporting a problem. Our bench marking question (Q1, Box1) showed that the respondents were similar to the English GP patient survey(24) in terms of their level of trust and confidence in their GP and not a group with a more negative attitude towards primary care as might have happened given the nature of the survey.

Strengths and weaknesses in relation to other studies

Our finding that 35% of respondents perceived that they had experienced a potentially-harmful problem in in their lifetime is consistent with a European survey (43% of UK respondents felt that it was "likely" that patients could be harmed by non-hospital healthcare).(17) This study offers some insight in to the type of concerns that might underlie this apparent lack of confidence in primary care. A face to face interview in family practice waiting rooms in the USA reported that 16% of respondents believed a physician had made a mistake in their care.(27) The types of problem and patient responses to the problem are similar to those that have been described qualitatively (1, 22) but we have taken this a step further by quantifying the frequency of occurrence and other descriptors of the problem from the patient's perspective. In this small study we did not find that patients were particularly likely to attribute blame to individual members of staff as has been observed previously (3, 4), perhaps partly due to the high proportion of respondents working or volunteering in healthcare.

Unanswered questions and future research

Our finding that 21% of respondents perceived that they had experienced a potentially-harmful problem in the last 12 months, and the corresponding proportion following clinician review of 3% (higher threshold) to 11% (lower threshold) may well reflect the self-selected nature of the study population and needs to be validated in a large population level survey. We anticipate that a population level survey would be fruitful since this approach yielded a number of patient-described scenarios that were amenable to further analysis including coding by clinicians. The high response to this pilot survey by healthcare professionals coupled with the likelihood that they provided better information, given the higher ranking given by clinicians to scenarios originating from healthcare professionals, points towards an opportunity. Healthcare professionals are an educated and accessible group who could provide a valuable resource for learning about preventable-problems in primary care. This survey could be used to ask NHS staff anonymously about their *personal experiences, as a patient,* of potentially-harmful preventable-problems in primary care. Of course complete anonymity for responders would need to be guaranteed and any identifying aspects in their reported scenarios would have to be removed. It would be very different to whistle-blowing,

For peer review only - http://bmjopen.bmj.com/site/about/guidelines.xhtml

respondents would feed back on the care they received personally rather make observations on their own colleagues practice. The aim would be to anonymously describe and monitor problems over time through individuals with the expectations of a patient who also had an understanding of the healthcare system.

Acknowledgements: The authors would like to express their thanks and appreciation for the work done by the Mary Aldred, Gitanjali Holt, Manoj Mistry, Carole Bennett and Lindsey Brown in coding the patient-described scenarios.

This work was supported by the NIHR grant number gmpstrc-2012-1

Funding: This study was funded by the National Institute for Health Research (http://www.nihr.ac.uk) through the Greater Manchester Primary Care Patient Safety Translational Research Centre (NIHR GM PSTRC), grant number gmpstrc-2012-1. The views expressed are those of the author(s) and not necessarily those of the NHS, the NIHR or the Department of Health. The funders had no role in study design, data collection and analysis, decision to publish, or preparation of the manuscript.

Competing interests: All authors have completed the ICMJE uniform disclosure form at www.icmje.org/coi_disclosure.pdf (available on request from the corresponding author) and declare no support from any organisation for the submitted work; no financial relationships with any organisations that might have an interest in the submitted work in the previous three years, no other relationships or activities that could appear to have influenced the submitted work.

Copyright statement: the Corresponding Author has the right to grant on behalf of all authors and does grant on behalf of all authors, an exclusive licence on a worldwide basis to the BMJ Publishing Group Ltd to permit this article (if accepted) to be published in BMJ editions and any other BMJPGL products and sublicences such use and exploit all subsidiary rights, as set out in our licence.

Contributors: SJS, AD, JB and CG conceived and designed the study. SJS, AD, JB, CG, AE, PB, JA, DT, SL, AD, RD and NM analysed the data. SJS wrote the manuscript, and is guarantor. AD, JB, CG, AE, PB, JA, DT, SL, AD, RD, NM and SC edited the manuscript.

Data sharing: Raw data (coded only) is available from jill.stocks@manchester.ac.uk
Ethics approval: University of Manchester Ethics Committee 2 (Approval 15372)

Figure legends

Figure 1. Median estimates as to the likelihood that the patient describes a potentially-harmful preventable-problem occurring in the last 12 months by six clinicians and seven members of the public

Footnote to Figure 2: See Appendix 2 for details of coding; A coded to 2 levels, B coded to 1 level, C medication problems coded to 3 levels

Fig 2. Numbers of patient-perceived problems occurring in the last 12 months categorised according to the patient's description with clinician ranking as to the likelihood it is a potentially-harmful preventable problem (Table 2).

For peer review only

<u>Box 1. Brief summary of questionnaire – see online appendix 1 for full version of survey.</u>

Q1. Did you have confidence and trust in the GP you saw or spoke to at your last appointment? (benchmarking question)
Q2. When using primary care have you ever felt concerned that your health might be worsened, or actually was made worse, because of a mistake or a problem that could have been prevented?
If yes to Q2<br>Q3. How long ago did the mistake or preventable problem happen?
Q4. How did this affect your health?
Q5. Which primary care service were you using when the mistake or preventable problem occurred?
Q6. Briefly describe the mistake or problem and how it happened
Q7. Could the mistake or problem have been avoided? If so how?
Q8. Were you able to talk about the mistake or problem with anybody working in the primary care service? If not –why not?
Q9. If you discussed the mistake or problem with somebody working in primary care please describe their job or role
Q10. In the list below are some examples of **preventable** problems[1] that might happen when using primary care. Has ***<u>anything similar</u>*** happened to you ***<u>in the last 12 months</u>***? *If yes go to Q4*<br>[1]See online appendix 1 for list of preventable problems

For peer review only

Table 1. Characteristics of survey respondents

Variable	All respondents n=638	Ever had problem n=223	Had problem in last 12 months n=132	UK population comparator
GP satisfaction	missing=0	missing=0	missing=0	GP patient survey(24)
Yes definitely	384 (60%)	81 (36%)	55 (42%)	64%
Yes, to some extent	208 (33%)	110 (49%)	52 (39%)	28%
No, not at all	39 (6%)	27 (12%)	21 (16%)	4%
Don't know / can't say	7 (1%)	5 (2%)	4 (3%)	3%
Worked or volunteered in healthcare	missing=92	missing=40	missing=19	NHS workforce[1]
Yes	166 (30%)	64 (35%)	41 (36%)	3%
Gender	missing=87	missing=38	missing=16	ONS mid-2015 estimates[2]
Female	268 (49%)	106 (57%)	63 (54%)	51%
Age	missing=85	missing=37	missing=15	ONS mid-2015 estimates[2]
16 to 34 years	42 (8%)	22 (12%)	11 (9%)	31%
35 to 54 years	143 (26%)	54 (29%)	34 (29%)	34%
55 to 64 years	162 (29%)	59 (32%)	31 (27%)	14%
65 to 74 years	170 (31%)	44 (24%)	32 (27%)	12%
Over 75 years	36 (7%)	7 (4%)	9 (8%)	9%
Last primary care contact	missing=88	missing=39	missing=14	GP patient survey(24)
Within last week	169 (31%)	65 (35%)	48 (41%)	84% within last 12 months
Within last month	248 (45%)	79 (43%)	47 (40%)	
Within last 12 months	121 (22%)	34 (18%)	20 (17%)	
Over 12 months ago	12 (2%)	6 (3%)	3 (3%)	15%
Usual primary care usage	missing=88	missing=40	missing=17	
At least once a month	181 (33%)	73 (40%)	52 (45%)	-
At least once per 6 months	285 (52%)	79 (43%)	45 (39%)	-
Once per 12 months or less	84 (15%)	31 (17%)	18 (16%)	-

[1]http://content.digital.nhs.uk/searchcatalogue?productid=24139&topics=1%2fWorkforce%2fStaff+numbers&sort=Relevance&size=10&page=1#top

[2]https://www.ons.gov.uk/peoplepopulationandcommunity/populationandmigration/populationestimates/bulletins/annualmidyearpopulationestimates/latest

For peer review only

Table 2. Categorisation of patient-perceived potentially-harmful preventable problems occurring in the last 12 months following review by clinicians and members of the public

Group label	Threshold criteria	GP scores n=132	PPI scores n=132
1. Higher threshold	Median score of "very likely or certain" or "probably" or at least one score of "very likely or certain"	18 (14%)	87 (66%)
2. Lower threshold	Median score of "possibly" or at least one score of "probably" or higher	71 (54%)	104(79%)
3. Any possibility	At least one score of "unlikely" or higher	106 (80%)	109 (83%)
4. No problem	All scores "definitely not" or not-coded	1 (1%)	0
5. Not-coded	Insufficient information for coding by all coders	25 (19%)	23 (17%)

For peer review only

Table 3. Prevalence of respondents reporting a potentially-harmful preventable problem within the last 12 months and unadjusted and adjusted odds ratios estimated by logistic regression

Respondent characteristics n=638	Frequency – all reported n=132	Unadjusted OR–all reports	Adjusted[1] OR- all reports	Adjusted[1] OR - after GP review (lower threshold, Table 2)
**Gender (87 missing)**				
male	53/283 (19%)	1 (ref)	1 (ref)	1 (ref)
female	63/268 (24%)	1.3 (0.9 to 2.0)	1.4 (0.9 to 2.2)	1.3 (0.7 to 2.3)
**Age (85 missing)**				
16 to 34 years	11/42 (26%)	1 (ref)	1 (ref)	1 (ref)
35 to 54 years	34/143 (24%)	0.9 (0.4 to 1.9)	0.8 (0.3 to 1.8)	0.8 (0.3 to 2.1)
55 to 64 years	31/162 (19%)	0.7 (0.3 to 1.5)	0.7 (0.3 to 1.5)	0.6 (0.2 to 1.7)
65 to 74 years	32/170 (19%)	0.7 (0.3 to 1.4)	0.6 (0.3 to 1.4)	0.4 (0.2 to 1.2)
Over 75 years	9/36 (25%)	0.9 (0.3 to 2.6)	1.1 (0.4 to 3.2)	0.9 (0.2 to 3.2)
**Last primary care contact (88 missing)**				
Within last week	48/169 (28%)	1 (ref)	1 (ref)	1 (ref)
Within last month	47/248 (19%)	0.6 (0.4 to 0.9)	0.7 (0.4 to 1.1)	0.6 (0.3 to 1.0)
Within last 12 months	20/121 (17%)	0.5 (0.3 to 0.9)	0.6 (0.3 to 1.2)	0.5 (0.2 to 1.3)
Over 12 months ago	3/12 (25%)	0.8 (0.2 to 4.0)	0.9 (0.2 to 4.2)	0.4 (0.0 to 3.9)
**Usual primary care usage (88 missing)**				
At least once a month	52/181 (29%)	1 (ref)	1 (ref)	1 (ref)
At least once per 6 months	45/285 (16%)	0.5 (0.3 to 0.7)	0.6 (0.3 to 0.9)	0.5 (0.3 to 0.9)
Once per 12 months or less	18/84 (21%)	0.7 (0.4 to 1.2)	0.8 (0.4 to 1.6)	0.7 (0.3 to 1.8)
**Works or volunteers in healthcare (92 missing)**				
No	72/380 (19%)	1 (ref)	1 (ref)	1 (ref)
Yes	41/166 (25%)	1.4 (0.9 to 2.2)	1.3 (0.8 to 2.1)	1.5 (0.9 to 2.7)

[1]adjusted for gender, age, last primary care contact, usual primary care usage, works or volunteers in healthcare

For peer review only

Table 4. The patient's response to their perceived potentially-harmful preventable-problem and the primary care service involved for problems occurring in the last 12 months

Primary care service	All reported problems	Clinician ranked "possibly or higher" (Lower threshold)
All services	132	71
GP surgery	97 (73%)	61 (86%)
Out of hours care/A&E/ambulance	4 (3%)	1 (1%)
Walk in clinic	2 (2%)	0
Dental surgery	4 (3%)	1 (1%)
Pharmacy	7 (5%)	6 (8%)
Community or district nursing	4 (3%)	0
Opticians	2 (2%)	1 (1%)
Mental health services	1 (1%)	0
missing	11 (8%)	1 (1%)
**Did you discuss the problem with primary care staff?**		
All respondents	132	71
Yes – discussed with primary care staff	56 (42%)	42 (59%)
No – did not discuss with primary care staff	67 (51%)	29 (41%)
missing	9 (7%)	0
**Reason not discussed with primary care staff**		
All not discussing problem	67	29
Did not feel comfortable to discuss the problem	16 (24%)	8 (28%)
Could not find anybody with whom to discuss the problem	21 (31%)	10 (34%)
Unconcerned about the problem	7 (10%)	5 (17%)
Did not notice the problem at the time (or too ill)	11 (16%)	4 (14%)
Other	5 (7%)	2 (7%)
missing	7 (10%)	0
**Profession of discussant**		
All discussing problem	56	42
GP	28 (50%)	19 (45%)
Practice manager	5 (9%)	5 (21%)
Receptionist	2 (4%)	1 (2%)
Practice nurse	6 (11%)	5 (12%)
Pharmacist or dispenser	7 (13%)	7 (17%)
Dentist	2 (4%)	1 (2%)
Dietician	1 (2%)	1 (2%)
Missing	5 (9%)	3 (7%)
**Role of discussant in patient's care**		
Member of staff directly involved	23 (41%)	16 (38%)
Another member of staff at same institution	25 (45%)	20 (48%)
Above unclear	8 (14%)	6 (14%)

For peer review only - http://bmjopen.bmj.com/site/about/guidelines.xhtml

Table 5. Patient suggestions as to how the potentially-harmful preventable problem might have been prevented

**How could it be prevented?**	All reported problems n=132	Clinician ranked "possibly or higher" (Lower threshold) n=71
**1. More resources - all**	**14 (11%)**	**3 (4%)**
1.1 Quicker access to primary care	7 (5%)	2 (3%)
1.2 More thorough and quicker investigations	2 (2%)	1 (1%)
1.3 Fewer demands on primary care – more staff or fewer patients	1 (1%)	0
1.4 More time with clinicians for treatment and diagnosis	2 (2%)	0
1.9 Provision of resources to manage long term conditions	1 (1%)	0
1.10 Provision of patient travel service for routine appointments	1 (1%)	0
**2. Improved communication and involvement of patients**	**26 (20%)**	**18 (25%)**
1.1 Listen to the patient and trust their judgement more	21 (16%)	15 (21%)
1.2 Tell patients about their diagnosis, test results, changes in medication or loss of results	3 (2%)	1 (1%)
1.3 Improve communication between staff (within or outside primary care)	2 (2%)	2 (3%)
**3. Better organisation and administration**	**17 (13%)**	**10 (14%)**
3.1 Follow up referrals and appointments to ensure they happen, be consistent in sending routine reminders	10 (8%)	3 (4%)
3.2 Log in or process results as soon as received to avoid loss	1 (1%)	1 (1%)
3.3 Keep the notes up to date, well-organised, safe and ensure information is transcribed accurately	5 (4%)	5 (7%)
3.4 Keep a record of the location of equipment	1 (1%)	1 (1%)
**4. Improved prescribing systems**	**18 (14%)**	**17 (24%)**
4.1 More when checks on prescribing and dispensing	8 (6%)	8 (11%)
4.2 Check repeat prescriptions carefully, especially for transcribing errors	8 (6%)	7 (10%)
4.3 Use medication reviews and IT clinical decision support systems	2 (2%)	2 (3%)
**5. Better clinical practice**	**19 (14%)**	**10 (14%)**
5.1 Take in to account all the patient's information - their medical history and results and letters	13 (10%)	7 (10%)
5.2 Address the patient's problem in some way – patients can feel their problem is being ignored	5 (4%)	2 (3%)
5.3 Act on advice from other clinicians and test results	1 (1%)	1 (1%)
**6. Staff training**	**11 (8%)**	**7 (10%)**
6.1 More informed and better trained staff	11 (8%)	7 (10%)
**Other responses**	**27 (20%)**	**6 (8%)**
• Don't know/missing	21 (16%)	3 (4%)
• Problem was due to an individual member of staff	2 (2%)	1 (1%)
• Prescribe right, better, different, more, less medicine	1 (1%)	0
• Better organisation	1 (1%)	0
• Laboratory procedures were the problem	2 (2%)	2 (3%)

For peer review only - http://bmjopen.bmj.com/site/about/guidelines.xhtml

References

1. Rhodes P, Campbell S, Sanders C. Trust, temporality and systems: how do patients understand patient safety in primary care? A qualitative study. Health Expectations. 2016;19(2):253-63.

2. Vincent C, Amalberti R. Safer Healthcare: Strategies for the Real World. Springer, 2016. http://www.springer.com/gb/book/9783319255576 Accessed 04/04/17

3. Blendon RJ, DesRoches CM, Brodie M, Benson JM, Rosen AB, Schneider E, et al. Views of practicing physicians and the public on medical errors. N Engl J Med. 2002;347(24):1933-40.

4. Hotvedt R, Forde OH. Doctors are to blame for perceived medical adverse events. A cross sectional population study. The Tromso Study. BMC health services research. 2013;13:46.

5. Kuzel AJ, Woolf SH, Gilchrist VJ, Engel JD, LaVeist TA, Vincent C, et al. Patient reports of preventable problems and harms in primary health care. Ann Fam Med. 2004;2(4):333-40.

6. The Health Foundation. Evidence scan: Involving patients in improving safety. January 2013. http://www.health.org.uk/sites/health/files/InvolvingPatientsInImprovingSafety.pdf Accessed 04/04/17

7. Basch E. The missing voice of patients in drug-safety reporting. N Engl J Med. 2010;362(10):865-9.

8. King A, Daniels J, Lim J, Cochrane DD, Taylor A, Ansermino JM. Time to listen: a review of methods to solicit patient reports of adverse events. Qual Saf Health Care. 2010;19(2):148-57.

9. Longtin Y, Sax H, Leape LL, Sheridan SE, Donaldson L, Pittet D. Patient participation: current knowledge and applicability to patient safety. Mayo Clinic proceedings. 2010;85(1):53-62.

10. Sandars J, Esmail A. The frequency and nature of medical error in primary care: understanding the diversity across studies. Family practice. 2003;20(3):231-6.

11. Panesar SS, deSilva D, Carson-Stevens A, Cresswell KM, Salvilla SA, Slight SP, et al. How safe is primary care? A systematic review. BMJ Quality & Safety. 2016;25(7):544-53.

12. Michel P, Brami J, Chaneliere M, Kret M, Mosnier A, Dupie I, et al. Patient safety incidents are common in primary care: A national prospective active incident reporting survey. PLoS One. 2017;12(2):e0165455.

13. NHS Improvement. National quarterly data on patient safety incident reports. September 2016. https://improvement.nhs.uk/resources/national-quarterly-data-patient-safety-incident-reports-september-2016/ Accessed 04/04/17

14. Hutchinson A, Young TA, Cooper KL, McIntosh A, Karnon JD, Scobie S, et al. Trends in healthcare incident reporting and relationship to safety and quality data in acute hospitals: results from the National Reporting and Learning System. Qual Saf Health Care. 2009;18(1):5-10.

15. NHS England. General practice patient safety reporting form launched. 26th February 2015. https://www.england.nhs.uk/2015/02/gp-patient-safety-reporting/ Accessed 04/04/17

For peer review only - http://bmjopen.bmj.com/site/about/guidelines.xhtml

16. The Health Foundation. Evidence Scan: Improving safety in primary care. November 2011. http://www.health.org.uk/sites/health/files/ImprovingSafetyInPrimaryCare.pdf Accessed 04/04/17

17. European Commission. Special Eurobarometer 411 Patient Safety and Quality of Care 2014. DOI:10.2772/33467 https://ec.europa.eu/health/sites/health/files/patient_safety/docs/ebs_411_sum_en.pdf Accessed 04/04/17

18. Mira JJ, Nebot C, Lorenzo S, Perez-Jover V. Patient report on information given, consultation time and safety in primary care. Qual Saf Health Care. 2010;19(5):e33.

19. Wasson JH, MacKenzie TA, Hall M. Patients use an internet technology to report when things go wrong. Qual Saf Health Care. 2007;16(3):213-5.

20. Ricci-Cabello I, Goncalves DC, Rojas-Garcia A, Valderas JM. Measuring experiences and outcomes of patient safety in primary care: a systematic review of available instruments. Family practice. 2015;32(1):106-19.

21. Ricci-Cabello I, Avery AJ, Reeves D, Kadam UT, Valderas JM. Measuring Patient Safety in Primary Care: The Development and Validation of the "Patient Reported Experiences and Outcomes of Safety in Primary Care" (PREOS-PC). Ann Fam Med. 2016;14(3):253-61.

22. Ricci-Cabello I, Saletti-Cuesta L, Slight SP, Valderas JM. Identifying patient-centred recommendations for improving patient safety in General Practices in England: a qualitative content analysis of free-text responses using the Patient Reported Experiences and Outcomes of Safety in Primary Care (PREOS-PC) questionnaire. Health Expectations. 2017.

23. Stocks SJ, Giles SJ, Cheraghi-Sohi S, Campbell SM. Application of a tool for the evaluation of public and patient involvement in research. BMJ Open. 2015;5(3).

24. NHS England. GP Patient Survey – National summary report. January 2016. http://gp-survey-production.s3.amazonaws.com/archive/2016/January/January+2016+National+Summary+Report.pdf Accessed 04/04/17

25. Dovey SM, Meyers DS, Phillips RL, Jr., Green LA, Fryer GE, Galliher JM, et al. A preliminary taxonomy of medical errors in family practice. Qual Saf Health Care. 2002;11(3):233-8.

26. Makeham MA, Dovey SM, County M, Kidd MR. An international taxonomy for errors in general practice: a pilot study. Med J Aust. 2002;177(2):68-72.

27. Kistler CE, Walter LC, Mitchell C, Sloane PD. Patient perceptions of mistakes in ambulatory care. Archives of Internal Medicine. 2010;170(16):1480-7.

For peer review only - http://bmjopen.bmj.com/site/about/guidelines.xhtml

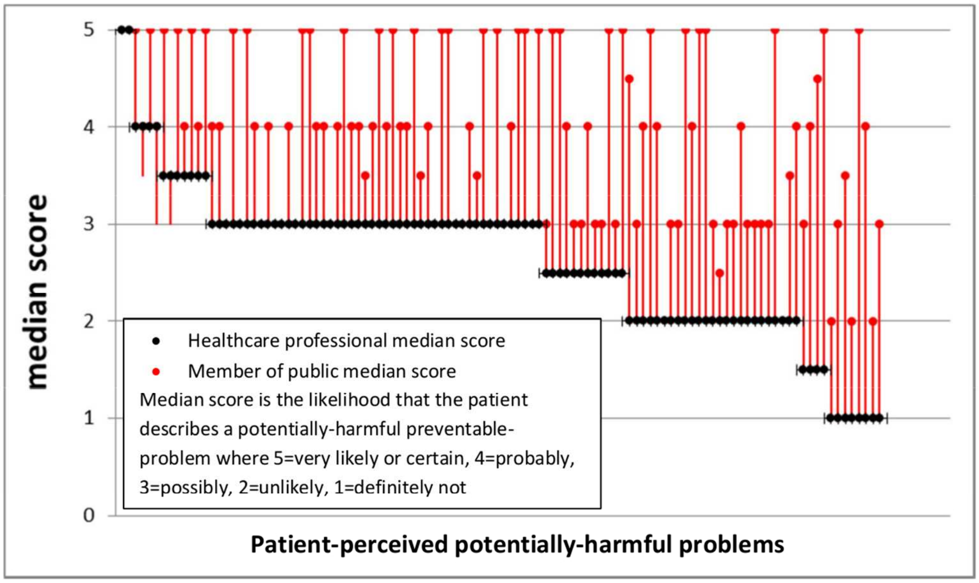

Figure 1. Median estimates as to the likelihood that the patient describes a potentially-harmful preventable-problem occurring in the last 12 months by six clinicians and seven members of the public

170x102mm (200 x 200 DPI)

For peer review only

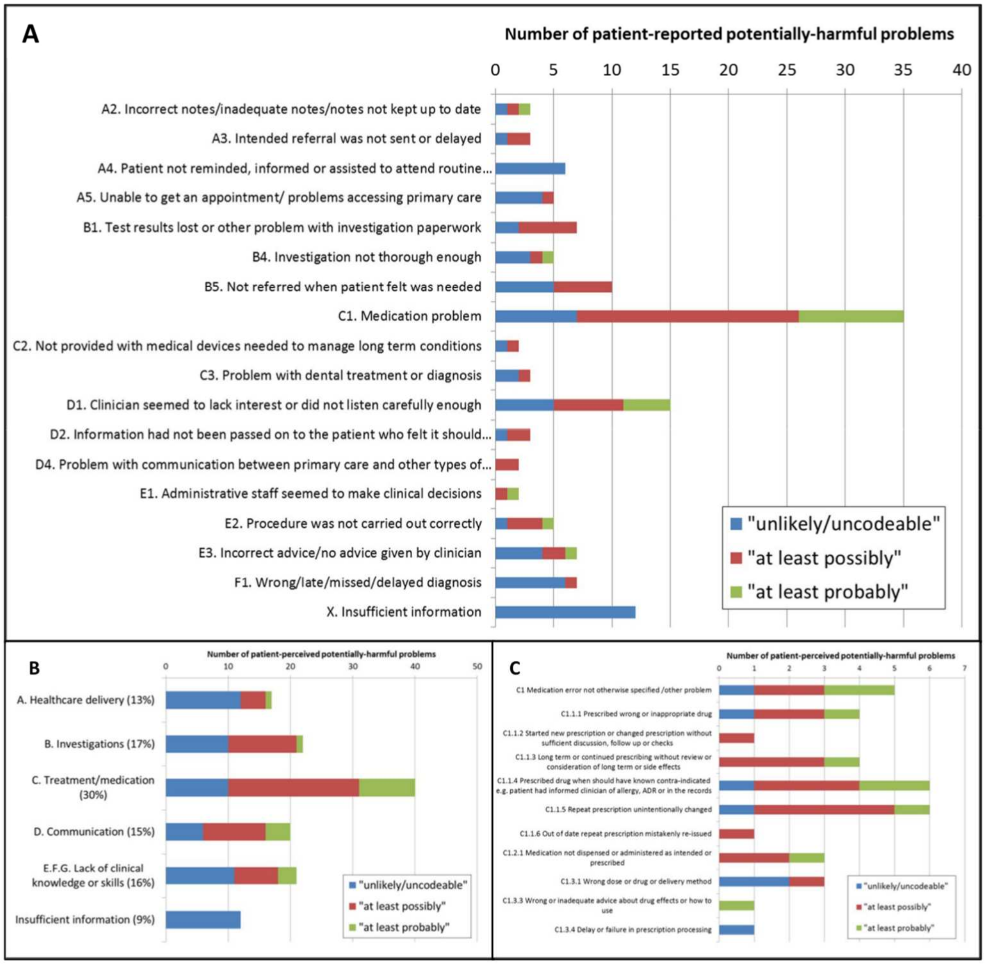

See Appendix 2 for details of coding; A coded to 2 levels, B coded to 1 level, C medication problems coded to 3 levels

Fig 2. Numbers of patient-perceived problems occurring in the last 12 months categorised according to the patient's description with clinician ranking as to the likelihood it is a potentially-harmful preventable problem (Table 2).

173x168mm (200 x 200 DPI)

Appendix 1. Details of public and clinician contributors and surveys

Table A. Demographics of clinicians and members of the public reviewing the patient-reported problems and estimating the likelihood the scenarios describes a potentially-harmful preventable-problem occurring in primary care

**Demographics of GP and dentist coders**	**frequency n=6**
**Gender**	
Female	3
Male	3
**Years working as a GP or dentist**	
Less than 15 years	1
15 to 25 years	2
Over 25 years	3
**Current position**	
Partner	4
Retired within last 12 months	2
**Demographics of the members of the public**	**frequency n=7**
**Gender**	
Female	6
Male	1
**Age**	
30 to 39 years	2
40 to 49 years	1
50 to 59 years	2
60 to 69 years	2
**Ethnicity**	
White British	5
British Indian	2
**Years of PPI experience**	
None	2
Less than 1 year	1
1 to 5 years	2
Over 5 years	2
**Further background information**	
PPI reviewer 1. Currently working freelance on education and PPI projects; previously worked in a pastoral role at a college; a lay representative for courses training healthcare scientists.	
PPI reviewer 2. Retired primary school teacher with several long term health conditions; single parent; was a young carer for a parent with a long term condition.	
PPI reviewer 3. Former higher education administrator; current university tutor; patient partner on varied research projects; carer for family members aged 0-100 with physical and/or mental health long term conditions.	
PI reviewer 4. Currently working as a civil servant and has several long term health conditions.	
PPI reviewer 5. Full-time parent of school age children; previously ten years working in a medical school in an administrative role and 5 years working in the drug and alcohol sector	
PPI reviewer 6. Lay representative for several healthcare-related professional bodies and involved in health research at several universities; family-carer for over 35 years; has had over 6 years of involvement with a mental and community health as a carer	
PPI reviewer 7. Retired university administrator; a parent and carer for elderly parents.	

For peer review only

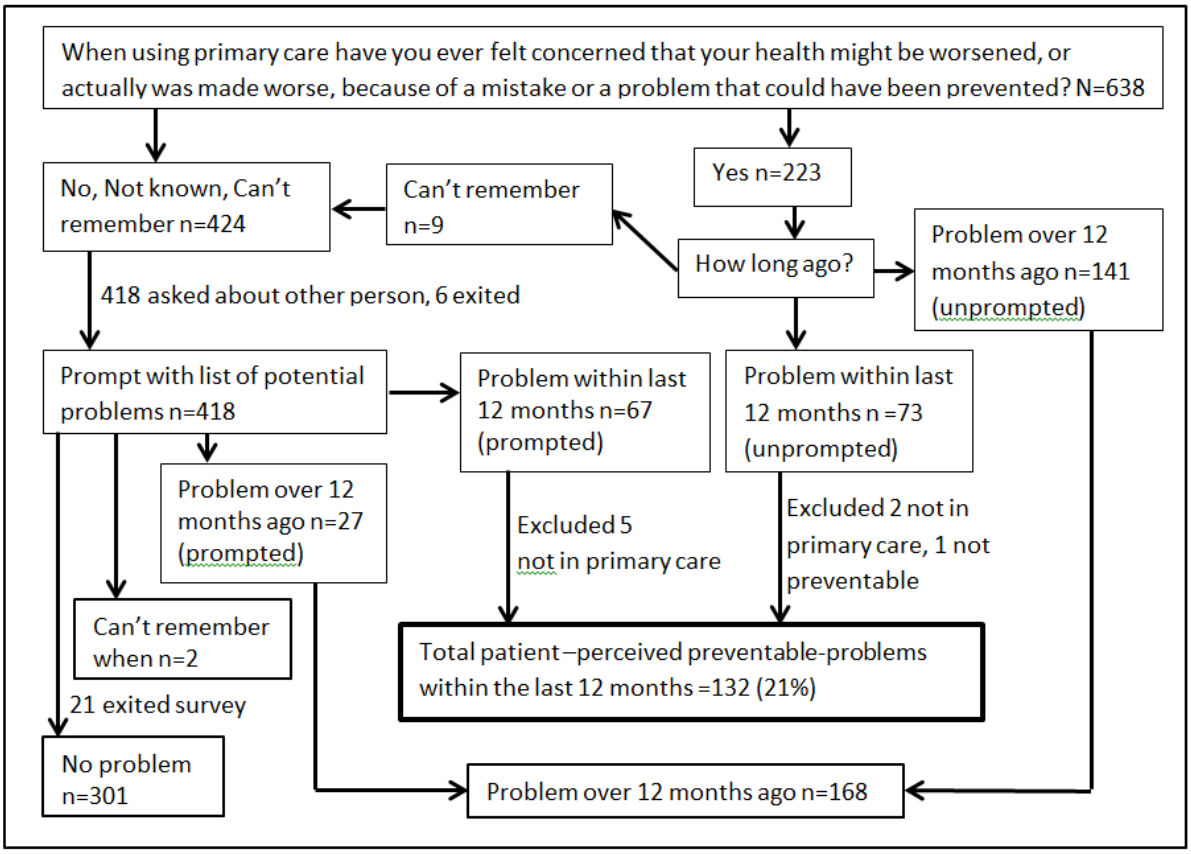

Figure A. Flow chart of participants who reported a potentially-unsafe preventable-problem in primary care through the online pilot survey

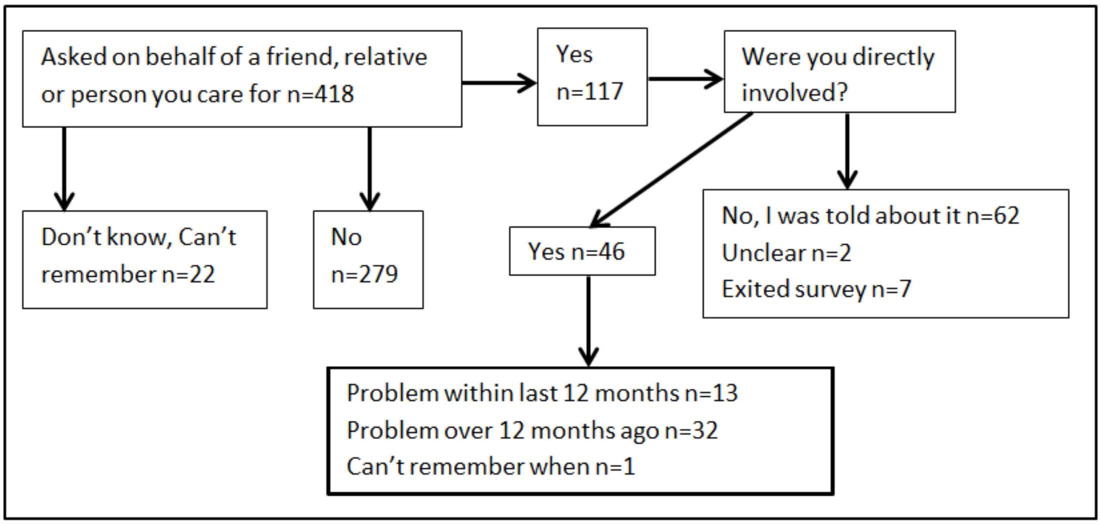

Figure B. Flow chart of participants who reported a potentially-unsafe preventable-problem in primary care on behalf of another person through the online pilot survey

Box A. List of public and patient involvement groups used to distribute the pilot survey
Associate Research User Group of the Greater Manchester Primary Care Patient Safety Translational Research Centre http://research.bmh.manchester.ac.uk/primary-care-patient-safety/GetInvolved/
The Primary Care Research in Manchester Engagement Resource http://research.bmh.manchester.ac.uk/PRIMER/about/
HelpBeatDiabetes https://www.researchforthefuture.org/diabetes/
The Nowgen Centre https://research.cmft.nhs.uk/getting-involved/involvement
The Citizen Scientist project http://www.citizenscientist.org.uk/
North West People in Research Forum https://www.northwestpeopleinresearchforum.org/

**Box B. Pilot survey administered online November and December 2015**
Q1. Did you have confidence and trust in the GP you saw or spoke to at your last appointment?<br>Response options: Yes, definitely, Yes, to some extent, No, not at all, Don't know / can't say
Q2. When using primary care have you ever felt concerned that your health might be worsened, or actually was made worse, because of a mistake or a problem that could have been prevented?<br>Response options: Yes, No- *go to Q10,* Do not understand the question- *go to Q10*, Don't know / can't remember- *go to Q10*
Q3. How long ago did the mistake or preventable problem happen?<br>Response options: Within the last 12 months, More than 12 months ago- *go to Q10,* Can't remember- *go to Q10*
Q4. In your opinion did this experience<br>Response options: Make your health worse, Not certain but it might have made your health worse, Could have made your health worse if you had not noticed the problem, Delayed your treatment but had no effect on your health, Not affect you, or your health, Other, please explain
Q5. Which primary care service were you using when the mistake or preventable problem occurred?<br>Response options: GP surgery, Out of hours care, Walk in clinic, Dental, Pharmacy, Community or district nursing, Ambulance, Opticians, Other- please specify
Q6. Briefly describe the mistake or problem and how it happened<br>Response options: free text
Q7. Could the mistake or problem have been avoided? If so how?<br>Response options: free text
Q8. Were you able to talk about the mistake or problem with anybody working in the primary care service?<br>Response options: Yes, Yes had the opportunity but did not feel comfortable to discuss the mistake or problem, No I could not find anybody with whom I could discuss the mistake or problem, No I was not concerned about the problem, No I did not notice the mistake or problem at the time, I was

For peer review only

too distressed to discuss the mistake or problem, Other or don't know - please describe
Q9. If you discussed the mistake or problem with somebody working in primary care please describe their job or role<br><br>Response options: free text
Q10. In the list below are some examples of preventable problems that might happen when using primary care. Has anything similar happened to you in the last 12 months? Please check as many as applicable or "NONE OF BELOW"<br><br>NONE OF BELOW<br>Wrong or late diagnosis<br>Not referred for further investigation when needed<br>Test results being lost or mixed up<br>Receiving the wrong medicine or wrong dose<br>Should not be prescribed the medicine because of another health problem<br>Should not be prescribed the medicine because of another medication already taking<br>Poor communication leading to misunderstanding of diagnosis or treatment<br>Not referred to a specialist when needed<br>Unclear instructions about treatment<br>Not offering of prevention or screening programmes eg CVD/stroke prevention clinics<br>Failure to recognise or act on vulnerable people's needs eg child abuse, suicide risk or mental health problems<br>Mistake with a procedure eg dental treatment, injection, ear syringing, physiotherapy<br>Failure to notify about recommended vaccinations eg flu, HPV<br>Poor hygiene<br>Unsafe building or premises<br>Any other preventable problem in the last 12 months (in your opinion)<br>Other, please explain below
Q11. Are you male or female? Response options: Male, Female, prefer not to say
Q12. How old are you?<br>Response options: under 16, 16 to 24, 25 to 34, 45 to 54, 55 to 64, 65 to 74, 75 to 84, 85 or older
Q13. When was your last contact with primary care?<br>Response options: Last week, Last month, Last 12 months, Over 12 months ago
Q14. What best describes your usual pattern of use of primary care? Response options: Once per week, Once per 2 weeks, Once per month, Once per 6 months, Once per 12 months or less often
Q15. Are you registered with a GP practice?<br>Response options: Yes, No, I only use walk in centres, Don't know
Q16. Do you work or volunteer in healthcare or healthcare research as a professional, patient, carer or member of the public? (if you are retired answer for your occupation before retirement)<br>Response options: Yes, No
Q17. We are still trying to improve this questionnaire so would be grateful for any feedback about how easy you found the questionnaire to complete? How can it be improved?<br>Response options: free text

For peer review only

Appendix 2.

Table A. Coding of patient-reported potentially-unsafe scenarios in primary care

**1. Errors in the process of the healthcare delivery system**	
Makeham 2002, Dovey 2002	Common threads reported in this study
1.1. Errors in the process of conducting an administrative task	A1. Administrative problem not otherwise specified
1.1.1. Information filed in wrong place or wrong time	
1.1.2. Unavailability of information that should have been in patients charts<br>1.1.2.1. Entire chart or part of chart could not be accessed when needed<br>1.1.2.2. Care provided was not documented<br>1.1.2.3. Item(s) of information missing from chart	A2. Incorrect notes/inadequate notes/notes not kept up to date
1.1.3. Errors in patient's movement through the healthcare delivery system	A3. Intended referral was not sent or delayed<br>A4. Patient not reminded, informed or assisted to attend regular check-ups or other necessary routine treatments
1.1.4. Errors in the taking and distributing of messages	
1.1.5. Errors in managing appointments for healthcare	A5. Unable to get an appointment/other problems with making appointment<br>A6. Ambulance delayed or did not arrive
**1.2. Errors in the process of investigating a patient's condition**	
1.2.1. Laboratory errors<br>1.2.1.1. Wrong test ordered or test not ordered when appropriate<br>1.2.1.2. Errors in the process of obtaining or processing a laboratory specimen<br>1.2.1.3. Error in the process of physician receiving accurate laboratory results in a timely fashion<br>1.2.1.4. Inappropriate response to an abnormal laboratory result<br>1.2.3. Errors in the processes of other investigations<br>1.2.3.1. Wrong test ordered or test not ordered when appropriate<br>1.2.3.2. Errors in the process of obtaining or processing of other diagnostic investigation<br>1.2.3.3. Error in the process of physician receiving accurate test results of other investigation in a timely fashion<br>1.2.3.4. Inappropriate response to an abnormal result of other investigation	B1. Test results lost or other problem with investigation paperwork<br>B2. Incorrect interpretation of tests or other investigation results<br>B3. Clinician did not consider patient history sufficiently/did not use patient's notes adequately<br>B4. Investigation not thorough enough<br>B5. Not referred when patient felt was needed
**1.3. Errors in the process of treating a patient's condition**	
1.3.1. Errors in the process of treating with medications<br>1.3.1.1. Wrong medication or wrong dose of medication ordered or medication not ordered by physician when appropriate<br>1.3.1.2. Error in the process of delivering a medication order or inappropriate medication order by a provider working under physician supervision<br>1.3.1.3. Error in the process of dispensing medication as ordered	C1. Medication problem<br><br>C2. Not provided with medical devices needed to manage long term conditions
1.3.2. Errors in other treatments	C3. Problem with dental treatment or

For peer review only

	diagnosis
**1.4. Errors in the process of communication**	
1.4.1. Errors in communication between primary healthcare provider and patients	D1. Clinician seemed to lack interest in the patient's health problem or did not listen carefully enough<br>D2. Information about the patient's health had not been passed on to the patient who felt it should have been<br>D3. Communication problem between patient and primary care staff
1.4.2. Errors in communication between healthcare providers	D4. Problem with communication between primary care and other types of care including secondary care<br>D5. Disagreement between 2 clinicians
**2. Errors arising from lack of clinical knowledge or skills**	
2.1. Errors in the execution of a clinical task<br>2.1.1. Non-clinical staff made the wrong clinical decision<br>2.1.2. Failed to follow standard practice<br>2.1.3. Lacked needed experience or expertise in a clinical task	E1. Administrative staff seemed to make clinical decisions<br>E2. Procedure was not carried out correctly<br>E3. Incorrect advice/no advice given by clinician
**2.2. Errors in diagnosis**<br>2.2.1. Wrong or delayed diagnosis	F1. Wrong/late/missed/delayed diagnosis
**2.3. Wrong treatment decision**	G1. Wrong treatment decision
	H. Other
	X. Not a problem/ insufficient information/refused/don't know

Table B. Level 4 coding of patient-reported potentially-unsafe medication scenarios

Common threads reported in this study grouped as described by Makeham 2002, Dovey 2002
C1 Medication error not otherwise specified /other problem
• **1.3.1.1. Ordering medications (prescribing)**
C1.1.1 Prescribed wrong or inappropriate drug
C1.1.2 Started new prescription or changed prescription without sufficient discussion, follow up or checks
C1.1.3 Long term or continued prescribing without review or consideration of long term or side effects
C1.1.4 Prescribed drug when should have known contra-indicated *e.g.* patient had informed clinician of allergy, adverse reaction or it was in the records
C1.1.5 Repeat prescription unintentionally changed
C1.1.6 Out of date repeat prescription mistakenly re-issued
• **1.3.1.2./1.3.1.3. Implementing or receiving medications (dispensing or issuing)**
C1.2.1 Medication not dispensed or administered as intended or prescribed
• **1.3.1.1/1.3.1.2./1.3.1.3. Ordering, implementing or receiving medications**
C1.3.1 Wrong dose or drug or delivery method
C1.3.2 Being given another patient's drugs or prescription
C1.3.3 Wrong or inadequate advice about drug effects or how to use
C1.3.4 Delay or failure in prescription processing

For peer review only

Table C. Scoring for likelihood that the patient-reported scenario is potentially-unsafe

Score	How likely do you think it is the patient was correct in thinking that their health might be worsened, or actually was made worse, because of a mistake or a problem in primary care that could have been prevented? Choose from the options below.
5	Very likely or certain (75-100% confident is a potentially unsafe scenario)
4	Probably (50-74% confident is a potentially unsafe scenario)
3	Possibly (25-49% confident is a potentially unsafe scenario)
2	Unlikely (bottom 25% confident is a potentially unsafe scenario)
1	Definitely not a potentially unsafe event (0% chance is a potentially unsafe scenario)
-	Insufficient information
-	Don't know
-	Other - add text at end of row

For peer review only

Patient reported scenarios occurring during the past 12 months that GPs scored as higher likelihood to be a potentially-unsafe preventable-problem in primary care (median score is higher than "possibly" and at least 2 GPs gave a score or one GP scored "very likely or certain") originating from the pilot survey

Scenario4. GP surgery

Briefly describe the mistake or problem and how it happened. *"Prescription drug, anti-inflammatory for arthritis, caused acute stomach pains & violent vomiting. Repeat prescription for twelve years without any discussion."*

Could the mistake or problem have been avoided? If so how? "*Possible discussion about dangers of continuous taking of prescription drugs, which in the event were stopped after the incident."*

Were you able to talk about the mistake or problem with anybody working in the primary care service? *"No I did not notice the mistake or problem at the time"*

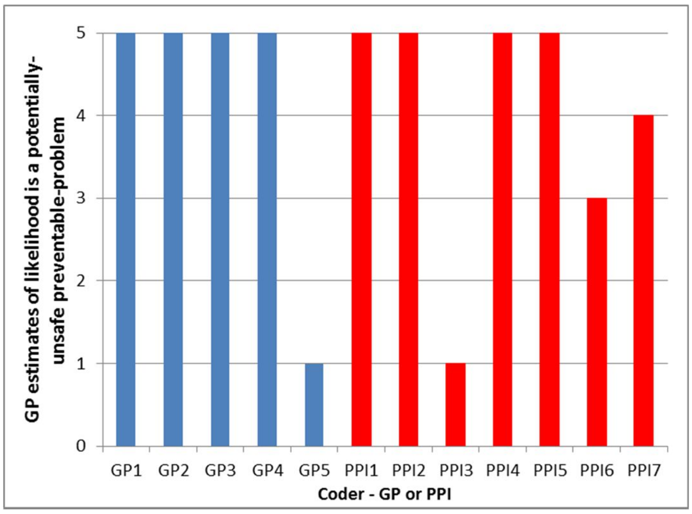

5=very likely or certain, 4=probably, 3=possibly, 2=unlikely, 1=definitely not, 0 = insufficient information or don't know

Patient-reported prospect of harm: suspected your health has been made worse by a problem or error that could have been prevented

Patient-perspective problem-type code: C1.1.3 Long term or continued prescribing without review or consideration of long term or side effects

Scenario236. GP surgery

Briefly describe the mistake or problem and how it happened. *"Insulin type was changed by specialist but previous insulin prescribed by GP as notes had not been updated"*

Could the mistake or problem have been avoided? If so how? "*Yes GP notes should have been updated with new medication"*

Were you able to talk about the mistake or problem with anybody working in the primary care service? *"Practice manager resolved the problem and apologised"*

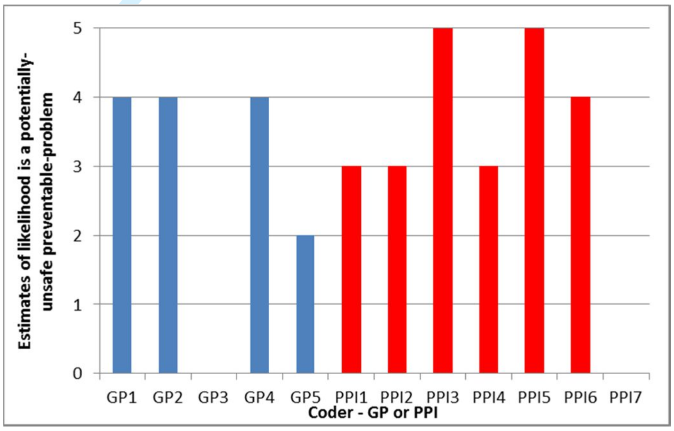

5=very likely or certain, 4=probably, 3=possibly, 2=unlikely, 1=definitely not, 0 = insufficient information or don't know

Patient-reported prospect of harm: prompted via Q10 (Box 1 main paper)

Patient-perspective problem-type code: A2. Incorrect notes/inadequate notes/notes not kept up to date; C1.1.6 Out of date repeat prescription mistakenly re-issued

For peer review only - http://bmjopen.bmj.com/site/about/guidelines.xhtml

Scenario229. GP surgery

Briefly describe the mistake or problem and how it happened. *"Two out of three Doctors not listening to what I was asking; April I had two big bleeds from my Penis, Doctor 1 did a test and gave antibiotics. Went to 2nd Doctor for Diabetic check and told him of problem - nothing except another test come back in ten days. Went to the third doctor who said the test didn't show anything but when I mentioned my feelings about a problem, he look and said yes you do have a problem. In 2 weeks I was in having tests and 3 operations for cancer."*

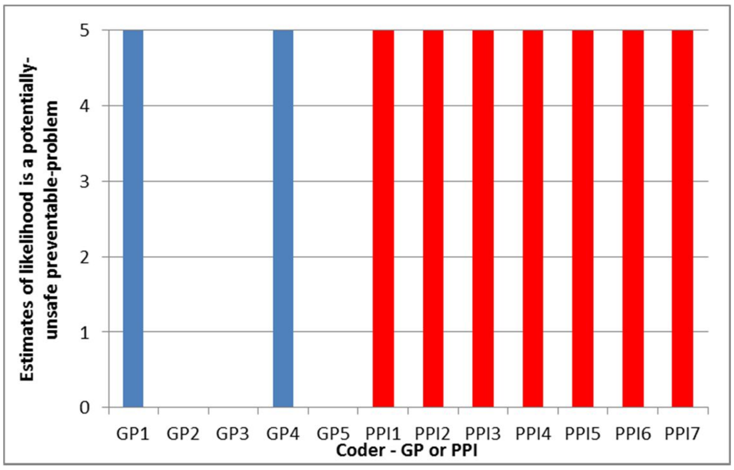

5=very likely or certain, 4=probably, 3=possibly, 2=unlikely, 1=definitely not, 0 = insufficient information or don't know

Could the mistake or problem have been avoided? If so how? "*Listen to me"*

Were you able to talk about the mistake or problem with anybody working in the primary care service? *"No, I could not find anybody with whom I could discuss the mistake or problem (The third doctor was amazing with me. He said to keep in touch and if I had any problems to ring him and he still wants me to ring him after my three operations.)"*

Patient-reported prospect of harm: suspected your health has been made worse by a problem or error that could have been prevented

Patient-perspective problem-type code: D1. Clinician seemed to lack interest in the patient's health problem or did not listen carefully enough; F1. Wrong/late/missed/delayed diagnosis

Scenario113. GP surgery

Briefly describe the mistake or problem and how it happened. *"Changed diabetes medication to an alternative which my notes from 1980's should show I respond badly to"*

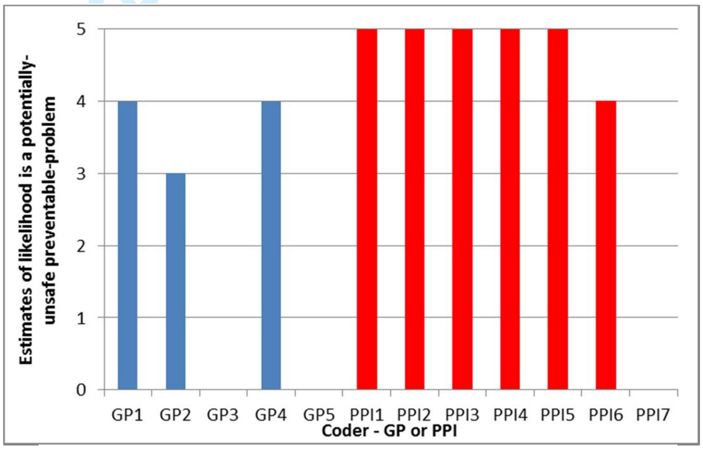

5=very likely or certain, 4=probably, 3=possibly, 2=unlikely, 1=definitely not, 0 = insufficient information or don't know

Could the mistake or problem have been avoided? If so how? "*Read the notes on every medication change but unfortunately that is unrealistic under the time restrictions on GP's. Put early notes on-line and flag medication allergies/problems."*

Were you able to talk about the mistake or problem with anybody working in the primary care service? *"Yes, my own GP who had returned from holiday"*

Patient-reported prospect of harm: suspected your health has been made worse by a problem or error that could have been prevented

Patient-perspective problem-type code: C1.1.4 Prescribed drug when should have known contra-indicated *e.g.* patient had informed clinician of allergy, adverse reaction or it was in the records

For peer review

Scenario297. GP surgery

Briefly describe the mistake or problem and how it happened. *"Told the GP the medication was making my hair fall out & he kept me on it for another 3 months. I had to see another GP to get him to change my medication. In the meantime I have lost 3/4 of my hair. Not sure if it will ever grow back."*

Could the mistake or problem have been avoided? If so how? "*yes, by the GP listening to what I was saying."*

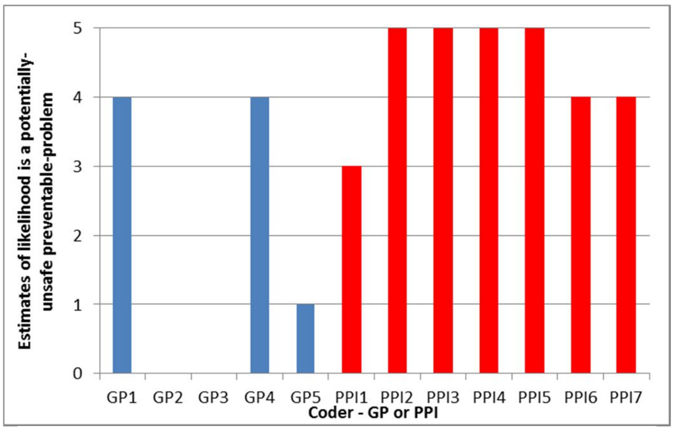

5=very likely or certain, 4=probably, 3=possibly, 2=unlikely, 1=definitely not, 0 = insufficient information or don't know

Were you able to talk about the mistake or problem with anybody working in the primary care service? *"Yes, GP"*

Patient-reported prospect of harm: suspected your health has been made worse by a problem or error that could have been prevented

Patient-perspective problem-type code: D1. Clinician seemed to lack interest in the patient's health problem or did not listen carefully enough; C1.1.3 Long term or continued prescribing without review or consideration of long term or side effects

Scenario177. GP surgery

Briefly describe the mistake or problem and how it happened. *"Successfully treated for prostate cancer 2006 but suffered some loss of sexual performance; Viagra recommended BUT I take isosorbide nitrate for a following heart attack; the two are contradictory and could produce further heart problems. A routine diabetes check-up at which the sexual problem was discussed saw an automatic prescribing of Viagra; obviously without reference to my medical records."*

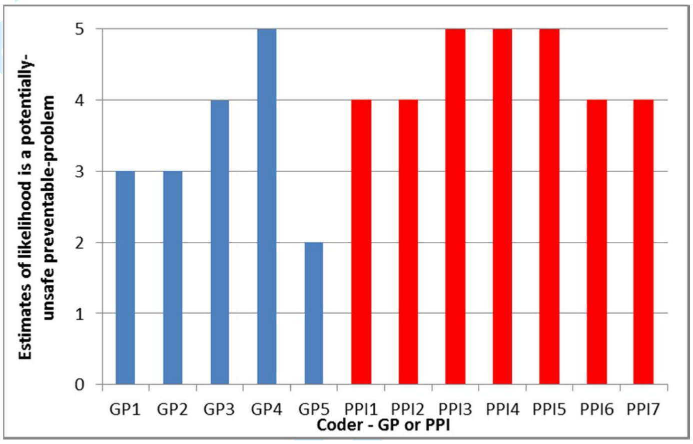

Could the mistake or problem have been avoided? If so how? "*Read the medical notes."*

5=very likely or certain, 4=probably, 3=possibly, 2=unlikely, 1=definitely not, 0 = insufficient information or don't know

Were you able to talk about the mistake or problem with anybody working in the primary care service? *"No; I felt I was going to cause trouble"*

Patient-reported prospect of harm: prompted via Q10 (Box 1 main paper)

Patient-perspective problem-type code: C1.1.1 Prescribed wrong or inappropriate drug

Scenario404. GP surgery

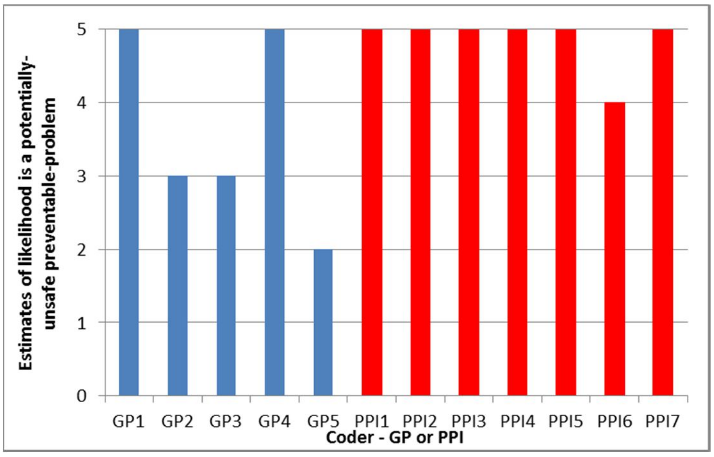

5=very likely or certain, 4=probably, 3=possibly, 2=unlikely, 1=definitely not, 0 = insufficient information or don't know

Briefly describe the mistake or problem and how it happened. *"I was given steroids for a chest infection but not alerted to the fact they make your sugars go massively high! Within a few hours I was high and not able to bring them down, fearing a DKA I headed for the hospital to correct a very easily avoidable issue. I also attended my GP 6 years ago to be given strong antacids for pain in my stomach that was actually a DKA I was admitted to hospital a few hours later! The GP never even suggested it could be linked to my diabetes and as it was my first DKA I had no idea that's how they can feel"*

Could the mistake or problem have been avoided? If so how? "*Both could have been avoided The steroids - if the prescribing nurse had considered my diabetes I'd have been given proper advice as to how to deal with them as a diabetic or given different meds. The DKA simple questions or explanation as to how DKAs can present would have made me family and the doctor realise I was in trouble."*

Were you able to talk about the mistake or problem with anybody working in the primary care service? *"I wrote a letter to the surgery concerning the steroids anonymously to alert them of my concern and the DKA. I was too poorly to even consider seeking correction or explanation"*

Patient-reported prospect of harm: health was actually made worse by a problem or error that could have been prevented

Patient-perspective problem-type code: C1.1.4 Prescribed drug when should have known contra-indicated *e.g.* patient had informed clinician of allergy, adverse reaction or it was in the records; E3. Incorrect advice/no advice given by clinician

Scenario29. GP surgery

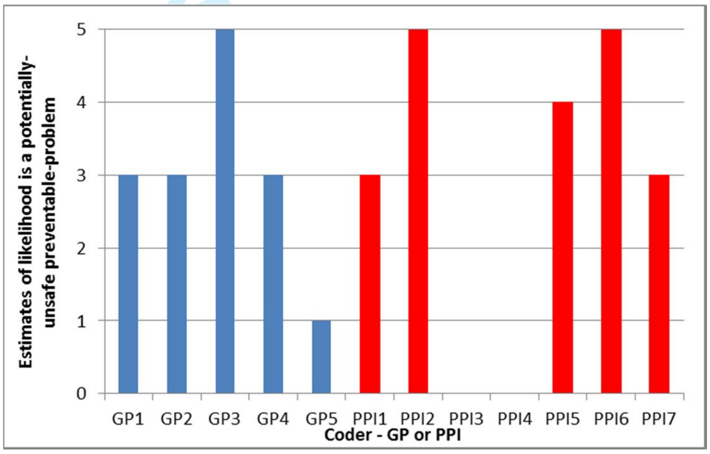

5=very likely or certain, 4=probably, 3=possibly, 2=unlikely, 1=definitely not, 0 = insufficient information or don't know

Briefly describe the mistake or problem and how it happened. *"reception staff making clinical decisions which were at odds with what had been discussed with my GP"*

Could the mistake or problem have been avoided? If so how? "*Yes, reception staff shouldn't be making clinical decisions"*

Were you able to talk about the mistake or problem with anybody working in the primary care service? *"No, had the opportunity but did not feel comfortable to discuss the mistake or problem"*

Patient-reported prospect of harm: suspected your health has been made worse by a problem or error that could have been prevented

Patient-perspective problem-type code: E1. Administrative staff seemed to make clinical decisions

For peer review only

Scenario621. Pharmacist

Briefly describe the mistake or problem and how it happened. *"I was given a medicine belonging to somebody else as part of my monthly repeat prescription"*

Could the mistake or problem have been avoided? If so how? "*More care and attention when checking"*

Were you able to talk about the mistake or problem with anybody working in the primary care service? *"Yes, pharmacist"*

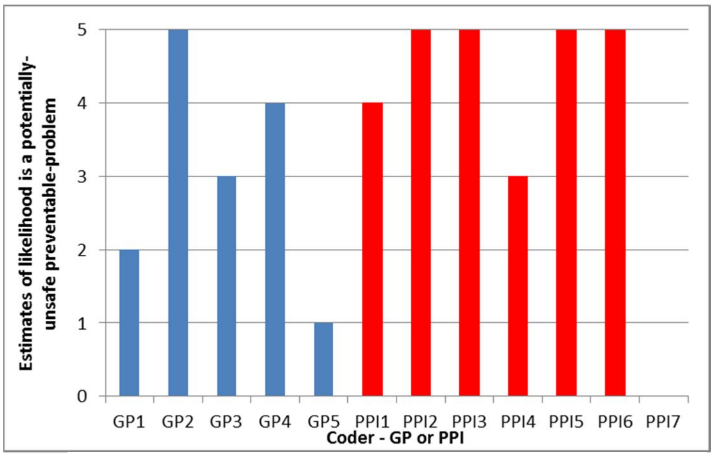

Patient-reported prospect of harm: prompted via Q10 (Box 1 main paper)

5=very likely or certain, 4=probably, 3=possibly, 2=unlikely, 1=definitely not, 0 = insufficient information or don't know

Patient-perspective problem-type code: C1.3.3 Wrong or inadequate advice about drug effects or how to use

Scenario296. GP surgery

Briefly describe the mistake or problem and how it happened. *"Poor diabetic annual review, foot check not correctly done just tested my foot pulses and nothing else"*

Could the mistake or problem have been avoided? If so how? "*Better training of staff"*

Were you able to talk about the mistake or problem with anybody working in the primary care service? *"No, had the opportunity but did not feel comfortable to discuss the mistake or problem"*

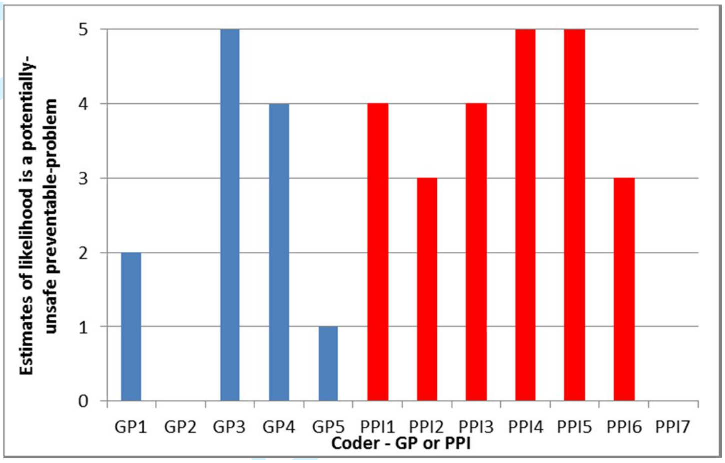

Patient-reported prospect of harm: suspected your health has been made worse by a problem or error that could have been prevented

5=very likely or certain, 4=probably, 3=possibly, 2=unlikely, 1=definitely not, 0 = insufficient information or don't know

Patient-perspective problem-type code: E2. Procedure was not carried out correctly

For peer review only

Scenario239. GP surgery

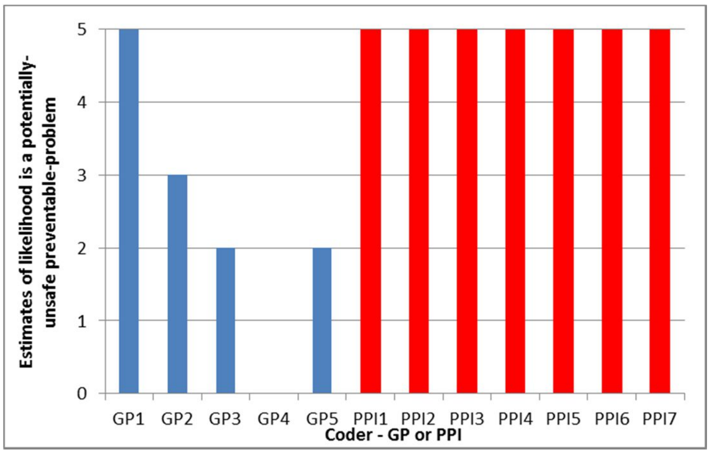

5=very likely or certain, 4=probably, 3=possibly, 2=unlikely, 1=definitely not, 0 = insufficient information or don't know

Briefly describe the mistake or problem and how it happened. *"Prior to a pain killing injection into my knee, I asked the GP who suggested the injection AND the GP who carried out the injection whether, as someone living with Type 1 diabetes, it would have any effect on my blood glucose levels. On both occasions, I was given an unequivocal No . In the event, within a few hours of the injection, my blood glucose rose significantly and remained high for several days. I felt unable to eat anything for 24 hours while I took on more and more insulin in order to bring my glucose levels down - I did not want to go to sleep that night simply because of the massive amount of insulin in my system."*

Could the mistake or problem have been avoided? If so how? "*Yes. I feel that both GPs should have a knowledge about the side effects of drugs they prescribe, administer and recommend."*

Were you able to talk about the mistake or problem with anybody working in the primary care service? *"No I could not find anybody with whom I could discuss the mistake or problem"*

Patient-reported prospect of harm: your health has been made worse by a problem or error that could have been prevented

Patient-perspective problem-type code: E3. Incorrect advice/no advice given by clinician

For peer review

Scenario87. GP surgery

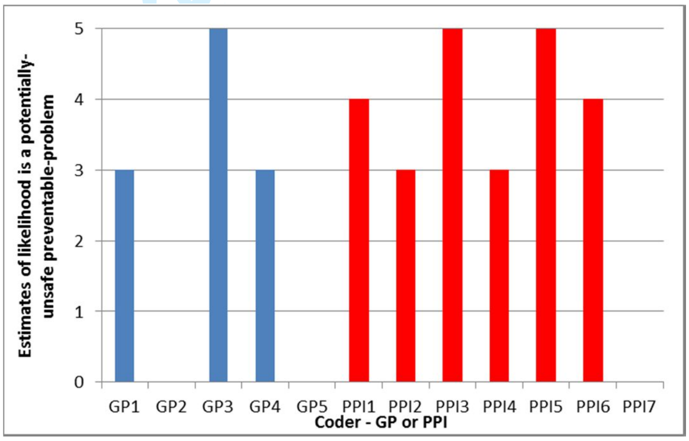

5=very likely or certain, 4=probably, 3=possibly, 2=unlikely, 1=definitely not, 0 = insufficient information or don't know

Briefly describe the mistake or problem and how it happened. *"GP completely overlooked symptoms and prescribed antibiotic after antibiotic without investigation or referral"*

Could the mistake or problem have been avoided? If so how? "*Yes by listening to history of complaints, carrying out appropriate tests instead of just giving antibiotics"*

Were you able to talk about the mistake or problem with anybody working in the primary care service? *"No I did not notice the mistake or problem at the time"*

Patient-reported prospect of harm: prompted via Q10 (Box 1 main paper)

Patient-perspective problem-type code: D1. Clinician seemed to lack interest in the patient's health problem or did not listen carefully enough; F1. Wrong/late/missed/delayed diagnosis

Scenario294. GP surgery

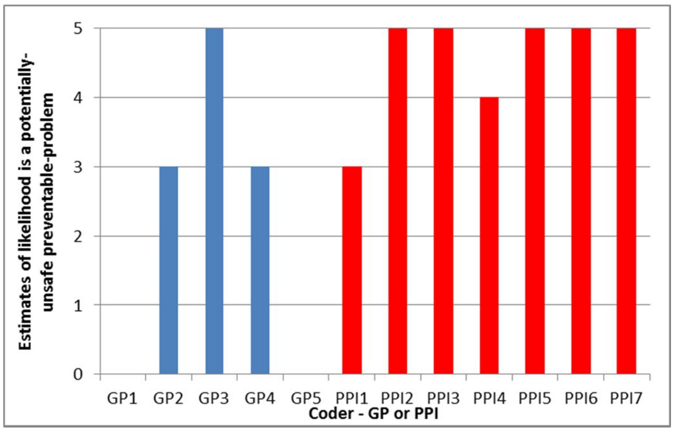

5=very likely or certain, 4=probably, 3=possibly, 2=unlikely, 1=definitely not, 0 = insufficient information or don't know

Briefly describe the mistake or problem and how it happened. *"Several times prescriptions have been incorrectly issued due to similar names for drugs or the same name with different strengths"*

Could the mistake or problem have been avoided? If so how? "*Yes, by more accurate or double data entry. Now solved by self-request using web systems."*

Were you able to talk about the mistake or problem with anybody working in the primary care service? "Yes, *they did not want to know or seem to care unless a formal complaint was made"*

Patient-reported prospect of harm: your health has been made worse by a problem or error that could have been prevented

Patient-perspective problem-type code: C1.1.5 Repeat prescription unintentionally changed

Scenario327. GP surgery

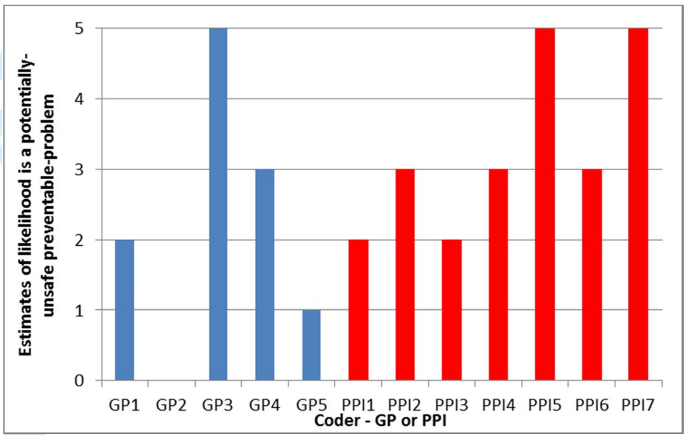

5=very likely or certain, 4=probably, 3=possibly, 2=unlikely, 1=definitely not, 0 = insufficient information or don't know

Briefly describe the mistake or problem and how it happened. *"A simple error occurred with an incorrect prescription. When I tried to bring this to the attention of the receptionist she treated me with disdain and in a challenging manner. She then proceeded to start to read my notes aloud in the public reception area. I felt that this was unacceptable behaviour. When I tried to tackle the receptionist about her behaviour I felt as if I was under threat. It caused me to feel very stressed, frustrated and ill tempered."*

Could the mistake or problem have been avoided? If so how? "*If the receptionist had been willing to listen to what I was saying."*

Were you able to talk about the mistake or problem with anybody working in the primary care service? *"I did speak to a lady who said she was the practice manager but I felt that they were not interested in resolving the problem"*

Patient-reported prospect of harm: suspected your health has been made worse by a problem or error that could have been prevented

Patient-perspective problem-type code: D3. Communication problem between patient and primary care staff; C1 Medication error not otherwise specified /other problem

For peer review only

Scenario330. GP Surgery

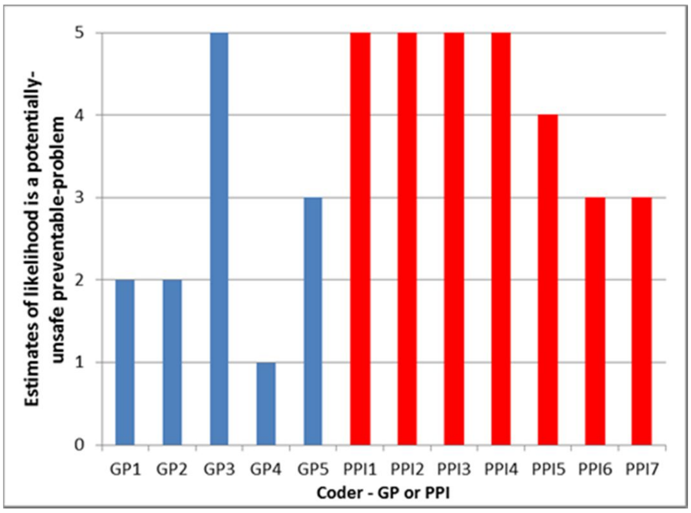

5=very likely or certain, 4=probably, 3=possibly, 2=unlikely, 1=definitely not, 0 = insufficient information or don't know

Briefly describe the mistake or problem and how it happened. *"Went to see GP because I feared the pain in one of my legs may have been Peripheral Artery Disease - hardening of the arteries, having had a (non-blood) relative who suffered from this and subsequently died - of a heart attack. Oh yes, said the GP, well, you will have it won't you? Why? I asked expecting her to say eg because you are a smoker, or maybe my age (65) or something else I wasn't aware of. But what she actually told me was 'Because you are a diabetic!' Whaaat? I exclaimed - you mean ALL diabetics will inevitably get this, and there's no way to prevent it? Yes she said and shrugged. I said 'Thanks for nothing then' and left. Instead I left, came home and went straight on-line to make an appointment with someone more sensible, which I did and after taking my leg/ankle pulses and BPs etc - he chatted to me and said he would refer me for a cardiology consultation at the hospital. This IS what I expected in the first place and now it IS being taken care of***."**

Could the mistake or problem have been avoided? If so how? *"By training the GP properly in the first place"*

Were you able to talk about the mistake or problem with anybody working in the primary care service? "? *"I explained to GP 2 But I don't know what if anything was done about it, or how I could find that out."*

Patient-reported prospect of harm: your health has been made worse by a problem or error that could have been prevented

Patient-perspective problem-type code: D1. Clinician seemed to lack interest in the patient's health problem or did not listen carefully enough

Patient reported scenarios occurring during the past 12 months that PPIs scored as higher likelihood to be a potentially-unsafe preventable-problem in primary care compared with GPs – pilot survey

Scenario3/179. GP Surgery

Briefly describe the mistake or problem and how it happened. *"I had a severe reaction to Atorvastatin after a dose increase so much so that I was almost immobile and took 4 months to recover"*

Could the mistake or problem have been avoided? If so how? *"According to guidelines I should have been on the increased dose - it took a long time to convince the GP that I needed blood tests to find out why I couldn't walk. My GP was very hesitant to admit that I did have a reaction to statins."*

Were you able to talk about the mistake or problem with anybody working in the primary care service? **"***No I could not find anybody with whom I could discuss the mistake or problem. It was not really the GPs fault per se, just took a lot of convincing that there was a problem"*

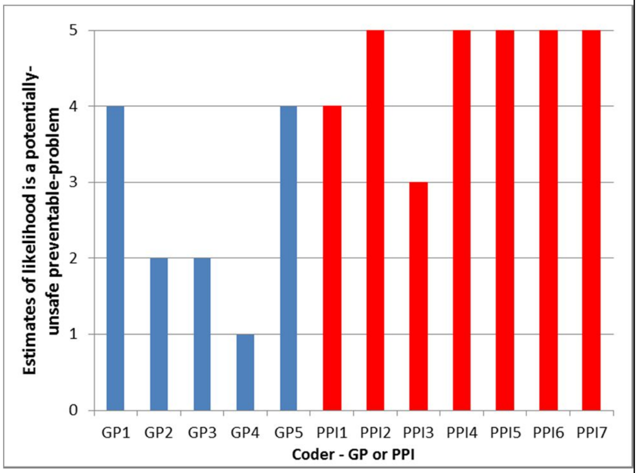

5=very likely or certain, 4=probably, 3=possibly, 2=unlikely, 1=definitely not, 0 = insufficient information or don't know

Patient-reported prospect of harm: health could have been made worse had someone not noticed a problem or error

Patient-perspective problem-type code: C1.1.3 Long term or continued prescribing without review or consideration of long term or side effects

For peer review only

Scenario3/285. GP Surgery

Briefly describe the mistake or problem and how it happened. *"Doctor kept saying I had vitamin deficiency B1, it turned out I had peripheral neuropathy which is very painful"*

Could the mistake or problem have been avoided? If so how? *"I just needed the proper medication to help"*

Were you able to talk about the mistake or problem with anybody working in the primary care service? *"Just saw another Doctor and she knew straight away what the problem was - she was experienced with Diabetic problems. Yes had the opportunity but did not feel comfortable to discuss the mistake or problem"*

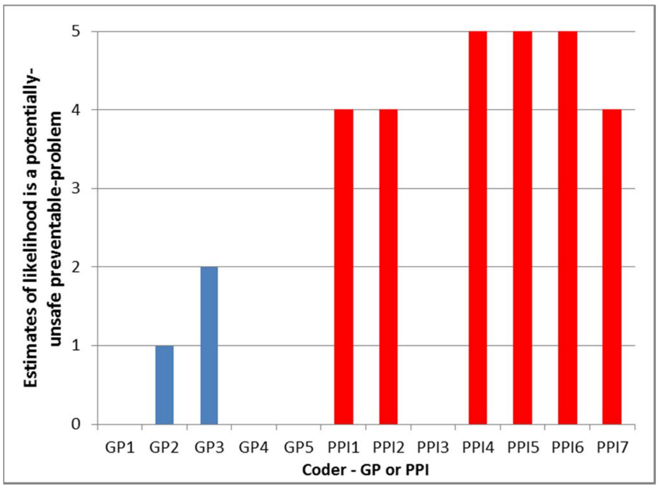

5=very likely or certain, 4=probably, 3=possibly, 2=unlikely, 1=definitely not, 0 = insufficient information or don't know

Patient-reported prospect of harm: prompted via Q10 (Box 1 main paper)

Patient-perspective problem-type code: F1. Wrong/late/missed/delayed diagnosis

Scenario3/347. GP Surgery

Briefly describe the mistake or problem and how it happened. *"Incapable diabetic doctor trying to take blood out the back of my hand haphazardly, not listening and resulting in me fitting and the student watching having to get help."*

Could the mistake or problem have been avoided? If so how? *"Yes. By listening to me"*

Were you able to talk about the mistake or problem with anybody working in the primary care service? *"No I could not find anybody with whom I could discuss the mistake or problem"*

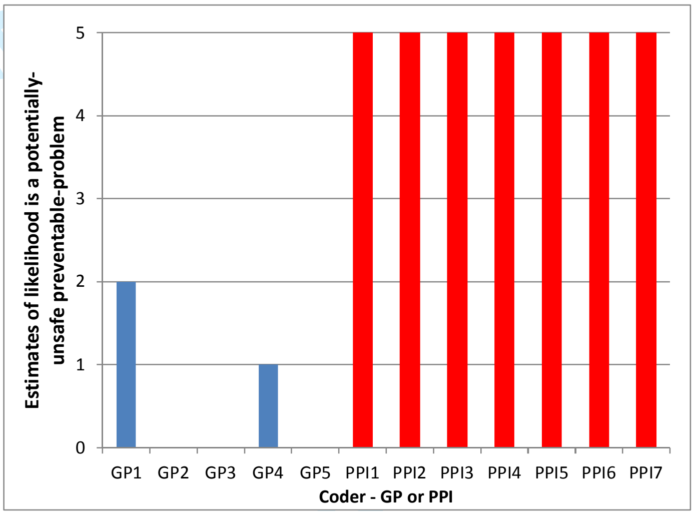

5=very likely or certain, 4=probably, 3=possibly, 2=unlikely, 1=definitely not, 0 = insufficient information or don't know

Patient-reported prospect of harm: prompted via Q10 (Box 1 main paper)

Patient-perspective problem-type code: E2. Procedure was not carried out correctly; D1. Clinician seemed to lack interest in the patient's health problem or did not listen carefully enough

Scenario3/384. Dental Surgery

Briefly describe the mistake or problem and how it happened. *"I had an infection under my wisdom tooth. They agreed that the only way to solve the problem was to take the tooth out. They gave me an appointment to do this in 6 weeks. I am a type 1 diabetic and the infection was affecting my blood sugars and I was concerned that I would have to go to A&E if my blood sugars continued to rise due to the infection. It would have affected my health if I had not paid to go to a private dentist."*

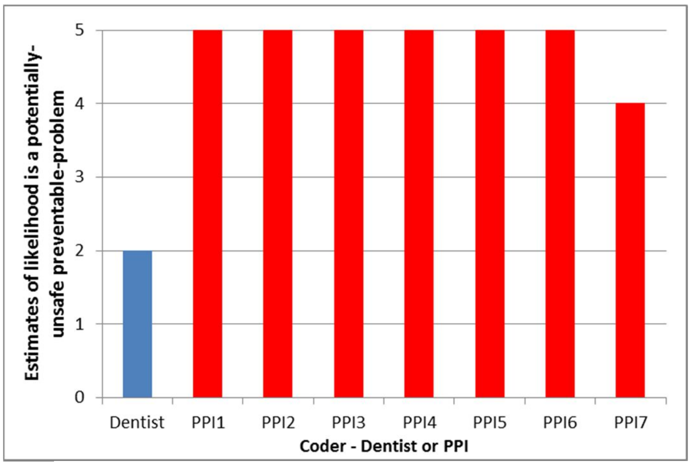

5=very likely or certain, 4=probably, 3=possibly, 2=unlikely, 1=definitely not, 0 = insufficient information or don't know

Could the mistake or problem have been avoided? If so how? *"They could have taken out the tooth straight away. I was happy to wait at the emergency dentist for them to do this."*

Were you able to talk about the mistake or problem with anybody working in the primary care service? *"I explained but they said I would have to wait. They also asked if I needed a sugary drink when I said that my sugars were high so I was too scared to eat and had not eaten in 12hrs. It was clear they didn't understand diabetes."*

Patient-reported prospect of harm: health could have been made worse had someone not noticed a problem or error

Patient-perspective problem-type code: A5. Unable to get an appointment/other problems with making appointment

Scenario3/366. Dental Surgery

Briefly describe the mistake or problem and how it happened. *"Caries, cavities and problem with crown not diagnosed or treated"*

Could the mistake or problem have been avoided? If so how? *"Better dentist & not working to tight time-scale imposed by company owning dental surgery"*

Were you able to talk about the mistake or problem with anybody working in the primary care service? *"No I could not find anybody with whom I could discuss the mistake or problem"*

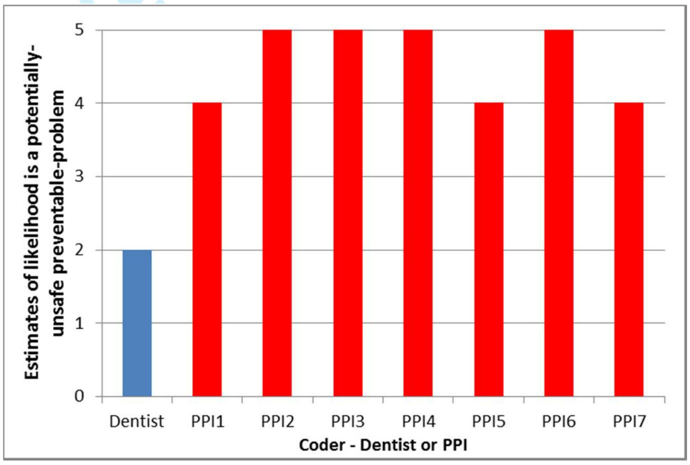

5=very likely or certain, 4=probably, 3=possibly, 2=unlikely, 1=definitely not, 0 = insufficient information or don't know

Patient-reported prospect of harm: prompted via Q10 (Box 1 main paper)

Patient-perspective problem-type code: C3. Problem with dental treatment or diagnosis

For peer review

Scenario3/458. GP Surgery

Briefly describe the mistake or problem and how it happened. *"Using the summary on discharge from hospital, one GP transcribed incorrectly on to my electronic notes ie size of ovarian cyst was 7.5cms and he put 7.5 mms. Another GP requested diagnostic bone density scan but either forgot or did not record it and she ended up questioning why I had it and who requested it. She also referred me for an orthopedic consultation then said I was not funded for the steroid injection put into my swollen elbows."*

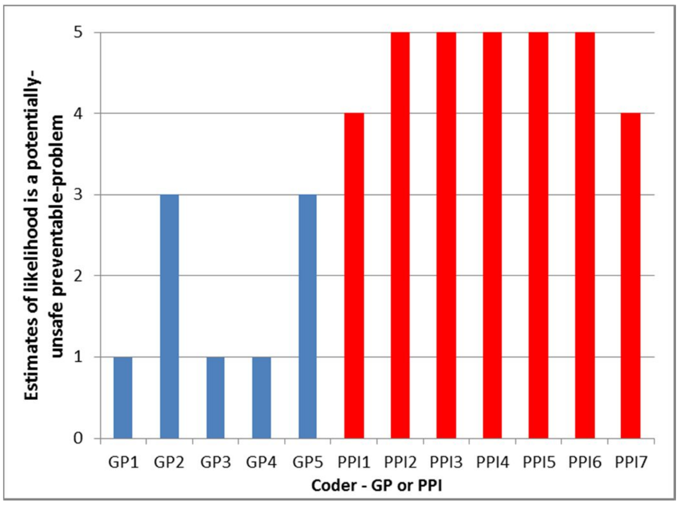

5=very likely or certain, 4=probably, 3=possibly, 2=unlikely, 1=definitely not, 0 = insufficient information or don't know

Could the mistake or problem have been avoided? If so how? *"Yes"*

Were you able to talk about the mistake or problem with anybody working in the primary care service? *"I was too scared to discuss my concerns for fear of being labelled a trouble maker"*

Patient-reported prospect of harm: health could have been made worse had someone not noticed a problem or error

Patient-perspective problem-type code: A2. Incorrect notes/inadequate notes/notes not kept up to date

Scenario3/484. GP Surgery

Briefly describe the mistake or problem and how it happened. *"GP prescribed pills, but then got phone call saying not to take them"*

Could the mistake or problem have been avoided? If so how? *"Not sure"*

Were you able to talk about the mistake or problem with anybody working in the primary care service? *"No I was not concerned about the problem"*

Patient-reported prospect of harm: prompted via Q10 (Box 1 main paper)

Patient-perspective problem-type code: C1. Medication problem

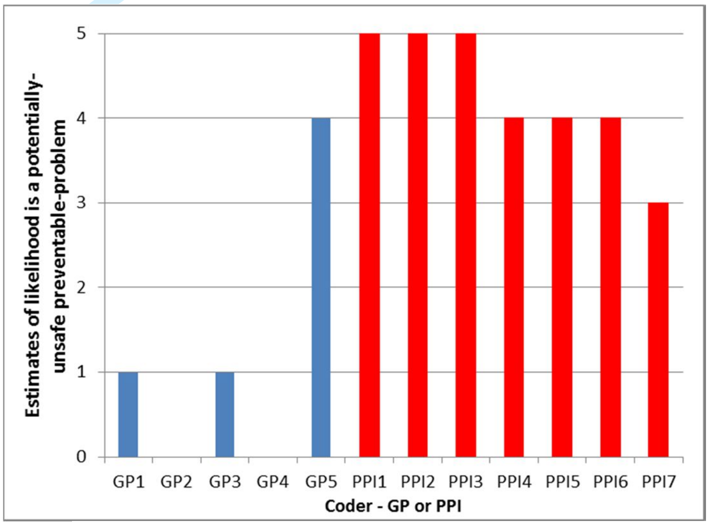

5=very likely or certain, 4=probably, 3=possibly, 2=unlikely, 1=definitely not, 0 = insufficient information or don't know

For peer review only - http://bmjopen.bmj.com/site/about/guidelines.xhtml

Scenario3/555. GP Surgery

Briefly describe the mistake or problem and how it happened. *"I had a burst appendix and peritonitis, something that even a scan couldn't detect adequately. My first visit to GP was when I said I think I have appendicitis, no other symptoms only the pain. It was ten days before seeing a consultant, a further 10 days to have a scan, then 2 weeks to be told that I had a lump on my colon which is what my GP had said 5 weeks previously. It was a further 2 weeks before I had surgery."*

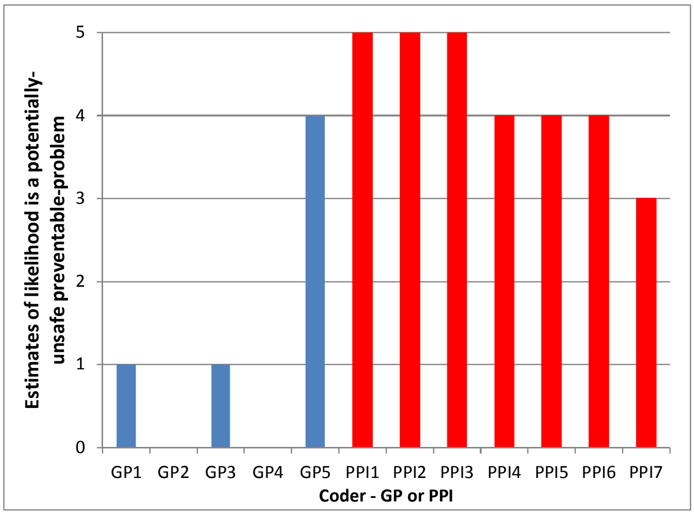

5=very likely or certain, 4=probably, 3=possibly, 2=unlikely, 1=definitely not, 0 = insufficient information or don't know

Could the mistake or problem have been avoided? If so how? *"If my GP had referred me for a scan immediately it would have saved 3 weeks out of the seven. It was two weeks from scan to results and I hear that is usual, but they're not looking at them for 2 weeks"*

Were you able to talk about the mistake or problem with anybody working in the primary care service? *"Had the outcome been different my widow might have pursued the matter further. The system is at fault rather than any individual."*

Patient-reported prospect of harm: your health has been made worse by a problem or error that could have been prevented

Patient-perspective problem-type code: B5. Not referred when patient felt was needed

For peer review only

STROBE Statement—Checklist of items that should be included in reports of ***cross-sectional studies***

	Item No	Recommendation
**Title and abstract**	1	(*a*) Indicate the study's design with a commonly used term in the title or the abstract Yes p1
		(*b*) Provide in the abstract an informative and balanced summary of what was done and what was found yes p2
**Introduction**		
Background/rationale	2	Explain the scientific background and rationale for the investigation being reported Yes p3
Objectives	3	State specific objectives, including any prespecified hypotheses yes p3-4
**Methods**		
Study design	4	Present key elements of study design early in the paper yes p4
Setting	5	Describe the setting, locations, and relevant dates, including periods of recruitment, exposure, follow-up, and data collection yes p4
Participants	6	(*a*) Give the eligibility criteria, and the sources and methods of selection of participants yes p4
Variables	7	Clearly define all outcomes, exposures, predictors, potential confounders, and effect modifiers. Give diagnostic criteria, if applicable yes box1, online appendix 1
Data sources/ measurement	8*	For each variable of interest, give sources of data and details of methods of assessment (measurement). Describe comparability of assessment methods if there is more than one group yes p5, online appendix 1
Bias	9	Describe any efforts to address potential sources of bias yes p4
Study size	10	Explain how the study size was arrived at n/a as is a pilot study.
Quantitative variables	11	Explain how quantitative variables were handled in the analyses. If applicable, describe which groupings were chosen and why yes p5, table2
Statistical methods	12	(*a*) Describe all statistical methods, including those used to control for confounding yes p5
		(*b*) Describe any methods used to examine subgroups and interactions, yes just chi2 tests p5
		(*c*) Explain how missing data were addressed all missing data is listed in the tables so it is completely transparent how this was dealt with, there were few missing data
		(*d*) If applicable, describe analytical methods taking account of sampling n/a
		(*e*) Describe any sensitivity analyses n/a
**Results**		
Participants	13*	(a) Report numbers of individuals at each stage of study—eg numbers potentially eligible, examined for eligibility, confirmed eligible, included in the study, completing follow-up, and analysed yes online appendix 1
		(b) Give reasons for non-participation at each stage yes online appendix 1
		(c) Consider use of a flow diagram yes online appendix 1
Descriptive data	14*	(a) Give characteristics of study participants (eg demographic, clinical, social) and information on exposures and potential confounders yes table 1
		(b) Indicate number of participants with missing data for each variable of interest yes all tables
Outcome data	15*	Report numbers of outcome events or summary measures yes all tables
Main results	16	(*a*) Give unadjusted estimates and, if applicable, confounder-adjusted estimates and their precision (eg, 95% confidence interval). Make clear which confounders were

For peer review only - http://bmjopen.bmj.com/site/about/guidelines.xhtml

		adjusted for and why they were included yes table 3
		(*b*) Report category boundaries when continuous variables were categorized yes all tables
		(*c*) If relevant, consider translating estimates of relative risk into absolute risk for a meaningful time period not appropriate as pilot study with self-selected sample
Other analyses	17	Report other analyses done—eg analyses of subgroups and interactions, and sensitivity analyses table 6 considers demographics for problems more likely to be a potentially harmful.
**Discussion**		
Key results	18	Summarise key results with reference to study objectives yes p7
Limitations	19	Discuss limitations of the study, taking into account sources of potential bias or imprecision. Discuss both direction and magnitude of any potential bias yes p8
Interpretation	20	Give a cautious overall interpretation of results considering objectives, limitations, multiplicity of analyses, results from similar studies, and other relevant evidence yes p7-8
Generalisability	21	Discuss the generalisability (external validity) of the study results yes p8, not generalisable
**Other information**		
Funding	22	Give the source of funding and the role of the funders for the present study and, if applicable, for the original study on which the present article is based yes p9

*Give information separately for exposed and unexposed groups.

Note: An Explanation and Elaboration article discusses each checklist item and gives methodological background and published examples of transparent reporting. The STROBE checklist is best used in conjunction with this article (freely available on the Web sites of PLoS Medicine at http://www.plosmedicine.org/, Annals of Internal Medicine at http://www.annals.org/, and Epidemiology at http://www.epidem.com/). Information on the STROBE Initiative is available at www.strobe-statement.org.

For peer review only

For peer review only - http://bmjopen.bmj.com/site/about/guidelines.xhtml

BMJ Open

Development and piloting of a survey to estimate the frequency and nature of potentially-harmful preventable-problems in primary care from a UK patient's perspective

Journal:	*BMJ Open*
Manuscript ID	bmjopen-2017-017786.R1
Article Type:	Research
Date Submitted by the Author:	03-Aug-2017
Complete List of Authors:	Stocks, Susan; University of Manchester, GM Patient Safety Translational Research Centre<br>Donnelly, Ailsa; University of Manchester, PRIMER<br>Esmail, Aneez; University of Manchester, School of Primary Care<br>Beresford, Joanne; Research User Group (RUG) of the NIHR Greater Manchester Primary Care Patient Safety Translational Research Centre<br>Gamble, Carolyn; University of Manchester, GM Patient Safety Translational Research Centre<br>Luty, Sarah; NHS Scotland<br>Deacon, Richard; St Gabriels Medical Centre<br>Danczak, Avril; Central and South Manchester Specialty Training Programme for General Practice<br>Mann, Nicola; University of Manchester, GMPSTRC<br>Townsend, David; University of Manchester, GM Patient Safety Translational Research Centre<br>Ashley, James; Division of Dentistry, School of Medical Sciences, University of Manchester<br>Bowie, Paul; NHS Scotland<br>Campbell, Stephen; University of Manchester, Centre for Primary Care
**Primary Subject Heading**:	Research methods
Secondary Subject Heading:	General practice / Family practice, Health services research, Communication, Dentistry and oral medicine
Keywords:	STATISTICS & RESEARCH METHODS, PRIMARY CARE, Quality in health care < HEALTH SERVICES ADMINISTRATION & MANAGEMENT

SCHOLARONE™
Manuscripts

Development and piloting of a survey to estimate the frequency and nature of potentially-harmful preventable-problems in primary care from a UK patient's perspective

S Jill Stocks *research fellow*[1], Ailsa Donnelly *public and patient involvement volunteer*[2], Aneez Esmail *professor*[1], Joanne Beresford *public and patient involvement volunteer*[2] Carolyn Gamble *public and patient involvement volunteer*[2], Sarah Luty *general practitioner*[3], Richard Deacon *general practitioner*[4], Avril Danczak *general practitioner*[5], Nicola Mann *general practitioner*[2], David Townsend *general practitioner*[2], James Ashley *dentist*[6], Paul Bowie *programme director*[7,8], Stephen M Campbell *professor*[1]

1. NIHR Greater Manchester Primary Care Patient Safety Translational Research Centre, Centre for Primary Care, Division of Population Health, Health Services Research and Primary Care, University of Manchester, Manchester M13 9PL, UK

2. Research User Group (RUG) of the NIHR Greater Manchester Primary Care Patient Safety Translational Research Centre, Centre for Primary Care, Division of Population Health, Health Services Research and Primary Care, University of Manchester, Manchester M13 9PL, UK

3. General Practitioner NHS Greater Glasgow and Clyde, Medical Directorate, NHS Education for Scotland, Glasgow G3 8BW

4. St Gabriels Medical Centre, 4, Bishops Road, Prestwich, Manchester M25 0HT, UK

5. Central and South Manchester Specialty Training Programme for General Practice, Health Education England North West (HEENWE) Education and Research Centre, Wythenshawe Hospital, Manchester M23 9LT, UK

6. Division of Dentistry, School of Medical Sciences, University of Manchester, Manchester M13 9PL, UK

7. Medical Directorate, NHS Education for Scotland, 2 Central Quay, Glasgow G3 8BW, UK.

8. Institute of Health and Wellbeing, University of Glasgow, Glasgow G12 0XH, UK

Correspondence to jill.stocks@manchester.ac.uk

Word count: 3541

For peer review only

For peer review only - http://bmjopen.bmj.com/site/about/guidelines.xhtml

Abstract

Objectives: To design and pilot a survey to be used at the population level to estimate the frequency of patient-perceived potentially-harmful preventable-problems occurring in UK primary care. To explore the nature of the problems, patient-suggested strategies for prevention and opinions of clinicians and the public regarding the potential for harm

Design: a survey was co-designed by three members of the public and one researcher and piloted through public and patient involvement and engagement networks

Setting: self-selected sample of the UK population

Participants: 977 members of the public accessed the online survey during October and November 2015

Primary outcome measures: respondent feedback about the ease of completion of the survey, quality of responses in terms of review by clinicians and members of the public, preliminary estimates of the frequency and nature of patient-perceived potentially-harmful problems occurring in the last 12 months

Results: 638 (65%) members of the public completed the survey and few respondents reported any difficulty in understanding or completing the survey. 132 (21%) respondents reported experiencing a potentially-harmful preventable-problem during the past 12 months and 108 (82%) of these respondents provided a description that was adequate for at least one clinician to form an opinion about the potentially-harmful problem. Respondents were older than the UK generally, more likely to work or volunteer in the healthcare sector and tended to use primary care more frequently but their confidence and trust in their own GP was similar to that of the UK population as measured by the annual English GP patient survey.

Conclusions: the survey was acceptable to patients and mostly provided data of sufficient quality for review by clinicians and members of the public. It is now ready to use at a population level to estimate the frequency and nature of potentially-harmful preventable-problems in primary care from a patient's perspective.

Strengths and limitations of this study

- We have designed and tested a survey to measure the frequency and nature of potentially-harmful preventable-problems in primary care from the patient's perspective
- The survey was co-designed by three members of the public and piloted through extensive public and patient involvement
- The patient-described scenarios were reviewed by primary care clinicians
- The study respondents were self-selected through public and patient involvement and engagement groups
- The survey is ready to be administered to a representative sample of the general population

For peer review only - http://bmjopen.bmj.com/site/about/guidelines.xhtml

Background

Patients are thought to take a different view of patient safety to healthcare professionals. (1) They tend to view safety in terms of the overall balance of benefit and harm over time whereas healthcare professionals often see high quality healthcare occasionally punctuated by safety incidents and adverse events.(2) Furthermore patients hold may different opinions about how to improve patient safety (3, 4) or different priorities to clinicians, for example identifying psychological and emotional harm rather than technical errors.(5) Involving patients in identifying errors and reducing harm occurs in secondary care (6) but patient reported outcomes can show poor concordance between patients and clinicians, for example, in reporting adverse symptom events in the context of drug safety.(7) Nonetheless patients are thought to be capable of reporting medical errors accurately. (6, 8) Involving patients is advocated as a way to improve safety (9) and this approach would be facilitated through patients and professionals having an understanding each other's expectations and priorities.

Studies that quantify patient safety problems in primary care are uncommon and incidence estimates from record review or incident reporting by clinicians range from less than 1 to 24 per 100 consultations or record review.(10-12) The National Reporting and Learning System (NRLS) in England and Wales records patient safety incidents reported by healthcare professionals; only 1% of these reports originate from primary care (13) which likely reflects under-reporting.(14, 15) Still fewer studies have quantified patient safety problems in primary care from the patient's perspective. (16) A 2013 European survey of the UK public reported that 43% of respondents felt that it was "likely" that patients could be harmed by non-hospital healthcare, an increase from 37% in 2009.(17) In Norway a population-level survey found that the patient-reported lifetime probability of ever experiencing an adverse event was 10%, of which around two thirds of respondents attributed the cause of their event as their general practitioner (GP).(4) In Spain a telephone survey of patients estimated that around 7% of patients experienced a self-reported adverse event during a 1 year period. (18) A USA practice-based website observed an incidence rate of patient-reported adverse events of 1.4% over 2 years.(19) Data from the UK is sparse; this may be partly due to the lack of a valid and reliable instrument to make a comprehensive measurement of safety in primary care.(20) The PREOS-PC should help to address this knowledge gap.(21, 22)

We aimed to design and pilot a survey to be used at the population level to estimate the frequency of patient-perceived potentially-harmful preventable-problems occurring in UK primary care. We relied on public and patient involvement (PPI) provided by the Greater Manchester Primary Care Patient Safety Translational Research Centre Research User Group (GMPSTRC RUG, 23) from the outset in order to ensure that our survey was easily understood by the public. We also aimed to explore the nature of the problems, patient-suggested strategies for prevention and differences in opinion between primary care clinicians and the public regarding the potential for harm in the patient-described scenarios. The study was conceived, designed and implemented by a team of three members of the public and one researcher. Primary care professionals provided their opinions after collection of the data. The specific aims of the study were to:

1. co-design with PPI partners and pilot a survey asking about problems occurring in primary care that caused, or had the potential to cause, preventable harm as perceived by patients

For peer review only - http://bmjopen.bmj.com/site/about/guidelines.xhtml

2. examine the potential of the survey to describe the type of patient-perceived problems reported, describe the demographics of the patients reporting a problem, the primary care service involved, with whom in the primary care service the problem was discussed (if it was) and patient suggestions as to how it might have been prevented.
3. examine the potential of the survey to compare the opinions of the reporting patient, members of the public and clinicians as to the likelihood the scenario describes a potentially-harmful preventable-problem.

Methods

Designing and piloting of the survey

Our aim was to design a survey asking about problems occurring in primary care that caused, or had the potential to cause, preventable harm as perceived by patients that was easily understood and free from jargon. Currently there is no well-established terminology for asking such a question.(8) The process began with a discussion between three members of the GMPSTRC RUG (AD, JB, CG) and one academic researcher (SJS). Questions used in previous surveys addressing a similar question (4, 17-19) were shared among the project team and used to generate several candidate questions. These questions were then discussed privately among the project team's friends and family and within the project team (SJS, AD, JB, CG). The discussion was facilitated by making the candidate questions available online. After two iterations of this process the survey (Box 1 & Box A online Appendix 1) was piloted online through newsletters or group mailings of several PPI and public engagement networks during November and December 2015. These networks were the associate GMPSTRC RUG, the Public Programmes team at Central Manchester Foundation Trust, the Citizen Scientist project, the Primary Care Research in Manchester Engagement Resource, North West People in Research Forum and HelpBeatDiabetes volunteers (Details of these groups and networks are provided in Box B, online Appendix 1).

The first question (Q1 Box 1) was taken from the English GP patient survey in order to compare the overall level of confidence and trust in their GP among the survey respondents with that across England.(24) The second question (Q2 Box 1) is the main screening question, those responding negatively to Q2 (*i.e.* not experienced a preventable-problem) were directed to a more specific question with a list of commonly understood patient safety events (Q10 Box A, online Appendix 1). If this prompted recognition of experiencing a potentially-harmful preventable-problem they were returned to Q4 (Box1). The rationale behind this approach was that the screening question (Q2 Box 1) should be non-leading and encourage the respondents to describe their preventable-problems through the subsequent questions without the suggestion that inevitably occurs following a list of possible potentially-harmful preventable-problems. However if the respondent did not believe that they had experienced a potentially-harmful preventable-problem then the prompt question (Q10, Box 1) would ensure that this was the case and also test the sensitivity of Q2 (Box 1). The option to answer on behalf of a friend or relative was offered to those who had not a personal experience to report. This was to ensure sufficient responses to adequately test the questionnaire but also to discourage respondents from answering with another person's experience as their own. Respondents were also asked whether they worked or volunteered in the healthcare profession and to comment on the ease of completion of the questionnaire.

For peer review only - http://bmjopen.bmj.com/site/about/guidelines.xhtml

Coding of reported events

Type of problem

The nature of the problem in each described scenario was coded at face value, *i.e.* as the patient described without further interpretation, by one author (SJS) and checked by a second author (JA for dental scenarios, PB for all other scenarios). A bottom-up (inductive) approach was used to identify similar topics which were coded then cross-matched to an existing taxonomy for errors in general practice (25, 26) (Table A, online Appendix 1). All the new codes matched the existing taxonomy within the higher two levels and the medication-related scenarios were coded to a finer level (Table B, online Appendix 1).

Likelihood the scenario described a potentially-harmful preventable-problem

Five GPs, one dentist and seven members of the public estimated the likelihood that, in their opinion, each patient-described scenario was a potentially-harmful preventable-problem. Brief biographies of the coders are provided in Table C, online Appendix 1. Some examples of the information provided to the coders are shown in boxes 1-23 in online Appendix 2 and consisted of the responses to Q5 to Q9 (Box 1). They were not given any demographic information or the patient's estimate of the impact on their health (Q4, Box 1). Coders were asked to score each scenario from very likely (5) to definitely not (1) in response to the question "*How likely do you think it is the patient was correct in thinking that their health might be worsened, or actually was made worse, because of a mistake or a problem in primary care that could have been prevented?*" Coders could also respond "insufficient information", "Don't know" and give free text feedback (Table D, Appendix 1). The clinician scores were used to categorise the scenarios in to groups with higher or lower estimated likelihoods that they were a potentially-harmful preventable-problem as below.

- Higher threshold - Median score of 5 ("very likely or certain") or 4 ("probably") or at least one score of 5 ("very likely or certain")
- Lower threshold - Median score of 3 ("possibly") or at least one score of 4 ("probably" or higher)
- All other scenarios – Median score below 3 ("possibly") and zero scores above 3 ("possibly")

Statistical analysis

Simple cross tabulations were used to describe the data and a binary logistic regression model was used to explore whether particular types of patient were more likely to perceive a potentially-harmful preventable-problems *e.g.* by demographics or their opinions. Comparisons between demographics and outcomes for the respondents and the UK (or England) population were made using a χ^2 test. All analyses were done using Stata 14.

Public and Patient Involvement (PPI)

PPI was central to this co-design study and was provided through the GMPSTRC RUG (23) and other PPI networks (Box C, online Appendix 1). The study was conceived, designed, implemented and analysed by a team of three members of the public (AD, CG, JB) and one researcher (SJS). At the outset the researcher presented the existing literature on this topic to the PPI members of the research team who then co-designed the first draft of the survey which was tested through the PPI members' personal contacts. The piloting of the survey was through existing PPI networks as listed in

For peer review only - http://bmjopen.bmj.com/site/about/guidelines.xhtml

Box B, online Appendix 1. The scoring of the questions as to the likelihood they described a potentially-harmful preventable-problem was undertaken by 7 members of the public, 2 of whom had no previous experience in PPI (as well as 5 GPs and 1 dentist as described in Table C, online Appendix 1). These findings will be disseminated to all the PPI groups that contributed to the pilot study and the authors will forward these results to their personal contacts who contributed to the questionnaire design.

Ethical approval was granted by the University of Manchester Ethics Committee 2 (Approval 15372).

Results

In total 977 members of the public accessed the online pilot survey and 638 (65%) completed the survey during October and November 2015. Flow charts of the participants through the survey are shown in Figures A&B, online Appendix 1. In total 223/638 (35%) of respondents reported ever experiencing a potentially-harmful preventable-problem in primary care of which 132 occurred within the past 12 months (21%, Fig A, online Appendix 1) and 62 (10%) of these problems were not identified through the initial screening question (Q2) but required prompting through Q10 (Box 1), see Fig A, online Appendix 1. A further 18 potentially-harmful preventable-problems involving friends or relatives where the respondent was present and occurred in the last 12 months were reported 13/418 (3%, Fig B, online Appendix 1). The majority of respondents (592, 93%) had confidence and trust in the GP seen at their last appointment similar to the 2016 England proportion of 92% (Q1, Box1 &Table 1). Demographic information was not provided by 83 (13%) respondents, possibly due to lack of clarity about the end of the survey since they completed all other questions. Respondents were older than the UK generally, more likely to work or volunteer in the healthcare sector and tended to use primary care more frequently (Table 1). Older respondents and those working or volunteering in the healthcare sector were no more likely to report a potentially-harmful preventable-problem occurring within the last 12 months but those using primary care more frequently were more likely to report a problem (Table 2).

The majority of respondents were recruited through the HelpBeatDiabetes group (533, 84%, Box B in online Appendix 1). Over 250 respondents provided free text feedback on the survey, 200 comments reported that the questionnaire was easy to complete and understand and just one comment described the survey as complex. Most of the remaining comments expressed the desire to be able to provide more information, *e.g.* more than one event or report for a relative or as a carer (reporting on behalf of another person was excluded for events occurring more than 12 months ago) and 13 comments actually provided this unrequested information. A few respondents found it difficult to find a suitable option to describe their pattern of use of primary care or their role as a worker or volunteer in healthcare.

The high completion rate and positive free text feedback suggested that respondents found the questionnaire easy to complete. Furthermore nobody used the “Do not understand the question” option as their response to Q2 Box1. There was a high response from healthcare professionals or volunteers (30% of respondents compared to approximately 3% of the UK adult population, Table 1) but they were no more likely to report a preventable problem than non-healthcare workers/volunteers (35%, $P\chi^2$=0.28). However the scenarios described by healthcare professionals or volunteers were significantly more likely to be categorised as a potentially-harmful preventable-

For peer review only - http://bmjopen.bmj.com/site/about/guidelines.xhtml

problem following to clinician review using both the lower (9% vs 16%, $P\chi^2$=0.01) and higher threshold (2% vs 6%, $P\chi^2$=0.004).

Likelihood the scenario described a potentially-harmful preventable-problem

Generally the members of the public assigned a higher probability to the likelihood that the patient-described scenario was a potentially-harmful preventable-problem compared with GPs (Fig 1, Table 3). In 89/108 (82%) scenarios the median score for the PPI researchers was higher than for the clinicians and for 38 (35%) scenarios the PPI median score was 2 or more points higher in a 5 point scale. Following clinician review 3% of the patient-reported scenarios occurring in the last 12 months were categorised as "probably" a potentially-unsafe preventable-problem and 11% as "possibly" (Table 3). Examples of the patient-reported scenarios with higher clinician rankings are shown in boxes 1-15, online Appendix 2 and those with greatest disagreement between members of the public and clinicians in boxes 16 to 23, online Appendix 2.

The nature of the potentially-harmful preventable-problems

The types of patient-reported scenarios and their categorisation following clinician review are shown in Figure 2. Medication-related problems were most frequently reported type of problem and also were ranked as more likely to be a potentially-harmful problem by clinicians. The type of scenario categorised according to whether it arose from the open-ended screening question (Q2) or prompted through the list of potential problems (Q10) is shown in Figures C&D, online Appendix 1. Information about the patient's response to the potentially-harmful preventable-problem and the primary care service involved is provided in Table 4. The majority of potentially-harmful preventable-problems in the past 12 months occurred in general practice (73%, Table 4) and pharmacy (5%, Table 4). Around half the respondents had not discussed their problem with anybody working in primary care (51%, Table 4). The most common reasons for not discussing the problem were being unable to find a primary care professional with whom to discuss the problem (31%, Table 4) or they did not feel comfortable with discussing their concerns (24%, Table 4) The patient suggestions for ways to prevent the problem from happening are summarised in Table 5. The most frequently occurring suggestions were that clinicians should involve the patient more fully in the healthcare process (*i.e.* listen to the patient and trust their judgement more) and be up to date with, and apply, the most recent information about the patient's condition (*i.e.* take in to account all of the patient's information - their medical history and results and letters).

Discussion

We have designed and tested a survey to measure the frequency of occurrence of potentially-harmful preventable-problems in primary care and found it to be well understood by patients. The survey is acceptable to patients based on the high completion rate and positive feedback. Furthermore none of the respondents indicated that they did not understand the screening question (Q2, Box1). The open-ended questions (Q6 to Q9, Box 1) led to patient-described scenarios that mapped well to an existing taxonomy designed and used by clinicians and researchers (Tables A&B, online Appendix 1, 25, 26). This implies agreement between clinicians, researchers and patients in identifying the characteristics of a potentially-harmful problem. Furthermore, the use of an open-ended screening question (Q2, Box 1) to ensure that any problems unique to the patient perspective were identified did not find additional new types of problem. The open-ended question elicited

For peer review only - http://bmjopen.bmj.com/site/about/guidelines.xhtml

more problems related to communication and medication suggesting that the public are more likely to view these as safety problems than problems related to appointments and referrals or investigations (Fig C&D online Appendix 1). We also observed that members of the public were more likely to rank the scenarios as a potentially-harmful preventable-problem than clinicians (Fig 1).

Strengths and weaknesses of the study

We believe that our survey captures the true patient perspective due to the involvement of members of the public as research partners through data acquisition to analysis and reporting in a co-designed study. By the use of a simple non-leading screening question we encouraged respondents to express their own perspective on what constituted a potentially-harmful preventable-problem rather than directing them towards existing definitions. To ensure that we did not miss any problems we followed up with a prompt that encouraged respondents to think in terms of the traditional view of patient safety problems. Furthermore our survey goes further than describing and counting the frequency of occurrence of potentially-harmful preventable-problems and provides information about how patients dealt with the problem and how it could have been prevented that offers insight in to ways to reduce the frequency of their occurrence. The absence of a link between practices and the patients allows for responses that might not occur if this survey were administered through the individual's practice. The main weakness of the study is the self-selection of the respondents who were older and tended to use primary care more frequently. More frequent users of primary care were more likely to report a problem but age was not associated with the likelihood of reporting a problem. Our bench marking question (Q1, Box1) showed that the respondents were similar to the English GP patient survey (24) in terms of their level of confidence and trust in their GP and not a group with a more negative attitude towards primary care as might have happened given the nature of the survey.

Strengths and weaknesses in relation to other studies

Our finding that 35% of respondents perceived that they had experienced a potentially-harmful problem in in their lifetime is consistent with a European survey (43% of UK respondents felt that it was "likely" that patients could be harmed by non-hospital healthcare).(17) This study offers some insight in to the type of concerns that might underlie this apparent lack of confidence in primary care. A face to face interview in family practice waiting rooms in the USA reported that 16% of respondents believed a physician had made a mistake in their care.(27) The types of problem and patient responses to the problem are similar to those that have been described qualitatively (1, 22) but we have taken this a step further by quantifying their frequency of occurrence and other descriptors of the problem from the patient's perspective. In this small study we did not find that patients were particularly likely to attribute blame to individual members of staff as has been observed previously (3, 4), perhaps partly due to the high proportion of respondents working or volunteering in healthcare.

Unanswered questions and future research

Our finding that 21% of respondents perceived that they had experienced a potentially-harmful problem in the last 12 months, and the corresponding proportion following clinician review of 3% (higher threshold) to 11% (lower threshold) may well reflect the self-selected nature of the study

For peer review only - http://bmjopen.bmj.com/site/about/guidelines.xhtml

population and needs to be validated in a large population level survey. We anticipate that a population level survey would be fruitful since this approach yielded a number of patient-described scenarios that were amenable to further analysis including coding by clinicians. The high response to this pilot survey by healthcare professionals coupled with the likelihood that they provided better information, given the higher ranking given by clinicians to scenarios originating from healthcare professionals, points towards an opportunity. Healthcare professionals are an educated and accessible group who could provide a valuable resource for learning about preventable-problems in primary care. This survey could be used to ask NHS staff anonymously about their *personal experiences, as a patient,* of potentially-harmful preventable-problems in primary care. Of course complete anonymity for responders would need to be guaranteed and any identifying aspects in their reported scenarios would have to be removed. It would be very different to whistle-blowing, respondents would feed back on the care they received personally rather make observations on their own colleagues practice. The aim would be to anonymously describe and monitor problems over time through individuals with the expectations of a patient who also had an understanding of the healthcare system.

Acknowledgements: The authors would like to express their thanks and appreciation for the work done by the Mary Aldred, Gitanjali Holt, Manoj Mistry, Carole Bennett and Lindsey Brown in coding the patient-described scenarios.

This work was supported by the NIHR grant number gmpstrc-2012-1

Funding: This study was funded by the National Institute for Health Research (http://www.nihr.ac.uk) through the Greater Manchester Primary Care Patient Safety Translational Research Centre (NIHR GM PSTRC), grant number gmpstrc-2012-1. The views expressed are those of the author(s) and not necessarily those of the NHS, the NIHR or the Department of Health. The funders had no role in study design, data collection and analysis, decision to publish, or preparation of the manuscript.

Competing interests: All authors have completed the ICMJE uniform disclosure form at www.icmje.org/coi_disclosure.pdf (available on request from the corresponding author) and declare no support from any organisation for the submitted work; no financial relationships with any organisations that might have an interest in the submitted work in the previous three years, no other relationships or activities that could appear to have influenced the submitted work.

Copyright statement: the Corresponding Author has the right to grant on behalf of all authors and does grant on behalf of all authors, an exclusive licence on a worldwide basis to the BMJ Publishing Group Ltd to permit this article (if accepted) to be published in BMJ editions and any other BMJPGL products and sublicences such use and exploit all subsidiary rights, as set out in our licence.

Contributors: SJS, AD, JB and CG conceived and designed the study. SJS, AD, JB, CG, AE, PB, JA, DT, SL, AD, RD and NM analysed the data. SJS wrote the manuscript, and is guarantor. AD, JB, CG, AE, PB, JA, DT, SL, AD, RD, NM and SC edited the manuscript.

Data sharing: Raw data (coded only) is available from jill.stocks@manchester.ac.uk

Ethics approval: University of Manchester Ethics Committee 2 (Approval 15372)

For peer review only - http://bmjopen.bmj.com/site/about/guidelines.xhtml

Figure legends

Figure 1. Median estimates as to the likelihood that the patient describes a potentially-harmful preventable-problem occurring in the last 12 months by six clinicians and seven members of the public

Footnote to Figure 2: See Tables A&B, online Appendix 1 for details of coding; A coded to 2 levels, B medication problems coded to 3 levels, C coded to 1 level

Fig 2. Numbers of patient-perceived problems occurring in the last 12 months categorised according to the patient's description with clinician ranking as to the likelihood it is a potentially-harmful preventable problem (Table 3).

For peer review only

Box 1. Brief summary of questionnaire – see Box A, online Appendix 1 for full version of survey.

Q1. Did you have confidence and trust in the GP you saw or spoke to at your last appointment? (benchmarking question)

Q2. When using primary care have you ever felt concerned that your health might be worsened, or actually was made worse, because of a mistake or a problem that could have been prevented?

If yes to Q2

Q3. How long ago did the mistake or preventable problem happen?

Q4. How did this affect your health?

Q5. Which primary care service were you using when the mistake or preventable problem occurred?

Q6. Briefly describe the mistake or problem and how it happened

Q7. Could the mistake or problem have been avoided? If so how?

Q8. Were you able to talk about the mistake or problem with anybody working in the primary care service? If not –why not?

Q9. If you discussed the mistake or problem with somebody working in primary care please describe their job or role

Q10. In the list below are some examples of **preventable** problems[1] that might happen when using primary care. Has ***anything similar*** happened to you ***in the last 12 months***? *If yes go to Q4*

[1]See Q10 Box A, online Appendix 1 for list of preventable problems

For peer review only

For peer review only - http://bmjopen.bmj.com/site/about/guidelines.xhtml

Table 1. Characteristics of survey respondents

Variable	All respondents n=638	Ever had problem n=223	Had problem in last 12 months n=132	UK population comparator
GP satisfaction	missing=0	missing=0	missing=0	English GP patient survey(24)
Yes definitely	384 (60%)	81 (36%)	55 (42%)	64%
Yes, to some extent	208 (33%)	110 (49%)	52 (39%)	28%
No, not at all	39 (6%)	27 (12%)	21 (16%)	4%
Don't know / can't say	7 (1%)	5 (2%)	4 (3%)	3%
Worked or volunteered in healthcare	missing=92	missing=40	missing=19	NHS workforce[1]
Yes	166 (30%)	64 (35%)	41 (36%)	3%
Gender	missing=87	missing=38	missing=16	ONS mid-2015 estimates[2]
Female	268 (49%)	106 (57%)	63 (54%)	51%
Age	missing=85	missing=37	missing=15	ONS mid-2015 estimates[2]
16 to 34 years	42 (8%)	22 (12%)	11 (9%)	31%
35 to 54 years	143 (26%)	54 (29%)	34 (29%)	34%
55 to 64 years	162 (29%)	59 (32%)	31 (27%)	14%
65 to 74 years	170 (31%)	44 (24%)	32 (27%)	12%
Over 75 years	36 (7%)	7 (4%)	9 (8%)	9%
Last primary care contact	missing=88	missing=39	missing=14	English GP patient survey(24)
Within last week	169 (31%)	65 (35%)	48 (41%)	84% within last 12 months
Within last month	248 (45%)	79 (43%)	47 (40%)	
Within last 12 months	121 (22%)	34 (18%)	20 (17%)	
Over 12 months ago	12 (2%)	6 (3%)	3 (3%)	15%
Usual primary care usage	missing=88	missing=40	missing=17	
At least once a month	181 (33%)	73 (40%)	52 (45%)	-
At least once per 6 months	285 (52%)	79 (43%)	45 (39%)	-
Once per 12 months or less	84 (15%)	31 (17%)	18 (16%)	-

[1]http://content.digital.nhs.uk/searchcatalogue?productid=24139&topics=1%2fWorkforce%2fStaff+numbers&sort=Relevance&size=10&page=1#top

[2]https://www.ons.gov.uk/peoplepopulationandcommunity/populationandmigration/populationestimates/bulletins/annualmidyearpopulationestimates/latest

For peer review only

For peer review only - http://bmjopen.bmj.com/site/about/guidelines.xhtml

1 Table 2. Prevalence of respondents reporting a potentially-harmful preventable problem within the
2 last 12 months and unadjusted and adjusted odds ratios estimated by logistic regression

Respondent characteristics n=638	Frequency – all reported n=132	Unadjusted odds ratio (OR) – all reports	Adjusted[1] OR - all reports	Adjusted[1] OR - after GP review (lower threshold, Table 3)
**Gender (87 missing)**				
male	53/283 (19%)	1 (ref)	1 (ref)	1 (ref)
female	63/268 (24%)	1.3 (0.9 to 2.0)	1.4 (0.9 to 2.2)	1.3 (0.7 to 2.3)
**Age (85 missing)**				
16 to 34 years	11/42 (26%)	1 (ref)	1 (ref)	1 (ref)
35 to 54 years	34/143 (24%)	0.9 (0.4 to 1.9)	0.8 (0.3 to 1.8)	0.8 (0.3 to 2.1)
55 to 64 years	31/162 (19%)	0.7 (0.3 to 1.5)	0.7 (0.3 to 1.5)	0.6 (0.2 to 1.7)
65 to 74 years	32/170 (19%)	0.7 (0.3 to 1.4)	0.6 (0.3 to 1.4)	0.4 (0.2 to 1.2)
Over 75 years	9/36 (25%)	0.9 (0.3 to 2.6)	1.1 (0.4 to 3.2)	0.9 (0.2 to 3.2)
**Last primary care contact (88 missing)**				
Within last week	48/169 (28%)	1 (ref)	1 (ref)	1 (ref)
Within last month	47/248 (19%)	0.6 (0.4 to 0.9)	0.7 (0.4 to 1.1)	0.6 (0.3 to 1.0)
Within last 12 months	20/121 (17%)	0.5 (0.3 to 0.9)	0.6 (0.3 to 1.2)	0.5 (0.2 to 1.3)
Over 12 months ago	3/12 (25%)	0.8 (0.2 to 4.0)	0.9 (0.2 to 4.2)	0.4 (0.0 to 3.9)
**Usual primary care usage (88 missing)**				
At least once a month	52/181 (29%)	1 (ref)	1 (ref)	1 (ref)
At least once per 6 months	45/285 (16%)	0.5 (0.3 to 0.7)	0.6 (0.3 to 0.9)	0.5 (0.3 to 0.9)
Once per 12 months or less	18/84 (21%)	0.7 (0.4 to 1.2)	0.8 (0.4 to 1.6)	0.7 (0.3 to 1.8)
**Works or volunteers in healthcare (92 missing)**				
No	72/380 (19%)	1 (ref)	1 (ref)	1 (ref)
Yes	41/166 (25%)	1.4 (0.9 to 2.2)	1.3 (0.8 to 2.1)	1.5 (0.9 to 2.7)

3

4 [1]adjusted for gender, age, last primary care contact, usual primary care usage, works or volunteers in healthcare

For peer review only - http://bmjopen.bmj.com/site/about/guidelines.xhtml

1 Table 3. Categorisation of patient-perceived potentially-harmful preventable problems occurring in
2 the last 12 months following review by clinicians and members of the public

Group label	Threshold criteria	Clinician scores n=132	Members of the public scores n=132
1. Higher threshold	Median score of “very likely or certain” or “probably” or at least one score of “very likely or certain”	18 (14%)	87 (66%)
2. Lower threshold	Median score of “possibly” or at least one score of “probably” or higher	71 (54%)	104(79%)
3. Any possibility	At least one score of “unlikely” or higher	106 (80%)	109 (83%)
4. No problem	All scores “definitely not” or not-coded	1 (1%)	0
5. Not-coded	Insufficient information for coding by all coders	25 (19%)	23 (17%)

3

4

For peer review only

Table 4. The patient's response to their perceived potentially-harmful preventable-problem and the primary care service involved for problems occurring in the last 12 months

Primary care service	All reported problems	Clinician ranked "possibly or higher" (Lower threshold)
All services	132	71
GP surgery	97 (73%)	61 (86%)
Out of hours care/A&E/ambulance	4 (3%)	1 (1%)
Walk in clinic	2 (2%)	0
Dental surgery	4 (3%)	1 (1%)
Pharmacy	7 (5%)	6 (8%)
Community or district nursing	4 (3%)	0
Opticians	2 (2%)	1 (1%)
Mental health services	1 (1%)	0
missing	11 (8%)	1 (1%)
**Did you discuss the problem with primary care staff?**		
All respondents	132	71
Yes – discussed with primary care staff	56 (42%)	42 (59%)
No – did not discuss with primary care staff	67 (51%)	29 (41%)
missing	9 (7%)	0
**Reason not discussed with primary care staff**		
All not discussing problem	67	29
Did not feel comfortable to discuss the problem	16 (24%)	8 (28%)
Could not find anybody with whom to discuss the problem	21 (31%)	10 (34%)
Unconcerned about the problem	7 (10%)	5 (17%)
Did not notice the problem at the time (or too ill)	11 (16%)	4 (14%)
Other	5 (7%)	2 (7%)
missing	7 (10%)	0
**Profession of discussant**		
All discussing problem	56	42
GP	28 (50%)	19 (45%)
Practice manager	5 (9%)	5 (21%)
Receptionist	2 (4%)	1 (2%)
Practice nurse	6 (11%)	5 (12%)
Pharmacist or dispenser	7 (13%)	7 (17%)
Dentist	2 (4%)	1 (2%)
Dietician	1 (2%)	1 (2%)
Missing	5 (9%)	3 (7%)
**Role of discussant in patient's care**		
Member of staff directly involved	23 (41%)	16 (38%)
Another member of staff at same institution	25 (45%)	20 (48%)
Above unclear	8 (14%)	6 (14%)

For peer review only - http://bmjopen.bmj.com/site/about/guidelines.xhtml

Table 5. Patient suggestions as to how the potentially-harmful preventable problem might have been prevented

How could it be prevented?	All reported problems n=132	Clinician ranked "possibly or higher" (Lower threshold) n=71
**1. More resources - all**	**14 (11%)**	**3 (4%)**
1.1 Quicker access to primary care	7 (5%)	2 (3%)
1.2 More thorough and quicker investigations	2 (2%)	1 (1%)
1.3 Fewer demands on primary care – more staff or fewer patients	1 (1%)	0
1.4 More time with clinicians for treatment and diagnosis	2 (2%)	0
1.9 Provision of resources to manage long term conditions	1 (1%)	0
1.10 Provision of patient travel service for routine appointments	1 (1%)	0
**2. Improved communication and involvement of patients**	**26 (20%)**	**18 (25%)**
1.1 Listen to the patient and trust their judgement more	21 (16%)	15 (21%)
1.2 Tell patients about their diagnosis, test results, changes in medication or loss of results	3 (2%)	1 (1%)
1.3 Improve communication between staff (within or outside primary care)	2 (2%)	2 (3%)
**3. Better organisation and administration**	**17 (13%)**	**10 (14%)**
3.1 Follow up referrals and appointments to ensure they happen, be consistent in sending routine reminders	10 (8%)	3 (4%)
3.2 Log in or process results as soon as received to avoid loss	1 (1%)	1 (1%)
3.3 Keep the notes up to date, well-organised, safe and ensure information is transcribed accurately	5 (4%)	5 (7%)
3.4 Keep a record of the location of equipment	1 (1%)	1 (1%)
**4. Improved prescribing systems**	**18 (14%)**	**17 (24%)**
4.1 More checks on prescribing and dispensing	8 (6%)	8 (11%)
4.2 Check repeat prescriptions carefully, especially for transcribing errors	8 (6%)	7 (10%)
4.3 Use medication reviews and IT clinical decision support systems	2 (2%)	2 (3%)
**5. Better clinical practice**	**19 (14%)**	**10 (14%)**
5.1 Take in to account all the patient's information - their medical history and results and letters	13 (10%)	7 (10%)
5.2 Address the patient's problem in some way – patients can feel their problem is being ignored	5 (4%)	2 (3%)
5.3 Act on advice from other clinicians and test results	1 (1%)	1 (1%)
**6. Staff training**	**11 (8%)**	**7 (10%)**
6.1 More informed and better trained staff	11 (8%)	7 (10%)
**Other responses**	**27 (20%)**	**6 (8%)**
• Don't know/missing	21 (16%)	3 (4%)
• Problem was due to an individual member of staff	2 (2%)	1 (1%)
• Prescribe right, better, different, more, less medicine	1 (1%)	0
• Better organisation	1 (1%)	0
• Laboratory procedures were the problem	2 (2%)	2 (3%)

For peer review only

For peer review only - http://bmjopen.bmj.com/site/about/guidelines.xhtml

References

1. Rhodes P, Campbell S, Sanders C. Trust, temporality and systems: how do patients understand patient safety in primary care? A qualitative study. Health Expectations. 2016;19(2):253-63.

2. Vincent C, Amalberti R. Safer Healthcare: Strategies for the Real World. Springer, 2016. http://www.springer.com/gb/book/9783319255576 Accessed 04/04/17

3. Blendon RJ, DesRoches CM, Brodie M, Benson JM, Rosen AB, Schneider E, et al. Views of practicing physicians and the public on medical errors. N Engl J Med. 2002;347(24):1933-40.

4. Hotvedt R, Forde OH. Doctors are to blame for perceived medical adverse events. A cross sectional population study. The Tromso Study. BMC health services research. 2013;13:46.

5. Kuzel AJ, Woolf SH, Gilchrist VJ, Engel JD, LaVeist TA, Vincent C, et al. Patient reports of preventable problems and harms in primary health care. Ann Fam Med. 2004;2(4):333-40.

6. The Health Foundation. Evidence scan: Involving patients in improving safety. January 2013. http://www.health.org.uk/sites/health/files/InvolvingPatientsInImprovingSafety.pdf Accessed 04/04/17

7. Basch E. The missing voice of patients in drug-safety reporting. N Engl J Med. 2010;362(10):865-9.

8. King A, Daniels J, Lim J, Cochrane DD, Taylor A, Ansermino JM. Time to listen: a review of methods to solicit patient reports of adverse events. Qual Saf Health Care. 2010;19(2):148-57.

9. Longtin Y, Sax H, Leape LL, Sheridan SE, Donaldson L, Pittet D. Patient participation: current knowledge and applicability to patient safety. Mayo Clinic proceedings. 2010;85(1):53-62.

10. Sandars J, Esmail A. The frequency and nature of medical error in primary care: understanding the diversity across studies. Family practice. 2003;20(3):231-6.

11. Panesar SS, deSilva D, Carson-Stevens A, Cresswell KM, Salvilla SA, Slight SP, et al. How safe is primary care? A systematic review. BMJ Quality & Safety. 2016;25(7):544-53.

12. Michel P, Brami J, Chaneliere M, Kret M, Mosnier A, Dupie I, et al. Patient safety incidents are common in primary care: A national prospective active incident reporting survey. PLoS One. 2017;12(2):e0165455.

13. NHS Improvement. National quarterly data on patient safety incident reports. September 2016. https://improvement.nhs.uk/resources/national-quarterly-data-patient-safety-incident-reports-september-2016/ Accessed 04/04/17

14. Hutchinson A, Young TA, Cooper KL, McIntosh A, Karnon JD, Scobie S, et al. Trends in healthcare incident reporting and relationship to safety and quality data in acute hospitals: results from the National Reporting and Learning System. Qual Saf Health Care. 2009;18(1):5-10.

15. NHS England. General practice patient safety reporting form launched. 26th February 2015. https://www.england.nhs.uk/2015/02/gp-patient-safety-reporting/ Accessed 04/04/17

For peer review only - http://bmjopen.bmj.com/site/about/guidelines.xhtml

16. The Health Foundation. Evidence Scan: Improving safety in primary care. November 2011. http://www.health.org.uk/sites/health/files/ImprovingSafetyInPrimaryCare.pdf Accessed 04/04/17

17. European Commission. Special Eurobarometer 411 Patient Safety and Quality of Care 2014. DOI:10.2772/33467 https://ec.europa.eu/health/sites/health/files/patient_safety/docs/ebs_411_sum_en.pdf Accessed 04/04/17

18. Mira JJ, Nebot C, Lorenzo S, Perez-Jover V. Patient report on information given, consultation time and safety in primary care. Qual Saf Health Care. 2010;19(5):e33.

19. Wasson JH, MacKenzie TA, Hall M. Patients use an internet technology to report when things go wrong. Qual Saf Health Care. 2007;16(3):213-5.

20. Ricci-Cabello I, Goncalves DC, Rojas-Garcia A, Valderas JM. Measuring experiences and outcomes of patient safety in primary care: a systematic review of available instruments. Family practice. 2015;32(1):106-19.

21. Ricci-Cabello I, Avery AJ, Reeves D, Kadam UT, Valderas JM. Measuring Patient Safety in Primary Care: The Development and Validation of the "Patient Reported Experiences and Outcomes of Safety in Primary Care" (PREOS-PC). Ann Fam Med. 2016;14(3):253-61.

22. Ricci-Cabello I, Saletti-Cuesta L, Slight SP, Valderas JM. Identifying patient-centred recommendations for improving patient safety in General Practices in England: a qualitative content analysis of free-text responses using the Patient Reported Experiences and Outcomes of Safety in Primary Care (PREOS-PC) questionnaire. Health Expectations. 2017.

23. Stocks SJ, Giles SJ, Cheraghi-Sohi S, Campbell SM. Application of a tool for the evaluation of public and patient involvement in research. BMJ Open. 2015;5(3).

24. NHS England. GP Patient Survey – National summary report. January 2016. http://gp-survey-production.s3.amazonaws.com/archive/2016/January/January+2016+National+Summary+Report.pdf Accessed 04/04/17

25. Dovey SM, Meyers DS, Phillips RL, Jr., Green LA, Fryer GE, Galliher JM, et al. A preliminary taxonomy of medical errors in family practice. Qual Saf Health Care. 2002;11(3):233-8.

26. Makeham MA, Dovey SM, County M, Kidd MR. An international taxonomy for errors in general practice: a pilot study. Med J Aust. 2002;177(2):68-72.

27. Kistler CE, Walter LC, Mitchell C, Sloane PD. Patient perceptions of mistakes in ambulatory care. Archives of Internal Medicine. 2010;170(16):1480-7.

For peer review only

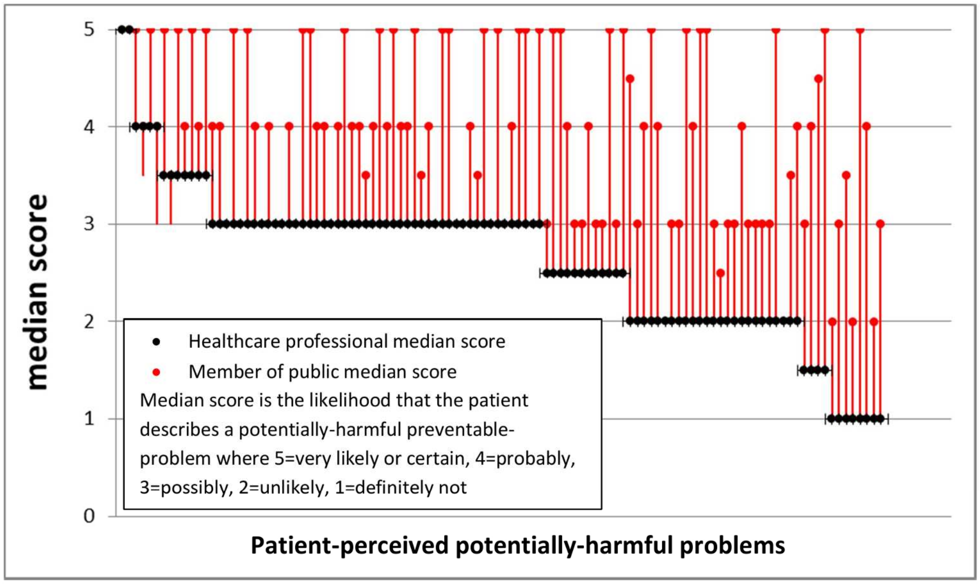

Figure 1. Median estimates as to the likelihood that the patient describes a potentially-harmful preventable-problem occurring in the last 12 months by six clinicians and seven members of the public

170x102mm (300 x 300 DPI)

review only

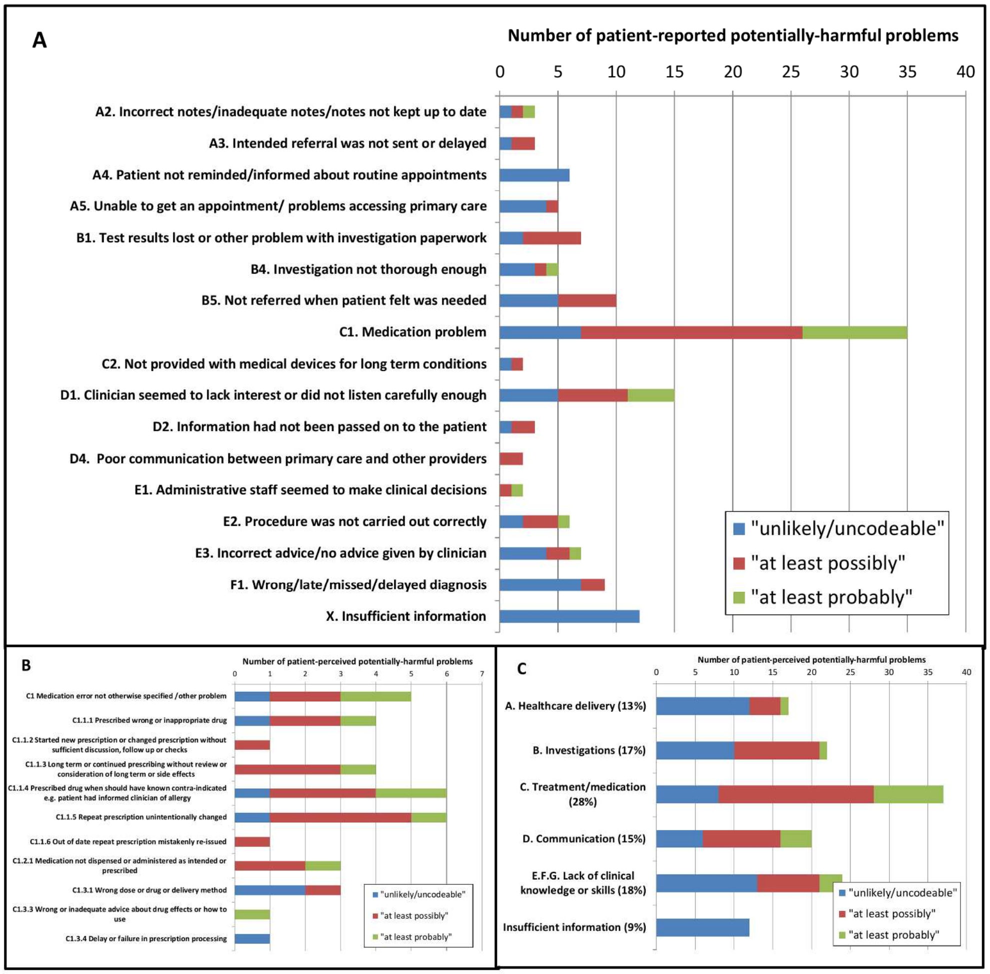

Footnote to Figure 2: See online Appendix 2 for details of coding; A coded to 2 levels, B medication problems coded to 3 levels, C coded to 1 level‼ ┼ Fig 2. Numbers of patient-perceived problems occurring in the last 12 months categorised according to the patient's description with clinician ranking as to the likelihood it is a potentially-harmful preventable problem (Table 3).‼ ┼

199x195mm (300 x 300 DPI)

Appendix 1. Details of survey, coding systems, public and clinician contributors and supplementary results

Box A. Pilot survey administered online November and December 2015

Q1. Did you have confidence and trust in the GP you saw or spoke to at your last appointment?

Response options: Yes, definitely, Yes, to some extent, No, not at all, Don't know / can't say

Q2. When using primary care have you ever felt concerned that your health might be worsened, or actually was made worse, because of a mistake or a problem that could have been prevented?

Response options: Yes, No- *go to Q10,* Do not understand the question- *go to Q10*, Don't know / can't remember- *go to Q10*

Q3. How long ago did the mistake or preventable problem happen?

Response options: Within the last 12 months, More than 12 months ago- *go to Q10,* Can't remember- *go to Q10*

Q4. In your opinion did this experience

Response options: Make your health worse, Not certain but it might have made your health worse, Could have made your health worse if you had not noticed the problem, Delayed your treatment but had no effect on your health, Not affect you, or your health, Other, please explain

Q5. Which primary care service were you using when the mistake or preventable problem occurred?

Response options: GP surgery, Out of hours care, Walk in clinic, Dental, Pharmacy, Community or district nursing, Ambulance, Opticians, Other- please specify

Q6. Briefly describe the mistake or problem and how it happened

Response options: free text

Q7. Could the mistake or problem have been avoided? If so how?

Response options: free text

Q8. Were you able to talk about the mistake or problem with anybody working in the primary care service?

Response options: Yes, Yes had the opportunity but did not feel comfortable to discuss the mistake or problem, No I could not find anybody with whom I could discuss the mistake or problem, No I was not concerned about the problem, No I did not notice the mistake or problem at the time, I was too distressed to discuss the mistake or problem, Other or don't know - please describe

Q9. If you discussed the mistake or problem with somebody working in primary care please describe their job or role

Response options: free text

Q10. In the list below are some examples of preventable problems that might happen when using primary care. Has anything similar happened to you in the last 12 months? Please check as many as applicable or "NONE OF BELOW"

NONE OF BELOW
Wrong or late diagnosis
Not referred for further investigation when needed
Test results being lost or mixed up
Receiving the wrong medicine or wrong dose
Should not be prescribed the medicine because of another health problem
Should not be prescribed the medicine because of another medication already taking
Poor communication leading to misunderstanding of diagnosis or treatment
Not referred to a specialist when needed

Unclear instructions about treatment<br>Not offering of prevention or screening programmes eg CVD/stroke prevention clinics<br>Failure to recognise or act on vulnerable people's needs eg child abuse, suicide risk or mental health problems<br>Mistake with a procedure eg dental treatment, injection, ear syringing, physiotherapy<br>Failure to notify about recommended vaccinations eg flu, HPV<br>Poor hygiene<br>Unsafe building or premises<br>Any other preventable problem in the last 12 months (in your opinion)<br>Other, please explain below
Q11. Are you male or female? Response options: Male, Female, prefer not to say
Q12. How old are you?<br>Response options: under 16, 16 to 24, 25 to 34, 45 to 54, 55 to 64, 65 to 74, 75 to 84, 85 or older
Q13. When was your last contact with primary care?<br>Response options: Last week, Last month, Last 12 months, Over 12 months ago
Q14. What best describes your usual pattern of use of primary care? Response options: Once per week, Once per 2 weeks, Once per month, Once per 6 months, Once per 12 months or less often
Q15. Are you registered with a GP practice?<br>Response options: Yes, No, I only use walk in centres, Don't know
Q16. Do you work or volunteer in healthcare or healthcare research as a professional, patient, carer or member of the public? (if you are retired answer for your occupation before retirement)<br>Response options: Yes, No
Q17. We are still trying to improve this questionnaire so would be grateful for any feedback about how easy you found the questionnaire to complete? How can it be improved?<br>Response options: free text

Box B. List of public and patient involvement groups used to distribute the pilot survey
Associate Research User Group of the Greater Manchester Primary Care Patient Safety Translational Research Centre http://research.bmh.manchester.ac.uk/primary-care-patient-safety/GetInvolved/
The Primary Care Research in Manchester Engagement Resource http://research.bmh.manchester.ac.uk/PRIMER/about/
HelpBeatDiabetes https://www.researchforthefuture.org/diabetes/
The Nowgen Centre https://research.cmft.nhs.uk/getting-involved/involvement
The Citizen Scientist project http://www.citizenscientist.org.uk/
North West People in Research Forum https://www.northwestpeopleinresearchforum.org/

Table A. Coding of patient-reported potentially-unsafe scenarios in primary care

**1. Errors in the process of the healthcare delivery system**	
Makeham 2002, Dovey 2002	Common threads reported in this study
1.1. Errors in the process of conducting an administrative task	A1. Administrative problem not otherwise specified
1.1.1. Information filed in wrong place or wrong time	
1.1.2. Unavailability of information that should have been in patients charts<br>1.1.2.1. Entire chart or part of chart could not be accessed when needed<br>1.1.2.2. Care provided was not documented<br>1.1.2.3. Item(s) of information missing from chart	A2. Incorrect notes/inadequate notes/notes not kept up to date
1.1.3. Errors in patient's movement through the healthcare delivery system	A3. Intended referral was not sent or delayed<br>A4. Patient not reminded, informed or assisted to attend regular check-ups or other necessary routine treatments
1.1.4. Errors in the taking and distributing of messages	
1.1.5. Errors in managing appointments for healthcare	A5. Unable to get an appointment/other problems with making appointment<br>A6. Ambulance delayed or did not arrive
**1.2. Errors in the process of investigating a patient's condition**	
1.2.1. Laboratory errors<br>1.2.1.1. Wrong test ordered or test not ordered when appropriate<br>1.2.1.2. Errors in the process of obtaining or processing a laboratory specimen<br>1.2.1.3. Error in the process of physician receiving accurate laboratory results in a timely fashion<br>1.2.1.4. Inappropriate response to an abnormal laboratory result<br>1.2.3. Errors in the processes of other investigations<br>1.2.3.1. Wrong test ordered or test not ordered when appropriate<br>1.2.3.2. Errors in the process of obtaining or processing of other diagnostic investigation<br>1.2.3.3. Error in the process of physician receiving accurate test results of other investigation in a timely fashion<br>1.2.3.4. Inappropriate response to an abnormal result of other investigation	B1. Test results lost or other problem with investigation paperwork<br>B2. Incorrect interpretation of tests or other investigation results<br>B3. Clinician did not consider patient history sufficiently/did not use patient's notes adequately<br>B4. Investigation not thorough enough<br>B5. Not referred when patient felt was needed
**1.3. Errors in the process of treating a patient's condition**	
1.3.1. Errors in the process of treating with medications<br>1.3.1.1. Wrong medication or wrong dose of medication ordered or medication not ordered by physician when appropriate<br>1.3.1.2. Error in the process of delivering a medication order or inappropriate medication order by a provider working under physician supervision<br>1.3.1.3. Error in the process of dispensing medication as ordered	C1. Medication problem<br>C2. Not provided with medical devices needed to manage long term conditions

For peer review only

1.3.2. Errors in other treatments	C3. Problem with dental treatment or diagnosis
**1.4. Errors in the process of communication**	
1.4.1. Errors in communication between primary healthcare provider and patients	D1. Clinician seemed to lack interest in the patient's health problem or did not listen carefully enough<br>D2. Information about the patient's health had not been passed on to the patient who felt it should have been<br>D3. Communication problem between patient and primary care staff
1.4.2. Errors in communication between healthcare providers	D4. Problem with communication between primary care and other types of care including secondary care<br>D5. Disagreement between 2 clinicians
**2. Errors arising from lack of clinical knowledge or skills**	
2.1. Errors in the execution of a clinical task<br>2.1.1. Non-clinical staff made the wrong clinical decision<br>2.1.2. Failed to follow standard practice<br>2.1.3. Lacked needed experience or expertise in a clinical task	E1. Administrative staff seemed to make clinical decisions<br>E2. Procedure was not carried out correctly<br>E3. Incorrect advice/no advice given by clinician
**2.2. Errors in diagnosis**<br>2.2.1. Wrong or delayed diagnosis	F1. Wrong/late/missed/delayed diagnosis
**2.3. Wrong treatment decision**	G1. Wrong treatment decision
	H. Other
	X. Not a problem/ insufficient information/refused/don't know

Table B. Level 4 coding of patient-reported potentially-unsafe medication scenarios

Common threads reported in this study grouped as described by Makeham 2002, Dovey 2002
C1 Medication error not otherwise specified /other problem
• **1.3.1.1. Ordering medications (prescribing)**
C1.1.1 Prescribed wrong or inappropriate drug
C1.1.2 Started new prescription or changed prescription without sufficient discussion, follow up or checks
C1.1.3 Long term or continued prescribing without review or consideration of long term or side effects
C1.1.4 Prescribed drug when should have known contra-indicated *e.g.* patient had informed clinician of allergy, adverse reaction or it was in the records
C1.1.5 Repeat prescription unintentionally changed
C1.1.6 Out of date repeat prescription mistakenly re-issued
• **1.3.1.2./1.3.1.3. Implementing or receiving medications (dispensing or issuing)**
C1.2.1 Medication not dispensed or administered as intended or prescribed
• **1.3.1.1/1.3.1.2./1.3.1.3. Ordering, implementing or receiving medications**
C1.3.1 Wrong dose or drug or delivery method
C1.3.2 Being given another patient's drugs or prescription
C1.3.3 Wrong or inadequate advice about drug effects or how to use
C1.3.4 Delay or failure in prescription processing

For peer review only

For peer review only - http://bmjopen.bmj.com/site/about/guidelines.xhtml

Table C. Demographics of clinicians and members of the public reviewing the patient-reported problems and estimating the likelihood the scenarios describes a potentially-harmful preventable-problem occurring in primary care

**Demographics of GP and dentist coders**	**frequency n=6**
**Gender**	
Female	3
Male	3
**Years working as a GP or dentist**	
Less than 15 years	1
15 to 25 years	2
Over 25 years	3
**Current position**	
Partner	4
Retired within last 12 months	2
**Demographics of the members of the public**	**frequency n=7**
**Gender**	
Female	6
Male	1
**Age**	
30 to 39 years	2
40 to 49 years	1
50 to 59 years	2
60 to 69 years	2
**Ethnicity**	
White British	5
British Indian	2
**Years of PPI experience**	
None	2
Less than 1 year	1
1 to 5 years	2
Over 5 years	2
**Further background information**	
PPI reviewer 1. Currently working freelance on education and PPI projects; previously worked in a pastoral role at a college; a lay representative for courses training healthcare scientists.	
PPI reviewer 2. Retired primary school teacher with several long term health conditions; single parent; was a young carer for a parent with a long term condition.	
PPI reviewer 3. Former higher education administrator; current university tutor; patient partner on varied research projects; carer for family members aged 0-100 with physical and/or mental health long term conditions.	
PPI reviewer 4. Currently working as a civil servant and has several long term health conditions.	
PPI reviewer 5. Full-time parent of school age children; previously ten years working in a medical school in an administrative role and 5 years working in the drug and alcohol sector	
PPI reviewer 6. Lay representative for several healthcare-related professional bodies and involved in health research at several universities; family-carer for over 35 years; has had over 6 years of involvement with a mental and community health as a carer	
PPI reviewer 7. Retired university administrator; a parent and carer for elderly parents.	

For peer review only

Table D. Scoring for likelihood that the patient-reported scenario is potentially-harmful preventable-problem

Score	How likely do you think it is the patient was correct in thinking that their health might be worsened, or actually was made worse, because of a mistake or a problem in primary care that could have been prevented? Choose from the options below.
5	Very likely or certain (75-100% confident is a potentially-harmful preventable-problem)
4	Probably (50-74% confident is a potentially-harmful preventable-problem)
3	Possibly (25-49% confident is a potentially-harmful preventable-problem)
2	Unlikely (bottom 25% confident is a potentially-harmful preventable-problem)
1	Definitely not a potentially unsafe event (0% chance is a potentially-harmful preventable-problem)
-	Insufficient information
-	Don't know
-	Other - add text at end of row

For peer review only

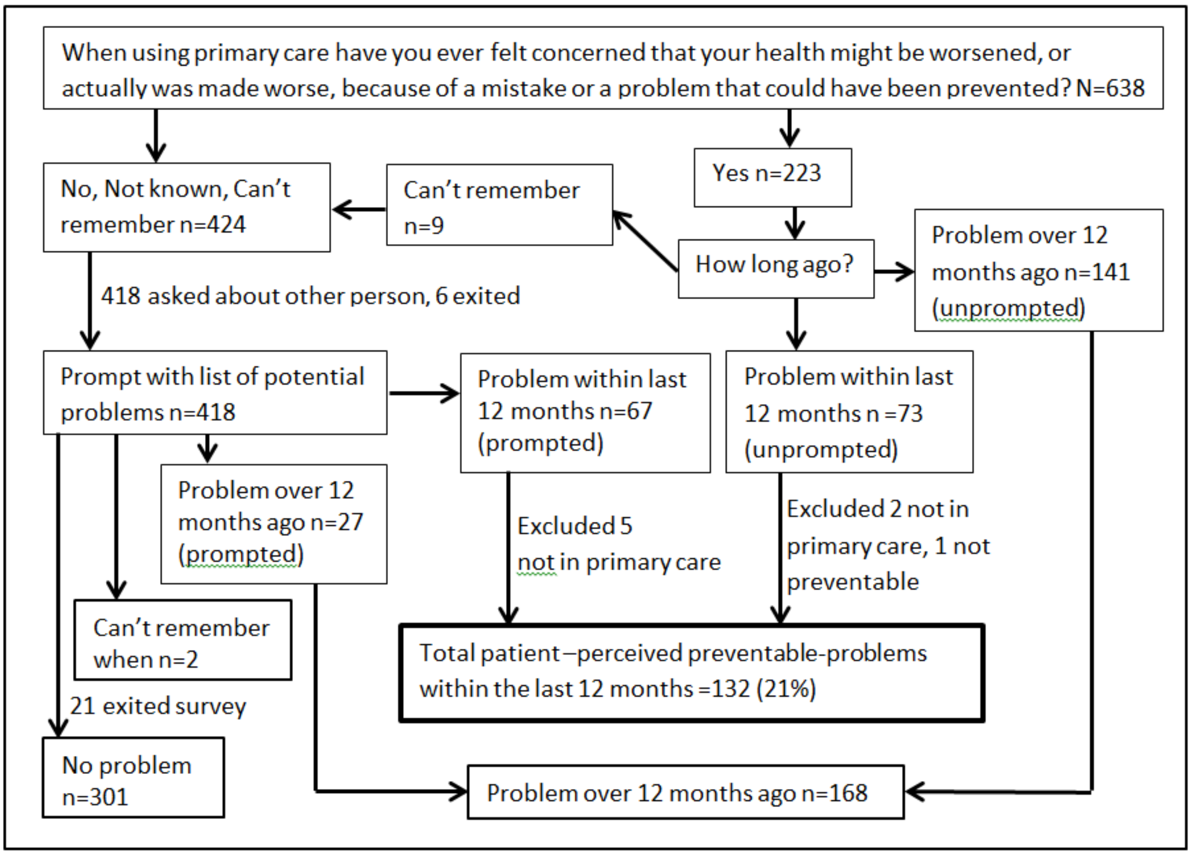

Figure A. Flow chart of participants who reported a potentially-unsafe preventable-problem in primary care through the online pilot survey

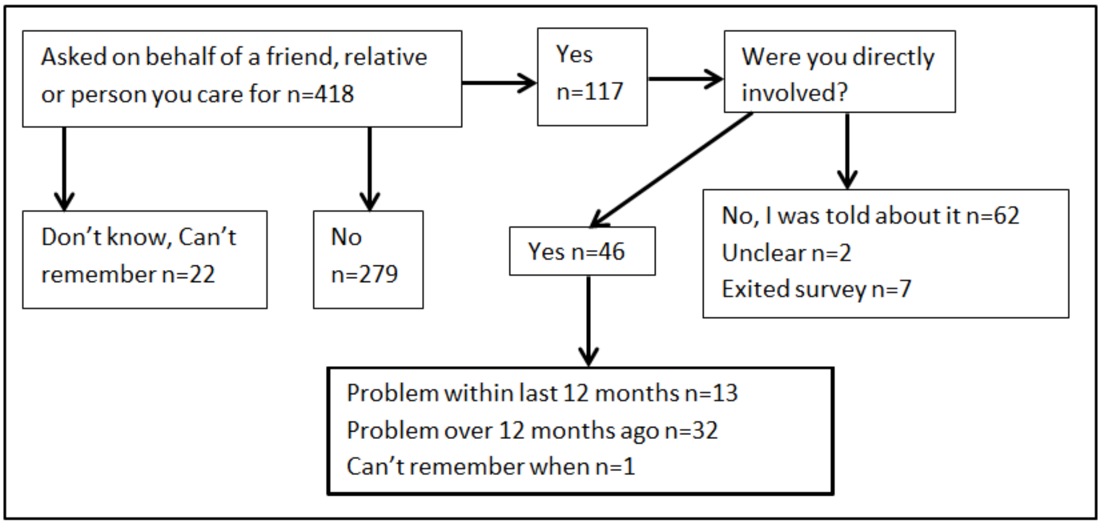

Figure B. Flow chart of participants who reported a potentially-unsafe preventable-problem in primary care on behalf of another person through the online pilot survey

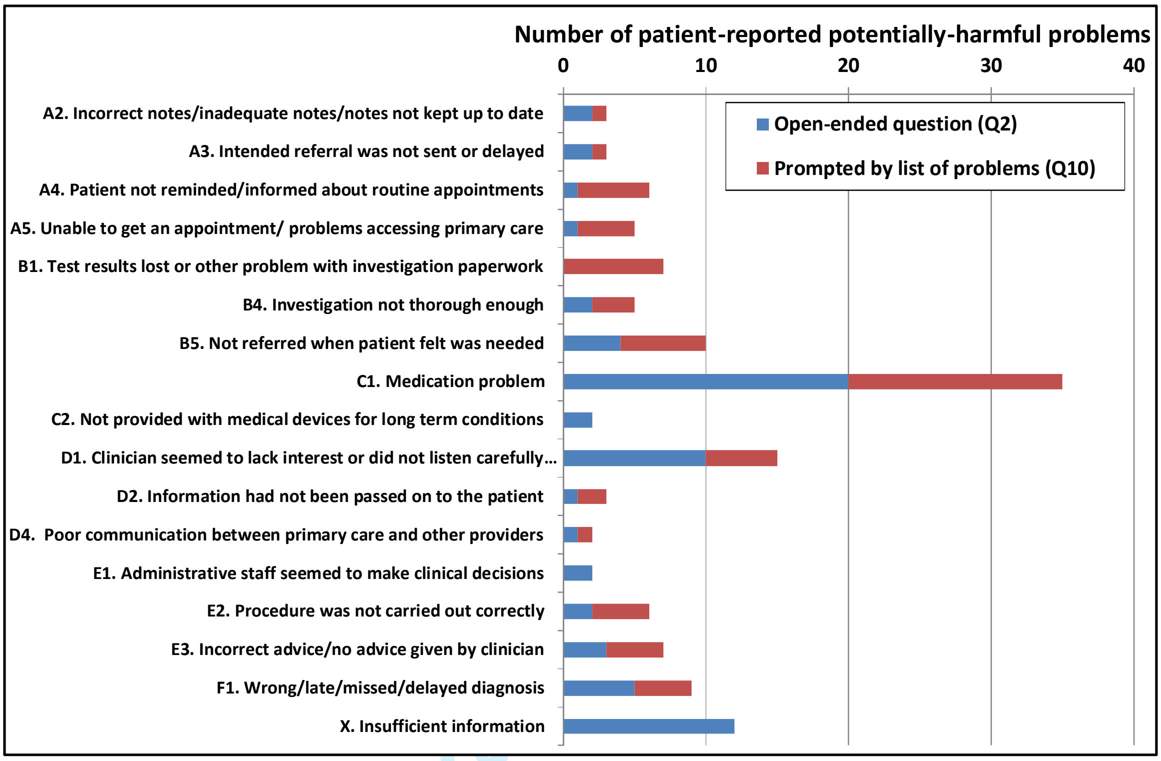

Fig C. Numbers of patient-perceived problems occurring in the last 12 months categorised according to the patient's description (see Table 2) and route through survey *i.e.* originated from open-ended question (Q2) or prompted by list of potential safety problems (Q10)

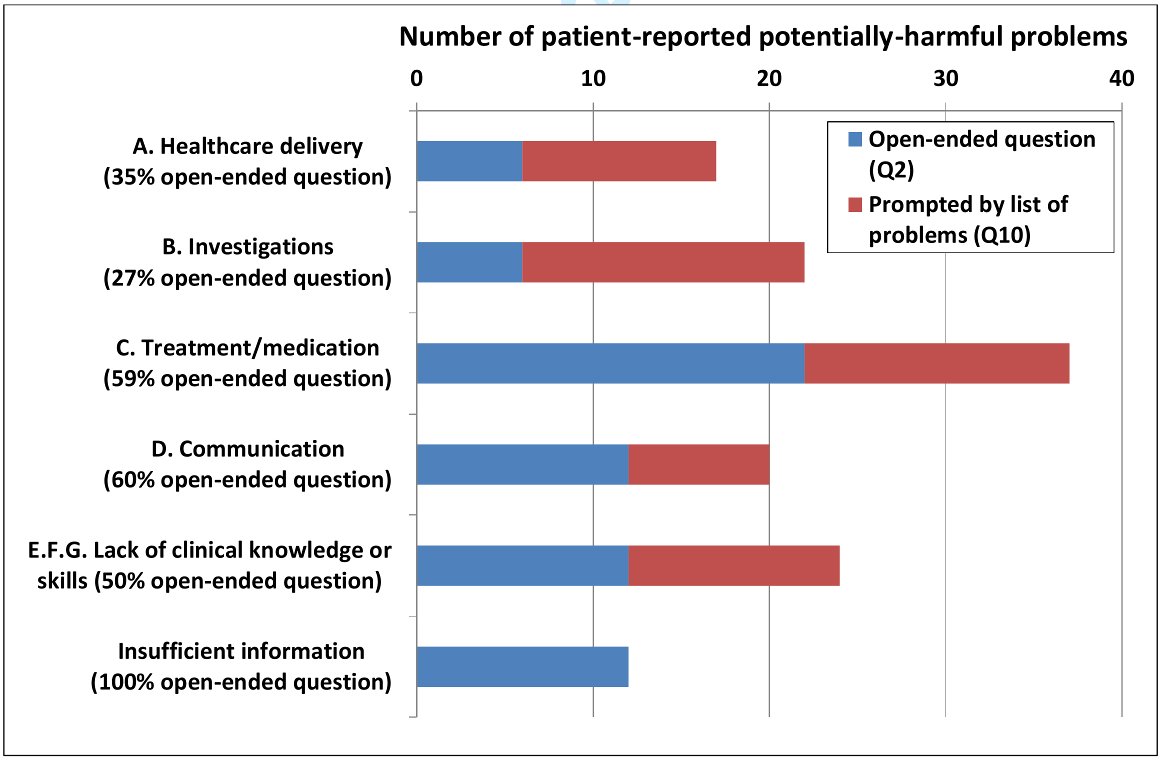

Fig D. Numbers of patient-perceived problems occurring in the last 12 months categorised according to the patient's description coded at a higher level (see Table 2) and route through survey *i.e.* originated from open-ended question (Q2) or prompted by list of potential safety problems (Q10)

Appendix 2. Boxes 1 to 15

Patient reported scenarios occurring during the past 12 months that GPs scored as higher likelihood to be a potentially-unsafe preventable-problem in primary care (median score is higher than "possibly" and at least 2 GPs gave a score or one GP scored "very likely or certain")

Scenario1. GP surgery

Briefly describe the mistake or problem and how it happened. *"Prescription drug, anti-inflammatory for arthritis, caused acute stomach pains & violent vomiting. Repeat prescription for twelve years without any discussion."*

Could the mistake or problem have been avoided? If so how? "*Possible discussion about dangers of continuous taking of prescription drugs, which in the event were stopped after the incident."*

Were you able to talk about the mistake or problem with anybody working in the primary care service? *"No I did not notice the mistake or problem at the time"*

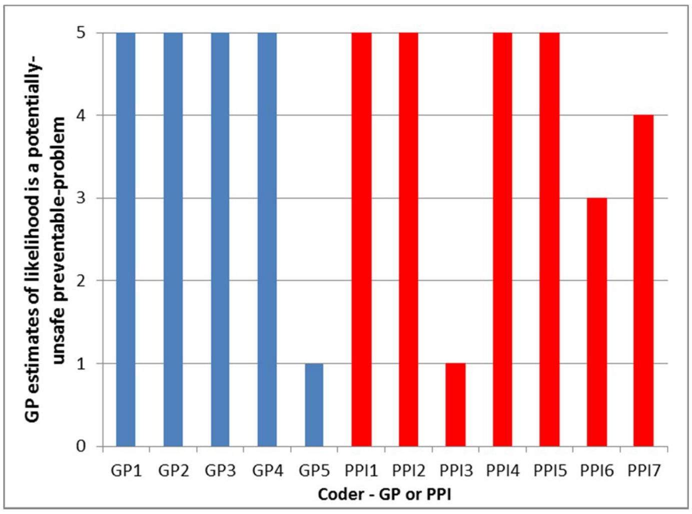

5=very likely or certain, 4=probably, 3=possibly, 2=unlikely, 1=definitely not, 0 = insufficient information or don't know

Patient-reported prospect of harm: suspected your health has been made worse by a problem or error that could have been prevented

Patient-perspective problem-type code: C1.1.3 Long term or continued prescribing without review or consideration of long term or side effects

For peer review

Scenario2. GP surgery

Briefly describe the mistake or problem and how it happened. *"Insulin type was changed by specialist but previous insulin prescribed by GP as notes had not been updated"*

Could the mistake or problem have been avoided? If so how? "*Yes GP notes should have been updated with new medication"*

Were you able to talk about the mistake or problem with anybody working in the primary care service? *"Practice manager resolved the problem and apologised"*

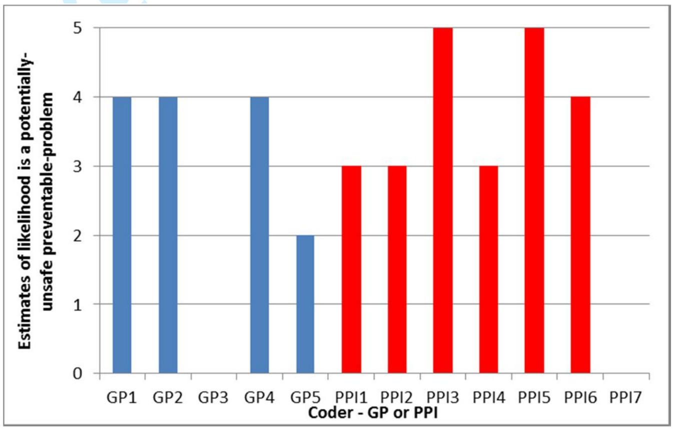

5=very likely or certain, 4=probably, 3=possibly, 2=unlikely, 1=definitely not, 0 = insufficient information or don't know

Patient-reported prospect of harm: prompted via Q10 (Box 1 main paper)

Patient-perspective problem-type code: A2. Incorrect notes/inadequate notes/notes not kept up to date; C1.1.6 Out of date repeat prescription mistakenly re-issued

For peer review only - http://bmjopen.bmj.com/site/about/guidelines.xhtml

Scenario3. GP surgery

Briefly describe the mistake or problem and how it happened. *"Two out of three Doctors not listening to what I was asking; April I had two big bleeds from my Penis, Doctor 1 did a test and gave antibiotics. Went to 2nd Doctor for Diabetic check and told him of problem - nothing except another test come back in ten days. Went to the third doctor who said the test didn't show anything but when I mentioned my feelings about a problem, he look and said yes you do have a problem. In 2 weeks I was in having tests and 3 operations for cancer."*

Could the mistake or problem have been avoided? If so how? "*Listen to me"*

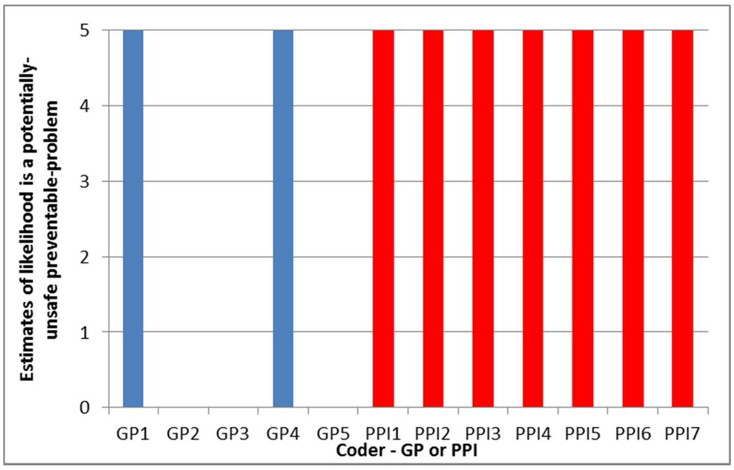

5=very likely or certain, 4=probably, 3=possibly, 2=unlikely, 1=definitely not, 0 = insufficient information or don't know

Were you able to talk about the mistake or problem with anybody working in the primary care service? *"No, I could not find anybody with whom I could discuss the mistake or problem (The third doctor was amazing with me. He said to keep in touch and if I had any problems to ring him and he still wants me to ring him after my three operations.)"*

Patient-reported prospect of harm: suspected your health has been made worse by a problem or error that could have been prevented

Patient-perspective problem-type code: D1. Clinician seemed to lack interest in the patient's health problem or did not listen carefully enough; F1. Wrong/late/missed/delayed diagnosis

Scenario4. GP surgery

Briefly describe the mistake or problem and how it happened. *"Changed diabetes medication to an alternative which my notes from 1980's should show I respond badly to"*

Could the mistake or problem have been avoided? If so how? "*Read the notes on every medication change but unfortunately that is unrealistic under the time restrictions on GP's. Put early notes on-line and flag medication allergies/problems."*

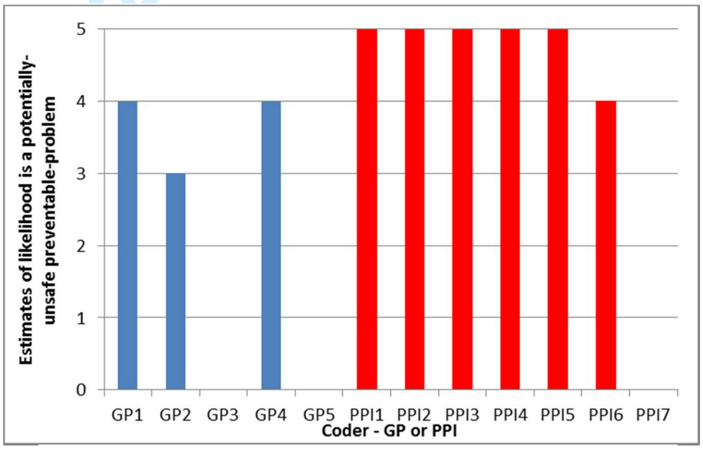

5=very likely or certain, 4=probably, 3=possibly, 2=unlikely, 1=definitely not, 0 = insufficient information or don't know

Were you able to talk about the mistake or problem with anybody working in the primary care service? *"Yes, my own GP who had returned from holiday"*

Patient-reported prospect of harm: suspected your health has been made worse by a problem or error that could have been prevented

Patient-perspective problem-type code: C1.1.4 Prescribed drug when should have known contra-indicated *e.g.* patient had informed clinician of allergy, adverse reaction or it was in the records

Scenario5. GP surgery

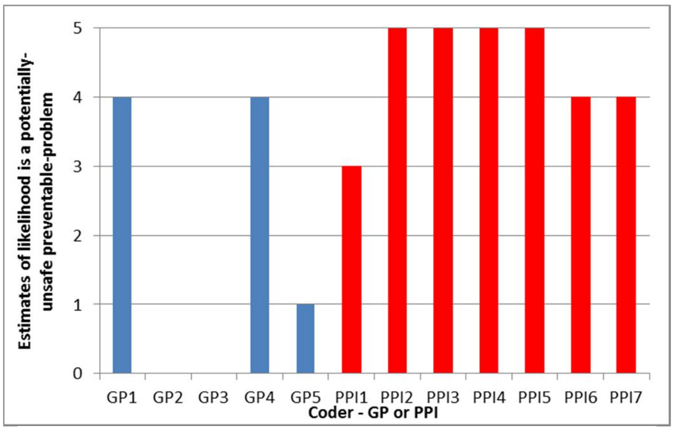

5=very likely or certain, 4=probably, 3=possibly, 2=unlikely, 1=definitely not, 0 = insufficient information or don't know

Briefly describe the mistake or problem and how it happened. *"Told the GP the medication was making my hair fall out & he kept me on it for another 3 months. I had to see another GP to get him to change my medication. In the meantime I have lost 3/4 of my hair. Not sure if it will ever grow back."*

Could the mistake or problem have been avoided? If so how? "*yes, by the GP listening to what I was saying."*

Were you able to talk about the mistake or problem with anybody working in the primary care service? *"Yes, GP"*

Patient-reported prospect of harm: suspected your health has been made worse by a problem or error that could have been prevented

Patient-perspective problem-type code: D1. Clinician seemed to lack interest in the patient's health problem or did not listen carefully enough; C1.1.3 Long term or continued prescribing without review or consideration of long term or side effects

Scenario6. GP surgery

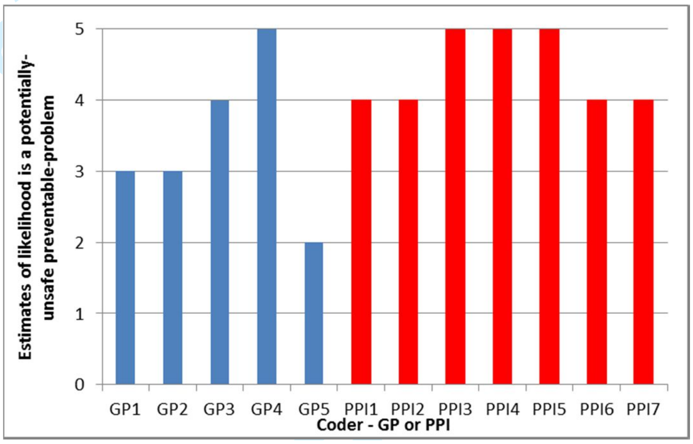

5=very likely or certain, 4=probably, 3=possibly, 2=unlikely, 1=definitely not, 0 = insufficient information or don't know

Briefly describe the mistake or problem and how it happened. *"Successfully treated for prostate cancer 2006 but suffered some loss of sexual performance; Viagra recommended BUT I take isosorbide nitrate for a following heart attack; the two are contradictory and could produce further heart problems. A routine diabetes check-up at which the sexual problem was discussed saw an automatic prescribing of Viagra; obviously without reference to my medical records."*

Could the mistake or problem have been avoided? If so how? "*Read the medical notes."*

Were you able to talk about the mistake or problem with anybody working in the primary care service? *"No; I felt I was going to cause trouble"*

Patient-reported prospect of harm: prompted via Q10 (Box 1 main paper)

Patient-perspective problem-type code: C1.1.1 Prescribed wrong or inappropriate drug

Scenario7. GP surgery

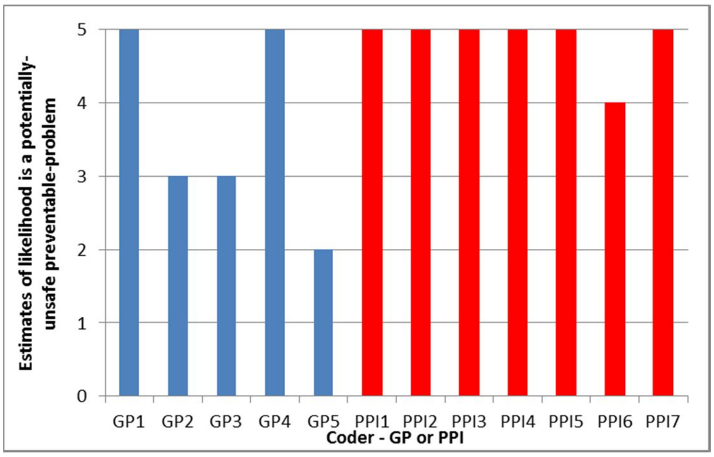

5=very likely or certain, 4=probably, 3=possibly, 2=unlikely, 1=definitely not, 0 = insufficient information or don't know

Briefly describe the mistake or problem and how it happened. *"I was given steroids for a chest infection but not alerted to the fact they make your sugars go massively high! Within a few hours I was high and not able to bring them down, fearing a DKA I headed for the hospital to correct a very easily avoidable issue. I also attended my GP 6 years ago to be given strong antacids for pain in my stomach that was actually a DKA I was admitted to hospital a few hours later! The GP never even suggested it could be linked to my diabetes and as it was my first DKA I had no idea that's how they can feel"*

Could the mistake or problem have been avoided? If so how? "*Both could have been avoided The steroids - if the prescribing nurse had considered my diabetes I'd have been given proper advice as to how to deal with them as a diabetic or given different meds. The DKA simple questions or explanation as to how DKAs can present would have made me family and the doctor realise I was in trouble."*

Were you able to talk about the mistake or problem with anybody working in the primary care service? *"I wrote a letter to the surgery concerning the steroids anonymously to alert them of my concern and the DKA. I was too poorly to even consider seeking correction or explanation"*

Patient-reported prospect of harm: health was actually made worse by a problem or error that could have been prevented

Patient-perspective problem-type code: C1.1.4 Prescribed drug when should have known contra-indicated *e.g.* patient had informed clinician of allergy, adverse reaction or it was in the records; E3. Incorrect advice/no advice given by clinician

Scenario8. GP surgery

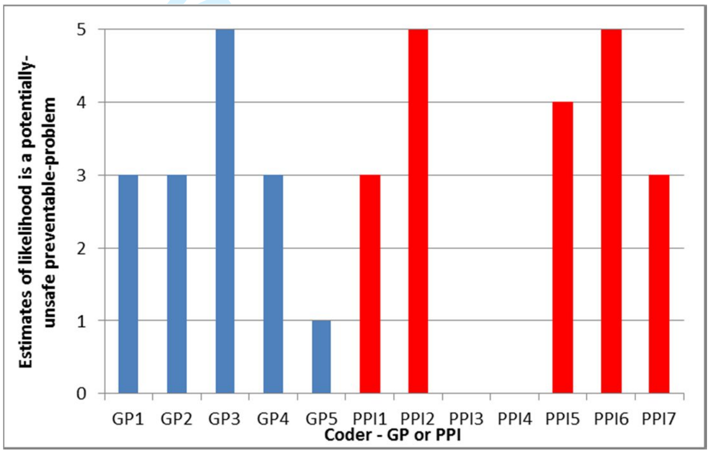

5=very likely or certain, 4=probably, 3=possibly, 2=unlikely, 1=definitely not, 0 = insufficient information or don't know

Briefly describe the mistake or problem and how it happened. *"reception staff making clinical decisions which were at odds with what had been discussed with my GP"*

Could the mistake or problem have been avoided? If so how? "*Yes, reception staff shouldn't be making clinical decisions"*

Were you able to talk about the mistake or problem with anybody working in the primary care service? *"No, had the opportunity but did not feel comfortable to discuss the mistake or problem"*

Patient-reported prospect of harm: suspected your health has been made worse by a problem or error that could have been prevented

Patient-perspective problem-type code: E1. Administrative staff seemed to make clinical decisions

For peer review only - http://bmjopen.bmj.com/site/about/guidelines.xhtml

Scenario9. Pharmacist

Briefly describe the mistake or problem and how it happened. *"I was given a medicine belonging to somebody else as part of my monthly repeat prescription"*

Could the mistake or problem have been avoided? If so how? "*More care and attention when checking"*

Were you able to talk about the mistake or problem with anybody working in the primary care service? *"Yes, pharmacist"*

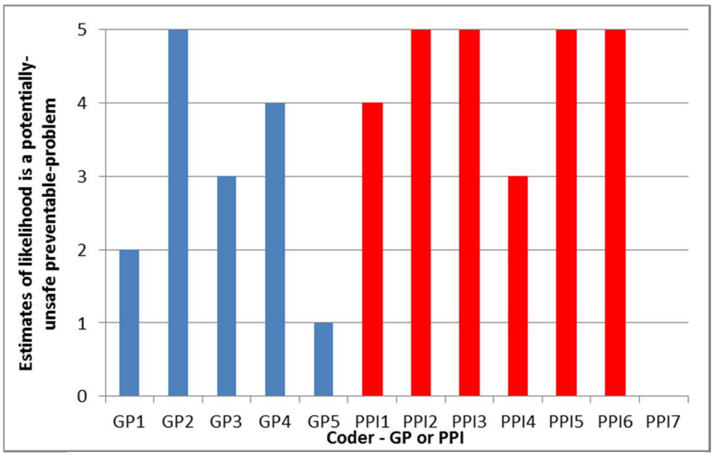

5=very likely or certain, 4=probably, 3=possibly, 2=unlikely, 1=definitely not, 0 = insufficient information or don't know

Patient-reported prospect of harm: prompted via Q10 (Box 1 main paper)

Patient-perspective problem-type code: C1.3.3 Wrong or inadequate advice about drug effects or how to use

Scenario10. GP surgery

Briefly describe the mistake or problem and how it happened. *"Poor diabetic annual review, foot check not correctly done just tested my foot pulses and nothing else"*

Could the mistake or problem have been avoided? If so how? "*Better training of staff"*

Were you able to talk about the mistake or problem with anybody working in the primary care service? *"No, had the opportunity but did not feel comfortable to discuss the mistake or problem"*

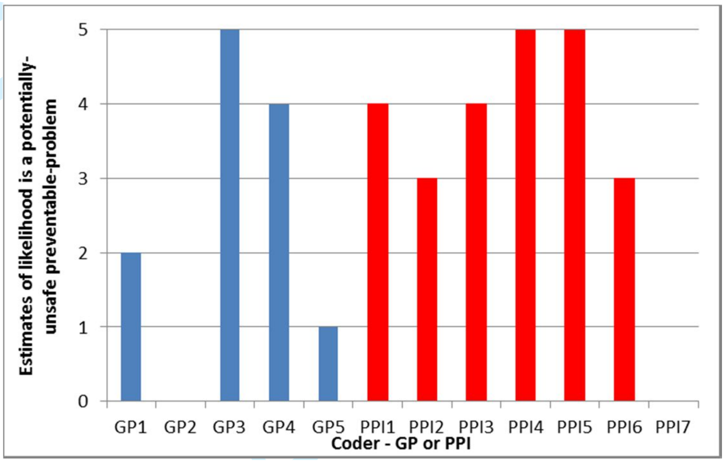

5=very likely or certain, 4=probably, 3=possibly, 2=unlikely, 1=definitely not, 0 = insufficient information or don't know

Patient-reported prospect of harm: suspected your health has been made worse by a problem or error that could have been prevented

Patient-perspective problem-type code: E2. Procedure was not carried out correctly

Scenario11. GP surgery

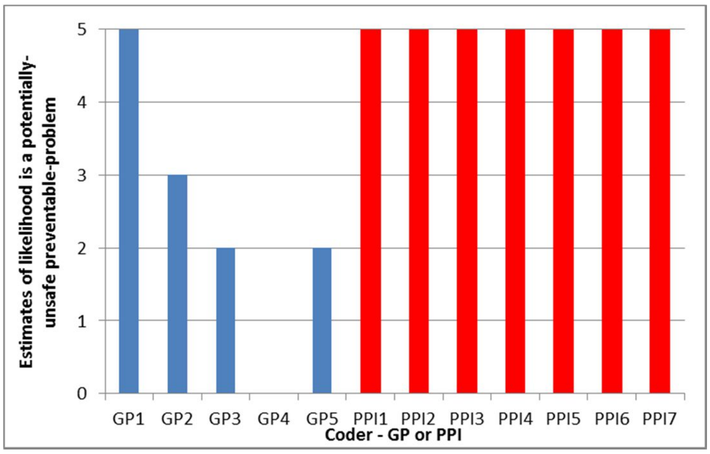

5=very likely or certain, 4=probably, 3=possibly, 2=unlikely, 1=definitely not, 0 = insufficient information or don't know

Briefly describe the mistake or problem and how it happened. *"Prior to a pain killing injection into my knee, I asked the GP who suggested the injection AND the GP who carried out the injection whether, as someone living with Type 1 diabetes, it would have any effect on my blood glucose levels. On both occasions, I was given an unequivocal No . In the event, within a few hours of the injection, my blood glucose rose significantly and remained high for several days. I felt unable to eat anything for 24 hours while I took on more and more insulin in order to bring my glucose levels down - I did not want to go to sleep that night simply because of the massive amount of insulin in my system."*

Could the mistake or problem have been avoided? If so how? "*Yes. I feel that both GPs should have a knowledge about the side effects of drugs they prescribe, administer and recommend."*

Were you able to talk about the mistake or problem with anybody working in the primary care service? *"No I could not find anybody with whom I could discuss the mistake or problem"*

Patient-reported prospect of harm: your health has been made worse by a problem or error that could have been prevented

Patient-perspective problem-type code: E3. Incorrect advice/no advice given by clinician

Scenario12. GP surgery

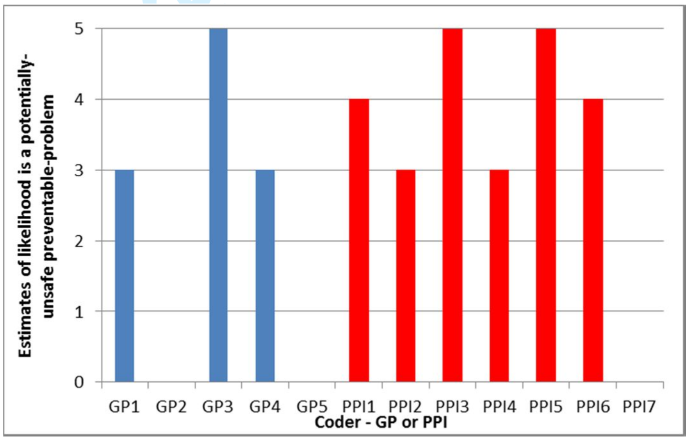

5=very likely or certain, 4=probably, 3=possibly, 2=unlikely, 1=definitely not, 0 = insufficient information or don't know

Briefly describe the mistake or problem and how it happened. *"GP completely overlooked symptoms and prescribed antibiotic after antibiotic without investigation or referral"*

Could the mistake or problem have been avoided? If so how? "*Yes by listening to history of complaints, carrying out appropriate tests instead of just giving antibiotics"*

Were you able to talk about the mistake or problem with anybody working in the primary care service? *"No I did not notice the mistake or problem at the time"*

Patient-reported prospect of harm: prompted via Q10 (Box 1 main paper)

Patient-perspective problem-type code: D1. Clinician seemed to lack interest in the patient's health problem or did not listen carefully enough; F1. Wrong/late/missed/delayed diagnosis

For peer review only - http://bmjopen.bmj.com/site/about/guidelines.xhtml

Scenario13. GP surgery

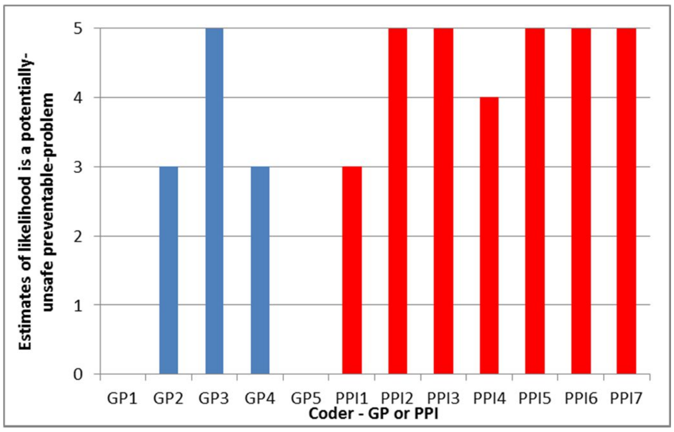

5=very likely or certain, 4=probably, 3=possibly, 2=unlikely, 1=definitely not, 0 = insufficient information or don't know

Briefly describe the mistake or problem and how it happened. *"Several times prescriptions have been incorrectly issued due to similar names for drugs or the same name with different strengths"*

Could the mistake or problem have been avoided? If so how? "*Yes, by more accurate or double data entry. Now solved by self-request using web systems."*

Were you able to talk about the mistake or problem with anybody working in the primary care service? "Yes, *they did not want to know or seem to care unless a formal complaint was made"*

Patient-reported prospect of harm: your health has been made worse by a problem or error that could have been prevented

Patient-perspective problem-type code: C1.1.5 Repeat prescription unintentionally changed

Scenario14. GP surgery

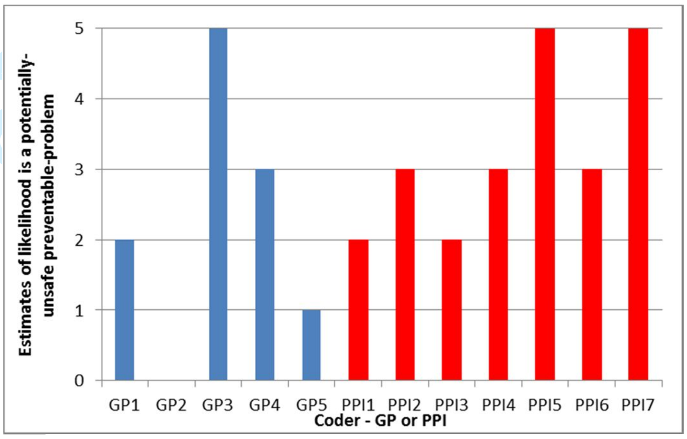

5=very likely or certain, 4=probably, 3=possibly, 2=unlikely, 1=definitely not, 0 = insufficient information or don't know

Briefly describe the mistake or problem and how it happened. *"A simple error occurred with an incorrect prescription. When I tried to bring this to the attention of the receptionist she treated me with disdain and in a challenging manner. She then proceeded to start to read my notes aloud in the public reception area. I felt that this was unacceptable behaviour. When I tried to tackle the receptionist about her behaviour I felt as if I was under threat. It caused me to feel very stressed, frustrated and ill tempered."*

Could the mistake or problem have been avoided? If so how? "*If the receptionist had been willing to listen to what I was saying."*

Were you able to talk about the mistake or problem with anybody working in the primary care service? *"I did speak to a lady who said she was the practice manager but I felt that they were not interested in resolving the problem"*

Patient-reported prospect of harm: suspected your health has been made worse by a problem or error that could have been prevented

Patient-perspective problem-type code: D3. Communication problem between patient and primary care staff; C1 Medication error not otherwise specified /other problem

For peer review only

Scenario15. GP Surgery

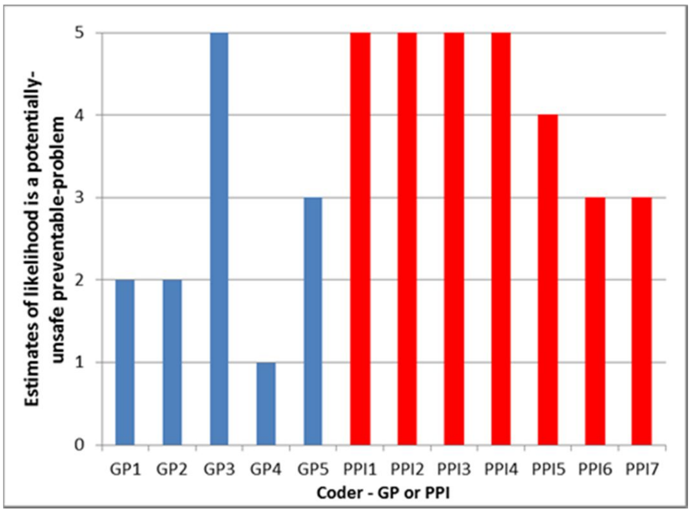

5=very likely or certain, 4=probably, 3=possibly, 2=unlikely, 1=definitely not, 0 = insufficient information or don't know

Briefly describe the mistake or problem and how it happened. *"Went to see GP because I feared the pain in one of my legs may have been Peripheral Artery Disease - hardening of the arteries, having had a (non-blood) relative who suffered from this and subsequently died - of a heart attack. Oh yes, said the GP, well, you will have it won't you? Why? I asked expecting her to say eg because you are a smoker, or maybe my age (65) or something else I wasn't aware of. But what she actually told me was 'Because you are a diabetic!' Whaaat? I exclaimed - you mean ALL diabetics will inevitably get this, and there's no way to prevent it? Yes she said and shrugged. I said 'Thanks for nothing then' and left. Instead I left, came home and went straight on-line to make an appointment with someone more sensible, which I did and after taking my leg/ankle pulses and BPs etc - he chatted to me and said he would refer me for a cardiology consultation at the hospital. This IS what I expected in the first place and now it IS being taken care of***."

Could the mistake or problem have been avoided? If so how? *"By training the GP properly in the first place"*

Were you able to talk about the mistake or problem with anybody working in the primary care service? "? *"I explained to GP 2 But I don't know what if anything was done about it, or how I could find that out."*

Patient-reported prospect of harm: your health has been made worse by a problem or error that could have been prevented

Patient-perspective problem-type code: D1. Clinician seemed to lack interest in the patient's health problem or did not listen carefully enough

For peer review only

Appendix 2. Boxes 16 to 23

Patient reported scenarios occurring during the past 12 months that PPIs scored as higher likelihood to be a potentially-unsafe preventable-problem in primary care compared with GPs

Scenario16. GP Surgery

Briefly describe the mistake or problem and how it happened. *"I had a severe reaction to Atorvastatin after a dose increase so much so that I was almost immobile and took 4 months to recover"*

Could the mistake or problem have been avoided? If so how? *"According to guidelines I should have been on the increased dose - it took a long time to convince the GP that I needed blood tests to find out why I couldn't walk. My GP was very hesitant to admit that I did have a reaction to statins."*

Were you able to talk about the mistake or problem with anybody working in the primary care service? **"***No I could not find anybody with whom I could discuss the mistake or problem. It was not really the GPs fault per se, just took a lot of convincing that there was a problem"*

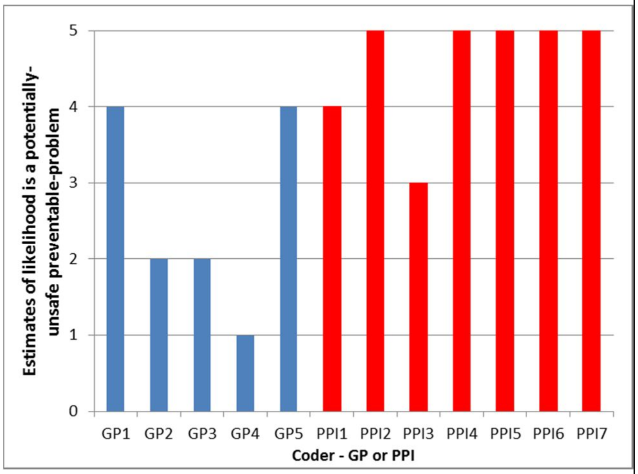

5=very likely or certain, 4=probably, 3=possibly, 2=unlikely, 1=definitely not, 0 = insufficient information or don't know

Patient-reported prospect of harm: health could have been made worse had someone not noticed a problem or error

Patient-perspective problem-type code: C1.1.3 Long term or continued prescribing without review or consideration of long term or side effects

For peer review only

Scenario17. GP Surgery

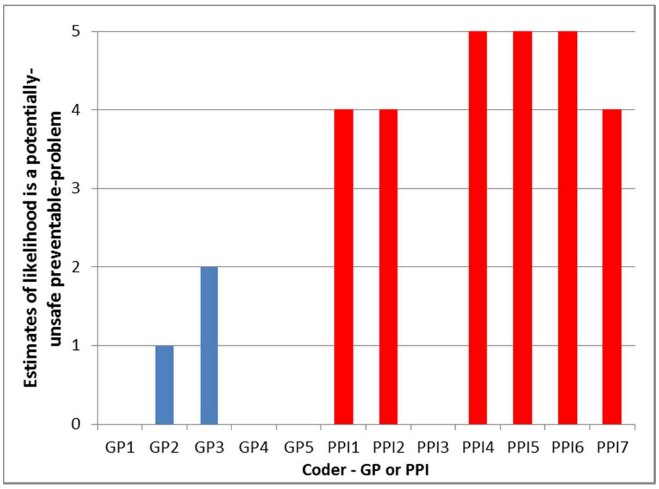

5=very likely or certain, 4=probably, 3=possibly, 2=unlikely, 1=definitely not, 0 = insufficient information or don't know

Briefly describe the mistake or problem and how it happened. *"Doctor kept saying I had vitamin deficiency B1, it turned out I had peripheral neuropathy which is very painful"*

Could the mistake or problem have been avoided? If so how? *"I just needed the proper medication to help"*

Were you able to talk about the mistake or problem with anybody working in the primary care service? *"Just saw another Doctor and she knew straight away what the problem was - she was experienced with Diabetic problems. Yes had the opportunity but did not feel comfortable to discuss the mistake or problem"*

Patient-reported prospect of harm: prompted via Q10 (Box 1 main paper)

Patient-perspective problem-type code: F1. Wrong/late/missed/delayed diagnosis

Scenario18. GP Surgery

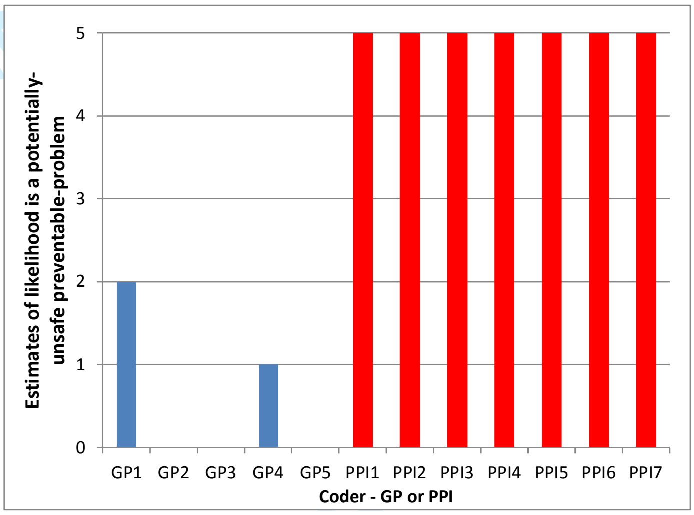

5=very likely or certain, 4=probably, 3=possibly, 2=unlikely, 1=definitely not, 0 = insufficient information or don't know

Briefly describe the mistake or problem and how it happened. *"Incapable diabetic doctor trying to take blood out the back of my hand haphazardly, not listening and resulting in me fitting and the student watching having to get help."*

Could the mistake or problem have been avoided? If so how? *"Yes. By listening to me"*

Were you able to talk about the mistake or problem with anybody working in the primary care service? *"No I could not find anybody with whom I could discuss the mistake or problem"*

Patient-reported prospect of harm: prompted via Q10 (Box 1 main paper)

Patient-perspective problem-type code: E2. Procedure was not carried out correctly; D1. Clinician seemed to lack interest in the patient's health problem or did not listen carefully enough

For peer review only - http://bmjopen.bmj.com/site/about/guidelines.xhtml

Scenario19. Dental Surgery

Briefly describe the mistake or problem and how it happened. *"I had an infection under my wisdom tooth. They agreed that the only way to solve the problem was to take the tooth out. They gave me an appointment to do this in 6 weeks. I am a type 1 diabetic and the infection was affecting my blood sugars and I was concerned that I would have to go to A&E if my blood sugars continued to rise due to the infection. It would have affected my health if I had not paid to go to a private dentist."*

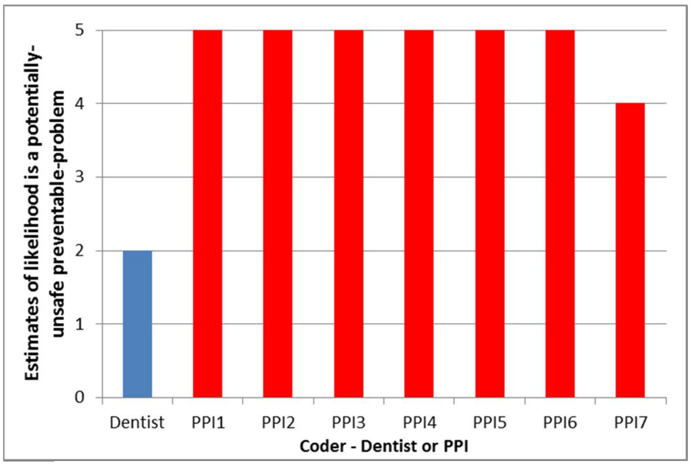

5=very likely or certain, 4=probably, 3=possibly, 2=unlikely, 1=definitely not, 0 = insufficient information or don't know

Could the mistake or problem have been avoided? If so how? *"They could have taken out the tooth straight away. I was happy to wait at the emergency dentist for them to do this."*

Were you able to talk about the mistake or problem with anybody working in the primary care service? *"I explained but they said I would have to wait. They also asked if I needed a sugary drink when I said that my sugars were high so I was too scared to eat and had not eaten in 12hrs. It was clear they didn't understand diabetes."*

Patient-reported prospect of harm: health could have been made worse had someone not noticed a problem or error

Patient-perspective problem-type code: A5. Unable to get an appointment/other problems with making appointment

Scenario20. Dental Surgery

Briefly describe the mistake or problem and how it happened. *"Caries, cavities and problem with crown not diagnosed or treated"*

Could the mistake or problem have been avoided? If so how? *"Better dentist & not working to tight time-scale imposed by company owning dental surgery"*

Were you able to talk about the mistake or problem with anybody working in the primary care service? *"No I could not find anybody with whom I could discuss the mistake or problem"*

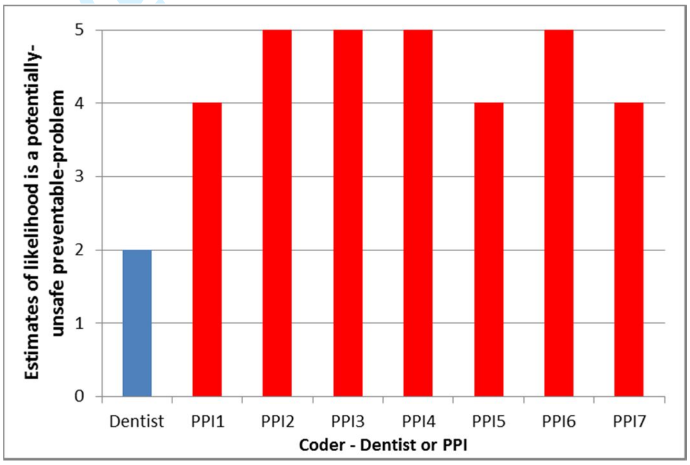

5=very likely or certain, 4=probably, 3=possibly, 2=unlikely, 1=definitely not, 0 = insufficient information or don't know

Patient-reported prospect of harm: prompted via Q10 (Box 1 main paper)

Patient-perspective problem-type code: C3. Problem with dental treatment or diagnosis

For peer review

Scenario21GP Surgery

Briefly describe the mistake or problem and how it happened. ***“****Using the summary on discharge from hospital, one GP transcribed incorrectly on to my electronic notes ie size of ovarian cyst was 7.5cms and he put 7.5 mms. Another GP requested diagnostic bone density scan but either forgot or did not record it and she ended up questioning why I had it and who requested it. She also referred me for an orthopedic consultation then said I was not funded for the steroid injection put into my swollen elbows.”*

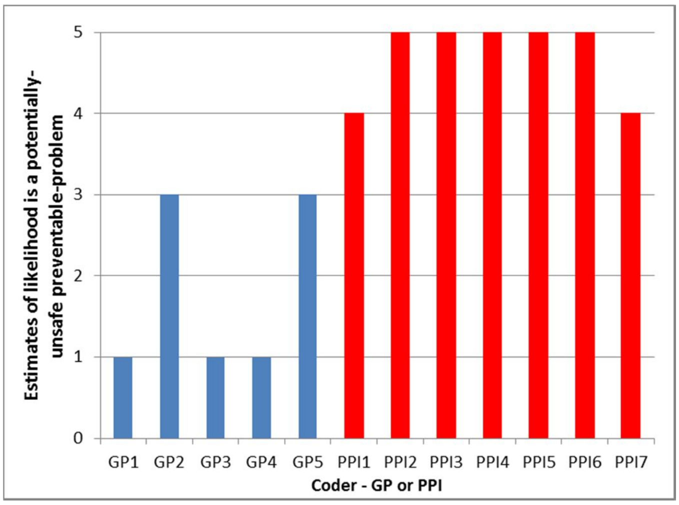

5=very likely or certain, 4=probably, 3=possibly, 2=unlikely, 1=definitely not, 0 = insufficient information or don’t know

Could the mistake or problem have been avoided? If so how? *“Yes”*

Were you able to talk about the mistake or problem with anybody working in the primary care service? *“I was too scared to discuss my concerns for fear of being labelled a trouble maker”*

Patient-reported prospect of harm: health could have been made worse had someone not noticed a problem or error

Patient-perspective problem-type code: A2. Incorrect notes/inadequate notes/notes not kept up to date

Scenario22. GP Surgery

Briefly describe the mistake or problem and how it happened. ***“****GP prescribed pills, but then got phone call saying not to take them”*

Could the mistake or problem have been avoided? If so how? *“Not sure”*

Were you able to talk about the mistake or problem with anybody working in the primary care service? *“No I was not concerned about the problem”*

Patient-reported prospect of harm: prompted via Q10 (Box 1 main paper)

Patient-perspective problem-type code: C1. Medication problem

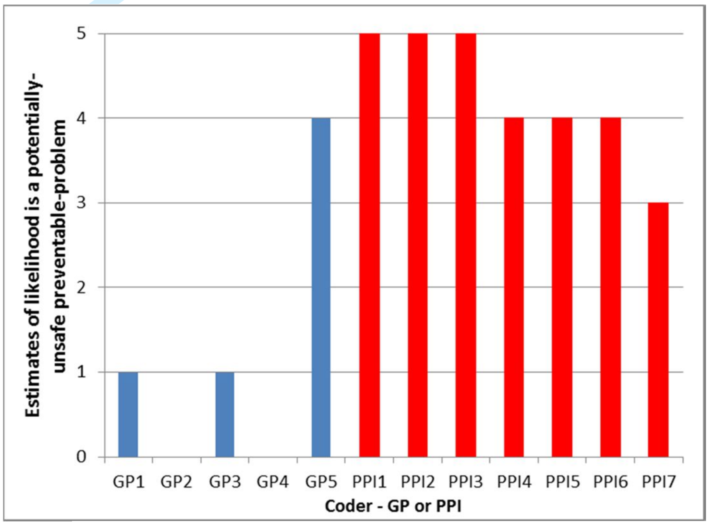

5=very likely or certain, 4=probably, 3=possibly, 2=unlikely, 1=definitely not, 0 = insufficient information or don’t know

For peer revi

Scenario23. GP Surgery

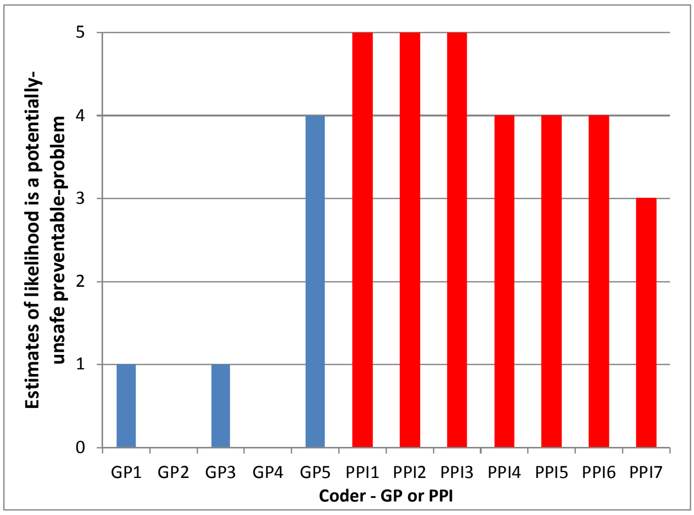

5=very likely or certain, 4=probably, 3=possibly, 2=unlikely, 1=definitely not, 0 = insufficient information or don't know

Briefly describe the mistake or problem and how it happened. *"I had a burst appendix and peritonitis, something that even a scan couldn't detect adequately. My first visit to GP was when I said I think I have appendicitis, no other symptoms only the pain. It was ten days before seeing a consultant, a further 10 days to have a scan, then 2 weeks to be told that I had a lump on my colon which is what my GP had said 5 weeks previously. It was a further 2 weeks before I had surgery."*

Could the mistake or problem have been avoided? If so how? *"If my GP had referred me for a scan immediately it would have saved 3 weeks out of the seven. It was two weeks from scan to results and I hear that is usual, but they're not looking at them for 2 weeks"*

Were you able to talk about the mistake or problem with anybody working in the primary care service? *"Had the outcome been different my widow might have pursued the matter further. The system is at fault rather than any individual."*

Patient-reported prospect of harm: your health has been made worse by a problem or error that could have been prevented

Patient-perspective problem-type code: B5. Not referred when patient felt was needed

For peer review only

STROBE Statement—Checklist of items that should be included in reports of ***cross-sectional studies***

	Item No	Recommendation
**Title and abstract**	1	(*a*) Indicate the study's design with a commonly used term in the title or the abstract Yes p1
		(*b*) Provide in the abstract an informative and balanced summary of what was done and what was found yes p2
**Introduction**		
Background/rationale	2	Explain the scientific background and rationale for the investigation being reported Yes p3
Objectives	3	State specific objectives, including any prespecified hypotheses yes p3-4
**Methods**		
Study design	4	Present key elements of study design early in the paper yes p4
Setting	5	Describe the setting, locations, and relevant dates, including periods of recruitment, exposure, follow-up, and data collection yes p4
Participants	6	(*a*) Give the eligibility criteria, and the sources and methods of selection of participants yes p4
Variables	7	Clearly define all outcomes, exposures, predictors, potential confounders, and effect modifiers. Give diagnostic criteria, if applicable yes box1, online appendix 1
Data sources/ measurement	8*	For each variable of interest, give sources of data and details of methods of assessment (measurement). Describe comparability of assessment methods if there is more than one group yes p5, online appendix 1
Bias	9	Describe any efforts to address potential sources of bias yes p4
Study size	10	Explain how the study size was arrived at n/a as is a pilot study.
Quantitative variables	11	Explain how quantitative variables were handled in the analyses. If applicable, describe which groupings were chosen and why yes p5, table2
Statistical methods	12	(*a*) Describe all statistical methods, including those used to control for confounding yes p5
		(*b*) Describe any methods used to examine subgroups and interactions, yes just chi2 tests p5
		(*c*) Explain how missing data were addressed all missing data is listed in the tables so it is completely transparent how this was dealt with, there were few missing data
		(*d*) If applicable, describe analytical methods taking account of sampling n/a
		(*e*) Describe any sensitivity analyses n/a
**Results**		
Participants	13*	(a) Report numbers of individuals at each stage of study—eg numbers potentially eligible, examined for eligibility, confirmed eligible, included in the study, completing follow-up, and analysed yes online appendix 1
		(b) Give reasons for non-participation at each stage yes online appendix 1
		(c) Consider use of a flow diagram yes online appendix 1
Descriptive data	14*	(a) Give characteristics of study participants (eg demographic, clinical, social) and information on exposures and potential confounders yes table 1
		(b) Indicate number of participants with missing data for each variable of interest yes all tables
Outcome data	15*	Report numbers of outcome events or summary measures yes all tables
Main results	16	(*a*) Give unadjusted estimates and, if applicable, confounder-adjusted estimates and their precision (eg, 95% confidence interval). Make clear which confounders were

For peer review only - http://bmjopen.bmj.com/site/about/guidelines.xhtml

		adjusted for and why they were included yes table 3
		(*b*) Report category boundaries when continuous variables were categorized yes all tables
		(*c*) If relevant, consider translating estimates of relative risk into absolute risk for a meaningful time period not appropriate as pilot study with self-selected sample
Other analyses	17	Report other analyses done—eg analyses of subgroups and interactions, and sensitivity analyses table 6 considers demographics for problems more likely to be a potentially harmful.
**Discussion**		
Key results	18	Summarise key results with reference to study objectives yes p7
Limitations	19	Discuss limitations of the study, taking into account sources of potential bias or imprecision. Discuss both direction and magnitude of any potential bias yes p8
Interpretation	20	Give a cautious overall interpretation of results considering objectives, limitations, multiplicity of analyses, results from similar studies, and other relevant evidence yes p7-8
Generalisability	21	Discuss the generalisability (external validity) of the study results yes p8, not generalisable
**Other information**		
Funding	22	Give the source of funding and the role of the funders for the present study and, if applicable, for the original study on which the present article is based yes p9

*Give information separately for exposed and unexposed groups.

Note: An Explanation and Elaboration article discusses each checklist item and gives methodological background and published examples of transparent reporting. The STROBE checklist is best used in conjunction with this article (freely available on the Web sites of PLoS Medicine at http://www.plosmedicine.org/, Annals of Internal Medicine at http://www.annals.org/, and Epidemiology at http://www.epidem.com/). Information on the STROBE Initiative is available at www.strobe-statement.org.

For peer review only

BMJ Open

Development and piloting of a survey to estimate the frequency and nature of potentially-harmful preventable-problems in primary care from a UK patient's perspective

Journal:	*BMJ Open*
Manuscript ID	bmjopen-2017-017786.R2
Article Type:	Research
Date Submitted by the Author:	02-Oct-2017
Complete List of Authors:	Stocks, Susan; University of Manchester, GM Patient Safety Translational Research Centre<br>Donnelly, Ailsa; University of Manchester, PRIMER<br>Esmail, Aneez; University of Manchester, School of Primary Care<br>Beresford, Joanne; Research User Group (RUG) of the NIHR Greater Manchester Primary Care Patient Safety Translational Research Centre<br>Gamble, Carolyn; University of Manchester, GM Patient Safety Translational Research Centre<br>Luty, Sarah; NHS Scotland<br>Deacon, Richard; St Gabriels Medical Centre<br>Danczak, Avril; Central and South Manchester Specialty Training Programme for General Practice<br>Mann, Nicola; University of Manchester, GMPSTRC<br>Townsend, David; University of Manchester, GM Patient Safety Translational Research Centre<br>Ashley, James; Division of Dentistry, School of Medical Sciences, University of Manchester<br>Bowie, Paul; NHS Scotland<br>Campbell, Stephen; University of Manchester, Centre for Primary Care
**Primary Subject Heading**:	Research methods
Secondary Subject Heading:	General practice / Family practice, Health services research, Communication, Patient-centred medicine, Research methods
Keywords:	STATISTICS & RESEARCH METHODS, PRIMARY CARE, Quality in health care < HEALTH SERVICES ADMINISTRATION & MANAGEMENT

SCHOLARONE™
Manuscripts

Development and piloting of a survey to estimate the frequency and nature of potentially-harmful preventable-problems in primary care from a UK patient's perspective

S Jill Stocks *research fellow*[1], Ailsa Donnelly *public and patient involvement volunteer*[2], Aneez Esmail *professor*[1], Joanne Beresford *public and patient involvement volunteer*[2] Carolyn Gamble *public and patient involvement volunteer*[2], Sarah Luty *general practitioner*[3], Richard Deacon *general practitioner*[4], Avril Danczak *general practitioner*[5], Nicola Mann *general practitioner*[2], David Townsend *general practitioner*[2], James Ashley *general dental practitioner*[6], Paul Bowie *programme director*[7,8], Stephen M Campbell *professor*[1]

1. NIHR Greater Manchester Primary Care Patient Safety Translational Research Centre, Centre for Primary Care, Division of Population Health, Health Services Research and Primary Care, University of Manchester, Manchester M13 9PL, UK

2. Research User Group (RUG) of the NIHR Greater Manchester Primary Care Patient Safety Translational Research Centre, Centre for Primary Care, Division of Population Health, Health Services Research and Primary Care, University of Manchester, Manchester M13 9PL, UK

3. General Practitioner NHS Greater Glasgow and Clyde, Medical Directorate, NHS Education for Scotland, Glasgow G3 8BW

4. St Gabriels Medical Centre, 4, Bishops Road, Prestwich, Manchester M25 0HT, UK

5. Central and South Manchester Specialty Training Programme for General Practice, Health Education England North West (HEENWE) Education and Research Centre, Wythenshawe Hospital, Manchester M23 9LT, UK

6. Woodlands Dental Practice, Rock Ferry, Wirral, CH42 4NG, UK

7. Medical Directorate, NHS Education for Scotland, 2 Central Quay, Glasgow G3 8BW, UK.

8. Institute of Health and Wellbeing, University of Glasgow, Glasgow G12 0XH, UK

Correspondence to jill.stocks@manchester.ac.uk

Word count: 3856

For peer review only - http://bmjopen.bmj.com/site/about/guidelines.xhtml

ABSTRACT

Objectives: To design and pilot a survey to be used at the population level to estimate the frequency of patient-perceived potentially-harmful preventable-problems occurring in UK primary care. To explore the nature of the problems, patient-suggested strategies for prevention and opinions of clinicians and the public regarding the potential for harm

Design: a survey was co-designed by three members of the public and one researcher and piloted through public and patient involvement and engagement networks

Setting: self-selected sample of the UK population

Participants: 977 members of the public accessed the online survey during October and November 2015

Primary outcome measures: respondent feedback about the ease of completion of the survey, quality of responses in terms of review by clinicians and members of the public, preliminary estimates of the frequency and nature of patient-perceived potentially-harmful problems occurring in the last 12 months

Results: 638 (65%) members of the public completed the survey and few respondents reported any difficulty in understanding or completing the survey. 132 (21%) respondents reported experiencing a potentially-harmful preventable-problem during the past 12 months and 108 (82%) of these respondents provided a description that was adequate for at least one clinician to form an opinion about the potentially-harmful problem. Respondents were older than the UK generally, more likely to work or volunteer in the healthcare sector and tended to use primary care more frequently but their confidence and trust in their own GP was similar to that of the UK population as measured by the annual English GP patient survey.

Conclusions: the survey was acceptable to patients and mostly provided data of sufficient quality for review by clinicians and members of the public. It is now ready to use at a population level to estimate the frequency and nature of potentially-harmful preventable-problems in primary care from a patient's perspective.

Strengths and limitations of this study

- We have designed and tested a survey to measure the frequency and nature of potentially-harmful preventable-problems in primary care from the patient's perspective
- The survey was co-designed by three members of the public and piloted through extensive public and patient involvement
- The patient-described scenarios were reviewed by primary care clinicians
- The study respondents were self-selected through public and patient involvement and engagement groups
- The survey is ready to be administered to a representative sample of the general population

For peer review only

For peer review only - http://bmjopen.bmj.com/site/about/guidelines.xhtml

BACKGROUND

Patients are thought to take a different view of patient safety to healthcare professionals. (1) They tend to view safety in terms of the overall balance of benefit and harm over time whereas healthcare professionals often see high quality healthcare occasionally punctuated by safety incidents and adverse events.(2) Furthermore patients hold may different opinions about how to improve patient safety (3, 4) or different priorities to clinicians, for example identifying psychological and emotional harm rather than technical errors.(5) Involving patients in identifying errors and reducing harm occurs in secondary care (6) but patient reported outcomes can show poor concordance between patients and clinicians, for example, in reporting adverse symptom events in the context of drug safety.(7) Nonetheless patients are thought to be capable of reporting medical errors accurately. (6, 8) Involving patients is advocated as a way to improve safety (9) and this approach would be facilitated through patients and professionals having an understanding each other's expectations and priorities.

Studies that quantify patient safety problems in primary care are uncommon and incidence estimates from record review or incident reporting by clinicians range from less than 1 to 24 per 100 consultations or record review.(10-12) The National Reporting and Learning System (NRLS) in England and Wales records patient safety incidents reported by healthcare professionals; only 1% of these reports originate from primary care (13) which likely reflects under-reporting.(14, 15) Still fewer studies have quantified patient safety problems in primary care from the patient's perspective. (16) A 2013 European survey of the UK public reported that 43% of respondents felt that it was "likely" that patients could be harmed by non-hospital healthcare, an increase from 37% in 2009.(17) In Norway a population-level survey found that the patient-reported lifetime probability of ever experiencing an adverse event was 10%, of which around two thirds of respondents attributed the cause of their event as their general practitioner (GP).(4) In Spain a telephone survey of patients estimated that around 7% of patients experienced a self-reported adverse event during a 1 year period. (18) A USA practice-based website observed an incidence rate of patient-reported adverse events of 1.4% over 2 years.(19) Data from the UK is sparse; this may be partly due to the lack of a valid and reliable instrument to make a comprehensive measurement of safety in primary care.(20) The PREOS-PC should help to address this knowledge gap.(21, 22)

Although it is acknowledged that patients tend to take a different view to professionals (1-2) most research into patient safety is initiated by clinicians with patients invited to contribute. We choose to take an alternative approach whereby the study design was conceived, designed and implemented by a team of three members of the public and one researcher with primary care professionals being invited to contribute later. Previous work has shown that patient-initiated surveys can provide meaningful feedback and guide improvements. (23) Our aim was to design a survey asking about potentially-harmful preventable-problems occurring in UK primary care in partnership with the Greater Manchester Primary Care Patient Safety Translational Research Centre Research User Group, (GMPSTRC RUG) a public and patient involvement (PPI) group.(24) Specifically we aimed to:

1. co-design (with PPI partners) and test a survey asking about problems occurring in primary care that caused, or had the potential to cause, preventable harm as perceived by patients

For peer review only - http://bmjopen.bmj.com/site/about/guidelines.xhtml

2. pilot the survey to examine the usefulness and overall quality of the information collected with respect to describing the patient-perceived problems, the primary care service involved, how the problem was discussed (if it was) and how it might have been prevented.
3. compare the opinions of the survey respondents, members of the public and primary care clinicians as to the likelihood the patient-reported scenario describes a potentially-harmful preventable-problem.

METHODS

Designing and piloting of the survey (Aim 1)

Our main aim was to design a survey asking about problems occurring in primary care that caused, or had the potential to cause, preventable harm as perceived by patients that was easily understood and free from jargon. Currently there is no well-established terminology for asking such a question.(8) The process began with a discussion between three members of the GMPSTRC RUG (AD, JB, CG) and one academic researcher (SJS). Questions used in previous surveys addressing a similar question (4, 17-19) were shared among the project team and used to generate several candidate questions. These questions were then discussed privately among the project team's friends and family and within the project team (SJS, AD, JB, CG). The discussion was facilitated by making the candidate questions available online. After two iterations of this process the survey (Box 1 & Box A in online Appendix 1) was piloted online through newsletters or group mailings of several PPI and public engagement networks during November and December 2015. These networks were the associate GMPSTRC RUG, the Public Programmes team at Central Manchester Foundation Trust, the Citizen Scientist project, the Primary Care Research in Manchester Engagement Resource, North West People in Research Forum and HelpBeatDiabetes volunteers (Details of these groups and networks are provided in Box B in online Appendix 1).

The first question (Q1 Box 1) was taken from the English GP patient survey in order to compare the overall level of confidence and trust in their GP among the survey respondents with that across England.(25) The second question (Q2 Box 1) is the main screening question, those responding negatively to Q2 (*i.e.* not experienced a preventable-problem) were directed to a more specific question with a list of commonly understood patient safety events (Q10 Box A, online Appendix 1). If this prompted recognition of experiencing a potentially-harmful preventable-problem they were returned to Q4 (Box1). The rationale behind this approach was that the screening question (Q2 Box 1) should be non-leading and encourage the respondents to describe their preventable-problems through the subsequent questions without the suggestion that inevitably occurs following a list of possible potentially-harmful preventable-problems. However if the respondent did not believe that they had experienced a potentially-harmful preventable-problem then the prompt question (Q10, Box 1) would ensure that this was the case and also test the sensitivity of Q2 (Box 1). The option to answer on behalf of a friend or relative was offered to those who had not a personal experience to report. This was to ensure sufficient responses to adequately test the questionnaire but also to discourage respondents from answering with another person's experience as their own. Respondents were also asked whether they worked or volunteered in the healthcare profession and to comment on the ease of completion of the questionnaire.

For peer review only - http://bmjopen.bmj.com/site/about/guidelines.xhtml

Coding of reported events (Aims 2&3)

Type of problem (Aim 2)

The nature of the problem in each described scenario was coded at face value, *i.e.* as the patient described without further interpretation, by one author (SJS) and checked by a second author (JA for dental scenarios, PB for all other scenarios). A bottom-up (inductive) approach was used to identify similar topics which were coded then cross-matched to an existing taxonomy for errors in general practice (26, 27) (Table A, online Appendix 1). All the new codes matched the existing taxonomy within the higher two levels and the medication-related scenarios were coded to a finer level (Table B, online Appendix 1).

Likelihood the scenario described a potentially-harmful preventable-problem (Aim 3)

Five GPs, one general dental practitioner and seven members of the public estimated the likelihood that, in their opinion, each patient-described scenario was a potentially-harmful preventable-problem. Brief biographies of the coders are provided in Table C, online Appendix 1. Some examples of the information provided to the coders are shown in boxes 1-23 in online Appendix 2 and consisted of the responses to Q5 to Q9 (Box 1). They were not given any demographic information or the patient's estimate of the impact on their health (Q4, Box 1). Coders were asked to score each scenario from very likely (5) to definitely not (1) in response to the question "*How likely do you think it is the patient was correct in thinking that their health might be worsened, or actually was made worse, because of a mistake or a problem in primary care that could have been prevented?*" Coders could also respond "insufficient information", "Don't know" and give free text feedback (Table D, Appendix 1). The clinician scores were used to categorise the scenarios in to groups with higher or lower estimated likelihoods that they were a potentially-harmful preventable-problem as below.

- Higher threshold - Median score of 5 ("very likely or certain") or 4 ("probably") or at least one score of 5 ("very likely or certain")
- Lower threshold - Median score of 3 ("possibly") or at least one score of 4 ("probably" or higher)
- All other scenarios – Median score below 3 ("possibly") and zero scores above 3 ("possibly")

Statistical analysis

Simple cross tabulations were used to describe the data and a binary logistic regression model was used to explore whether particular types of patient were more likely to perceive a potentially-harmful preventable-problems *e.g.* by demographics or their opinions. Comparisons between demographics and outcomes for the respondents and the UK (or England) population were made using a χ^2 test. All analyses were done using Stata 14.

Public and Patient Involvement (PPI)

PPI was central to this co-design study and was provided through the GMPSTRC RUG (24) and other PPI networks (Box B, online Appendix 1). The study was conceived, designed, implemented and analysed by a team of three members of the public (AD, CG, JB) and one researcher (SJS). At the outset the researcher presented the existing literature on this topic to the PPI members of the

For peer review only - http://bmjopen.bmj.com/site/about/guidelines.xhtml

research team who then co-designed the first draft of the survey which was tested through the PPI members' personal contacts. The piloting of the survey was through existing PPI networks as listed in Box B, online Appendix 1. The scoring of the questions as to the likelihood they described a potentially-harmful preventable-problem was undertaken by 7 members of the public, 2 of whom had no previous experience in PPI (as well as 5 GPs and 1 general dental practitioner as described in Table C, online Appendix 1). These findings will be disseminated to all the PPI groups that contributed to the pilot study and the authors will forward these results to their personal contacts who contributed to the questionnaire design.

Ethical approval was granted by the University of Manchester Ethics Committee 2 (Approval 15372).

RESULTS

The survey design (Aim 1)

The involvement of the PPI partners in the survey design had a profound impact on the piloted version of the survey. Professional researchers may have focussed more on asking questions in a way that forces the responses in to categories but the PPI partners were more concerned that respondents should have the freedom to express themselves and the categorisation should occur during the analysis. They themselves had often completed surveys where there was no appropriate option in the categorical responses. We did not find any of the previous approaches (4, 17-19) suitable for this survey and chose to design a new question. The best option was felt to be an open question with a prompt question for individuals who did not recognise the concept of a preventable potentially-harmful event. Another point of debate was whether we should ask initially about a "problem" then ask if it was "preventable" in a second question. The difficulty with simply asking about a "problem" is that most patients visit their GP because they have a health problem therefore we thought it was more practical to focus immediately on the concept of a preventable-problem encapsulated in a single phrase with a back-up question to ensure it was indeed preventable.

Ease of use of the survey (Aim 1)

Over 250 respondents provided free text feedback on the survey, 200 comments reported that the questionnaire was easy to complete and understand and just one comment described the survey as complex. Most of the remaining comments expressed the desire to be able to provide more information, *e.g.* more than one event or report for a relative or as a carer (reporting on behalf of another person was excluded for events occurring more than 12 months ago) and 13 comments actually provided this unrequested information. Nobody used the "Do not understand the question" option as their response to Q2 Box1. A few respondents found it difficult to find a suitable option to describe their pattern of use of primary care or their role as a worker or volunteer in healthcare. Demographic information was not provided by 83 (13%) respondents, possibly due to lack of clarity about the end of the survey since they completed all other questions.

Summary statistics (Aim 2)

In total 977 members of the public accessed the online pilot survey and 638 (65%) completed the survey during October and November 2015. The majority of respondents were recruited through the HelpBeatDiabetes group (533, 84%, Box B in online Appendix 1). A flow chart of respondents

For peer review only - http://bmjopen.bmj.com/site/about/guidelines.xhtml

through the survey is shown in Fig A in online Appendix 1, 223/638 (35%) of respondents reported ever experiencing a potentially-harmful preventable-problem in primary care of which 132 occurred within the past 12 months (21%). 62 (10%) of these problems were not identified through the initial screening question (Q2) but required prompting through Q10 (Box 1). A further 18 potentially-harmful preventable-problems involving friends or relatives where the respondent was present and occurred in the last 12 months were reported 13/418 (3%, Fig B, online Appendix 1).

Characteristics of the respondents (Aim 2)

The majority of respondents (592, 93%) had confidence and trust in the GP seen at their last appointment similar to the 2016 England proportion of 92% (Q1, Box1 &Table 1). Respondents were older than the UK generally, more likely to work or volunteer in the healthcare sector and tended to use primary care more frequently (Table 1). Older respondents and those working or volunteering in the healthcare sector were no more likely to report a potentially-harmful preventable-problem occurring within the last 12 months but those using primary care more frequently were more likely to report a problem (Table 2). There was a high response from healthcare professionals or volunteers (30% of respondents compared to approximately 3% of the UK adult population, Table 1) but they were no more likely to report a preventable problem than non-healthcare workers/volunteers (35%, $P\chi^2$=0.28).

The nature of the potentially-harmful preventable-problems (Aim 2)

The types of patient-reported scenarios and their categorisation following clinician review are shown in Figure 1. Medication-related problems were most frequently reported type of problem and also more likely to be ranked as a potentially-harmful problem by clinicians, as were communication problems. The type of scenario categorised according to whether it arose from the open-ended screening question (Q2) or prompted through the list of potential problems (Q10) is shown in Figures C&D, online Appendix 1. Scenarios describing problems with appointments, accessing healthcare or loss of test results were more likely to arrive via the prompt question suggesting that patients did not see these as a potentially harmful problem in the first instance. The majority of potentially-harmful preventable-problems in the past 12 months occurred in general practice (73%, Table 3) and pharmacy (5%, Table 3).

The patient's response to the potentially-harmful preventable-problem (Aim 2)

Around half the respondents had not discussed their problem with anybody working in primary care (51%, Table 3). The most common reasons for not discussing the problem were being unable to find a primary care professional with whom to discuss the problem (31%, Table 3) or they did not feel comfortable with discussing their concerns (24%, Table 3). The respondent's suggestions for ways to prevent the problem from happening are summarised in Table 4. The most frequently suggestions were that clinicians should involve the patient more fully in the healthcare process (*i.e.* listen to the patient and trust their judgement more) and be up to date with, and apply, the most recent information about the patient's condition (*i.e.* take in to account all of the patient's information - their medical history and results and letters).

Likelihood the patient-reported scenario described a potentially-harmful preventable-problem (Aim 3)

For peer review only - http://bmjopen.bmj.com/site/about/guidelines.xhtml

Generally the members of the public assigned a higher probability to the likelihood that the patient-described scenario was a potentially-harmful preventable-problem compared with clinicians (Fig 2, Table 5). In 89/108 (82%) scenarios the median score for the PPI researchers was higher than for the clinicians and for 38 (35%) scenarios the PPI median score was 2 or more points higher in a 5 point scale. Following clinician review 3% of the respondents were judged to have “probably” experienced potentially-unsafe preventable-problem during the past 12 months and 11% as “possibly” (using higher and lower thresholds described in Table 5). Scenarios described by healthcare professionals or volunteers were significantly more likely to be categorised as a potentially-harmful preventable-problem following to clinician review using both the lower (9% vs 16%, $P\chi^2$=0.01) and higher threshold (2% vs 6%, $P\chi^2$=0.004). Examples of the patient-reported scenarios with higher clinician rankings are shown in boxes 1-15, online Appendix 2 and those with greatest disagreement between members of the public and clinicians in boxes 16 to 23, online Appendix 2.

DISCUSSION

We have designed and tested a survey to measure the frequency of occurrence of potentially-harmful preventable-problems in primary care and found it to be well understood and acceptable to patients. The open-ended questions (Q6 to Q9, Box 1) led to patient-described scenarios that mapped well to an existing taxonomy designed and used by clinicians and researchers (Tables A&B, online Appendix 1, 26, 27). This implies agreement between clinicians, researchers and patients in identifying the characteristics of a potentially-harmful problem. Furthermore, the use of an open-ended screening question (Q2, Box 1) to ensure that any problems unique to the patient perspective were identified did not find additional new types of problem. However, the open-ended question elicited more problems related to communication and medication suggesting that the public are more likely to view these as safety problems than problems related to appointments and referrals or investigations (Fig C&D online Appendix 1) in agreement with clinicians who were more likely to rank these types of scenarios as potentially harmful. The observation that members of the public were generally more likely to rank the scenarios as a potentially-harmful preventable-problem than clinicians (Fig 2) is important. It is important that primary care not only is safe but that it is perceived as safe by patients.

Strengths and weaknesses of the study

We believe that our survey captures the true patient perspective due to the involvement of members of the public as research partners through data acquisition to analysis and reporting in a co-designed study. By the use of a simple non-leading screening question we encouraged respondents to express their own perspective on what constituted a potentially-harmful preventable-problem rather than directing them towards existing definitions. To ensure that we did not miss any problems we followed up with a prompt that encouraged respondents to think in terms of the traditional view of patient safety problems. Furthermore our survey goes further than describing and counting the frequency of occurrence of potentially-harmful preventable-problems and provides information about how patients dealt with the problem and how it could have been prevented that offers insight in to ways to reduce the frequency of their occurrence. The absence of a link between practices and the patients allows for responses that might not occur if this survey were administered through the individual’s practice. The main weakness of the study is the self-selection of the respondents who were older and tended to use primary care more frequently. More

For peer review only - http://bmjopen.bmj.com/site/about/guidelines.xhtml

frequent users of primary care were more likely to report a problem but age was not associated with the likelihood of reporting a problem. Our bench marking question (Q1, Box1) showed that the respondents were similar to the English GP patient survey (25) in terms of their level of confidence and trust in their GP and not a group with a more negative attitude towards primary care as might have happened given the nature of the survey. We also acknowledge that, by design, this study is totally from the patient perspective. We aim to provide insight into the patient's perspective and not to imply that one or the other point of view is more important but rather there are differences in perceptions that need to be understood and reconciled.

Strengths and weaknesses in relation to other studies

Our finding that 35% of respondents perceived that they had experienced a potentially-harmful problem in in their lifetime is consistent with a European survey (43% of UK respondents felt that it was "likely" that patients could be harmed by non-hospital healthcare).(17) This study offers some insight in to the type of concerns that might underlie this apparent lack of confidence in primary care. A face to face interview in family practice waiting rooms in the USA reported that 16% of respondents believed a physician had made a mistake in their care.(28) The types of problem and patient responses to the problem are similar to those that have been described qualitatively (1, 22) but we have taken this a step further by quantifying their frequency of occurrence and other descriptors of the problem from the patient's perspective. In this small study we did not find that patients were particularly likely to attribute blame to individual members of staff as has been observed previously (3, 4), perhaps partly due to the high proportion of respondents working or volunteering in healthcare.

Unanswered questions and future research

Our finding that 21% of respondents perceived that they had experienced a potentially-harmful problem in the last 12 months, and the corresponding proportion following clinician review of 3% (higher threshold) to 11% (lower threshold) may well reflect the self-selected nature of the study population and needs to be validated in a large population level survey. We anticipate that a population level survey would be fruitful since this approach yielded a number of patient-described scenarios that were amenable to further analysis including coding by clinicians. The high response to this pilot survey by healthcare professionals and volunteers probably reflects the population invited to complete the survey as well as an interest in this topic. It is likely that these respondents are better at articulating their potentially-harmful problem given the higher ranking given by clinicians to scenarios originating from healthcare professionals. Healthcare professionals are an educated and accessible group with the expectations of a patient but also an understanding of the healthcare system who could provide a valuable resource for learning about preventable-problems in primary care. Further work is also needed to understand and reconcile the differences between members of the public and clinicians' perceptions of a potentially-harmful problem. In 1997 Professor Berwick stated "The ultimate measure by which to judge the quality of a medical effort is whether it helps patients (and their families) as they see it. Anything done in health care that does not help a patient or family is, by definition, waste, whether or not the professions and their associations traditionally hallow it." If this tenet still holds then we suggest there is a real need to influence patient's expectations and beliefs about primary care.

For peer review only - http://bmjopen.bmj.com/site/about/guidelines.xhtml

Acknowledgements: The authors would like to express their thanks and appreciation for the work done by the Mary Aldred, Gitanjali Holt, Manoj Mistry, Carole Bennett and Lindsey Brown in coding the patient-described scenarios.

This work was supported by the NIHR grant number gmpstrc-2012-1

Funding: This study was funded by the National Institute for Health Research (http://www.nihr.ac.uk) through the Greater Manchester Primary Care Patient Safety Translational Research Centre (NIHR GM PSTRC), grant number gmpstrc-2012-1. The views expressed are those of the author(s) and not necessarily those of the NHS, the NIHR or the Department of Health. The funders had no role in study design, data collection and analysis, decision to publish, or preparation of the manuscript.

Competing interests: All authors have completed the ICMJE uniform disclosure form at www.icmje.org/coi_disclosure.pdf (available on request from the corresponding author) and declare no support from any organisation for the submitted work; no financial relationships with any organisations that might have an interest in the submitted work in the previous three years, no other relationships or activities that could appear to have influenced the submitted work.

Copyright statement: the Corresponding Author has the right to grant on behalf of all authors and does grant on behalf of all authors, an exclusive licence on a worldwide basis to the BMJ Publishing Group Ltd to permit this article (if accepted) to be published in BMJ editions and any other BMJPGL products and sublicences such use and exploit all subsidiary rights, as set out in our licence.

Contributors: SJS, AD, JB and CG conceived and designed the study. SJS, AD, JB, CG, AE, PB, JA, DT, SL, AD, RD and NM analysed the data. SJS wrote the manuscript, and is guarantor. AD, JB, CG, AE, PB, JA, DT, SL, AD, RD, NM and SC edited the manuscript.

Data sharing: Raw data (coded only) is available from jill.stocks@manchester.ac.uk

Ethics approval: University of Manchester Ethics Committee 2 (Approval 15372)

Figure legends

Fig 1. Numbers of patient-perceived problems occurring in the last 12 months categorised according to the patient's description with clinician ranking as to the likelihood it is a potentially-harmful preventable problem (Table 5).

Footnote to Figure 1: See Tables A&B, online Appendix 1 for details of coding; A coded to 2 levels, B medication problems coded to 3 levels, C coded to 1 level

Figure 2. Median estimates as to the likelihood that the patient describes a potentially-harmful preventable-problem occurring in the last 12 months by six clinicians and seven members of the public

For peer review only - http://bmjopen.bmj.com/site/about/guidelines.xhtml

Box 1. Brief summary of questionnaire – see Box A, online Appendix 1 for full version of survey.

Q1. Did you have confidence and trust in the GP you saw or spoke to at your last appointment? (benchmarking question)

Q2. When using primary care have you ever felt concerned that your health might be worsened, or actually was made worse, because of a mistake or a problem that could have been prevented?

If yes to Q2

Q3. How long ago did the mistake or preventable problem happen?

Q4. How did this affect your health?

Q5. Which primary care service were you using when the mistake or preventable problem occurred?

Q6. Briefly describe the mistake or problem and how it happened

Q7. Could the mistake or problem have been avoided? If so how?

Q8. Were you able to talk about the mistake or problem with anybody working in the primary care service? If not –why not?

Q9. If you discussed the mistake or problem with somebody working in primary care please describe their job or role

Q10. In the list below are some examples of **preventable** problems[1] that might happen when using primary care. Has ***anything similar*** happened to you ***in the last 12 months***? *If yes go to Q4*

[1]See Q10 Box A, online Appendix 1 for list of preventable problems

For peer review only

Table 1. Characteristics of survey respondents

Variable	All respondents n=638	Ever had problem n=223	Had problem in last 12 months n=132	UK population comparator
GP satisfaction	missing=0	missing=0	missing=0	English GP patient survey(25)
Yes definitely	384 (60%)	81 (36%)	55 (42%)	64%
Yes, to some extent	208 (33%)	110 (49%)	52 (39%)	28%
No, not at all	39 (6%)	27 (12%)	21 (16%)	4%
Don't know / can't say	7 (1%)	5 (2%)	4 (3%)	3%
Worked or volunteered in healthcare	missing=92	missing=40	missing=19	NHS workforce[1]
Yes	166 (30%)	64 (35%)	41 (36%)	3%
Gender	missing=87	missing=38	missing=16	ONS mid-2015 estimates[2]
Female	268 (49%)	106 (57%)	63 (54%)	51%
Age	missing=85	missing=37	missing=15	ONS mid-2015 estimates[2]
16 to 34 years	42 (8%)	22 (12%)	11 (9%)	31%
35 to 54 years	143 (26%)	54 (29%)	34 (29%)	34%
55 to 64 years	162 (29%)	59 (32%)	31 (27%)	14%
65 to 74 years	170 (31%)	44 (24%)	32 (27%)	12%
Over 75 years	36 (7%)	7 (4%)	9 (8%)	9%
Last primary care contact	missing=88	missing=39	missing=14	English GP patient survey(25)
Within last week	169 (31%)	65 (35%)	48 (41%)	84% within last 12 months
Within last month	248 (45%)	79 (43%)	47 (40%)	
Within last 12 months	121 (22%)	34 (18%)	20 (17%)	
Over 12 months ago	12 (2%)	6 (3%)	3 (3%)	15%
Usual primary care usage	missing=88	missing=40	missing=17	
At least once a month	181 (33%)	73 (40%)	52 (45%)	-
At least once per 6 months	285 (52%)	79 (43%)	45 (39%)	-
Once per 12 months or less	84 (15%)	31 (17%)	18 (16%)	-

[1]http://content.digital.nhs.uk/searchcatalogue?productid=24139&topics=1%2fWorkforce%2fStaff+numbers&sort=Relevance&size=10&page=1#top

[2]https://www.ons.gov.uk/peoplepopulationandcommunity/populationandmigration/populationestimates/bulletins/annualmidyearpopulationestimates/latest

For peer review only

1 Table 2. Prevalence of respondents reporting a potentially-harmful preventable problem within the
2 last 12 months and unadjusted and adjusted odds ratios estimated by logistic regression

Respondent characteristics n=638	Frequency – all reported n=132	Unadjusted odds ratio (OR) – all reports	Adjusted[1] OR - all reports	Adjusted[1] OR - after GP review (lower threshold, Table 5)
**Gender (87 missing)**				
male	53/283 (19%)	1 (ref)	1 (ref)	1 (ref)
female	63/268 (24%)	1.3 (0.9 to 2.0)	1.4 (0.9 to 2.2)	1.3 (0.7 to 2.3)
**Age (85 missing)**				
16 to 34 years	11/42 (26%)	1 (ref)	1 (ref)	1 (ref)
35 to 54 years	34/143 (24%)	0.9 (0.4 to 1.9)	0.8 (0.3 to 1.8)	0.8 (0.3 to 2.1)
55 to 64 years	31/162 (19%)	0.7 (0.3 to 1.5)	0.7 (0.3 to 1.5)	0.6 (0.2 to 1.7)
65 to 74 years	32/170 (19%)	0.7 (0.3 to 1.4)	0.6 (0.3 to 1.4)	0.4 (0.2 to 1.2)
Over 75 years	9/36 (25%)	0.9 (0.3 to 2.6)	1.1 (0.4 to 3.2)	0.9 (0.2 to 3.2)
**Last primary care contact (88 missing)**				
Within last week	48/169 (28%)	1 (ref)	1 (ref)	1 (ref)
Within last month	47/248 (19%)	0.6 (0.4 to 0.9)	0.7 (0.4 to 1.1)	0.6 (0.3 to 1.0)
Within last 12 months	20/121 (17%)	0.5 (0.3 to 0.9)	0.6 (0.3 to 1.2)	0.5 (0.2 to 1.3)
Over 12 months ago	3/12 (25%)	0.8 (0.2 to 4.0)	0.9 (0.2 to 4.2)	0.4 (0.0 to 3.9)
**Usual primary care usage (88 missing)**				
At least once a month	52/181 (29%)	1 (ref)	1 (ref)	1 (ref)
At least once per 6 months	45/285 (16%)	0.5 (0.3 to 0.7)	0.6 (0.3 to 0.9)	0.5 (0.3 to 0.9)
Once per 12 months or less	18/84 (21%)	0.7 (0.4 to 1.2)	0.8 (0.4 to 1.6)	0.7 (0.3 to 1.8)
**Works or volunteers in healthcare (92 missing)**				
No	72/380 (19%)	1 (ref)	1 (ref)	1 (ref)
Yes	41/166 (25%)	1.4 (0.9 to 2.2)	1.3 (0.8 to 2.1)	1.5 (0.9 to 2.7)

3

4 [1]adjusted for gender, age, last primary care contact, usual primary care usage, works or volunteers in healthcare

For peer review only - http://bmjopen.bmj.com/site/about/guidelines.xhtml

Table 3. The patient's response to their perceived potentially-harmful preventable-problem and the primary care service involved for problems occurring in the last 12 months

Primary care service	All reported problems	Clinician ranked "possibly or higher" (Lower threshold)
All services	132	71
GP surgery	97 (73%)	61 (86%)
Out of hours care/A&E/ambulance	4 (3%)	1 (1%)
Walk in clinic	2 (2%)	0
Dental surgery	4 (3%)	1 (1%)
Pharmacy	7 (5%)	6 (8%)
Community or district nursing	4 (3%)	0
Opticians	2 (2%)	1 (1%)
Mental health services	1 (1%)	0
missing	11 (8%)	1 (1%)
**Did you discuss the problem with primary care staff?**		
All respondents	132	71
Yes – discussed with primary care staff	56 (42%)	42 (59%)
No – did not discuss with primary care staff	67 (51%)	29 (41%)
missing	9 (7%)	0
**Reason not discussed with primary care staff**		
All not discussing problem	67	29
Did not feel comfortable to discuss the problem	16 (24%)	8 (28%)
Could not find anybody with whom to discuss the problem	21 (31%)	10 (34%)
Unconcerned about the problem	7 (10%)	5 (17%)
Did not notice the problem at the time (or too ill)	11 (16%)	4 (14%)
Other	5 (7%)	2 (7%)
missing	7 (10%)	0
**Profession of discussant**		
All discussing problem	56	42
GP	28 (50%)	19 (45%)
Practice manager	5 (9%)	5 (21%)
Receptionist	2 (4%)	1 (2%)
Practice nurse	6 (11%)	5 (12%)
Pharmacist or dispenser	7 (13%)	7 (17%)
General dental practitioner	2 (4%)	1 (2%)
Dietician	1 (2%)	1 (2%)
Missing	5 (9%)	3 (7%)
**Role of discussant in patient's care**		
Member of staff directly involved	23 (41%)	16 (38%)
Another member of staff at same institution	25 (45%)	20 (48%)
Above unclear	8 (14%)	6 (14%)

For peer review only - http://bmjopen.bmj.com/site/about/guidelines.xhtml

Table 4. Patient suggestions as to how the potentially-harmful preventable problem might have been prevented

**How could it be prevented?**	All reported problems n=132	Clinician ranked "possibly or higher" (Lower threshold) n=71
**1. More resources - all**	**14 (11%)**	**3 (4%)**
1.1 Quicker access to primary care	7 (5%)	2 (3%)
1.2 More thorough and quicker investigations	2 (2%)	1 (1%)
1.3 Fewer demands on primary care – more staff or fewer patients	1 (1%)	0
1.4 More time with clinicians for treatment and diagnosis	2 (2%)	0
1.9 Provision of resources to manage long term conditions	1 (1%)	0
1.10 Provision of patient travel service for routine appointments	1 (1%)	0
**2. Improved communication and involvement of patients**	**26 (20%)**	**18 (25%)**
1.1 Listen to the patient and trust their judgement more	21 (16%)	15 (21%)
1.2 Tell patients about their diagnosis, test results, changes in medication or loss of results	3 (2%)	1 (1%)
1.3 Improve communication between staff (within or outside primary care)	2 (2%)	2 (3%)
**3. Better organisation and administration**	**17 (13%)**	**10 (14%)**
3.1 Follow up referrals and appointments to ensure they happen, be consistent in sending routine reminders	10 (8%)	3 (4%)
3.2 Log in or process results as soon as received to avoid loss	1 (1%)	1 (1%)
3.3 Keep the notes up to date, well-organised, safe and ensure information is transcribed accurately	5 (4%)	5 (7%)
3.4 Keep a record of the location of equipment	1 (1%)	1 (1%)
**4. Improved prescribing systems**	**18 (14%)**	**17 (24%)**
4.1 More checks on prescribing and dispensing	8 (6%)	8 (11%)
4.2 Check repeat prescriptions carefully, especially for transcribing errors	8 (6%)	7 (10%)
4.3 Use medication reviews and IT clinical decision support systems	2 (2%)	2 (3%)
**5. Better clinical practice**	**19 (14%)**	**10 (14%)**
5.1 Take in to account all the patient's information - their medical history and results and letters	13 (10%)	7 (10%)
5.2 Address the patient's problem in some way – patients can feel their problem is being ignored	5 (4%)	2 (3%)
5.3 Act on advice from other clinicians and test results	1 (1%)	1 (1%)
**6. Staff training**	**11 (8%)**	**7 (10%)**
6.1 More informed and better trained staff	11 (8%)	7 (10%)
**Other responses**	**27 (20%)**	**6 (8%)**
• Don't know/missing	21 (16%)	3 (4%)
• Problem was due to an individual member of staff	2 (2%)	1 (1%)
• Prescribe right, better, different, more, less medicine	1 (1%)	0
• Better organisation	1 (1%)	0
• Laboratory procedures were the problem	2 (2%)	2 (3%)

For peer review only - http://bmjopen.bmj.com/site/about/guidelines.xhtml

Table 5. Categorisation of patient-perceived potentially-harmful preventable problems occurring in the last 12 months following review by clinicians and members of the public

Group label	Threshold criteria	Clinician scores n=132	Members of the public scores n=132
1. Higher threshold	Median score of "very likely or certain" or "probably" or at least one score of "very likely or certain"	18 (14%)	87 (66%)
2. Lower threshold	Median score of "possibly" or at least one score of "probably" or higher	71 (54%)	104(79%)
3. Any possibility	At least one score of "unlikely" or higher	106 (80%)	109 (83%)
4. No problem	All scores "definitely not" or not-coded	1 (1%)	0
5. Not-coded	Insufficient information for coding by all coders	25 (19%)	23 (17%)

For peer review only

References

1. Rhodes P, Campbell S, Sanders C. Trust, temporality and systems: how do patients understand patient safety in primary care? A qualitative study. Health Expectations. 2016;19(2):253-63.

2. Vincent C, Amalberti R. Safer Healthcare: Strategies for the Real World. Springer, 2016. http://www.springer.com/gb/book/9783319255576 Accessed 04/04/17

3. Blendon RJ, DesRoches CM, Brodie M, Benson JM, Rosen AB, Schneider E, et al. Views of practicing physicians and the public on medical errors. N Engl J Med. 2002;347(24):1933-40.

4. Hotvedt R, Forde OH. Doctors are to blame for perceived medical adverse events. A cross sectional population study. The Tromso Study. BMC health services research. 2013;13:46.

5. Kuzel AJ, Woolf SH, Gilchrist VJ, Engel JD, LaVeist TA, Vincent C, et al. Patient reports of preventable problems and harms in primary health care. Ann Fam Med. 2004;2(4):333-40.

6. The Health Foundation. Evidence scan: Involving patients in improving safety. January 2013. http://www.health.org.uk/sites/health/files/InvolvingPatientsInImprovingSafety.pdf Accessed 04/04/17

7. Basch E. The missing voice of patients in drug-safety reporting. N Engl J Med. 2010;362(10):865-9.

8. King A, Daniels J, Lim J, Cochrane DD, Taylor A, Ansermino JM. Time to listen: a review of methods to solicit patient reports of adverse events. Qual Saf Health Care. 2010;19(2):148-57.

9. Longtin Y, Sax H, Leape LL, Sheridan SE, Donaldson L, Pittet D. Patient participation: current knowledge and applicability to patient safety. Mayo Clinic proceedings. 2010;85(1):53-62.

10. Sandars J, Esmail A. The frequency and nature of medical error in primary care: understanding the diversity across studies. Family practice. 2003;20(3):231-6.

11. Panesar SS, deSilva D, Carson-Stevens A, Cresswell KM, Salvilla SA, Slight SP, et al. How safe is primary care? A systematic review. BMJ Quality & Safety. 2016;25(7):544-53.

12. Michel P, Brami J, Chaneliere M, Kret M, Mosnier A, Dupie I, et al. Patient safety incidents are common in primary care: A national prospective active incident reporting survey. PLoS One. 2017;12(2):e0165455.

13. NHS Improvement. National quarterly data on patient safety incident reports. September 2016. https://improvement.nhs.uk/resources/national-quarterly-data-patient-safety-incident-reports-september-2016/ Accessed 04/04/17

14. Hutchinson A, Young TA, Cooper KL, McIntosh A, Karnon JD, Scobie S, et al. Trends in healthcare incident reporting and relationship to safety and quality data in acute hospitals: results from the National Reporting and Learning System. Qual Saf Health Care. 2009;18(1):5-10.

15. NHS England. General practice patient safety reporting form launched. 26th February 2015. https://www.england.nhs.uk/2015/02/gp-patient-safety-reporting/ Accessed 04/04/17

For peer review only - http://bmjopen.bmj.com/site/about/guidelines.xhtml

16. The Health Foundation. Evidence Scan: Improving safety in primary care. November 2011. http://www.health.org.uk/sites/health/files/ImprovingSafetyInPrimaryCare.pdf Accessed 04/04/17

17. European Commission. Special Eurobarometer 411 Patient Safety and Quality of Care 2014. DOI:10.2772/33467 https://ec.europa.eu/health/sites/health/files/patient_safety/docs/ebs_411_sum_en.pdf Accessed 04/04/17

18. Mira JJ, Nebot C, Lorenzo S, Perez-Jover V. Patient report on information given, consultation time and safety in primary care. Qual Saf Health Care. 2010;19(5):e33.

19. Wasson JH, MacKenzie TA, Hall M. Patients use an internet technology to report when things go wrong. Qual Saf Health Care. 2007;16(3):213-5.

20. Ricci-Cabello I, Goncalves DC, Rojas-Garcia A, Valderas JM. Measuring experiences and outcomes of patient safety in primary care: a systematic review of available instruments. Family practice. 2015;32(1):106-19.

21. Ricci-Cabello I, Avery AJ, Reeves D, Kadam UT, Valderas JM. Measuring Patient Safety in Primary Care: The Development and Validation of the "Patient Reported Experiences and Outcomes of Safety in Primary Care" (PREOS-PC). Ann Fam Med. 2016;14(3):253-61.

22. Ricci-Cabello I, Saletti-Cuesta L, Slight SP, Valderas JM. Identifying patient-centred recommendations for improving patient safety in General Practices in England: a qualitative content analysis of free-text responses using the Patient Reported Experiences and Outcomes of Safety in Primary Care (PREOS-PC) questionnaire. Health Expectations. 2017.

23. Southwick FS, Cranley NM, Hallisy JA. A patient-initiated voluntary online survey of adverse medical events: the perspective of 696 injured patients and families. BMJ Qual Saf. 2015;24(10):620-9.

24. Stocks SJ, Giles SJ, Cheraghi-Sohi S, Campbell SM. Application of a tool for the evaluation of public and patient involvement in research. BMJ Open. 2015;5(3).

25. NHS England. GP Patient Survey – National summary report. January 2016. http://gp-survey-production.s3.amazonaws.com/archive/2016/January/January+2016+National+Summary+Report.pdf Accessed 04/04/17

26. Dovey SM, Meyers DS, Phillips RL, Jr., Green LA, Fryer GE, Galliher JM, et al. A preliminary taxonomy of medical errors in family practice. Qual Saf Health Care. 2002;11(3):233-8.

27. Makeham MA, Dovey SM, County M, Kidd MR. An international taxonomy for errors in general practice: a pilot study. Med J Aust. 2002;177(2):68-72.

28. Kistler CE, Walter LC, Mitchell C, Sloane PD. Patient perceptions of mistakes in ambulatory care. Archives of Internal Medicine. 2010;170(16):1480-7.

For peer review only - http://bmjopen.bmj.com/site/about/guidelines.xhtml

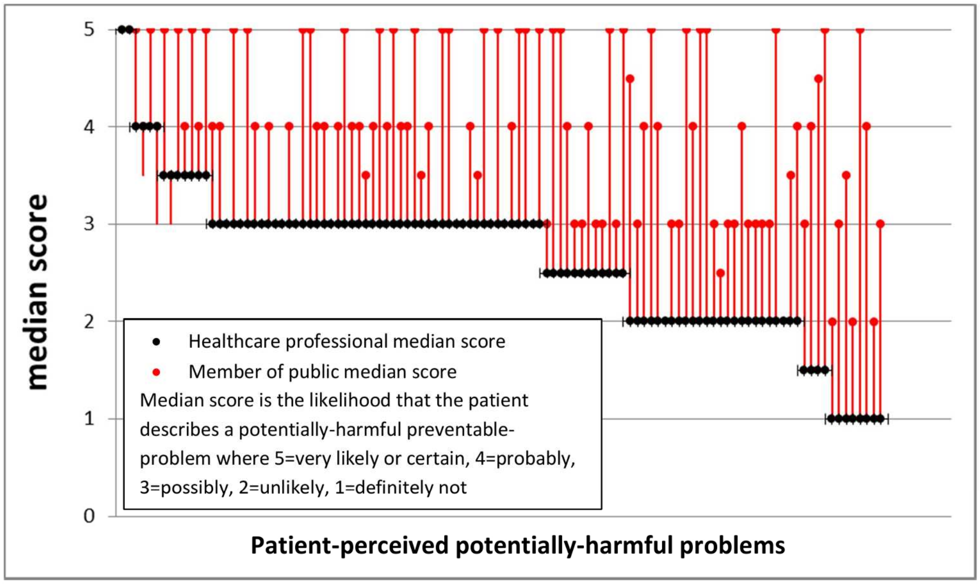

Figure 2. Median estimates as to the likelihood that the patient describes a potentially-harmful preventable-problem occurring in the last 12 months by six clinicians and seven members of the public

170x102mm (300 x 300 DPI)

For peer review only

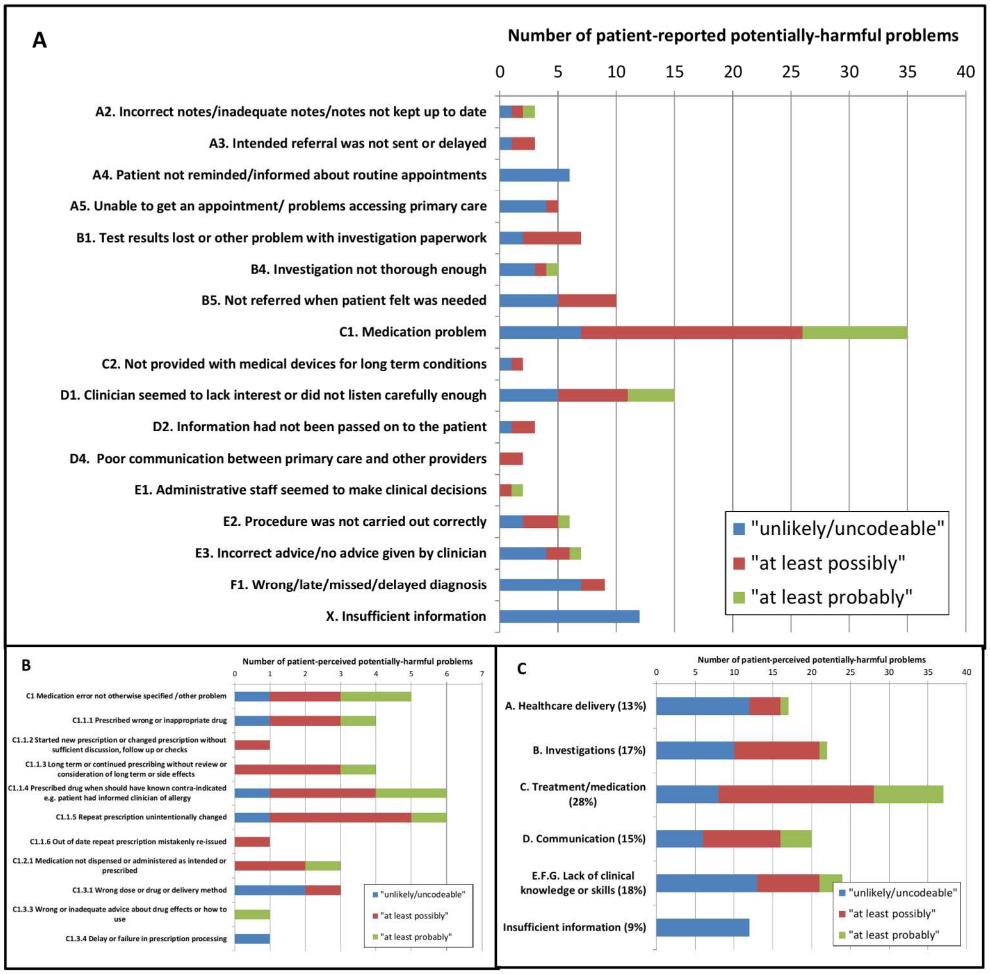

Footnote to Figure 1: See online Appendix 2 for details of coding; A coded to 2 levels, B medication problems coded to 3 levels, C coded to 1 level. ├ ┬ Fig 1. Numbers of patient-perceived problems occurring in the last 12 months categorised according to the patient's description with clinician ranking as to the likelihood it is a potentially-harmful preventable problem (Table 3).├ ┬

199x195mm (300 x 300 DPI)

Appendix 1. Details of survey, coding systems, public and clinician contributors and supplementary results

Box A. Pilot survey administered online November and December 2015

Q1. Did you have confidence and trust in the GP you saw or spoke to at your last appointment?

Response options: Yes, definitely, Yes, to some extent, No, not at all, Don't know / can't say

Q2. When using primary care have you ever felt concerned that your health might be worsened, or actually was made worse, because of a mistake or a problem that could have been prevented?

Response options: Yes, No- *go to Q10,* Do not understand the question- *go to Q10*, Don't know / can't remember- *go to Q10*

Q3. How long ago did the mistake or preventable problem happen?

Response options: Within the last 12 months, More than 12 months ago- *go to Q10,* Can't remember- *go to Q10*

Q4. In your opinion did this experience

Response options: Make your health worse, Not certain but it might have made your health worse, Could have made your health worse if you had not noticed the problem, Delayed your treatment but had no effect on your health, Not affect you, or your health, Other, please explain

Q5. Which primary care service were you using when the mistake or preventable problem occurred?

Response options: GP surgery, Out of hours care, Walk in clinic, Dental, Pharmacy, Community or district nursing, Ambulance, Opticians, Other- please specify

Q6. Briefly describe the mistake or problem and how it happened

Response options: free text

Q7. Could the mistake or problem have been avoided? If so how?

Response options: free text

Q8. Were you able to talk about the mistake or problem with anybody working in the primary care service?

Response options: Yes, Yes had the opportunity but did not feel comfortable to discuss the mistake or problem, No I could not find anybody with whom I could discuss the mistake or problem, No I was not concerned about the problem, No I did not notice the mistake or problem at the time, I was too distressed to discuss the mistake or problem, Other or don't know - please describe

Q9. If you discussed the mistake or problem with somebody working in primary care please describe their job or role

Response options: free text

Q10. In the list below are some examples of preventable problems that might happen when using primary care. Has anything similar happened to you in the last 12 months? Please check as many as applicable or "NONE OF BELOW"

NONE OF BELOW
Wrong or late diagnosis
Not referred for further investigation when needed
Test results being lost or mixed up
Receiving the wrong medicine or wrong dose
Should not be prescribed the medicine because of another health problem
Should not be prescribed the medicine because of another medication already taking
Poor communication leading to misunderstanding of diagnosis or treatment
Not referred to a specialist when needed

Unclear instructions about treatment<br>Not offering of prevention or screening programmes eg CVD/stroke prevention clinics<br>Failure to recognise or act on vulnerable people's needs eg child abuse, suicide risk or mental health problems<br>Mistake with a procedure eg dental treatment, injection, ear syringing, physiotherapy<br>Failure to notify about recommended vaccinations eg flu, HPV<br>Poor hygiene<br>Unsafe building or premises<br>Any other preventable problem in the last 12 months (in your opinion)<br>Other, please explain below
Q11. Are you male or female? Response options: Male, Female, prefer not to say
Q12. How old are you?<br>Response options: under 16, 16 to 24, 25 to 34, 45 to 54, 55 to 64, 65 to 74, 75 to 84, 85 or older
Q13. When was your last contact with primary care?<br>Response options: Last week, Last month, Last 12 months, Over 12 months ago
Q14. What best describes your usual pattern of use of primary care? Response options: Once per week, Once per 2 weeks, Once per month, Once per 6 months, Once per 12 months or less often
Q15. Are you registered with a GP practice?<br>Response options: Yes, No, I only use walk in centres, Don't know
Q16. Do you work or volunteer in healthcare or healthcare research as a professional, patient, carer or member of the public? (if you are retired answer for your occupation before retirement)<br>Response options: Yes, No
Q17. We are still trying to improve this questionnaire so would be grateful for any feedback about how easy you found the questionnaire to complete? How can it be improved?<br>Response options: free text

Box B. List of public and patient involvement groups used to distribute the pilot survey
Associate Research User Group of the Greater Manchester Primary Care Patient Safety Translational Research Centre http://research.bmh.manchester.ac.uk/primary-care-patient-safety/GetInvolved/
The Primary Care Research in Manchester Engagement Resource http://research.bmh.manchester.ac.uk/PRIMER/about/
HelpBeatDiabetes https://www.researchforthefuture.org/diabetes/
The Nowgen Centre https://research.cmft.nhs.uk/getting-involved/involvement
The Citizen Scientist project http://www.citizenscientist.org.uk/
North West People in Research Forum https://www.northwestpeopleinresearchforum.org/

For peer review only - http://bmjopen.bmj.com/site/about/guidelines.xhtml

Table A. Coding of patient-reported potentially-unsafe scenarios in primary care

**1. Errors in the process of the healthcare delivery system**	
Makeham 2002, Dovey 2002	Common threads reported in this study
1.1. Errors in the process of conducting an administrative task	A1. Administrative problem not otherwise specified
1.1.1. Information filed in wrong place or wrong time	
1.1.2. Unavailability of information that should have been in patients charts<br>1.1.2.1. Entire chart or part of chart could not be accessed when needed<br>1.1.2.2. Care provided was not documented<br>1.1.2.3. Item(s) of information missing from chart	A2. Incorrect notes/inadequate notes/notes not kept up to date
1.1.3. Errors in patient's movement through the healthcare delivery system	A3. Intended referral was not sent or delayed<br>A4. Patient not reminded, informed or assisted to attend regular check-ups or other necessary routine treatments
1.1.4. Errors in the taking and distributing of messages	
1.1.5. Errors in managing appointments for healthcare	A5. Unable to get an appointment/other problems with making appointment<br>A6. Ambulance delayed or did not arrive
**1.2. Errors in the process of investigating a patient's condition**	
1.2.1. Laboratory errors<br>1.2.1.1. Wrong test ordered or test not ordered when appropriate<br>1.2.1.2. Errors in the process of obtaining or processing a laboratory specimen<br>1.2.1.3. Error in the process of physician receiving accurate laboratory results in a timely fashion<br>1.2.1.4. Inappropriate response to an abnormal laboratory result	B1. Test results lost or other problem with investigation paperwork<br>B2. Incorrect interpretation of tests or other investigation results<br>B3. Clinician did not consider patient history sufficiently/did not use patient's notes adequately<br>B4. Investigation not thorough enough<br>B5. Not referred when patient felt was needed
1.2.3. Errors in the processes of other investigations<br>1.2.3.1. Wrong test ordered or test not ordered when appropriate<br>1.2.3.2. Errors in the process of obtaining or processing of other diagnostic investigation<br>1.2.3.3. Error in the process of physician receiving accurate test results of other investigation in a timely fashion<br>1.2.3.4. Inappropriate response to an abnormal result of other investigation	
**1.3. Errors in the process of treating a patient's condition**	
1.3.1. Errors in the process of treating with medications<br>1.3.1.1. Wrong medication or wrong dose of medication ordered or medication not ordered by physician when appropriate<br>1.3.1.2. Error in the process of delivering a medication order or inappropriate medication order by a provider working under physician supervision<br>1.3.1.3. Error in the process of dispensing medication as ordered	C1. Medication problem<br><br>C2. Not provided with medical devices needed to manage long term conditions

For peer review only

For peer review only - http://bmjopen.bmj.com/site/about/guidelines.xhtml

1.3.2. Errors in other treatments	C3. Problem with dental treatment or diagnosis
**1.4. Errors in the process of communication**	
1.4.1. Errors in communication between primary healthcare provider and patients	D1. Clinician seemed to lack interest in the patient's health problem or did not listen carefully enough<br>D2. Information about the patient's health had not been passed on to the patient who felt it should have been<br>D3. Communication problem between patient and primary care staff
1.4.2. Errors in communication between healthcare providers	D4. Problem with communication between primary care and other types of care including secondary care<br>D5. Disagreement between 2 clinicians
**2. Errors arising from lack of clinical knowledge or skills**	
2.1. Errors in the execution of a clinical task<br>2.1.1. Non-clinical staff made the wrong clinical decision<br>2.1.2. Failed to follow standard practice<br>2.1.3. Lacked needed experience or expertise in a clinical task	E1. Administrative staff seemed to make clinical decisions<br>E2. Procedure was not carried out correctly<br>E3. Incorrect advice/no advice given by clinician
**2.2. Errors in diagnosis**<br>2.2.1. Wrong or delayed diagnosis	F1. Wrong/late/missed/delayed diagnosis
**2.3. Wrong treatment decision**	G1. Wrong treatment decision
	H. Other
	X. Not a problem/ insufficient information/refused/don't know

Table B. Level 4 coding of patient-reported potentially-unsafe medication scenarios

Common threads reported in this study grouped as described by Makeham 2002, Dovey 2002
C1 Medication error not otherwise specified /other problem
• **1.3.1.1. Ordering medications (prescribing)**
C1.1.1 Prescribed wrong or inappropriate drug
C1.1.2 Started new prescription or changed prescription without sufficient discussion, follow up or checks
C1.1.3 Long term or continued prescribing without review or consideration of long term or side effects
C1.1.4 Prescribed drug when should have known contra-indicated *e.g.* patient had informed clinician of allergy, adverse reaction or it was in the records
C1.1.5 Repeat prescription unintentionally changed
C1.1.6 Out of date repeat prescription mistakenly re-issued
• **1.3.1.2./1.3.1.3. Implementing or receiving medications (dispensing or issuing)**
C1.2.1 Medication not dispensed or administered as intended or prescribed
• **1.3.1.1/1.3.1.2./1.3.1.3. Ordering, implementing or receiving medications**
C1.3.1 Wrong dose or drug or delivery method
C1.3.2 Being given another patient's drugs or prescription
C1.3.3 Wrong or inadequate advice about drug effects or how to use
C1.3.4 Delay or failure in prescription processing

For peer review only - http://bmjopen.bmj.com/site/about/guidelines.xhtml

Table C. Demographics of clinicians and members of the public reviewing the patient-reported problems and estimating the likelihood the scenarios describes a potentially-harmful preventable-problem occurring in primary care

**Demographics of GP and dentist coders**	**frequency n=6**
**Gender**	
Female	3
Male	3
**Years working as a GP or dentist**	
Less than 15 years	1
15 to 25 years	2
Over 25 years	3
**Current position**	
Partner	4
Retired within last 12 months	2
**Demographics of the members of the public**	**frequency n=7**
**Gender**	
Female	6
Male	1
**Age**	
30 to 39 years	2
40 to 49 years	1
50 to 59 years	2
60 to 69 years	2
**Ethnicity**	
White British	5
British Indian	2
**Years of PPI experience**	
None	2
Less than 1 year	1
1 to 5 years	2
Over 5 years	2
**Further background information**	
PPI reviewer 1. Currently working freelance on education and PPI projects; previously worked in a pastoral role at a college; a lay representative for courses training healthcare scientists.	
PPI reviewer 2. Retired primary school teacher with several long term health conditions; single parent; was a young carer for a parent with a long term condition.	
PPI reviewer 3. Former higher education administrator; current university tutor; patient partner on varied research projects; carer for family members aged 0-100 with physical and/or mental health long term conditions.	
PPI reviewer 4. Currently working as a civil servant and has several long term health conditions.	
PPI reviewer 5. Full-time parent of school age children; previously ten years working in a medical school in an administrative role and 5 years working in the drug and alcohol sector	
PPI reviewer 6. Lay representative for several healthcare-related professional bodies and involved in health research at several universities; family-carer for over 35 years; has had over 6 years of involvement with a mental and community health as a carer	
PPI reviewer 7. Retired university administrator; a parent and carer for elderly parents.	

For peer review only

Table D. Scoring for likelihood that the patient-reported scenario is potentially-harmful preventable-problem

Score	How likely do you think it is the patient was correct in thinking that their health might be worsened, or actually was made worse, because of a mistake or a problem in primary care that could have been prevented? Choose from the options below.
5	Very likely or certain (75-100% confident is a potentially-harmful preventable-problem)
4	Probably (50-74% confident is a potentially-harmful preventable-problem)
3	Possibly (25-49% confident is a potentially-harmful preventable-problem)
2	Unlikely (bottom 25% confident is a potentially-harmful preventable-problem)
1	Definitely not a potentially unsafe event (0% chance is a potentially-harmful preventable-problem)
-	Insufficient information
-	Don't know
-	Other - add text at end of row

For peer review only

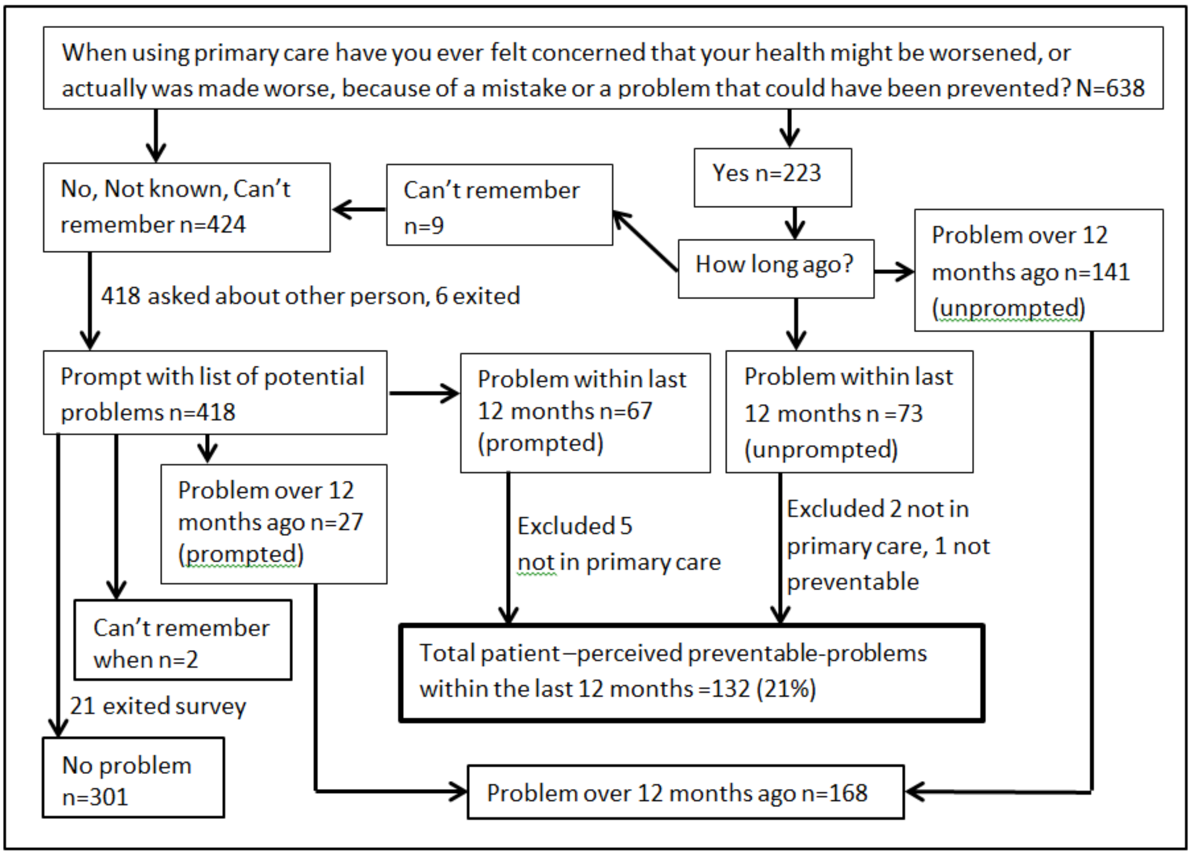

Figure A. Flow chart of participants who reported a potentially-unsafe preventable-problem in primary care through the online pilot survey

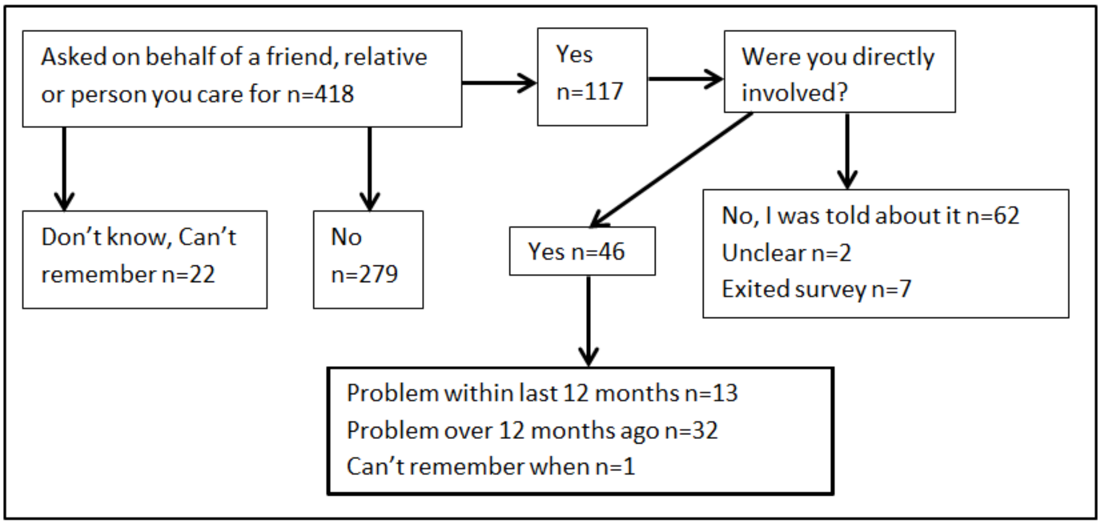

Figure B. Flow chart of participants who reported a potentially-unsafe preventable-problem in primary care on behalf of another person through the online pilot survey

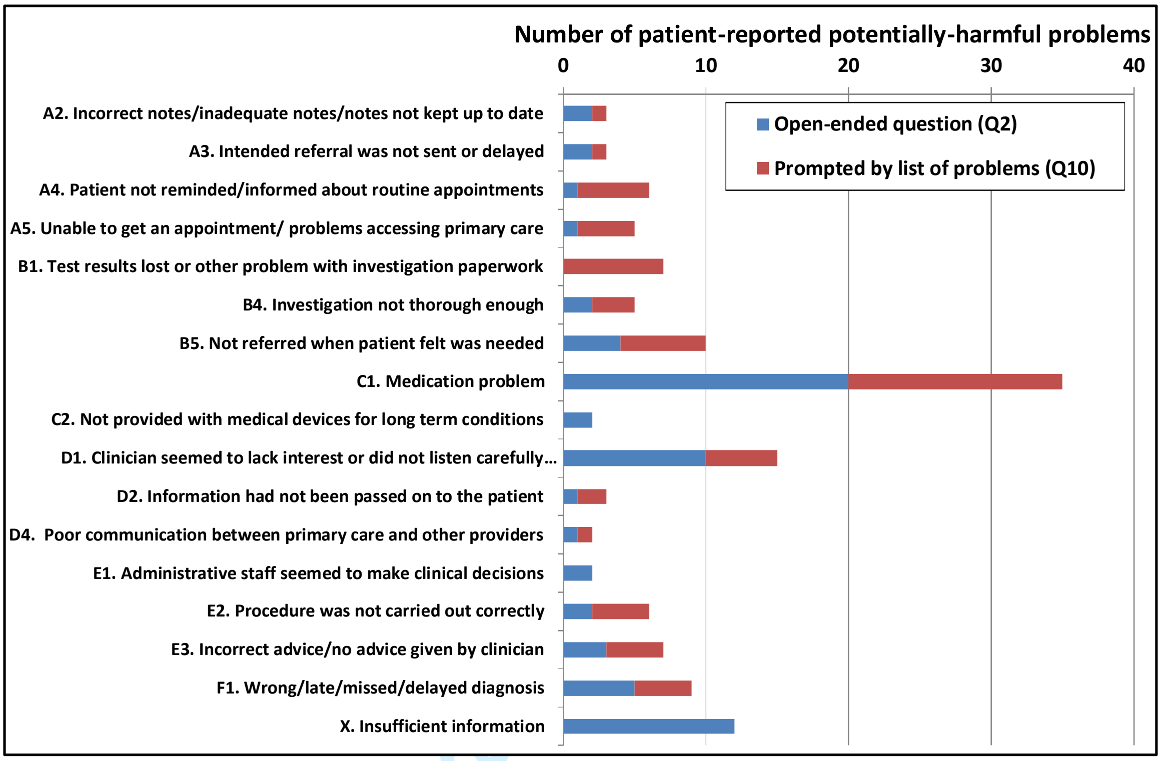

Fig C. Numbers of patient-perceived problems occurring in the last 12 months categorised according to the patient's description (see Table 2) and route through survey *i.e.* originated from open-ended question (Q2) or prompted by list of potential safety problems (Q10)

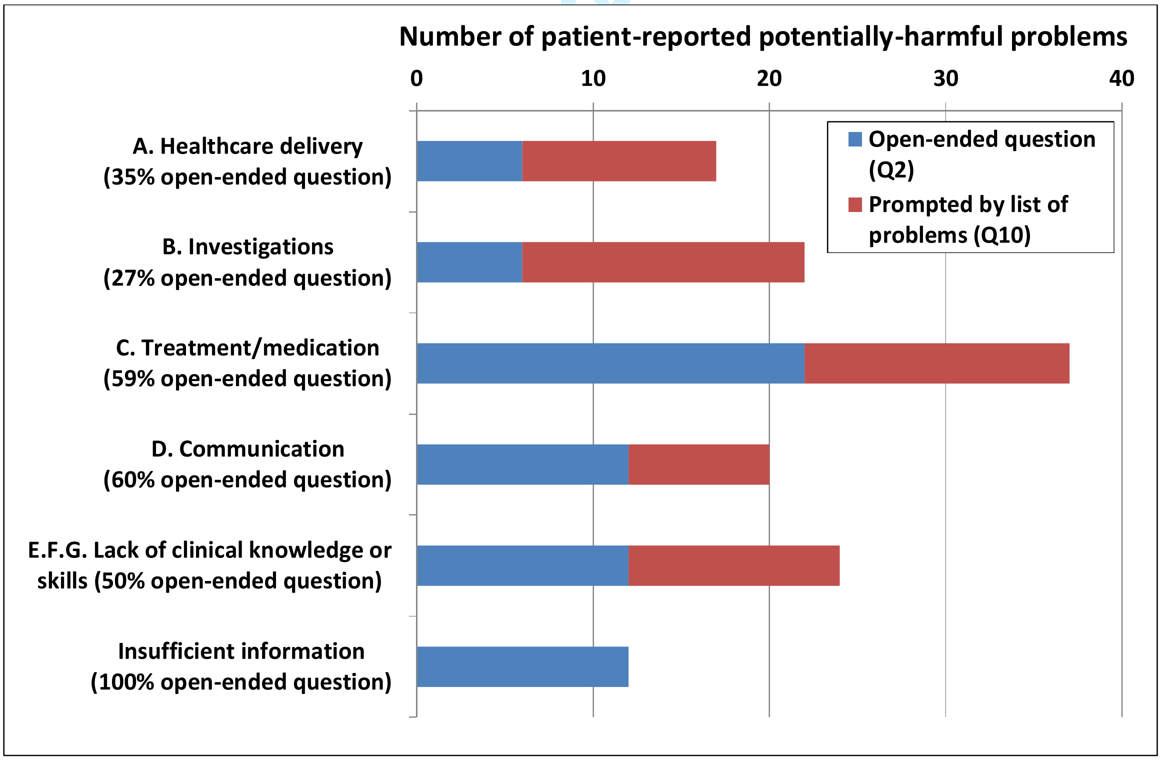

Fig D. Numbers of patient-perceived problems occurring in the last 12 months categorised according to the patient's description coded at a higher level (see Table 2) and route through survey *i.e.* originated from open-ended question (Q2) or prompted by list of potential safety problems (Q10)

Appendix 2. Boxes 1 to 15

Patient reported scenarios occurring during the past 12 months that GPs scored as higher likelihood to be a potentially-unsafe preventable-problem in primary care (median score is higher than "possibly" and at least 2 GPs gave a score or one GP scored "very likely or certain")

Scenario1. GP surgery

Briefly describe the mistake or problem and how it happened. *"Prescription drug, anti-inflammatory for arthritis, caused acute stomach pains & violent vomiting. Repeat prescription for twelve years without any discussion."*

Could the mistake or problem have been avoided? If so how? "*Possible discussion about dangers of continuous taking of prescription drugs, which in the event were stopped after the incident."*

Were you able to talk about the mistake or problem with anybody working in the primary care service? *"No I did not notice the mistake or problem at the time"*

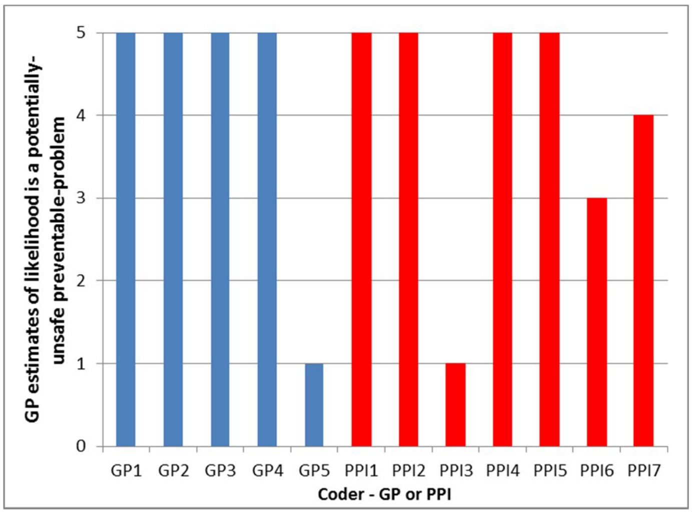

5=very likely or certain, 4=probably, 3=possibly, 2=unlikely, 1=definitely not, 0 = insufficient information or don't know

Patient-reported prospect of harm: suspected your health has been made worse by a problem or error that could have been prevented

Patient-perspective problem-type code: C1.1.3 Long term or continued prescribing without review or consideration of long term or side effects

Scenario2. GP surgery

Briefly describe the mistake or problem and how it happened. *"Insulin type was changed by specialist but previous insulin prescribed by GP as notes had not been updated"*

Could the mistake or problem have been avoided? If so how? "*Yes GP notes should have been updated with new medication"*

Were you able to talk about the mistake or problem with anybody working in the primary care service? *"Practice manager resolved the problem and apologised"*

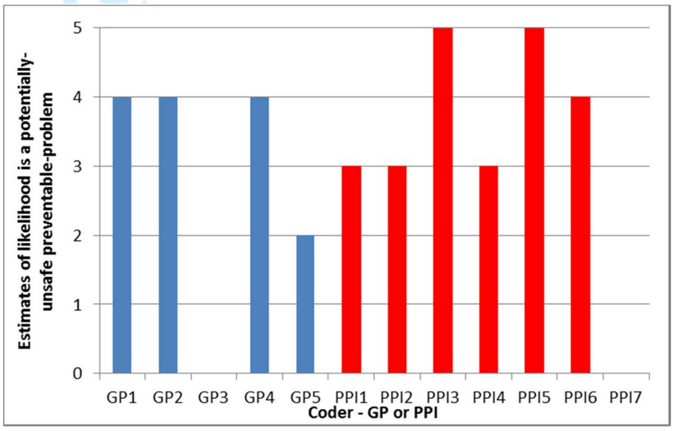

5=very likely or certain, 4=probably, 3=possibly, 2=unlikely, 1=definitely not, 0 = insufficient information or don't know

Patient-reported prospect of harm: prompted via Q10 (Box 1 main paper)

Patient-perspective problem-type code: A2. Incorrect notes/inadequate notes/notes not kept up to date; C1.1.6 Out of date repeat prescription mistakenly re-issued

For peer review only - http://bmjopen.bmj.com/site/about/guidelines.xhtml

Scenario3. GP surgery

Briefly describe the mistake or problem and how it happened. *"Two out of three Doctors not listening to what I was asking; April I had two big bleeds from my Penis, Doctor 1 did a test and gave antibiotics. Went to 2nd Doctor for Diabetic check and told him of problem - nothing except another test come back in ten days. Went to the third doctor who said the test didn't show anything but when I mentioned my feelings about a problem, he look and said yes you do have a problem. In 2 weeks I was in having tests and 3 operations for cancer."*

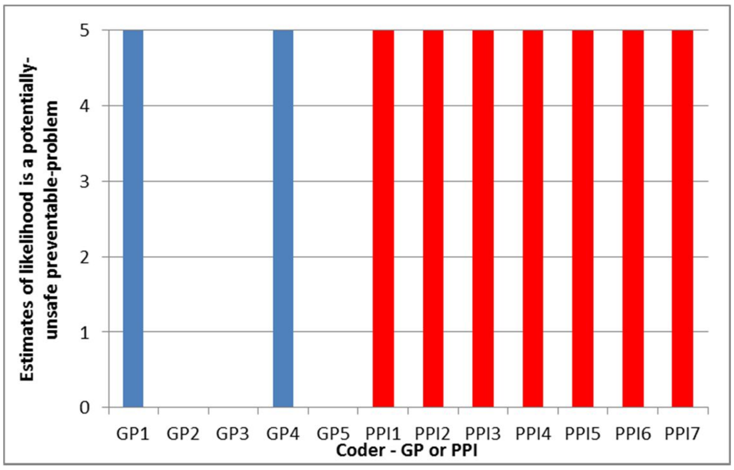

5=very likely or certain, 4=probably, 3=possibly, 2=unlikely, 1=definitely not, 0 = insufficient information or don't know

Could the mistake or problem have been avoided? If so how? "*Listen to me"*

Were you able to talk about the mistake or problem with anybody working in the primary care service? *"No, I could not find anybody with whom I could discuss the mistake or problem (The third doctor was amazing with me. He said to keep in touch and if I had any problems to ring him and he still wants me to ring him after my three operations.)"*

Patient-reported prospect of harm: suspected your health has been made worse by a problem or error that could have been prevented

Patient-perspective problem-type code: D1. Clinician seemed to lack interest in the patient's health problem or did not listen carefully enough; F1. Wrong/late/missed/delayed diagnosis

Scenario4. GP surgery

Briefly describe the mistake or problem and how it happened. *"Changed diabetes medication to an alternative which my notes from 1980's should show I respond badly to"*

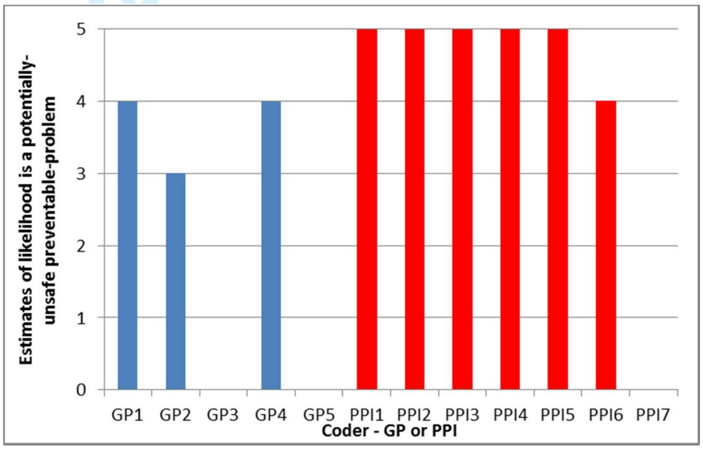

5=very likely or certain, 4=probably, 3=possibly, 2=unlikely, 1=definitely not, 0 = insufficient information or don't know

Could the mistake or problem have been avoided? If so how? "*Read the notes on every medication change but unfortunately that is unrealistic under the time restrictions on GP's. Put early notes on-line and flag medication allergies/problems."*

Were you able to talk about the mistake or problem with anybody working in the primary care service? *"Yes, my own GP who had returned from holiday"*

Patient-reported prospect of harm: suspected your health has been made worse by a problem or error that could have been prevented

Patient-perspective problem-type code: C1.1.4 Prescribed drug when should have known contra-indicated *e.g.* patient had informed clinician of allergy, adverse reaction or it was in the records

Scenario5. GP surgery

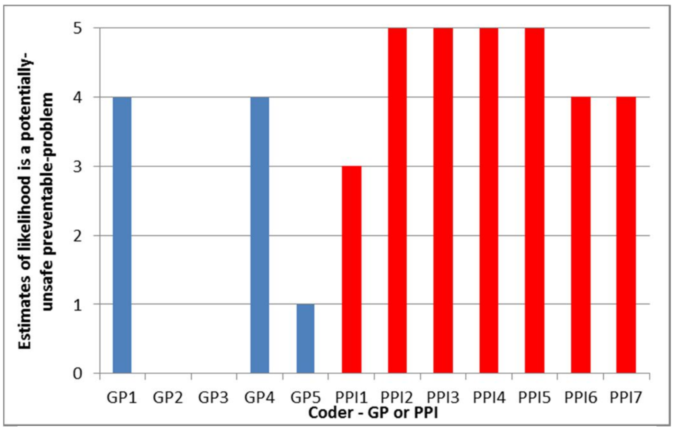

5=very likely or certain, 4=probably, 3=possibly, 2=unlikely, 1=definitely not, 0 = insufficient information or don't know

Briefly describe the mistake or problem and how it happened. *"Told the GP the medication was making my hair fall out & he kept me on it for another 3 months. I had to see another GP to get him to change my medication. In the meantime I have lost 3/4 of my hair. Not sure if it will ever grow back."*

Could the mistake or problem have been avoided? If so how? "*yes, by the GP listening to what I was saying."*

Were you able to talk about the mistake or problem with anybody working in the primary care service? *"Yes, GP"*

Patient-reported prospect of harm: suspected your health has been made worse by a problem or error that could have been prevented

Patient-perspective problem-type code: D1. Clinician seemed to lack interest in the patient's health problem or did not listen carefully enough; C1.1.3 Long term or continued prescribing without review or consideration of long term or side effects

Scenario6. GP surgery

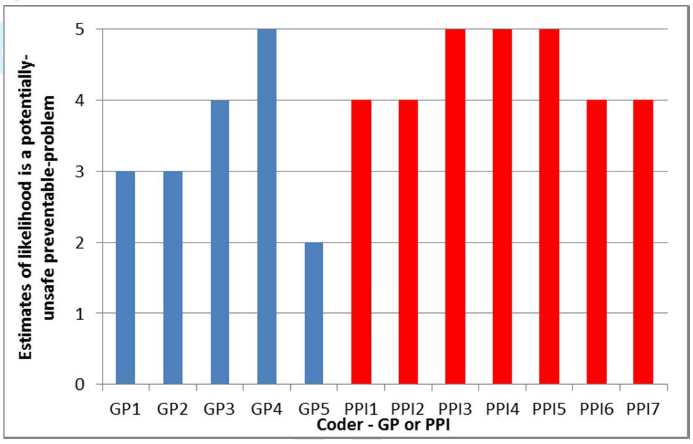

5=very likely or certain, 4=probably, 3=possibly, 2=unlikely, 1=definitely not, 0 = insufficient information or don't know

Briefly describe the mistake or problem and how it happened. *"Successfully treated for prostate cancer 2006 but suffered some loss of sexual performance; Viagra recommended BUT I take isosorbide nitrate for a following heart attack; the two are contradictory and could produce further heart problems. A routine diabetes check-up at which the sexual problem was discussed saw an automatic prescribing of Viagra; obviously without reference to my medical records."*

Could the mistake or problem have been avoided? If so how? "*Read the medical notes."*

Were you able to talk about the mistake or problem with anybody working in the primary care service? *"No; I felt I was going to cause trouble"*

Patient-reported prospect of harm: prompted via Q10 (Box 1 main paper)

Patient-perspective problem-type code: C1.1.1 Prescribed wrong or inappropriate drug

For peer review only

Scenario7. GP surgery

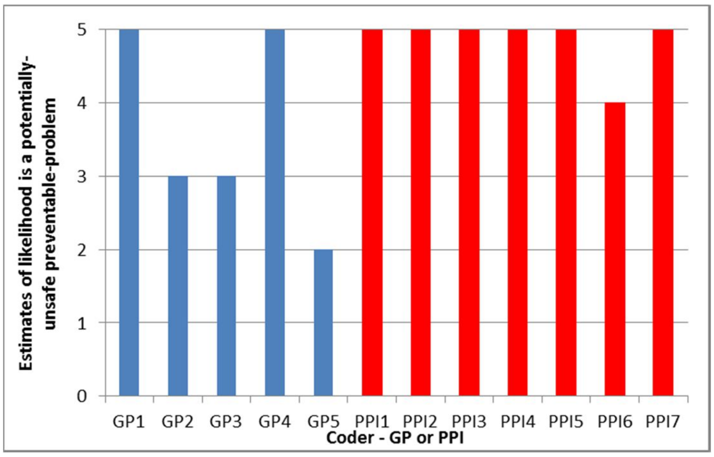

5=very likely or certain, 4=probably, 3=possibly, 2=unlikely, 1=definitely not, 0 = insufficient information or don't know

Briefly describe the mistake or problem and how it happened. *"I was given steroids for a chest infection but not alerted to the fact they make your sugars go massively high! Within a few hours I was high and not able to bring them down, fearing a DKA I headed for the hospital to correct a very easily avoidable issue. I also attended my GP 6 years ago to be given strong antacids for pain in my stomach that was actually a DKA I was admitted to hospital a few hours later! The GP never even suggested it could be linked to my diabetes and as it was my first DKA I had no idea that's how they can feel"*

Could the mistake or problem have been avoided? If so how? "*Both could have been avoided The steroids - if the prescribing nurse had considered my diabetes I'd have been given proper advice as to how to deal with them as a diabetic or given different meds. The DKA simple questions or explanation as to how DKAs can present would have made me family and the doctor realise I was in trouble."*

Were you able to talk about the mistake or problem with anybody working in the primary care service? *"I wrote a letter to the surgery concerning the steroids anonymously to alert them of my concern and the DKA. I was too poorly to even consider seeking correction or explanation"*

Patient-reported prospect of harm: health was actually made worse by a problem or error that could have been prevented

Patient-perspective problem-type code: C1.1.4 Prescribed drug when should have known contra-indicated *e.g.* patient had informed clinician of allergy, adverse reaction or it was in the records; E3. Incorrect advice/no advice given by clinician

Scenario8. GP surgery

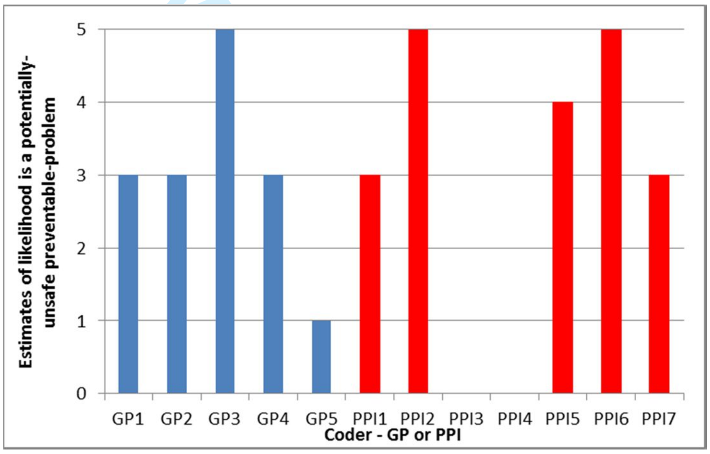

5=very likely or certain, 4=probably, 3=possibly, 2=unlikely, 1=definitely not, 0 = insufficient information or don't know

Briefly describe the mistake or problem and how it happened. *"reception staff making clinical decisions which were at odds with what had been discussed with my GP"*

Could the mistake or problem have been avoided? If so how? "*Yes, reception staff shouldn't be making clinical decisions"*

Were you able to talk about the mistake or problem with anybody working in the primary care service? *"No, had the opportunity but did not feel comfortable to discuss the mistake or problem"*

Patient-reported prospect of harm: suspected your health has been made worse by a problem or error that could have been prevented

Patient-perspective problem-type code: E1. Administrative staff seemed to make clinical decisions

For peer review only

Scenario9. Pharmacist

Briefly describe the mistake or problem and how it happened. *"I was given a medicine belonging to somebody else as part of my monthly repeat prescription"*

Could the mistake or problem have been avoided? If so how? "*More care and attention when checking"*

Were you able to talk about the mistake or problem with anybody working in the primary care service? *"Yes, pharmacist"*

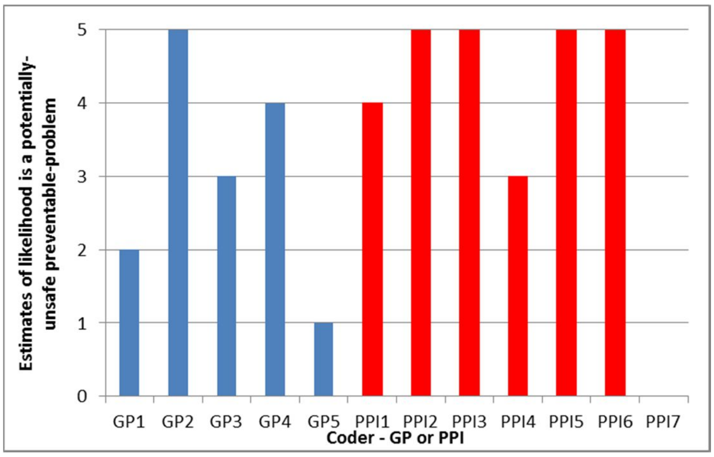

5=very likely or certain, 4=probably, 3=possibly, 2=unlikely, 1=definitely not, 0 = insufficient information or don't know

Patient-reported prospect of harm: prompted via Q10 (Box 1 main paper)

Patient-perspective problem-type code: C1.3.3 Wrong or inadequate advice about drug effects or how to use

Scenario10. GP surgery

Briefly describe the mistake or problem and how it happened. *"Poor diabetic annual review, foot check not correctly done just tested my foot pulses and nothing else"*

Could the mistake or problem have been avoided? If so how? "*Better training of staff"*

Were you able to talk about the mistake or problem with anybody working in the primary care service? *"No, had the opportunity but did not feel comfortable to discuss the mistake or problem"*

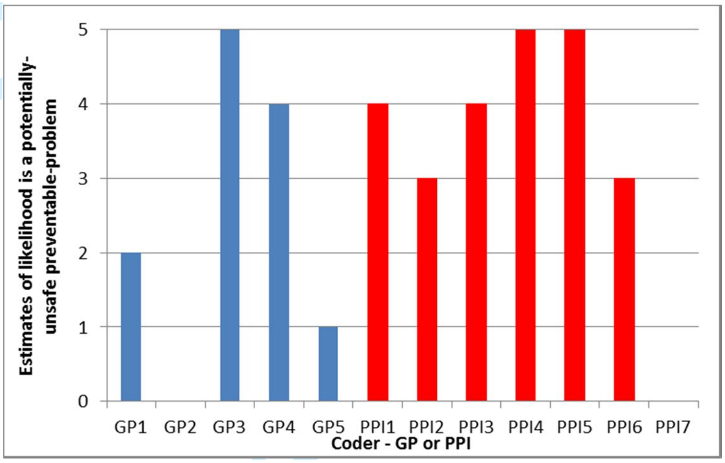

5=very likely or certain, 4=probably, 3=possibly, 2=unlikely, 1=definitely not, 0 = insufficient information or don't know

Patient-reported prospect of harm: suspected your health has been made worse by a problem or error that could have been prevented

Patient-perspective problem-type code: E2. Procedure was not carried out correctly

For peer review only

Scenario11. GP surgery

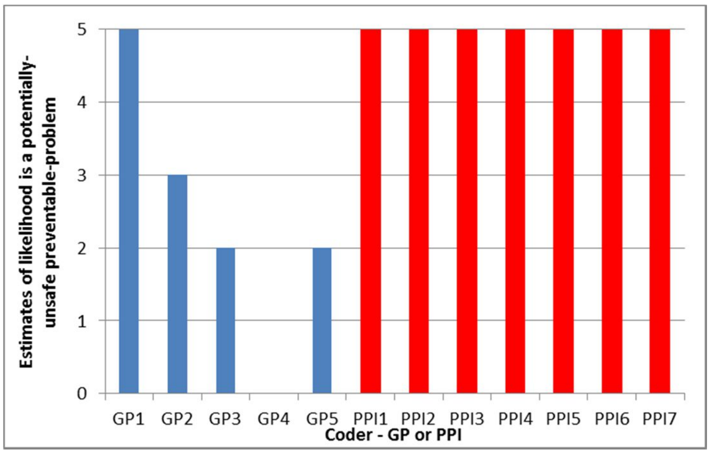

5=very likely or certain, 4=probably, 3=possibly, 2=unlikely, 1=definitely not, 0 = insufficient information or don't know

Briefly describe the mistake or problem and how it happened. *"Prior to a pain killing injection into my knee, I asked the GP who suggested the injection AND the GP who carried out the injection whether, as someone living with Type 1 diabetes, it would have any effect on my blood glucose levels. On both occasions, I was given an unequivocal No . In the event, within a few hours of the injection, my blood glucose rose significantly and remained high for several days. I felt unable to eat anything for 24 hours while I took on more and more insulin in order to bring my glucose levels down - I did not want to go to sleep that night simply because of the massive amount of insulin in my system."*

Could the mistake or problem have been avoided? If so how? "*Yes. I feel that both GPs should have a knowledge about the side effects of drugs they prescribe, administer and recommend."*

Were you able to talk about the mistake or problem with anybody working in the primary care service? *"No I could not find anybody with whom I could discuss the mistake or problem"*

Patient-reported prospect of harm: your health has been made worse by a problem or error that could have been prevented

Patient-perspective problem-type code: E3. Incorrect advice/no advice given by clinician

Scenario12. GP surgery

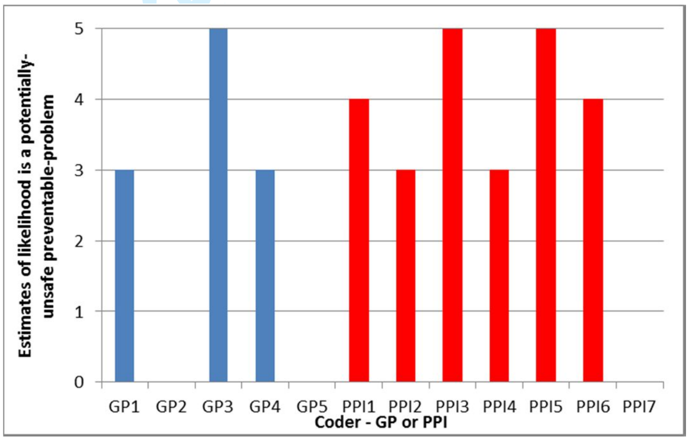

5=very likely or certain, 4=probably, 3=possibly, 2=unlikely, 1=definitely not, 0 = insufficient information or don't know

Briefly describe the mistake or problem and how it happened. *"GP completely overlooked symptoms and prescribed antibiotic after antibiotic without investigation or referral"*

Could the mistake or problem have been avoided? If so how? "*Yes by listening to history of complaints, carrying out appropriate tests instead of just giving antibiotics"*

Were you able to talk about the mistake or problem with anybody working in the primary care service? *"No I did not notice the mistake or problem at the time"*

Patient-reported prospect of harm: prompted via Q10 (Box 1 main paper)

Patient-perspective problem-type code: D1. Clinician seemed to lack interest in the patient's health problem or did not listen carefully enough; F1. Wrong/late/missed/delayed diagnosis

For peer review only - http://bmjopen.bmj.com/site/about/guidelines.xhtml

Scenario13. GP surgery

Briefly describe the mistake or problem and how it happened. *"Several times prescriptions have been incorrectly issued due to similar names for drugs or the same name with different strengths"*

Could the mistake or problem have been avoided? If so how? "*Yes, by more accurate or double data entry. Now solved by self-request using web systems."*

Were you able to talk about the mistake or problem with anybody working in the primary care service? "Yes, *they did not want to know or seem to care unless a formal complaint was made"*

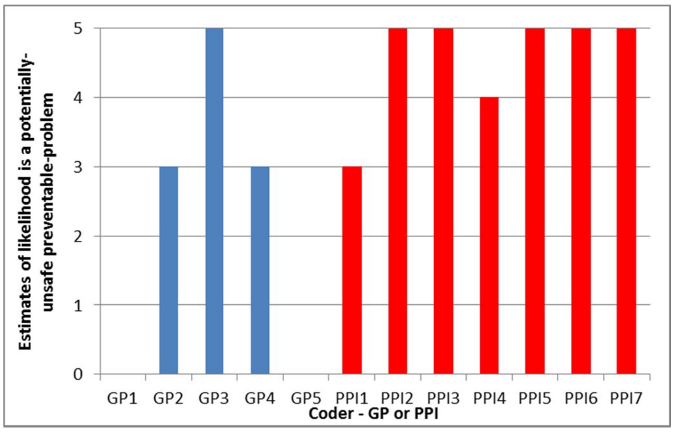

5=very likely or certain, 4=probably, 3=possibly, 2=unlikely, 1=definitely not, 0 = insufficient information or don't know

Patient-reported prospect of harm: your health has been made worse by a problem or error that could have been prevented

Patient-perspective problem-type code: C1.1.5 Repeat prescription unintentionally changed

Scenario14. GP surgery

Briefly describe the mistake or problem and how it happened. *"A simple error occurred with an incorrect prescription. When I tried to bring this to the attention of the receptionist she treated me with disdain and in a challenging manner. She then proceeded to start to read my notes aloud in the public reception area. I felt that this was unacceptable behaviour. When I tried to tackle the receptionist about her behaviour I felt as if I was under threat. It caused me to feel very stressed, frustrated and ill tempered."*

Could the mistake or problem have been avoided? If so how? "*If the receptionist had been willing to listen to what I was saying."*

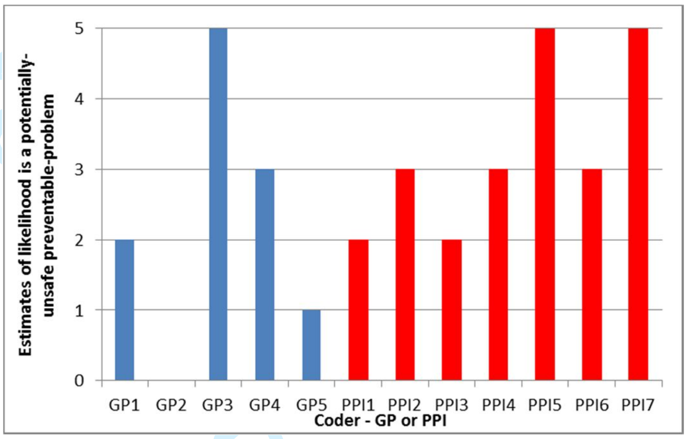

5=very likely or certain, 4=probably, 3=possibly, 2=unlikely, 1=definitely not, 0 = insufficient information or don't know

Were you able to talk about the mistake or problem with anybody working in the primary care service? *"I did speak to a lady who said she was the practice manager but I felt that they were not interested in resolving the problem"*

Patient-reported prospect of harm: suspected your health has been made worse by a problem or error that could have been prevented

Patient-perspective problem-type code: D3. Communication problem between patient and primary care staff; C1 Medication error not otherwise specified /other problem

Scenario15. GP Surgery

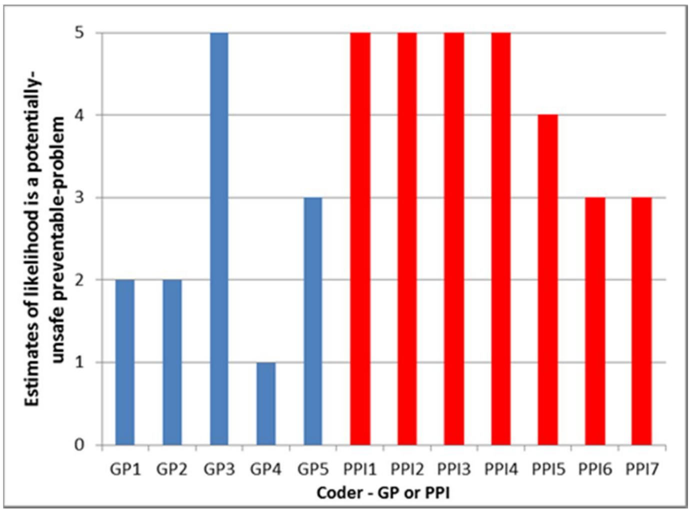

5=very likely or certain, 4=probably, 3=possibly, 2=unlikely, 1=definitely not, 0 = insufficient information or don't know

Briefly describe the mistake or problem and how it happened. *"Went to see GP because I feared the pain in one of my legs may have been Peripheral Artery Disease - hardening of the arteries, having had a (non-blood) relative who suffered from this and subsequently died - of a heart attack. Oh yes, said the GP, well, you will have it won't you? Why? I asked expecting her to say eg because you are a smoker, or maybe my age (65) or something else I wasn't aware of. But what she actually told me was 'Because you are a diabetic!' Whaaat? I exclaimed - you mean ALL diabetics will inevitably get this, and there's no way to prevent it? Yes she said and shrugged. I said 'Thanks for nothing then' and left. Instead I left, came home and went straight on-line to make an appointment with someone more sensible, which I did and after taking my leg/ankle pulses and BPs etc - he chatted to me and said he would refer me for a cardiology consultation at the hospital. This IS what I expected in the first place and now it IS being taken care of***."

Could the mistake or problem have been avoided? If so how? *"By training the GP properly in the first place"*

Were you able to talk about the mistake or problem with anybody working in the primary care service? "? *"I explained to GP 2 But I don't know what if anything was done about it, or how I could find that out."*

Patient-reported prospect of harm: your health has been made worse by a problem or error that could have been prevented

Patient-perspective problem-type code: D1. Clinician seemed to lack interest in the patient's health problem or did not listen carefully enough

For peer review only

Appendix 2. Boxes 16 to 23

Patient reported scenarios occurring during the past 12 months that PPIs scored as higher likelihood to be a potentially-unsafe preventable-problem in primary care compared with GPs

Scenario16. GP Surgery

Briefly describe the mistake or problem and how it happened. *"I had a severe reaction to Atorvastatin after a dose increase so much so that I was almost immobile and took 4 months to recover"*

Could the mistake or problem have been avoided? If so how? *"According to guidelines I should have been on the increased dose - it took a long time to convince the GP that I needed blood tests to find out why I couldn't walk. My GP was very hesitant to admit that I did have a reaction to statins."*

Were you able to talk about the mistake or problem with anybody working in the primary care service? **"***No I could not find anybody with whom I could discuss the mistake or problem. It was not really the GPs fault per se, just took a lot of convincing that there was a problem"*

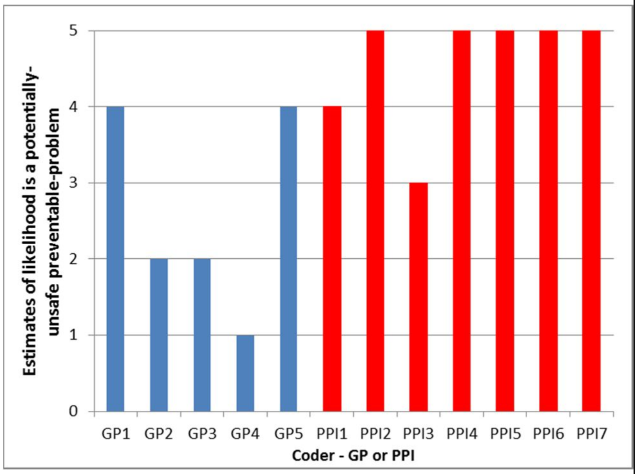

5=very likely or certain, 4=probably, 3=possibly, 2=unlikely, 1=definitely not, 0 = insufficient information or don't know

Patient-reported prospect of harm: health could have been made worse had someone not noticed a problem or error

Patient-perspective problem-type code: C1.1.3 Long term or continued prescribing without review or consideration of long term or side effects

For peer review only

Scenario17. GP Surgery

Briefly describe the mistake or problem and how it happened. *"Doctor kept saying I had vitamin deficiency B1, it turned out I had peripheral neuropathy which is very painful"*

Could the mistake or problem have been avoided? If so how? *"I just needed the proper medication to help"*

Were you able to talk about the mistake or problem with anybody working in the primary care service? *"Just saw another Doctor and she knew straight away what the problem was - she was experienced with Diabetic problems. Yes had the opportunity but did not feel comfortable to discuss the mistake or problem"*

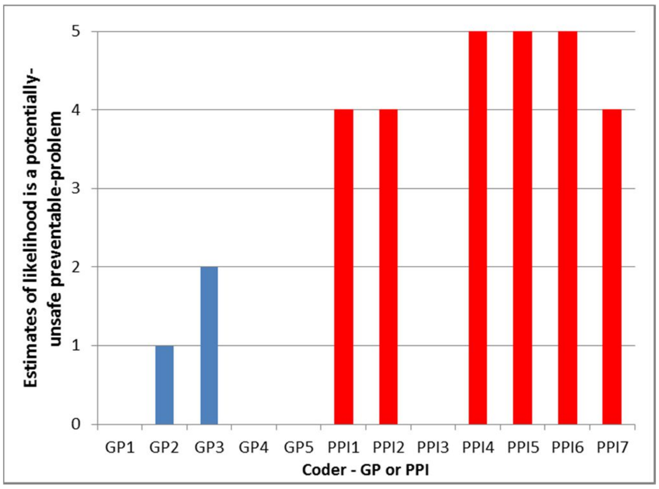

5=very likely or certain, 4=probably, 3=possibly, 2=unlikely, 1=definitely not, 0 = insufficient information or don't know

Patient-reported prospect of harm: prompted via Q10 (Box 1 main paper)

Patient-perspective problem-type code: F1. Wrong/late/missed/delayed diagnosis

For peer review only

Scenario18. GP Surgery

Briefly describe the mistake or problem and how it happened. *"Incapable diabetic doctor trying to take blood out the back of my hand haphazardly, not listening and resulting in me fitting and the student watching having to get help."*

Could the mistake or problem have been avoided? If so how? *"Yes. By listening to me"*

Were you able to talk about the mistake or problem with anybody working in the primary care service? *"No I could not find anybody with whom I could discuss the mistake or problem"*

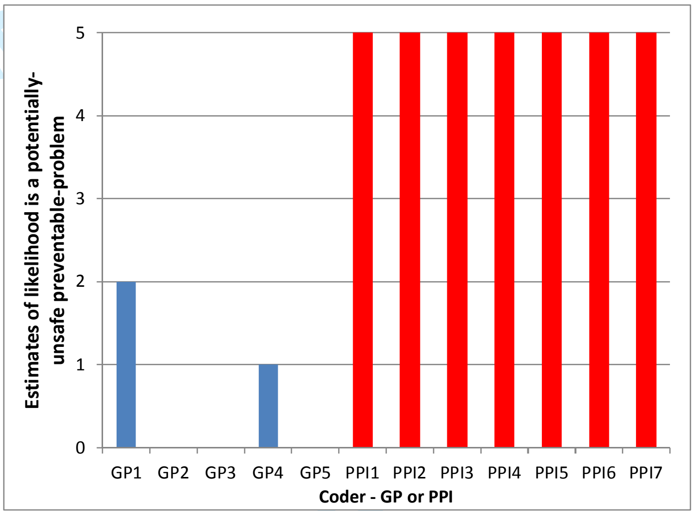

5=very likely or certain, 4=probably, 3=possibly, 2=unlikely, 1=definitely not, 0 = insufficient information or don't know

Patient-reported prospect of harm: prompted via Q10 (Box 1 main paper)

Patient-perspective problem-type code: E2. Procedure was not carried out correctly; D1. Clinician seemed to lack interest in the patient's health problem or did not listen carefully enough

Scenario19. Dental Surgery

Briefly describe the mistake or problem and how it happened. *"I had an infection under my wisdom tooth. They agreed that the only way to solve the problem was to take the tooth out. They gave me an appointment to do this in 6 weeks. I am a type 1 diabetic and the infection was affecting my blood sugars and I was concerned that I would have to go to A&E if my blood sugars continued to rise due to the infection. It would have affected my health if I had not paid to go to a private dentist."*

Could the mistake or problem have been avoided? If so how? *"They could have taken out the tooth straight away. I was happy to wait at the emergency dentist for them to do this."*

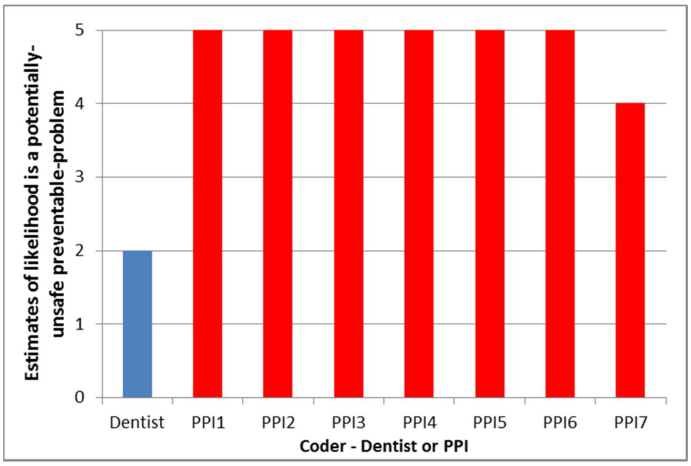

5=very likely or certain, 4=probably, 3=possibly, 2=unlikely, 1=definitely not, 0 = insufficient information or don't know

Were you able to talk about the mistake or problem with anybody working in the primary care service? *"I explained but they said I would have to wait. They also asked if I needed a sugary drink when I said that my sugars were high so I was too scared to eat and had not eaten in 12hrs. It was clear they didn't understand diabetes."*

Patient-reported prospect of harm: health could have been made worse had someone not noticed a problem or error

Patient-perspective problem-type code: A5. Unable to get an appointment/other problems with making appointment

Scenario20. Dental Surgery

Briefly describe the mistake or problem and how it happened. *"Caries, cavities and problem with crown not diagnosed or treated"*

Could the mistake or problem have been avoided? If so how? *"Better dentist & not working to tight time-scale imposed by company owning dental surgery"*

Were you able to talk about the mistake or problem with anybody working in the primary care service? *"No I could not find anybody with whom I could discuss the mistake or problem"*

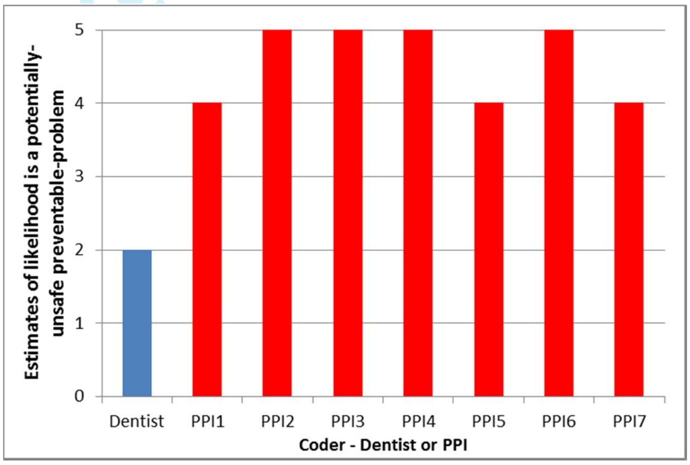

Patient-reported prospect of harm: prompted via Q10 (Box 1 main paper)

5=very likely or certain, 4=probably, 3=possibly, 2=unlikely, 1=definitely not, 0 = insufficient information or don't know

Patient-perspective problem-type code: C3. Problem with dental treatment or diagnosis

Scenario21. GP Surgery

Briefly describe the mistake or problem and how it happened. *"Using the summary on discharge from hospital, one GP transcribed incorrectly on to my electronic notes ie size of ovarian cyst was 7.5cms and he put 7.5 mms. Another GP requested diagnostic bone density scan but either forgot or did not record it and she ended up questioning why I had it and who requested it. She also referred me for an orthopedic consultation then said I was not funded for the steroid injection put into my swollen elbows."*

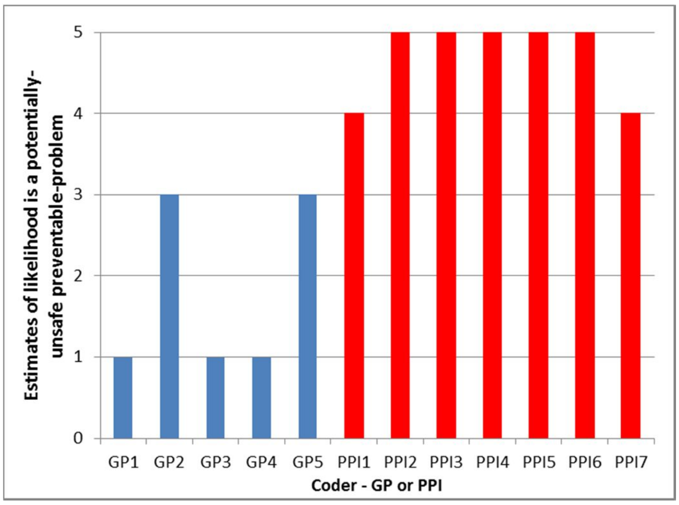

5=very likely or certain, 4=probably, 3=possibly, 2=unlikely, 1=definitely not, 0 = insufficient information or don't know

Could the mistake or problem have been avoided? If so how? *"Yes"*

Were you able to talk about the mistake or problem with anybody working in the primary care service? *"I was too scared to discuss my concerns for fear of being labelled a trouble maker"*

Patient-reported prospect of harm: health could have been made worse had someone not noticed a problem or error

Patient-perspective problem-type code: A2. Incorrect notes/inadequate notes/notes not kept up to date

For peer review

Scenario22. GP Surgery

Briefly describe the mistake or problem and how it happened. *"GP prescribed pills, but then got phone call saying not to take them"*

Could the mistake or problem have been avoided? If so how? *"Not sure"*

Were you able to talk about the mistake or problem with anybody working in the primary care service? *"No I was not concerned about the problem"*

Patient-reported prospect of harm: prompted via Q10 (Box 1 main paper)

Patient-perspective problem-type code: C1. Medication problem

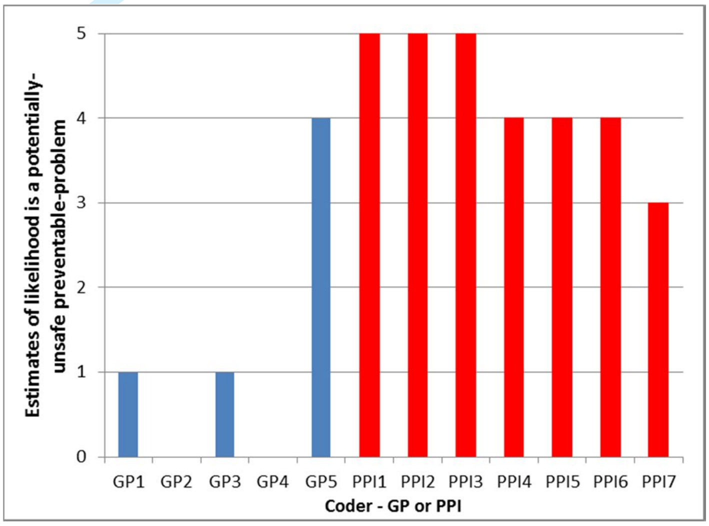

5=very likely or certain, 4=probably, 3=possibly, 2=unlikely, 1=definitely not, 0 = insufficient information or don't know

Scenario23. GP Surgery

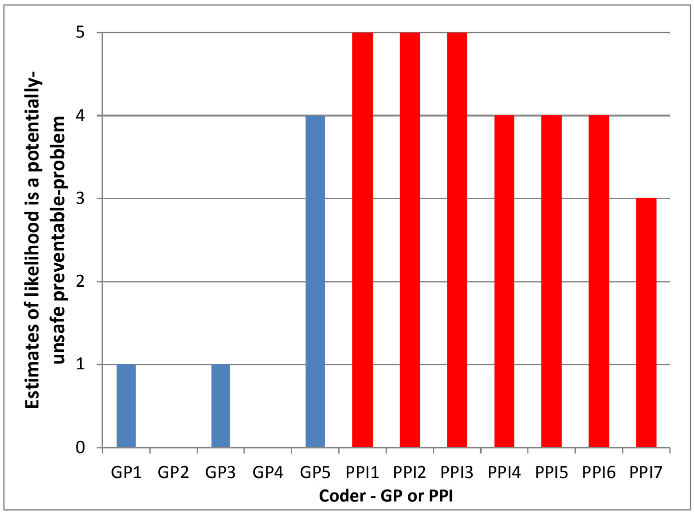

5=very likely or certain, 4=probably, 3=possibly, 2=unlikely, 1=definitely not, 0 = insufficient information or don't know

Briefly describe the mistake or problem and how it happened. *"I had a burst appendix and peritonitis, something that even a scan couldn't detect adequately. My first visit to GP was when I said I think I have appendicitis, no other symptoms only the pain. It was ten days before seeing a consultant, a further 10 days to have a scan, then 2 weeks to be told that I had a lump on my colon which is what my GP had said 5 weeks previously. It was a further 2 weeks before I had surgery."*

Could the mistake or problem have been avoided? If so how? *"If my GP had referred me for a scan immediately it would have saved 3 weeks out of the seven. It was two weeks from scan to results and I hear that is usual, but they're not looking at them for 2 weeks"*

Were you able to talk about the mistake or problem with anybody working in the primary care service? *"Had the outcome been different my widow might have pursued the matter further. The system is at fault rather than any individual."*

Patient-reported prospect of harm: your health has been made worse by a problem or error that could have been prevented

Patient-perspective problem-type code: B5. Not referred when patient felt was needed

For peer review only

STROBE Statement—Checklist of items that should be included in reports of ***cross-sectional studies***

	Item No	Recommendation
**Title and abstract**	1	(*a*) Indicate the study's design with a commonly used term in the title or the abstract Yes p1
		(*b*) Provide in the abstract an informative and balanced summary of what was done and what was found yes p2
**Introduction**		
Background/rationale	2	Explain the scientific background and rationale for the investigation being reported Yes p3
Objectives	3	State specific objectives, including any prespecified hypotheses yes p3-4
**Methods**		
Study design	4	Present key elements of study design early in the paper yes p4
Setting	5	Describe the setting, locations, and relevant dates, including periods of recruitment, exposure, follow-up, and data collection yes p4
Participants	6	(*a*) Give the eligibility criteria, and the sources and methods of selection of participants yes p4
Variables	7	Clearly define all outcomes, exposures, predictors, potential confounders, and effect modifiers. Give diagnostic criteria, if applicable yes box1, online appendix 1
Data sources/ measurement	8*	For each variable of interest, give sources of data and details of methods of assessment (measurement). Describe comparability of assessment methods if there is more than one group yes p5, online appendix 1
Bias	9	Describe any efforts to address potential sources of bias yes p4
Study size	10	Explain how the study size was arrived at n/a as is a pilot study.
Quantitative variables	11	Explain how quantitative variables were handled in the analyses. If applicable, describe which groupings were chosen and why yes p5, table2
Statistical methods	12	(*a*) Describe all statistical methods, including those used to control for confounding yes p5
		(*b*) Describe any methods used to examine subgroups and interactions, yes just chi2 tests p5
		(*c*) Explain how missing data were addressed all missing data is listed in the tables so it is completely transparent how this was dealt with, there were few missing data
		(*d*) If applicable, describe analytical methods taking account of sampling n/a
		(*e*) Describe any sensitivity analyses n/a
**Results**		
Participants	13*	(a) Report numbers of individuals at each stage of study—eg numbers potentially eligible, examined for eligibility, confirmed eligible, included in the study, completing follow-up, and analysed yes online appendix 1
		(b) Give reasons for non-participation at each stage yes online appendix 1
		(c) Consider use of a flow diagram yes online appendix 1
Descriptive data	14*	(a) Give characteristics of study participants (eg demographic, clinical, social) and information on exposures and potential confounders yes table 1
		(b) Indicate number of participants with missing data for each variable of interest yes all tables
Outcome data	15*	Report numbers of outcome events or summary measures yes all tables
Main results	16	(*a*) Give unadjusted estimates and, if applicable, confounder-adjusted estimates and their precision (eg, 95% confidence interval). Make clear which confounders were

For peer review only

		adjusted for and why they were included yes table 3
		(*b*) Report category boundaries when continuous variables were categorized yes all tables
		(*c*) If relevant, consider translating estimates of relative risk into absolute risk for a meaningful time period not appropriate as pilot study with self-selected sample
Other analyses	17	Report other analyses done—eg analyses of subgroups and interactions, and sensitivity analyses table 6 considers demographics for problems more likely to be a potentially harmful.
**Discussion**		
Key results	18	Summarise key results with reference to study objectives yes p7
Limitations	19	Discuss limitations of the study, taking into account sources of potential bias or imprecision. Discuss both direction and magnitude of any potential bias yes p8
Interpretation	20	Give a cautious overall interpretation of results considering objectives, limitations, multiplicity of analyses, results from similar studies, and other relevant evidence yes p7-8
Generalisability	21	Discuss the generalisability (external validity) of the study results yes p8, not generalisable
**Other information**		
Funding	22	Give the source of funding and the role of the funders for the present study and, if applicable, for the original study on which the present article is based yes p9

*Give information separately for exposed and unexposed groups.

Note: An Explanation and Elaboration article discusses each checklist item and gives methodological background and published examples of transparent reporting. The STROBE checklist is best used in conjunction with this article (freely available on the Web sites of PLoS Medicine at http://www.plosmedicine.org/, Annals of Internal Medicine at http://www.annals.org/, and Epidemiology at http://www.epidem.com/). Information on the STROBE Initiative is available at www.strobe-statement.org.

For peer review only
